# The Liebeskind–Srogl Cross-Coupling Reaction as a Crucial Step in the Synthesis of New Squaramide-Based Antituberculosis Agents

**DOI:** 10.1021/acsomega.4c04314

**Published:** 2024-07-29

**Authors:** Jan Chasák, Laurence Van Moll, An Matheeussen, Linda De Vooght, Paul Cos, Lucie Brulíková

**Affiliations:** † Department of Organic Chemistry, Faculty of Science, Palacký University, 17. listopadu 12, 77146 Olomouc, Czech Republic; ‡ Laboratory of Microbiology, Parasitology and Hygiene (LMPH), S7, Faculty of Pharmaceutical, Biomedical and Veterinary Sciences, University of Antwerp, 2610 Wilrijk, Belgium

## Abstract

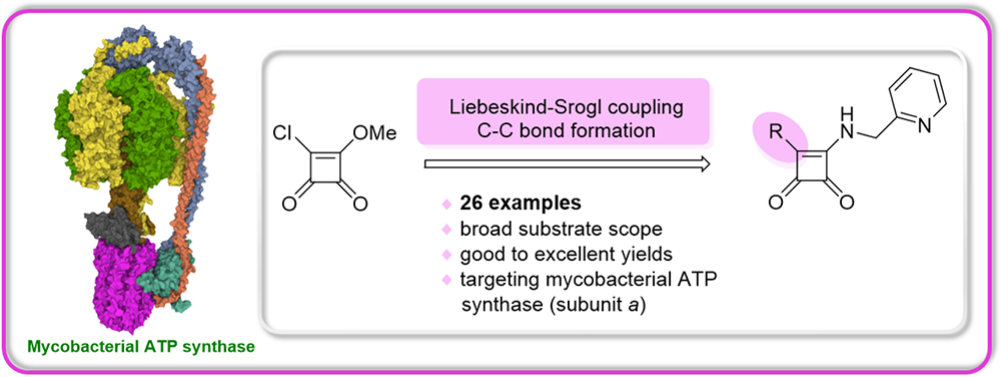

The synthesis of an extensive series of new squaramides with high potential in treating drug-resistant tuberculosis employing the Liebeskind–Srogl cross-coupling reaction is presented. Using the protocol given and various substrates, we assessed the scope and limitations of our methodology and prepared an extensive range of desired compounds. Moreover, ^1^H NMR spectra in solution show the presence of two rotational conformers (rotamers) in special cases. The results of antimycobacterial activity demonstrate the highly selective substrate specificity of the tested squaramides, requiring an efficient and widely applicable synthetic approach needed for the discovery of lead compounds. Our synthetic strategy confirms the versatility of squaramides that can be easily transformed into diverse functionalized molecules.

## Introduction

**Figure 1:**
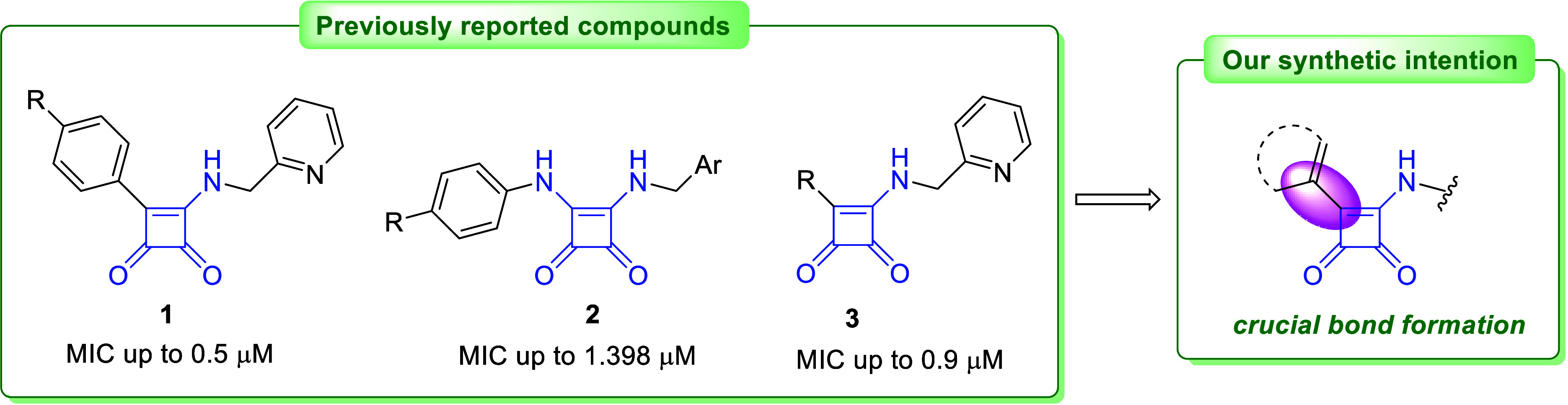
Reported squaramide as an antituberculosis agents and our synthetic intent.^2,4^

Squaric acid (3,4-dihydroxycyclobut-3-ene-1,2-dione) is a unique feature that has received special attention in many research areas, especially in medicinal chemistry.^1^ Many bioactive squaric acid–based heterocycles exhibited exciting antibacterial, antimycobacterial, antimalarial, or cytotoxic activity, among others.^1^ Interestingly, squaric acid analogues showed exceptional properties in the research of antituberculosis drugs. Thus, recently, efforts have increased to synthesize these attractive targets. The early first analogue with the extraordinary biological profile in this sense was published by Tantry and co-workers in 2017.^2^ They reported compounds highly active against *Mycobacterium tuberculosis* acting as a mycobacterial ATP synthase inhibitors (Figure 1, compounds **1**). Tantry et al. also experimentally identified subunit *a* of mycobacterial ATP synthase as the molecular target. This outstanding discovery started research into a new group of ATP synthase inhibitors, especially considering these substances act on a different active site than the FDA-approved ATP synthase inhibitor bedaquiline.^3^ Three years later, Li et al. described squaramides **2** (Figure 1) with additional amine formation as a promising anti-TB lead compound with good anti-TB activity and improved pharmacokinetic and safety profiles through a preliminary draggability evaluation.^4^

Our systematic investigation of various squaramides (Figure 1, compounds **3**) revealed that compounds with aromatic moiety directly bound to the squaric cycle exhibited the best biological profile.^5^ The Friedel–Crafts type reaction was described to form a new C–C bond.^2^ However, this methodology suffered from very low yields and was not applicable to various ranges of substrates. Thus, our team focused on developing a new synthetic approach to nonsymmetrical squaramides to enable the rapid and efficient preparation of extensive libraries for biological screening.

**Figure 2:**
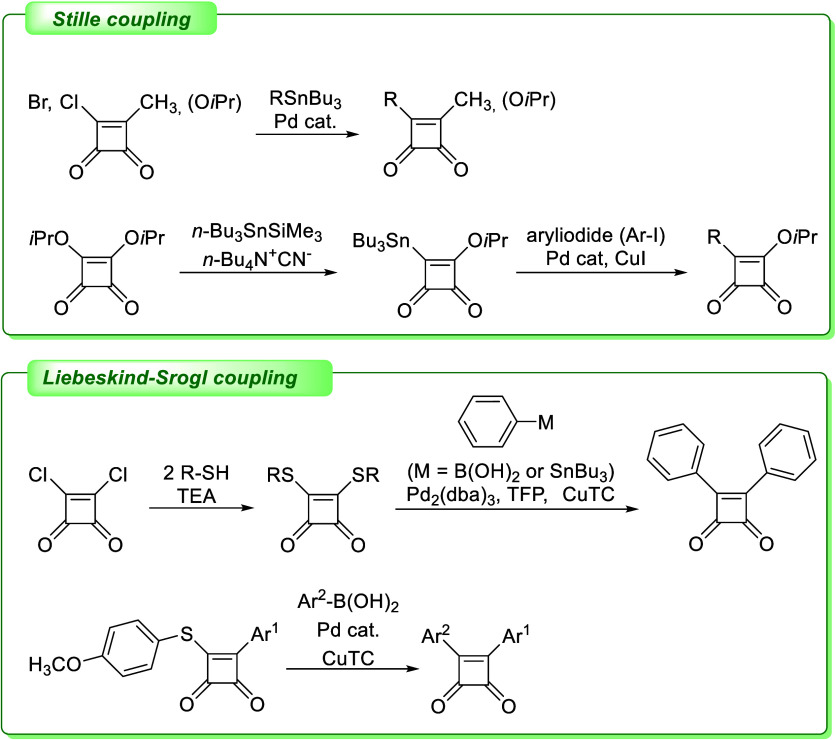
Reported coupling reactions on squaric cycle.^10,11,13,14^

In this context, coupling reactions attracted our interest as powerful synthetic methods. We found out the coupling reactions on squaramides have already received some attention in the past, especially Suzuki-Miyaura,^6^ Stille^7−11^ and Liebeskind–Srogl^11−15^ cross-coupling reactions (Figure 2). While Suzuki-Miyaura coupling failed on the cyclobutenedione molecules, Liebeskind–Srogl coupling and Stille coupling proved to be much more successful.

The first report on Stille coupling on squaramides was published in 1990,^10^ where the authors described the reaction of squaric acid chloride or bromide with various organotin reagents under palladium catalysis (Figure 2). In the same year, a very similar publication was released.^11^ Authors presented the possibility of the opposite reaction arrangement—the formation of an organotin compound from a cyclobutenedione component followed by a reaction with various organic iodides.

The Liebeskind–Srogl coupling stands out among the three mentioned cross-coupling reactions due to its unique reaction mechanism based on the formation of a suitable thio derivative for the coupling reaction. This uniqueness in connection with squaric acid derivatives was first discussed in 2007.^13,14^ Appropriate thio compound synthesized from dichlorocyclobutenedione undergoes efficient cross-coupling with boronic acids to produce functionalized bisarylocyclobutenediones.^13^ Moreover, appropriate substrate enabled the preparation of cyclobutenediones selectively substituted by two different aryls via the combination of Liebeskind–Srogl and Stille coupling.^14^ Final compounds were obtained upon the initial Stille coupling on the “chloride side” of the central skeleton, followed by the Liebeskind–Srogl coupling on the “thioester side”.

In the current work, we were inspired by the synthetic achievements mentioned above and used sulfur-based electrophile as readily available and stable starting material to excess the extensive library of new compounds with a high potential to act as antimycobacterial agents. Compared to the original Friedel–Crafts type reaction,^2,5^ this synthetic approach brings a number of significant advantages, such as wide substrate variability and readily available starting materials. Moreover, the Liebeskind–Srogl Pd^0^/Cu^I^-mediated coupling has several synthetic advantages, such as the mild and nonbasic reaction conditions and high specificity for thioorganic compounds. Through previous investigations, we uncovered the molecular target of squaramides. We discovered that squaramides bind very specifically to the subunit *a* of mycobacterial ATP synthases, and the antimycobacterial activity highly depends on the modification of the squaramide side. Consequently, developing a versatile synthetic approach became imperative to facilitate the preparation of a diverse array of squaramide compounds. The manuscript outlines such a methodology, which enabled us to synthesize a broad spectrum of desired compounds.

## Results and Discussion

### Chemistry

**Scheme 1:**
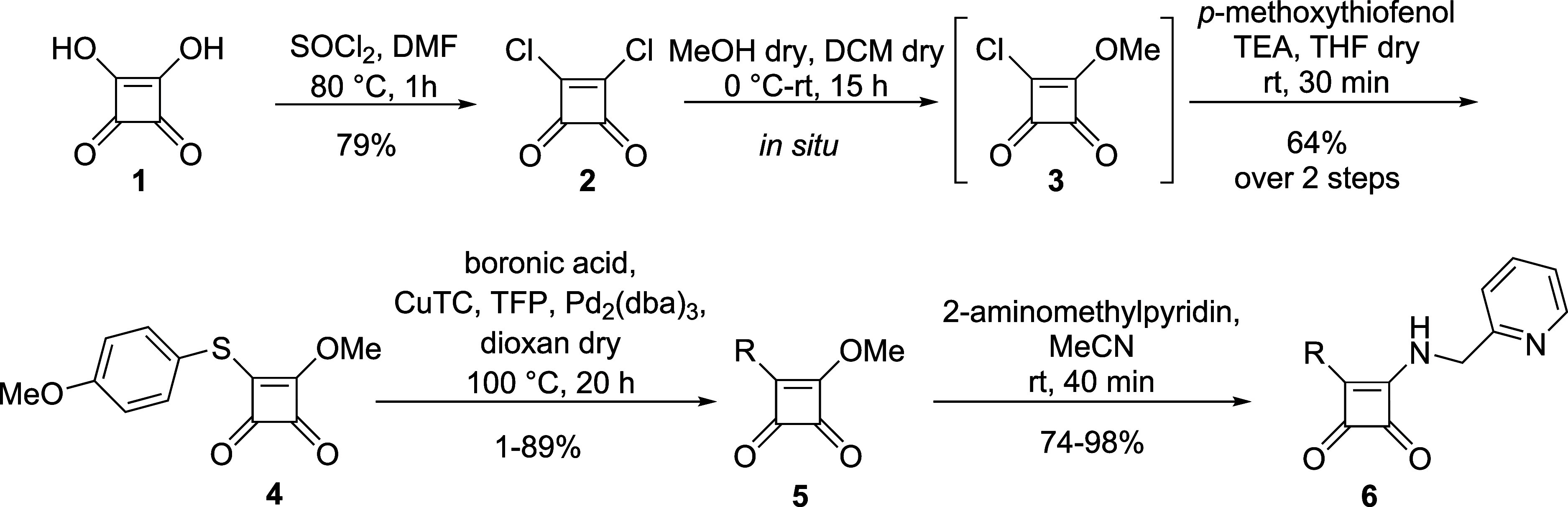
Synthetic Pathway Leading to Squaramides **6**

Based on our knowledge and previous results, we suggested squaramides **6** to be synthesized according to the depicted strategy (Scheme 1). Our synthetic effort leading to the target compounds **6** proceeded from commercially available squaric acid **1**. First, dichloride **2** was prepared and converted to the crucial pseudothioester **4**. With this key intermediate **4**, the cross-coupling reaction with boronic acid was carried out. The obtained precursor **5** was then substituted with 2-(aminomethyl)pyridine to afford the final derivative **6**. Further, the key steps of the synthetic pathway will be discussed in detail.

**Scheme 2:**
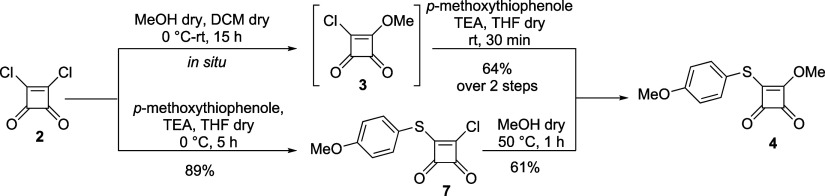
Substitution Reaction Leading to the Pseudothioester **4**

The pseudothioester **4** could be synthesized from the dichloride **2** via two reaction sequences (Scheme 2). During the first pathway, dichloride **2** reacted with dry MeOH, giving intermediate **3***in situ,* followed by the reaction with *p*-methoxythiophenol. On the other hand, dichloride **2** was substituted with *p*-methoxythiophenol, followed by methanolysis. Finally, the first reaction pathway was selected mainly for practical reasons because the intermediate **3** does not need to be isolated.

The crucial reaction of our methodology consisted of the Liebeskind–Srogl coupling reaction. We were inspired by reaction conditions published by Aguilar–Aguilar et al.^14^ (using 2.5 equiv. R-B(OH)_2_, 1 mol % Pd_2_(dba)_3_, 3 mol % TFP, 3 equiv. CuTC, THF, 55 °C) that had to be optimized for our reaction sequence (Table 1). To achieve the best reaction conditions for a diverse range of substrates, we started the optimization process with a set of organoboronic acids, including electron-donating as well as electron-withdrawing substituents. A total of four types of reaction conditions were tested (Table 1). We started our investigation under the described reaction conditions^14^ (conditions 1), which were successful only for electron-activated substrate (entry 1). When we performed this reaction under microwave irradiation (conditions 2) for 1 h at 200W, the conversion of the electron-activated substrate was only 50%. A longer reaction time was not desirable when using microwave irradiation. For this reason, we decided to increase the reaction temperature and selected dioxane as a suitable solvent (conditions 3 and 4). Temperature increasing to 100 °C led to a reaction of electron-deactivated substrates, but uncomplete conversion was observed in the case of 3,4-difluorophenylboronic acid (conditions 3). We tried to increase the conversion using a microwave reactor, but the reaction time of 1 h was insufficient. We did not prolong the reaction time since our experiences showed that longer microwave irradiation might lead to undesired side products.

**Table 1:**
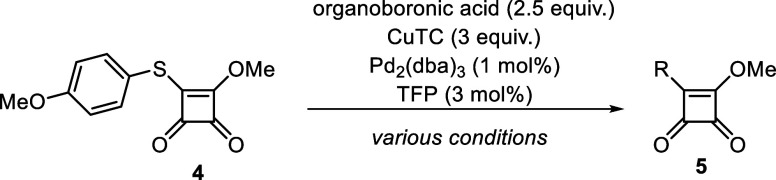

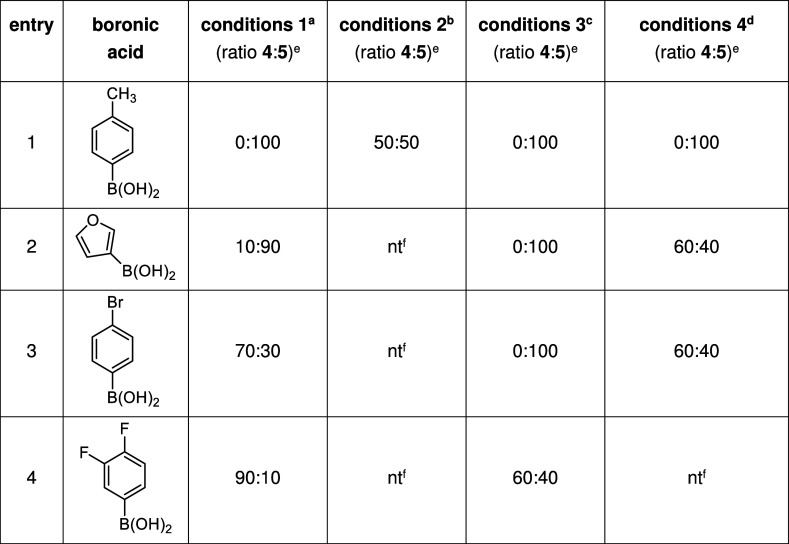
Optimization of the Reaction Conditions for the Cross-Coupling Reaction

a Reaction conditions 1:50 °C, 20 h, THF dry.

b Reaction conditions 2: MW, 200 W, 50 °C, 1 h, THF dry.

c Reaction conditions 3:100 °C, 20 h, dioxane dry.

d Reaction conditions 4: MW, 200 W, 100 °C, 1 h, dioxane dry.

e The ratio of the starting material **4** to the desired product **5** detected by LC-MS analysis (except 3,4-difluorophenyl)boronic acid, where the result of the reaction was evaluated by TLC due to the same retention times of starting material **4** and product **5** in UV/vis spectrum of the LC-MS analysis).

f nt = not tested

With the optimal reaction conditions established (conditions 3), the scope of this protocol was examined (Table 2). The cross-coupling reaction was tested on a wide range of substrates. A total of thirty-seven different boronic acids were coupled with the pseudothioester **4**, giving the intermediates **5** in the yields up to 89%. The electron-donating groups on the scaffold of the boronic acid facilitated the coupling reaction, and the products of those reactions were obtained in higher yields. However, two intermediates (**5aa**, **5ab**) were obtained in very low yield (about 1%), and some boronic acids were coupled unsuccessfully (**5ac**-**5ak**).

**Table 2:**
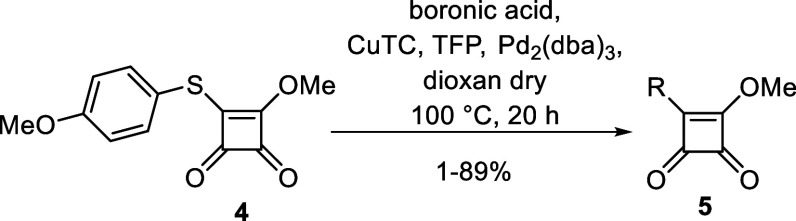

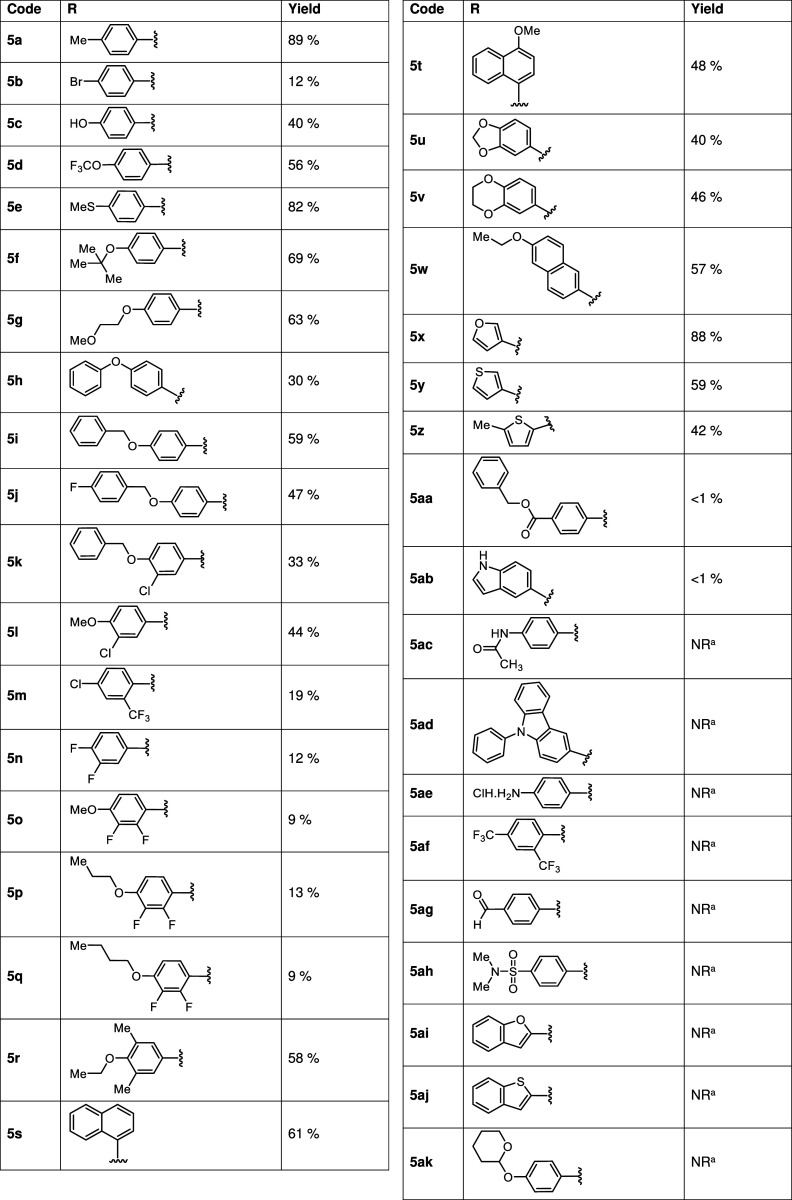
Substrate Scope Screening for the Liebeskind–Srogl Coupling Reaction

a NR = no reaction.

We proved that this key step in the sequence, the Liebeskind–Srogl cross-coupling, is highly dependent on the electron activation of the boronic acid. The coupling reaction proceeded smoothly with activated systems, and the products were usually isolated in decent yields. However, coupling reactions with boronic acids containing electron-withdrawing substituents proved difficult in some cases. In these instances, a higher percentage of homocoupling product was observed. In our specific examples, nitrogen heteroatoms further complicated the reaction, likely due to the free electron pair. These cases typically resulted in a complex reaction mixture with many different products. The desired product was often present in minimal concentration, if at all, and its purification proved very difficult. The fact that the nitrogen heteroatoms on the boronic acid scaffold were problematic and unsuitable for Liebeskind–Srogl coupling under these conditions was generally apparent from our substrate study depicted in Table 2. The proposed derivatives **5ac**-**5ak** were not prepared, even with increased amounts of Pd_2_(dba)_3_ and TFP (up to 5 and 15%) or with the addition of Co(OAc)_2_ (up to 2 equiv).

The last step of the reaction sequence consisted of substituting 2-(aminomethyl)pyridine as a relatively simple and straightforward reaction (Scheme 1). In all cases, 2-(aminomethyl)pyridine was added to the reaction mixture in an equivalent amount to intermediate **5**. Complete conversion to the desired product **6** was always detected after 40 min. Most of the final compounds **6** precipitated in the reaction mixture and were filtrated. Interestingly, derivatives detected as rotameric mixtures employing NMR spectroscopy (compounds **6m**, **6o**–**q**, **6s**, **6t**; will be described further) did not precipitate. The reaction mixture was evaporated under reduced pressure, and products were purified by LC. According to this methodology, we successfully prepared a series containing 26 aryl-substituted squaramides with both electron-donating and electron-withdrawing groups (Table 3) that were further subjected to biological screening.

**Table 3:**
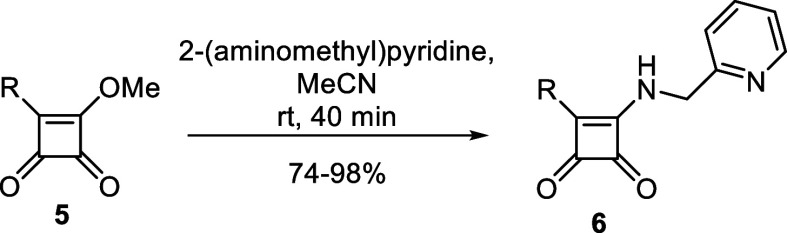

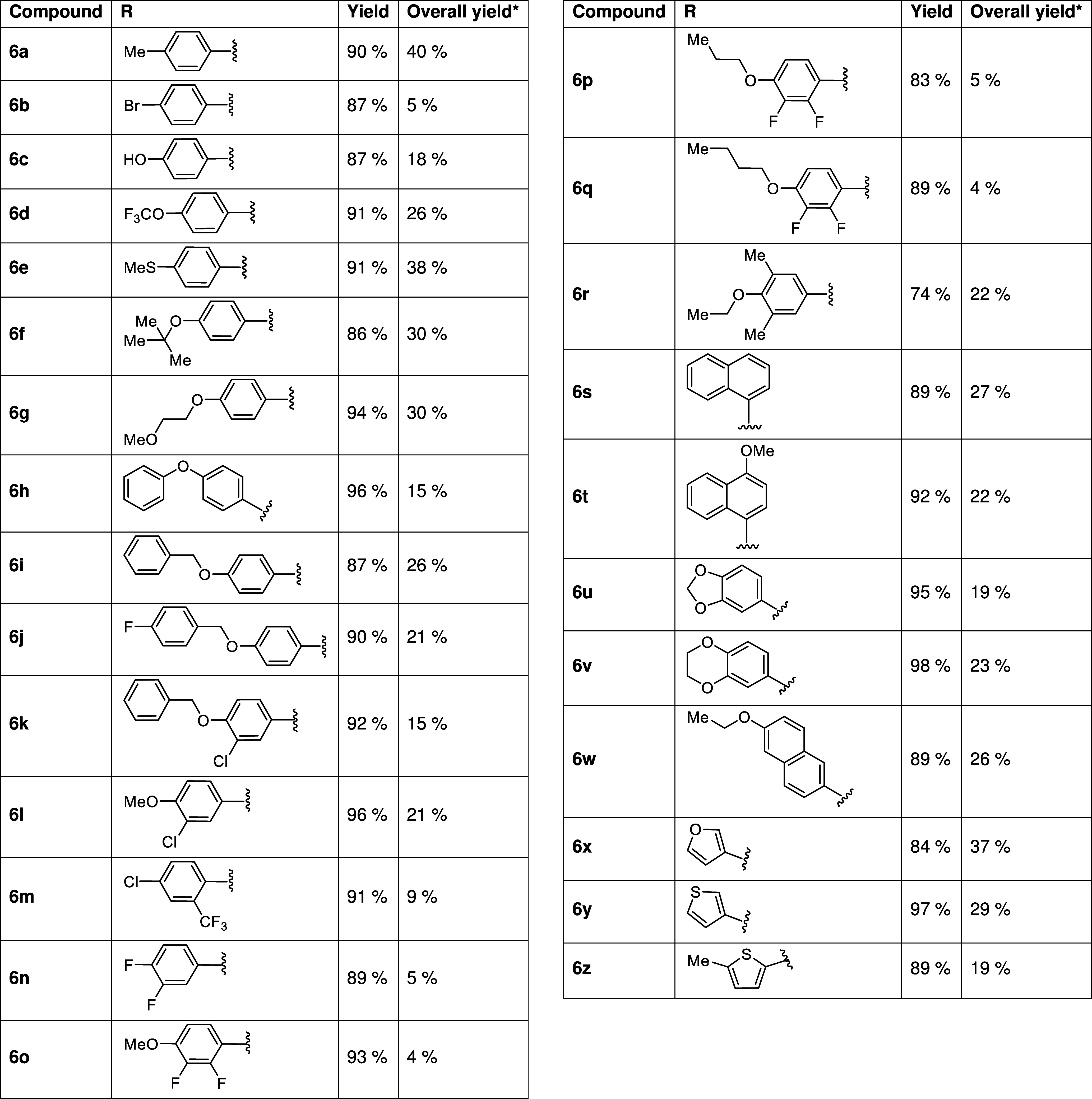
Overview of Final Products

a Overall yield is calculated from the starting squaric acid and covers all steps of the reaction sequence.

**Figure 3:**
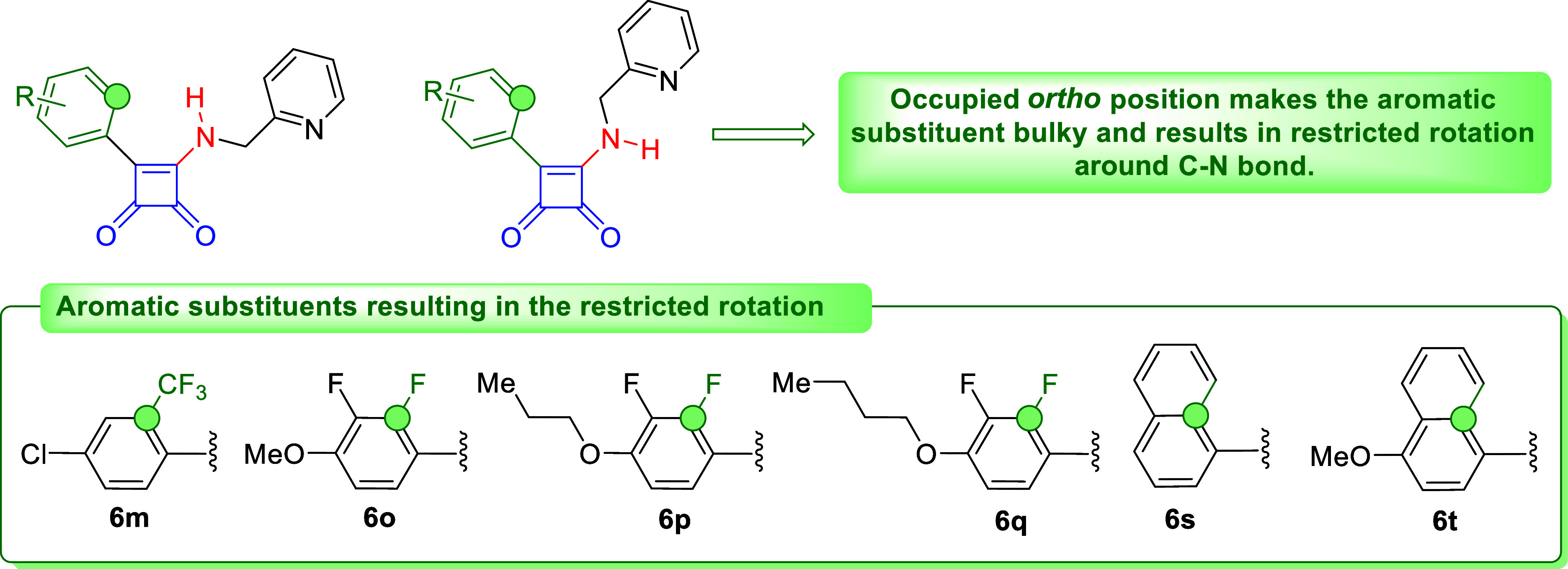
Depiction of conformational changes on “ortho-substituted” analogues **6**.

Interestingly, when NMR spectra were measured to fully characterized prepared compounds, the mixture of rotamers was observed in specific cases (Figure 3). A few papers discussed the conformational preferences of specific squaramides in the past, but this fact has never been described in such substrates synthesized in this work. Assuming the existence of amide-like restricted rotation around the C–N bond of a squaramide, specific squaramides **6** might exist, in principle, as a mixture of two rotamers (Figure 3). Obviously, the restricted rotation around the C–N bonds of squaramides **6** was observed in case of ortho-substituted aromatic substituents (compounds**6m**, **6o**–**q**, **6s**, **6t**). We distinguished both rotameric forms for all these compounds and determined their ratio using NMR spectroscopy.

**Figure 4:**
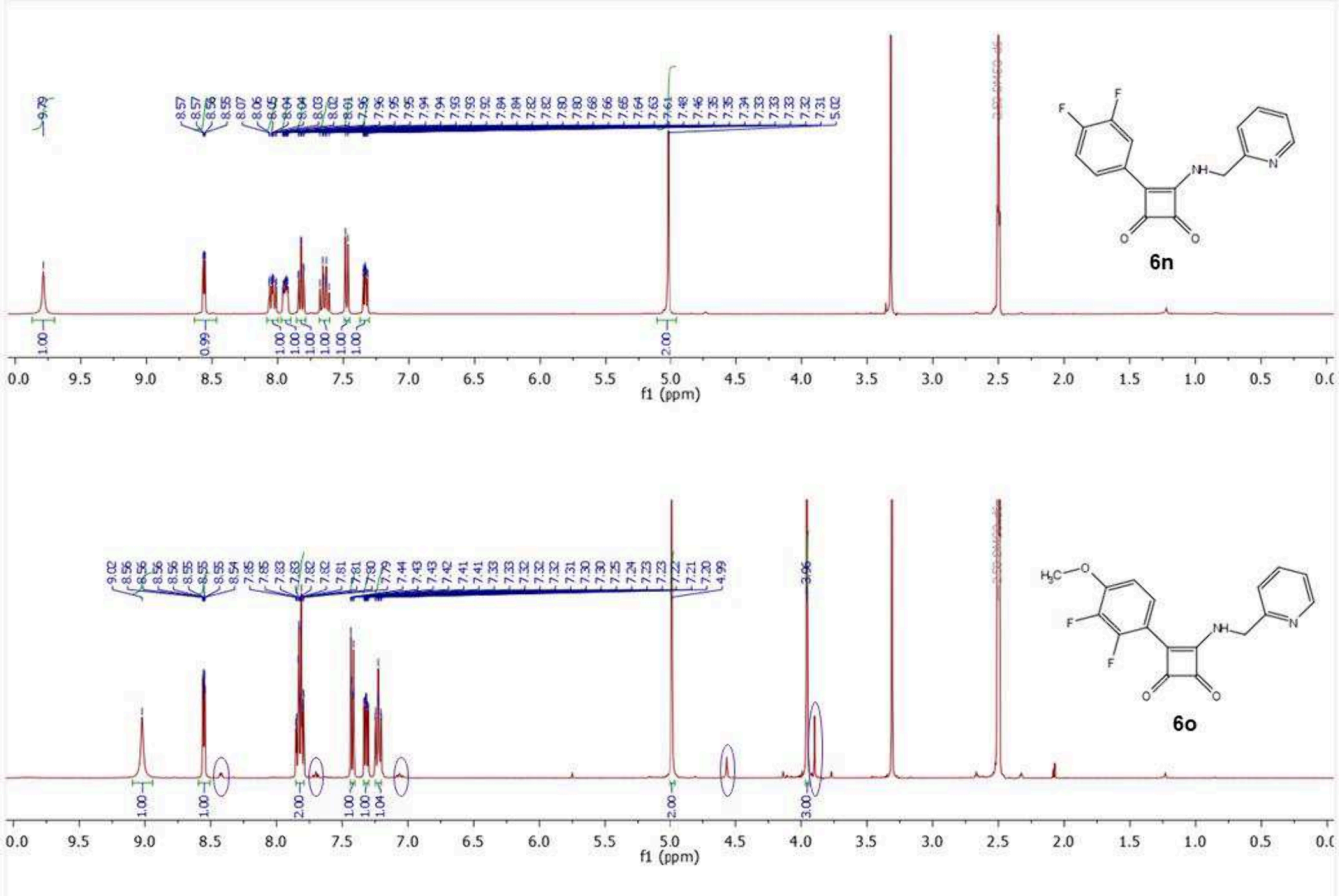
^1^H NMR spectra of compounds **6n** and **6o**.

**Figure 5:**
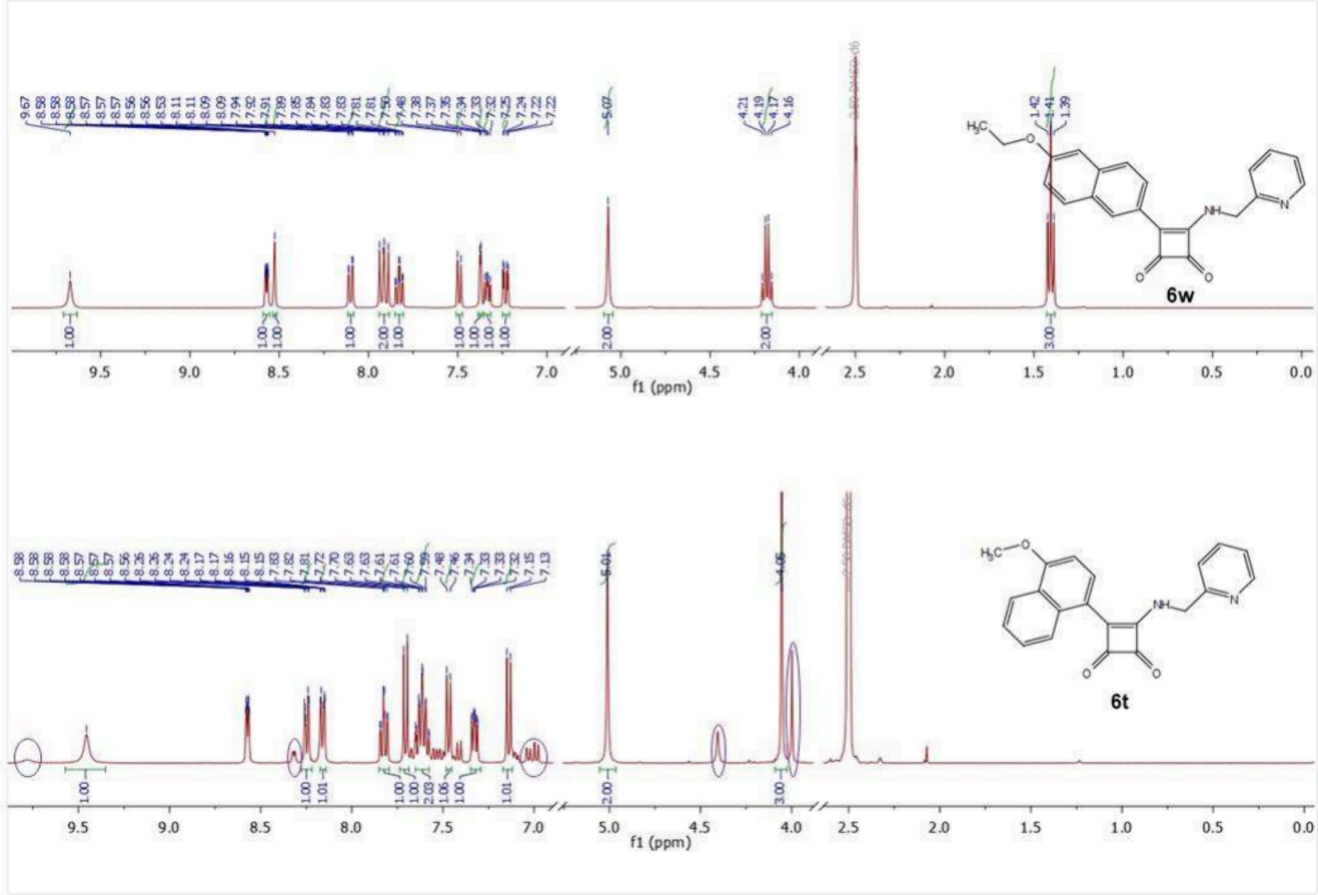
^1^H NMR spectra of compounds **6w** and **6t**.

To demonstrate the specific conformational behavior of “*ortho*-substituted” squaramides **6**, we selected two pairs of similar compounds (one *ortho*-substituted and the other *ortho*-unsubstituted) and discussed their ^1^H NMR spectra (Figure 4 and 5). From the ^1^H NMR analysis of compounds **6n** and **6o** (Figure 4) it is apparent that simple modification of phenyl ring by replacement of small fluorine atom to *ortho* position induces restricted rotation around a C–N bond and detection of the two rotamers for derivative **6o** in ratio 95:5 (minor rotamer signals detected by ^1^H NMR (400 MHz, DMSO-*d*_6_) δ 8.42 (d, *J* = 4.6 Hz), 7.70 (td, *J* = 7.7, 1.8 Hz), 7.06 (m) 4.57 (s), 3.90 (s); highlighted with purple marks).

Another example of the formation of two rotamers in the case of *ortho* substitution is depicted on the compounds **6w** and **6t** (Figure 5). Compound **6t** was detected as a mixture of rotamers in a ratio of 83:17 due to naphthalene substituent with modified *ortho* position. This example proves, that even a part of the aromatic scaffold can be considered as such *ortho* substitution resulting in rotameric mixture detection with characteristic minor rotamer signals: δ ^1^H NMR (400 MHz, DMSO-*d*_6_) δ 9.78 (br s), 8.32 (d, *J* = 4.2 Hz), 7.03 (d, *J* = 7.8 Hz), 6.99 (d, *J* = 8.0 Hz), 4.41 (s), 3.99 (s); minor rotamer signals are highlighted with purple marks.

**Figure 6:**
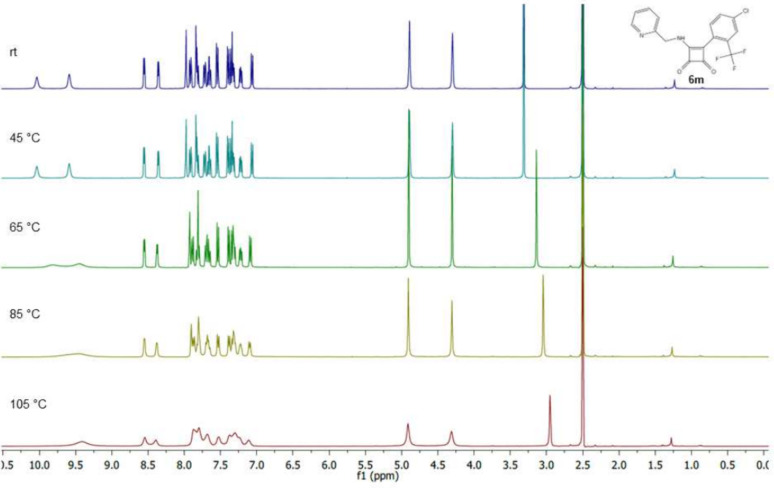
Temperature-dependent ^1^H NMR spectrum of squaramide **6m**.

**Figure 7:**
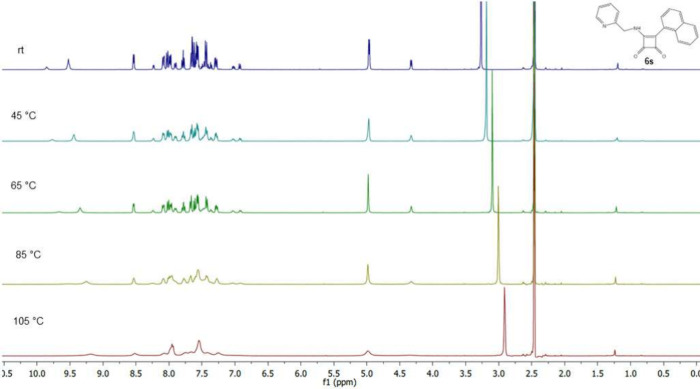
Temperature-dependent ^1^H NMR spectrum of squaramide **6s**.

Further, we performed temperature-dependent ^1^H NMR experiments with compounds **6m** and **6s** (Figure 6 and 7) to demonstrate an ability to overcome the energy barrier and convert one rotamer to another. NMR spectroscopy experiments at different temperatures (25 °C, 45 °C, 65 °C, 85 and 105 °C) have shown that chemical shift for hydrogen atom from NH in the case of compound **6m** (Figure 6) was changed and signals tended to merge (signals around 10 ppm). This fact, in our opinion, is clear evidence of the occurrence of rotamers. However, the complete fusion of the rotamer signals was not observed even at 105 °C, which indicates a high energy barrier between both states. A similar phenomenon was also observed in the ^19^F NMR experiments for the compound **6m**. ^19^F NMR spectra were measured only at 2 temperatures–rt and then at 105 °C. All measured spectra, including NMR experiments performed at higher temperatures, are attached in Supporting Information.

The same trend was observed even in the case of derivative **6s** (Figure 7), where signals tended to merge with the increasing temperature. This trend is apparent again from the chemical shift of hydrogen from the NH group (about 10 ppm) and also from the chemical shift of hydrogens of CH_2_ group of the 2-(aminomethyl)pyridine substituent (about 4–5 ppm).

It should also be mentioned that the measurements of the rotamer mixtures were repeated, at different concentrations of the compound in the deuterated solvent. However, the detected rotamer ratio remained unchanged.

### Antimycobacterial Activity

Our previous investigations revealed specific squaramides to be highly active against *Mycobacterium tuberculosis* and act as mycobacterial ATP synthase inhibitors.^5^ We investigated the spontaneous resistant mutants and revealed a single point mutation within the *atpB gene* responsible for encoding the ATP synthase subunit *a*. This fact was quite interesting since the only known and approved mycobacterial ATP synthase inhibitor bedaquiline acts in different active sites.^5^ For this reason, we decided to assess the antimycobacterial effect of the newly prepared derivatives against *Mtb* H37Ra and broaden the SAR study as a follow-up to previous research. Since there are significant differences between various mycobacterial strains, we also decided to test our compounds against *Mycobacterium abscessus*. *M. abscessus* is a fast-growing bacterium, while *Mtb* is a slow-growing bacterium. Importantly, *M. abscessus* is well-known for its high resistance profile against antibiotics. For this reason, revealing a novel derivative highly active against *M. abscessus* is quite challenging.

All prepared compounds were *in vitro* evaluated for their antimycobacterial activity against the *Mtb* H37Ra and *M. abscessus* strains. While none of the compounds tested showed any activity against *M. abscessus*, 14 compounds showed activity against *Mtb* (Table 4). Moreover, we also assessed the cytotoxicity (IC_50_) of all synthesized intermediates **5** and final compounds **6** on MRC-5 cell lines. The results revealed no detectable cytotoxic effects (IC_50_ higher than 64 μM**)** associated with the most potent compounds **6** depicted in Table 4.

**Table 4:**
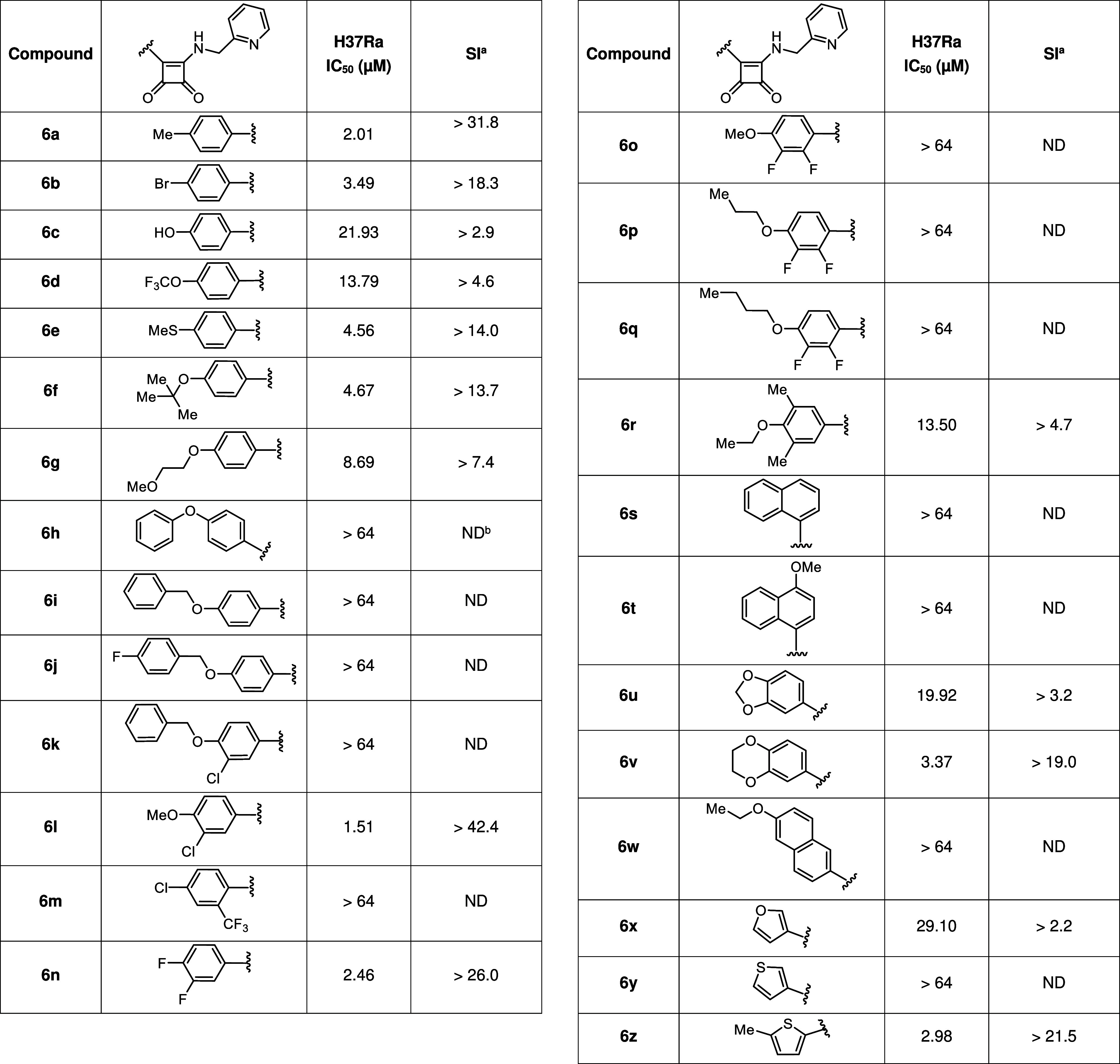
Activity of Final Compounds **6** against *Mycobacterium tuberculosis*, Strain H37Ra, Expressed as IC_50_ and Their Selectivity Index (SI)

a The selectivity index (SI) of the compounds was calculated with the formula CC_50_/IC_50_.

b ND = not determined

**Figure 8:**
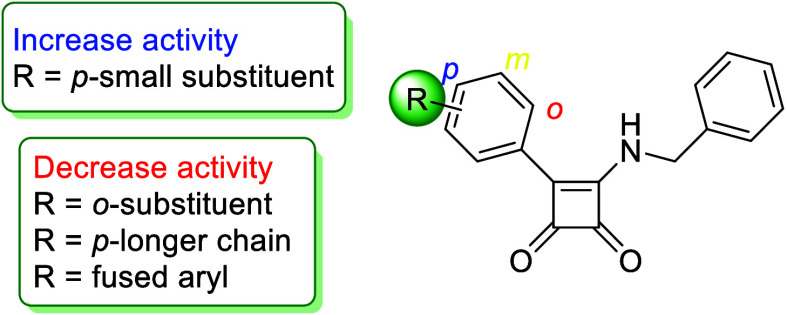
Structure–activity relationship for squaramides **6** against *Mtb* H37Ra.

A tremendous difference in activity was found for the tested compounds, depending on the nature of the aromatic substituent (Figure 8). Compounds with a simple substituent in the *para*-position showed the most exciting results (**6a**–**6g**, **6l**, **6n**). On the other hand, derivatives containing a longer and more complex chain were completely inactive (**6h**–**6k**, **6p**, **6q**, **6w**). Interesting differences can also be observed in a number of heterocyclic substituents (**6x**–**6z**), where the thiophene squaramide **6y** showed the most exciting results.

## Conclusion

In summary, we have developed a versatile and practical Pd-catalyzed Liebeskind–Srogl coupling reaction capable of forming a C–C bond between the squaric acid cycle and desired substrates. The reaction utilizes readily available and stable sulfur-based electrophile and commercially available boronic acids. With its broad substrate scope, we believe that our synthetic pathway will significantly expand the utilization of squaramides as effective antimycobacterial agents and will allow a broad insight into SAR studies with potency to find a suitable lead for the new drug discovery.

## Experimental Section

### Materials and Methods

Solvents and chemicals were purchased from Sigma-Aldrich (St. Louis, Missouri, USA) or Fluorochem (UK). The polystyrene resins were purchased from Aapptec (Canada). The synthesis was performed on Domino Blocks in disposable polypropylene reaction vessels obtained from Torviq (Niles, MI). Analytical thin-layer chromatography (TLC) was performed using aluminum plates precoated with silica gel 60 F254.

The LC-MS analyses were carried out on the UHPLC-MS system (Waters). This system consists of UHPLC chromatograph Acquity with a photodiode array detector and single quadrupole mass spectrometer and uses a XSelect C18 column (2.1 × 50 mm) at 30 °C and flow rate of 600 μL/min. The mobile phase was (A) 10 mM ammonium acetate in HPLC grade water and (B) HPLC grade acetonitrile. A gradient was formed from 10% A to 80% B in 2.5 min; kept for 1.5 min. The column was re-equilibrated with a 10% solution of B for 1 min. The ESI source operated at a discharge current of 5 μA, vaporiser temperature of 350 °C and capillary temperature of 200 °C.

NMR ^1^H/^13^C spectra were recorded on JEOL ECX-500SS (500 MHz) or JEOL ECA400II (400 MHz) spectrometer at magnetic field strengths of 11.75 T (with operating frequencies 500.16 MHz for ^1^H and 125.77 MHz for ^13^C) and 9.39 T (with operating frequencies 399.78 MHz for ^1^H and 100.53 MHz for ^13^C) at ambient temperature (∼21 °C). Chemical shifts (δ) are reported in parts per million (ppm), and coupling constants (J) are reported in Hertz (Hz). NMR spectra are recorded at room temperature (21 °C) and referenced to the residual signals of DMSO-d6.

HRMS analysis was performed on LC chromatograph (Dionex UltiMate 3000, Thermo Fisher Scientific, MA, USA) with mass spectrometer Exactive Plus Orbitrap high-resolution (Thermo Fisher Scientific, MA, USA) operating in positive scan mode in the range of 100–1000 *m*/*z*. Electrospray was used as a source of ionization. Samples were diluted to a final concentration of 0.1 mg/mL in a solution of water and acetonitrile (50:50, v/v). The samples were injected into the mass spectrometer following HPLC separation on a Phenomenex Gemini column (C18, 50 × 2 mm, 3 μm particle) using an isocratic mobile phase 80% ACN and 20% buffer (0,01 M ammonium acetate) or 95% MeOH and 5% H_2_O + 0,1% HCOOH) at a flow rate of 0.3 mL/min.

### Synthesis

#### 3,4-Dichlorocyclobut-3-ene-1,2-dione **2**

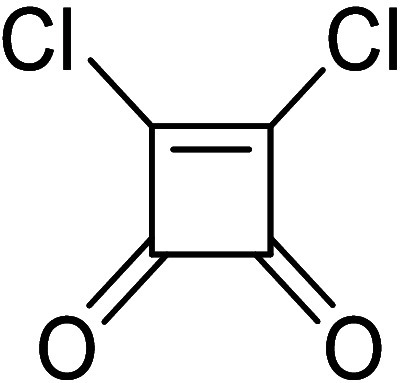



Squaric acid **1** (1 g; 8.77 mmol) was suspended in SOCl_2_ (1.28 mL; 17.54 mmol), and DMF was added in a catalytic amount. The reaction mixture was stirred under reflux at 80 °C for 1 h. After the reaction was complete, SOCl_2_ was evaporated under reduced pressure, and the residue was crystallized from hot hexane. The product was obtained as a yellow solid. Yield: 556–1044 mg (42–79%). ^13^C NMR (101 MHz, CDCl_3_) δ 189.55, 188.24.

#### 3-Methoxy-4-((4-methoxyphenyl)thio)cyclobut-3-ene-1,2-dione **4**

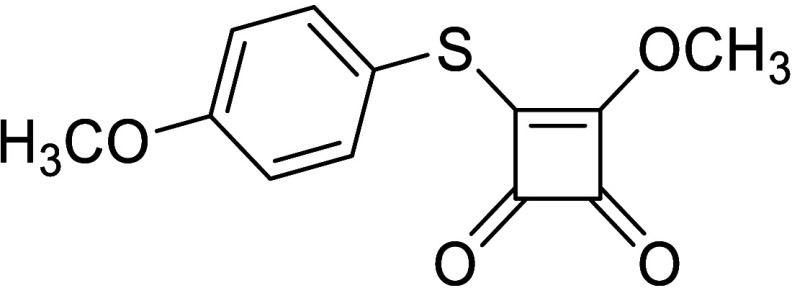



Squaric acid dichloride **2** (500 mg; 3.31 mmol) was dissolved in dry DCM (5 mL). Into the prepared solution, dry MeOH was added dropwisely (134 μL, 3.31 mmol). The reaction mixture was stirred at rt for 15 h. Next, the solvent was evaporated, and the crude product was used for the following reaction step without further purification.

Obtained crude precursor **3** (3.31 mmol theor.) was dissolved in dry THF (5 mL), and the solution was cooled to 0 °C. At this temperature, *p*-methoxythiophenol (407 μL, 3.31 mmol) and TEA (461 μL, 3.31 mmol) were added dropwisely. The prepared reaction mixture was stirred at rt for 30 min. After that time, the obtained triethylammonium salt was filtered. Desired product **4** was purified from the filtrate by column chromatography using DCM as the mobile phase. Product **4** was obtained as a yellow solid. Yield: 348–531 mg (42–64%). ^1^H NMR (400 MHz, CDCl_3_) δ 7.49–7.43 (m, 2H), 6.94–6.89 (m, 2H), 4.36 (s, 3H), 3.84 (s, 3H). ^13^C NMR (101 MHz, CDCl_3_) δ 192.4, 189.5, 187.9, 177.6, 161.3, 135.5, 115.5, 114.9, 61.4, 55.5. HRMS: *m*/*z*: calcd. for C_12_H_11_O_4_S^+^: 251.0373 [M + H]^+^; found: 251.0373.

### General Procedure for the Synthesis of Intermediates **5**

A 50 mL Schlenk flask under N_2_ was charged with compound ***4*** (150 mg; 0.6 mmol), boronic acid (2.5 equiv) and 1,4-dioxane dry (12 mL). Into the solution, CuTC (343 mg; 1.8 mmol), Pd_2_(dba)_3_ (5.5 mg; 0.006 mmol) and TFP (4.2 mg; 0.018 mmol) were then added. The prepared reaction mixture was stirred at 100 °C for 20 h. Then, the reaction solvent was evaporated under reduced pressure. To the obtained residue sat. aq. NH_4_Cl (30 mL) was added, and the mixture was extracted with EtOAc (3 × 30 mL). Combined organic extracts were washed with sat. aq. NH_4_Cl (20 mL) dried over MgSO_4_ and evaporated under reduced pressure. Product **5** was purified from obtained crude material by column chromatography using PE/EtOAc as the mobile phase.

#### 3-Methoxy-4-(*p*-tolyl)cyclobut-3-ene-1,2-dione **5a**

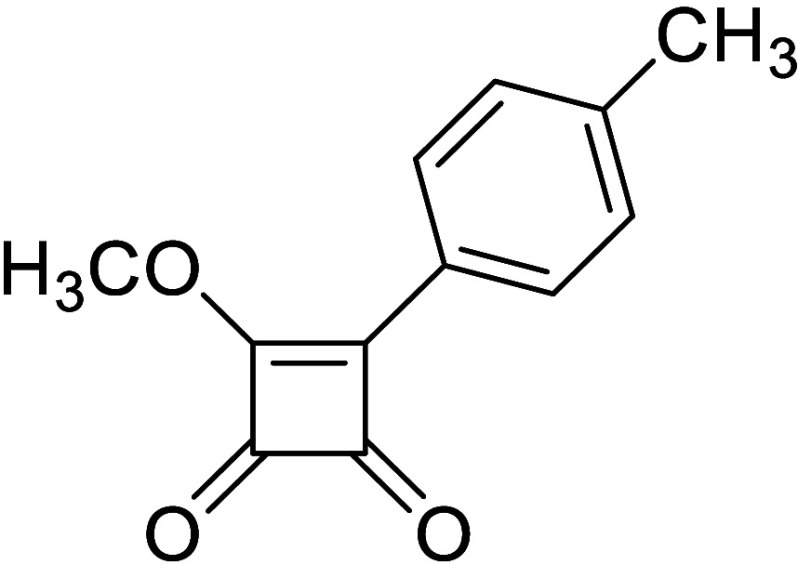



Product **5a** was obtained as a pale-yellow solid. Yield: 108 mg (89%). ^1^H NMR (400 MHz, CDCl_3_) δ 7.96–7.90 (m, 2H), 7.33–7.28 (m, 2H), 4.59 (s, 3H), 2.43 (s, 3H). ^13^C NMR (101 MHz, CDCl_3_) δ 194.4, 193.0, 192.3, 174.1, 144.0, 130.0, 127.9, 125.1, 61.7, 22.1. HRMS: *m*/*z*: calcd. for C_12_H_11_O_3_^+^: 203.0703 [M + H]^+^; found: 203.0707.

#### 3-(4-Bromophenyl)-4-methoxycyclobut-3-ene-1,2-dione **5b**

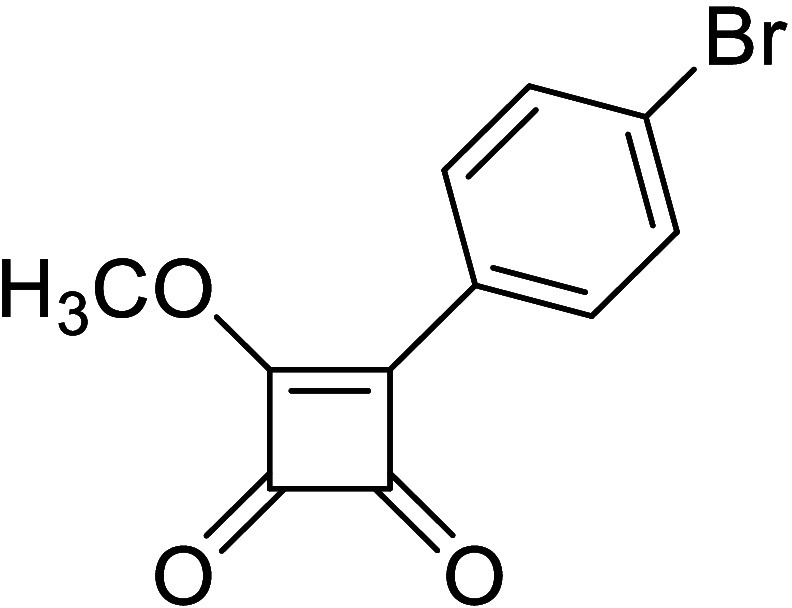



Product **5b** was obtained as a pale-yellow solid. Yield: 19 mg (12%). ^1^H NMR (400 MHz, CDCl_3_) δ 7.92–7.83 (m, 2H), 7.67–7.61 (m, 2H), 4.60 (s, 3H). ^13^C NMR (101 MHz, CDCl_3_) δ 195.0, 192.4, 192.2, 172.5, 132.7, 129.1, 127.8, 126.5, 62.1. HRMS: *m*/*z*: calcd. for C_11_H_8_BrO_3_^+^: 266.9651 [M + H]^+^; found: 266.9654.

#### 3-(4-Hydroxyphenyl)-4-methoxycyclobut-3-ene-1,2-dione **5c**

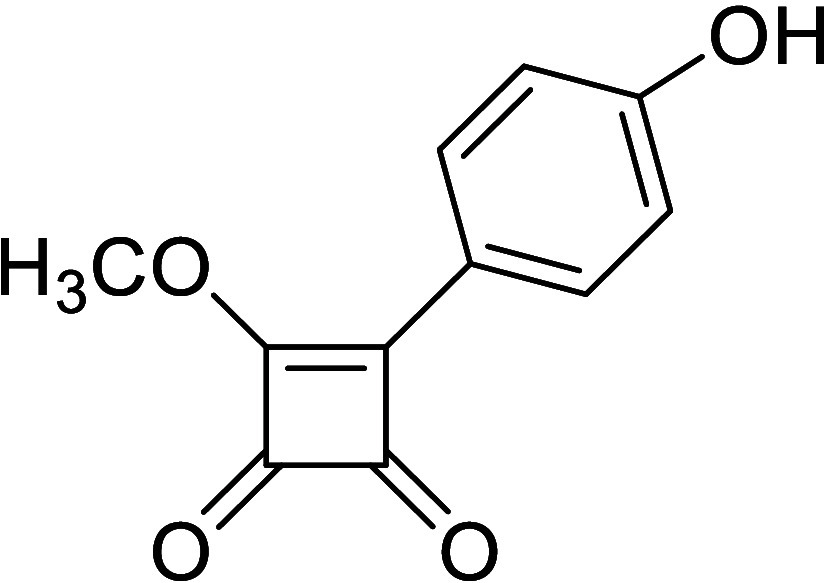



Product **5c** was obtained as a pale-yellow solid. Yield: 49 mg (40%). ^1^H NMR (400 MHz, DMSO-*d*_6_) δ 10.55 (s, 1H), 7.85–7.77 (m, 2H), 7.00–6.93 (m, 2H), 4.49 (s, 3H). ^13^C NMR (101 MHz, DMSO-*d*_6_) δ 193.4, 193.1, 191.0, 171.1, 161.7, 129.4, 118.8, 116.4, 61.7. HRMS: *m*/*z*: calcd. for C_11_H_9_O_4_^+^: 205.0495 [M + H]^+^; found: 205.0498.

#### 3-Methoxy-4-(4-(trifluoromethoxy)phenyl)cyclobut-3-ene-1,2-dione **5d**

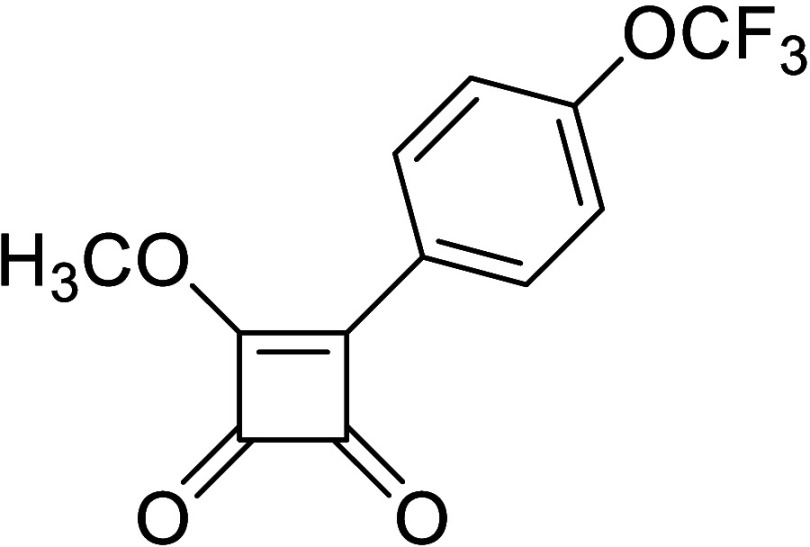



Product **5d** was obtained as a pale-yellow solid. Yield: 91 mg (56%). ^1^H NMR (400 MHz, CDCl_3_) δ 8.12–8.04 (m, 2H), 7.37–7.30 (m, 2H), 4.61 (s, 3H). ^13^C NMR (126 MHz, CDCl_3_) δ 195.0, 192.4, 192.3, 172.1, 152.2, 129.7, 126.1, 121.3, 120.4 (q, *J* = 259.1 Hz), 62.1. HRMS: *m*/*z*: calcd. For C_12_H_6_F_3_O_4_^–^: 271.0218 [M-H]^−^; found: 257.0063 (corresponds to a substance with hydroxyl instead of methoxy group).

#### 3-Methoxy-4-(4-(methylthio)phenyl)cyclobut-3-ene-1,2-dione **5e**

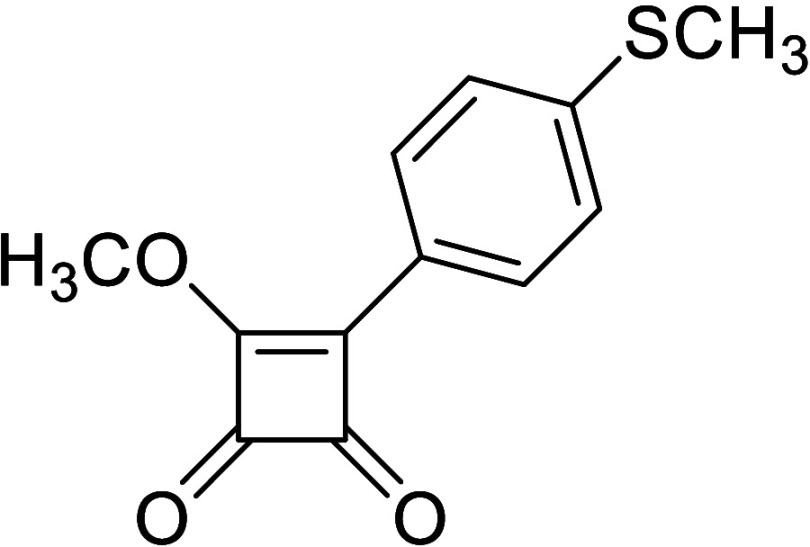



Product **5e** was obtained as a yellow solid. Yield: 115 mg (82%). ^1^H NMR (500 MHz, CDCl_3_) δ 7.95–7.90 (m, 2H), 7.32–7.28 (m, 2H), 4.59 (s, 3H), 2.53 (s, 3H). ^13^C NMR (126 MHz, CDCl_3_) δ 194.1, 192.9, 191.9, 173.4, 146.3, 128.1, 125.6, 123.9, 77.4, 77.2, 76.9, 61.8, 14.9. HRMS: *m*/*z*: calcd. for C_12_H_11_O_3_S^+^: 235.0423; [M + H]^+^; found: 235.0424.

#### 3-(4-(*tert*-Butoxy)phenyl)-4-methoxycyclobut-3-ene-1,2-dione **5f**

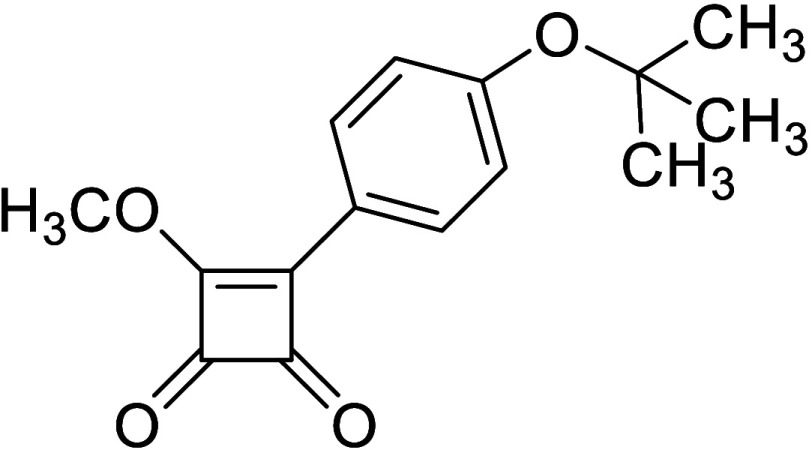



Product **5f** was obtained as a yellow solid. Yield: 108 mg (69%). ^1^H NMR (500 MHz, CDCl_3_) δ 8.00–7.93 (m, 2H), 7.11–7.05 (m, 2H), 4.58 (s, 3H), 1.43 (s, 9H). ^13^C NMR (126 MHz, CDCl_3_) δ 193.7, 193.0, 191.9, 173.7, 160.4, 129.4, 122.9, 122.0, 80.1, 61.6, 29.0. HRMS: *m*/*z*: calcd. for C_15_H_17_O_4_^+^: 261.1121; [M + H]^+^; found: 261.1123.

#### 3-Methoxy-4-(4-(2-methoxyethoxy)phenyl)cyclobut-3-ene-1,2-dione **5g**

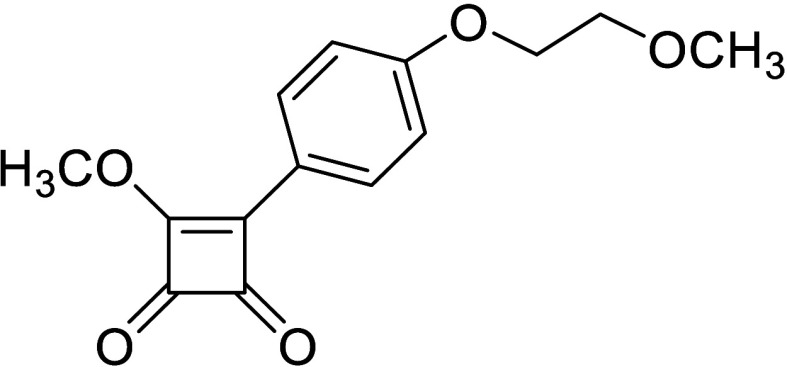



Product **5g** was obtained as a yellow solid. Yield: 99 mg (63%). ^1^H NMR (500 MHz, CDCl_3_) δ 8.03–7.98 (m, 2H), 7.05–7.0 (m, 2H), 4.58 (s, 3H), 4.22–4.17 (m, 2H), 3.81–3.76 (m, 2H), 3.46 (s, 3H). ^13^C NMR (126 MHz, CDCl_3_) δ 193.5, 193.1, 191.7, 173.7, 162.7, 130.2, 120.9, 115.4, 70.9, 67.7, 61.6, 59.4. HRMS: *m*/*z*: calcd. for C_14_H_15_O_5_^+^: 263.0914; [M + H]^+^; found: 263.0916.

#### 3-Methoxy-4-(4-phenoxyphenyl)cyclobut-3-ene-1,2-dione **5h**

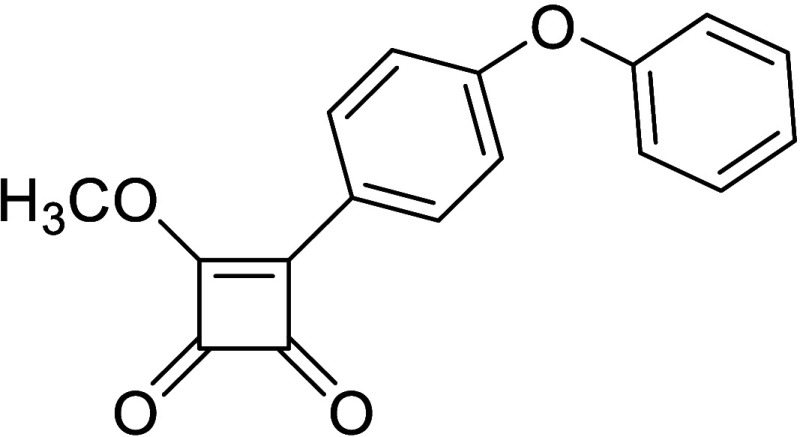



Product **5h** was obtained as a yellow solid. Yield: 51 mg (30%). ^1^H NMR (500 MHz, CDCl_3_) δ 8.04–7.98 (m, 2H), 7.41 (tt, *J* = 7.7, 2.2 Hz, 2H), 7.22 (tt, *J* = 7.2, 1.3 Hz, 1H), 7.10–7.03 (m, 4H), 4.58 (s, 3H). ^13^C NMR (126 MHz, CDCl_3_) δ 193.9, 192.9, 191.9, 173.3, 161.9, 155.3, 130.3, 130.1, 125.0, 122.3, 120.4, 118.2, 61.8. HRMS: *m*/*z*: calcd. for C_17_H_13_O_4_^+^: 281.0808; [M + H]^+^; found: 281.0812.

#### 3-(4-(Benzyloxy)phenyl)-4-methoxycyclobut-3-ene-1,2-dione **5i**

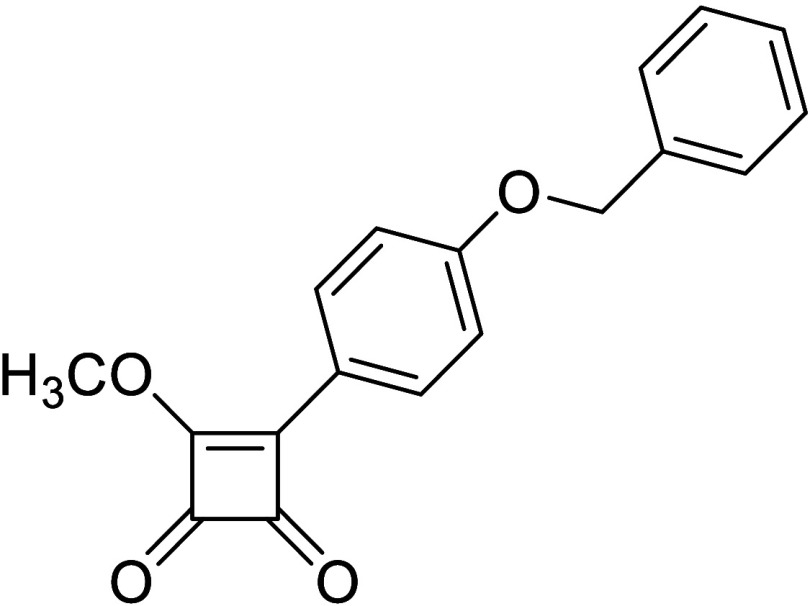



Product **5i** was obtained as a pale-yellow solid. Yield: 104 mg (59%). ^1^H NMR (400 MHz, CDCl_3_) δ 8.07–7.97 (m, 2H), 7.47–7.32 (m, 5H), 7.12–7.03 (m, 2H), 5.14 (s, 2H), 4.57 (s, 3H). ^13^C NMR (101 MHz, CDCl_3_) δ 193.5, 193.0, 191.7, 173.6, 162.5, 136.0, 130.1, 128.8, 128.4, 127.6, 120.9, 115.6, 70.3, 61.6. HRMS: *m*/*z*: calcd. for C_18_H_15_O_4_^+^: 295.0965 [M + H]^+^; found: 295.0963.

#### 3-(4-((4-Fluorobenzyl)oxy)phenyl)-4-methoxycyclobut-3-ene-1,2-dione **5j**

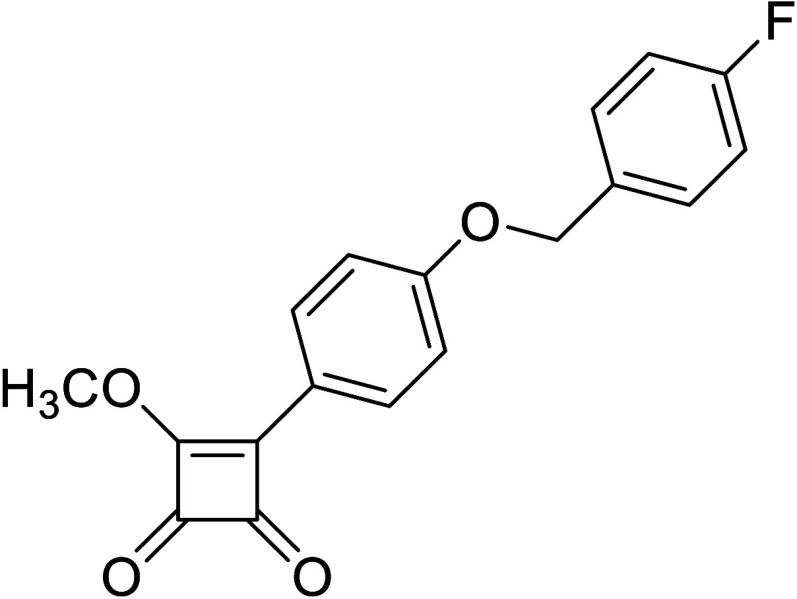



Product **5j** was obtained as a pale-yellow solid. Yield: 88 mg (47%). ^1^H NMR (400 MHz, CDCl_3_) δ 8.05–7.99 (m, 2H), 7.44–7.38 (m, 2H), 7.12–7.04 (m, 4H), 5.10 (s, 2H), 4.58 (s, 3H). ^13^C NMR (126 MHz, CDCl_3_) δ 193.6, 193.0, 191.7, 173.5, 162.8 (d, *J* = 247.1 Hz), 162.3, 131.8 (d, *J* = 3.2 Hz), 130.1, 129.5 (d, *J* = 8.2 Hz), 121.0, 115.8 (d, *J* = 21.9 Hz), 115.6, 69.6, 61.6. HRMS: *m*/*z*: calcd. for C_18_H_14_FO_4_^+^: 313.0871 [M + H]^+^; found: 313.0869.

#### 3-(4-(Benzyloxy)-3-chlorophenyl)-4-methoxycyclobut-3-ene-1,2-dione **5k**

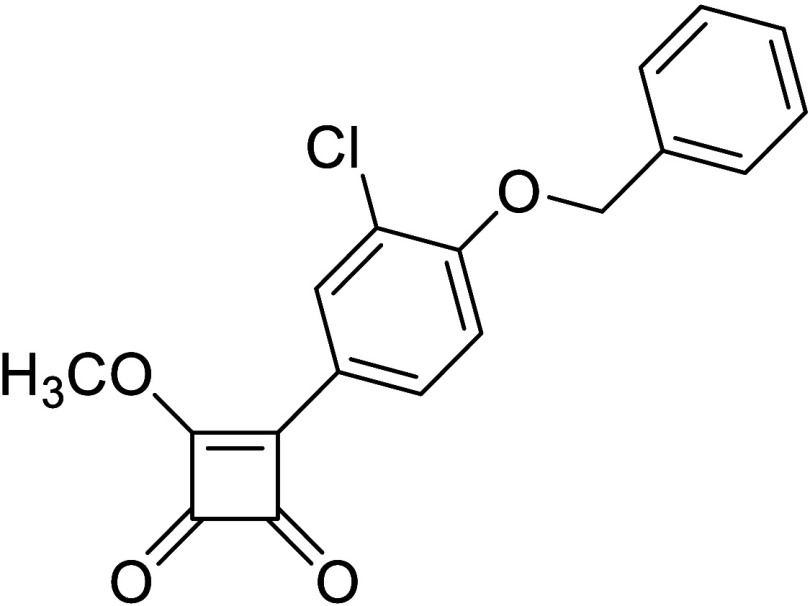



Product **5k** was obtained as a yellow solid. Yield: 65 mg (33%). ^1^H NMR (400 MHz, CDCl_3_) δ 8.05 (d, *J* = 2.0 Hz, 1H), 7.95 (dd, *J* = 8.6, 2.1 Hz, 1H), 7.50–7.31 (m, 5H), 7.06 (d, *J* = 8.6 Hz, 1H), 5.25 (s, 2H), 4.59 (s, 3H). ^13^C NMR (126 MHz, CDCl_3_) δ 193.9, 192.5, 191.6, 172.1, 157.6, 135.5, 129.7, 128.8, 128.4, 128.1, 127.1, 124.3, 121.3, 113.7, 71.0, 61.8. HRMS: *m*/*z*: calcd. for C_18_H_14_ClO_4_^+^: 329.0575 [M + H]^+^; found: 329.0572.

#### 3-(3-Chloro-4-methoxyphenyl)-4-methoxycyclobut-3-ene-1,2-dione **5l**

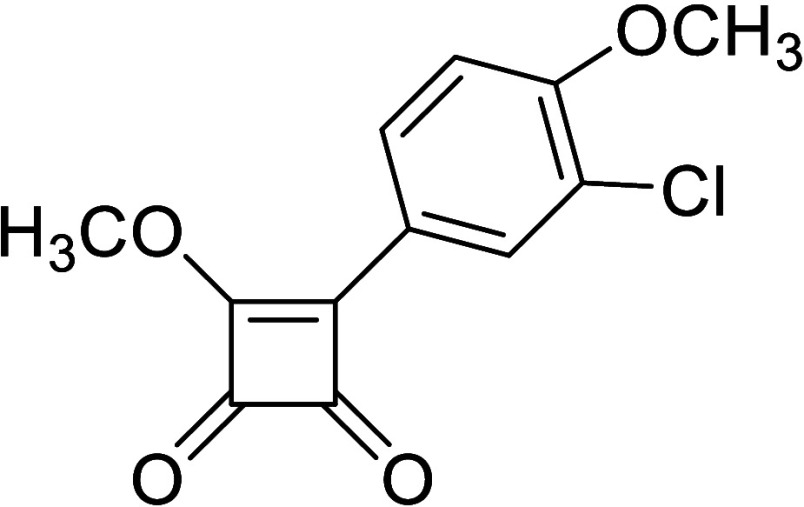



Product **5l** was obtained as a yellow solid. Yield: 67 mg (44%). ^1^H NMR (400 MHz, CDCl_3_) δ 8.05–7.95 (m, 2H), 7.02 (d, *J* = 8.4 Hz, 1H), 4.59 (s, 3H), 3.98 (s, 3H). ^13^C NMR (126 MHz, CDCl_3_) δ 193.9, 192.6, 191.7, 172.2, 158.6, 129.6, 128.4, 123.8, 121.2, 112.3, 61.9, 56.6. HRMS: *m*/*z*: calcd. for C_12_H_10_ClO_4_^+^: 253.0262 [M + H]^+^; found: 253.0260.

#### 3-(4-Chloro-2-(trifluoromethyl)phenyl)-4-methoxycyclobut-3-ene-1,2-dione **5m**

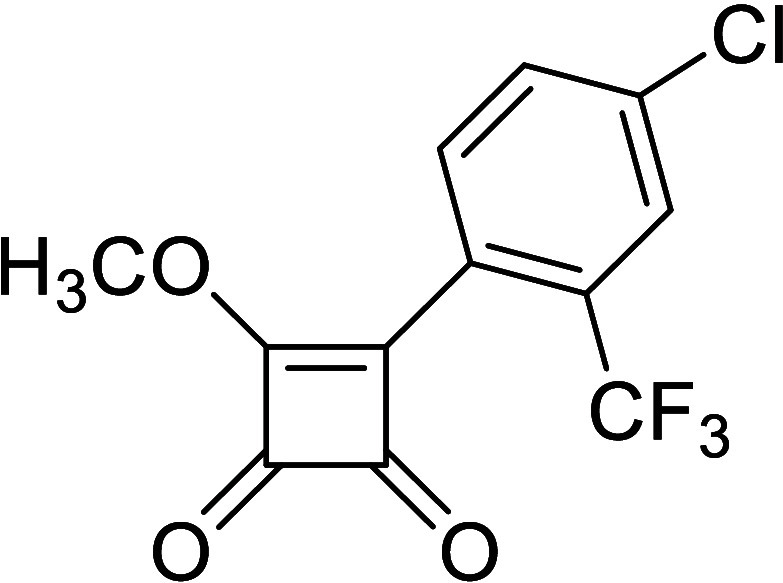



Product **5m** was obtained as a yellow solid. Yield: 33 mg (19%). ^1^H NMR (400 MHz, CDCl_3_) δ 7.79 (d, *J* = 2.1 Hz, 1H), 7.72–7.62 (m, 2H), 4.52 (s, 3H). ^13^C NMR (126 MHz, CDCl_3_) δ 195.9, 192.9, 190.8, 173.0, 137.7, 132.4, 130.7, 129.3 (q, *J* = 32.8 Hz), 127.9, 122.9, 122.7 (q, *J* = 274.2 Hz). HRMS: *m*/*z*: calcd. for C_12_H_7_ClF_3_O_3_^+^: 291.0030 [M + H]^+^; found: 291.0031.

#### 3-(3,4-Difluorophenyl)-4-methoxycyclobut-3-ene-1,2-dione **5n**

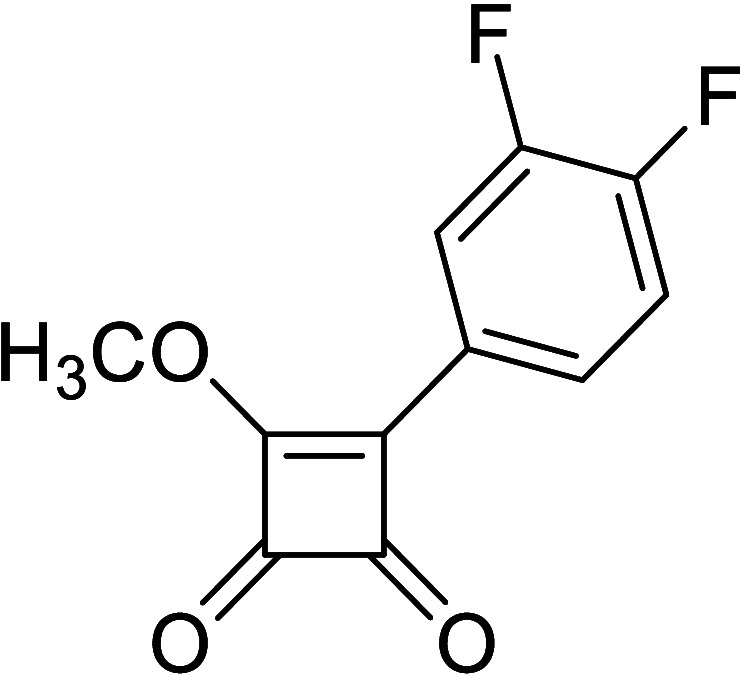



Product **5n** was obtained as a pale-yellow solid. Yield: 16 mg (12%). ^1^H NMR (400 MHz, CDCl_3_) δ 7.91–7.81 (m, 2H), 7.35–7.27 (m, 1H), 4.61 (s, 3H). ^13^C NMR (101 MHz, CDCl_3_) δ 194.9, 192.1, 171.4, 153.2 (dd, *J* = 230.6, 13.0 Hz), 150.7 (dd, *J* = 224.2, 13.0 Hz), 125.1 (dd, *J* = 6.9, 3.8 Hz), 124.6 (dd, *J* = 6.7, 4.3 Hz), 118.6 (d, *J* = 17.9 Hz), 116.8 (d, *J* = 18.1 Hz), 62.2. HRMS: *m*/*z*: calcd. For C_11_H_5_F_2_O_3_^–^: 223.0201 [M + H]^+^; found: 209.0042 (corresponds to a substance with hydroxyl instead of methoxy group).

#### 3-(2,3-Difluoro-4-methoxyphenyl)-4-methoxycyclobut-3-ene-1,2-dione **5o**

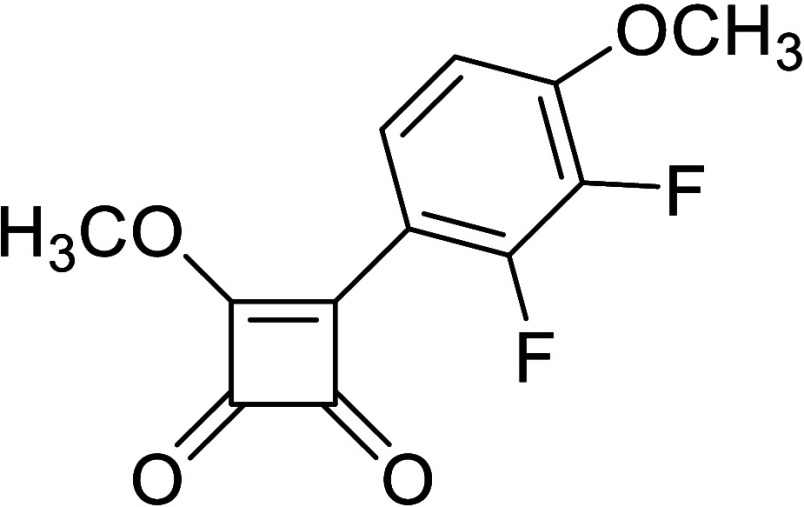



Product **5o** was obtained as a yellow solid. Yield: 7 mg (9%). ^1^H NMR (400 MHz, CDCl_3_) δ 8.00 (ddd, *J* = 9.2, 7.0, 2.4 Hz, 1H), 6.85 (ddd, *J* = 9.1, 7.3, 1.8 Hz, 1H), 4.58 (s, 3H), 3.99 (s, 3H). ^13^C NMR (101 MHz, CDCl_3_) δ 193.3, 192.3, 191.6, 168.4 (t, *J* = 2.5 Hz), 153.3 (dd, *J* = 7.7, 3.6 Hz), 148.6 (dd, *J* = 259.9, 11.2 Hz), 141.5 (dd, *J* = 250.0, 13.3 Hz), 123.8 (t, *J* = 4.0 Hz), 110.4 (d, *J* = 14.0 Hz), 108.6, 61.8, 57.0. HRMS: *m*/*z*: calcd. for C_12_H_9_F_2_O_4_^+^: 255.0463 [M + H]^+^; found: 255.0460.

#### 3-(2,3-Difluoro-4-propoxyphenyl)-4-methoxycyclobut-3-ene-1,2-dione **5p**

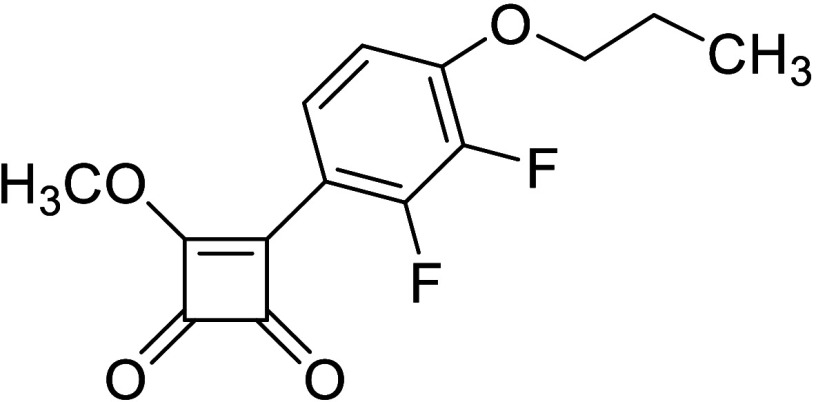



Product **5p** was obtained as a yellow solid. Yield: 22 mg (13%). ^1^H NMR (400 MHz, CDCl_3_) δ 8.97 (ddd, *J* = 9.2, 7.0, 2.4 Hz, 1H), 6.83 (ddd, *J* = 9.0, 7.2, 1.7 Hz, 1H), 4.58 (s, 3H), 4.09 (t, *J* = 6.5 Hz, 2H), 1.89 (h, *J* = 7.4 Hz, 2H), 1.07 (t, *J* = 7.4 Hz, 3H). ^13^C NMR (101 MHz, CDCl_3_) δ 193.2, 192.2, 191.7, 168.5 (t, *J* = 2.5 Hz), 152.9 (dd, *J* = 7.8, 3.7 Hz), 148.7 (dd, *J* = 259.7, 11.0 Hz), 141.6 (dd, *J* = 249.8, 13.2 Hz), 123.7 (t, *J* = 4.4 Hz), 110.0 (d, *J* = 14.1 Hz), 109.5, 71.6, 61.7, 22.5, 10.4. HRMS: *m*/*z*: calcd. for C_14_H_13_F_2_O_4_^+^: 283.0776 [M + H]^+^; found: 283.0774.

#### 3-(4-Butoxy-2,3-difluorophenyl)-4-methoxycyclobut-3-ene-1,2-dione **5q**

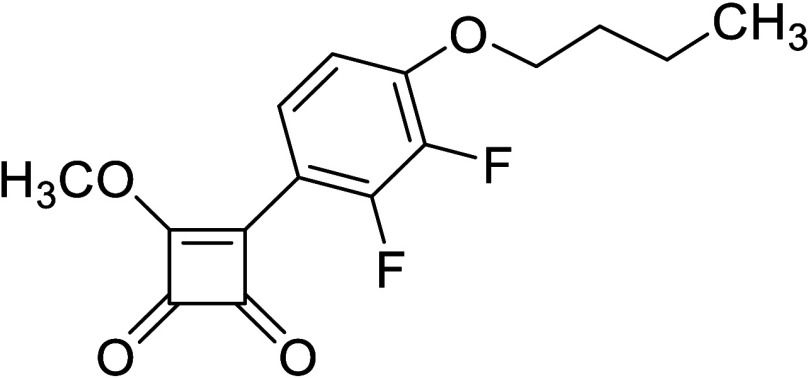



Product **5q** was obtained as a yellow solid. Yield: 16 mg (9%). ^1^H NMR (400 MHz, CDCl_3_) δ 7.97 (ddd, *J* = 9.2, 7.0, 2.4 Hz, 1H), 6.83 (ddd, *J* = 9.0, 7.2, 1.8 Hz, 1H), 4.58 (s, 3H), 4.13 (t, *J* = 6.5 Hz, 2H), 1.88–1.79 (m, 2H), 1.57–1.46 (m, 2H), 0.99 (t, *J* = 7.4 Hz, 3H). ^13^C NMR (101 MHz, CDCl_3_) δ 193.2, 192.3, 191.7, 168.6 (t, *J* = 3.0 Hz), 153.0 (dd, *J* = 8.1, 3.6 Hz), 148.7 (dd, *J* = 259.7, 11.1 Hz), 141.6 (dd, *J* = 249.8, 13.4 Hz), 123.7 (t, *J* = 3.5 Hz), 110.1 (d, *J* = 14.3 Hz), 109.5, 69.9, 61.7, 31.1, 19.2, 13.9. HRMS: *m*/*z*: calcd. for C_15_H_15_F_2_O_4_^+^: 297.0933 [M + H]^+^; found: 297.0934.

#### 3-(4-Ethoxy-3,5-dimethylphenyl)-4-methoxycyclobut-3-ene-1,2-dione **5r**

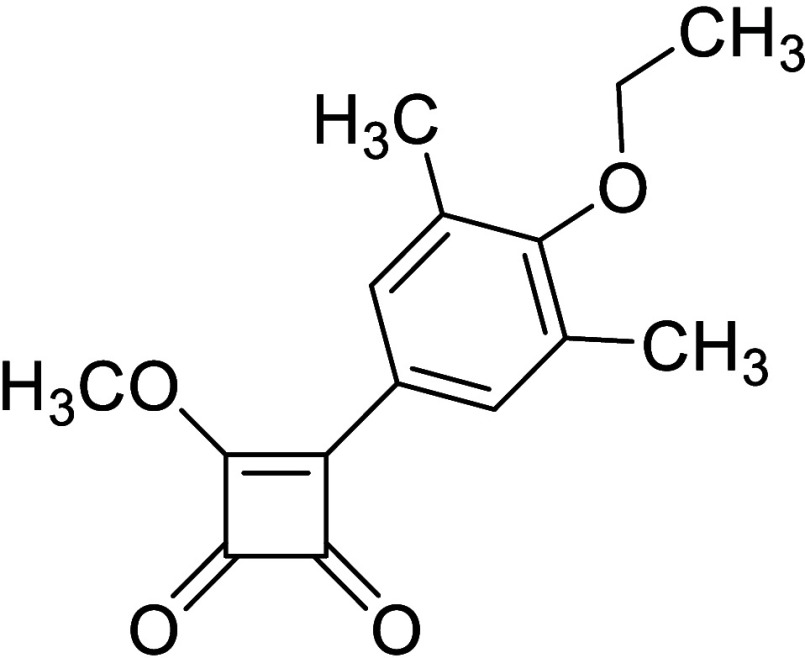



Product **5r** was obtained as a pale-yellow solid. Yield: 91 mg (58%). ^1^H NMR (400 MHz, CDCl_3_) δ 7.72 (s, 2H), 4.58 (s, 3H), 3.90 (q, *J* = 7.0 Hz, 2H), 2.32 (s, 6H), 1.44 (t, *J* = 7.0 Hz, 3H). ^13^C NMR (101 MHz, CDCl_3_) δ 194.1, 193.2, 192.2, 174.0, 160.5, 132.5, 128.9, 123.4, 68.4, 61.7, 16.5, 15.9. HRMS: *m*/*z*: calcd. for C_15_H_17_O_4_^+^: 261.1121 [M + H]^+^; found: 261.1123.

#### 3-Methoxy-4-(naphthalen-1-yl)cyclobut-3-ene-1,2-dione **5s**

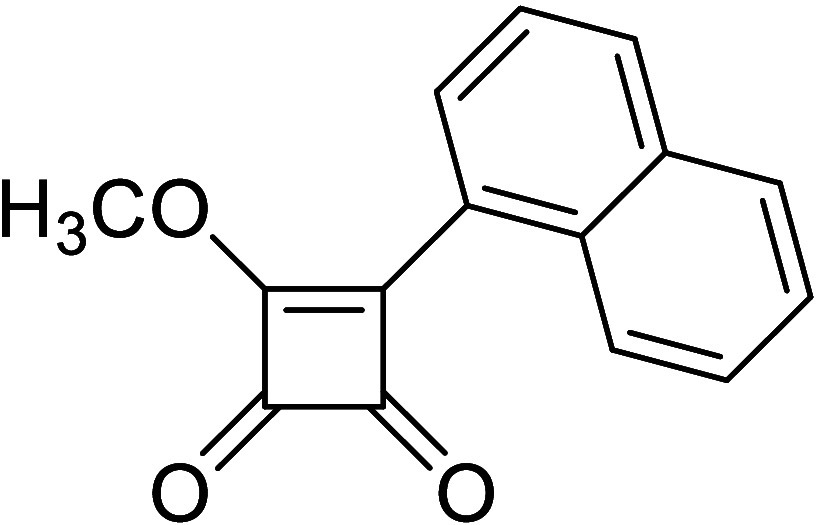



Product **5s** was obtained as a yellow solid. Yield: 87 mg (61%). ^1^H NMR (400 MHz, CDCl_3_) δ 8.39 (dd, *J* = 8.4, 0.9 Hz, 1H), 8.16 (dd, *J* = 7.3, 1.2 Hz, 1H), 8.02 (d, *J* = 8.2 Hz, 1H), 7.93–7.88 (m, 1H), 7.67–7.62 (m, 1H), 7.61–7.55 (m, 2H), 4.65 (s, 3H). ^13^C NMR (101 MHz, CDCl_3_) δ 194.8, 192.7, 192.5, 176.4, 134.0, 133.6, 130.4, 128.9, 128.5, 127.7, 126.9, 126.6, 125.6, 125.4, 62.0. HRMS: *m*/*z*: calcd. for C_15_H_11_O_3_^+^: 239.0703 [M + H]^+^; found: 239.0705.

#### 3-Methoxy-4-(4-methoxynaphthalen-1-yl)cyclobut-3-ene-1,2-dione **5t**

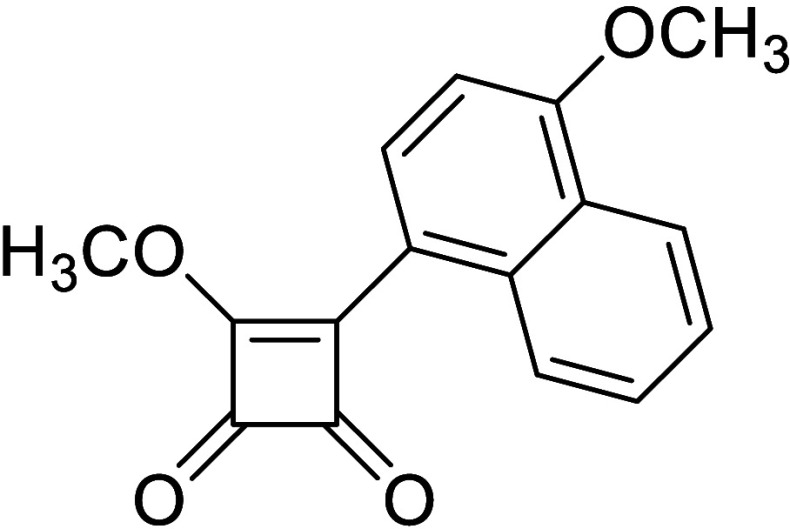



Product **5t** was obtained as a yellow solid. Yield: 78 mg (48%). ^1^H NMR (400 MHz, CDCl_3_) δ ^1^H NMR (400 MHz, Chloroform-*d*) δ 8.50 (d, *J* = 8.5 Hz, 1H), 8.33 (d, *J* = 8.3 Hz, 2H), 7.66 (ddd, *J* = 8.4, 6.9, 1.4 Hz, 1H), 7.56 (ddd, *J* = 8.1, 7.0, 1.0 Hz, 1H), 6.91 (d, *J* = 8.3 Hz, 1H), 4.65 (s, 3H), 4.09 (s, 3H). ^13^C NMR (101 MHz, CDCl_3_) δ 192.9, 191.9, 175.8, 160.1, 131.9, 130.8, 128.4, 126.5, 126.3, 125.9, 122.7, 118.9, 103.8, 61.8, 56.1. HRMS: *m*/*z*: calcd. for C_16_H_13_O_4_^+^: 269.0808 [M + H]^+^; found: 269.0809.

#### 3-(Benzo[*d*][1,3]dioxol-5-yl)-4-methoxycyclobut-3-ene-1,2-dione **5u**

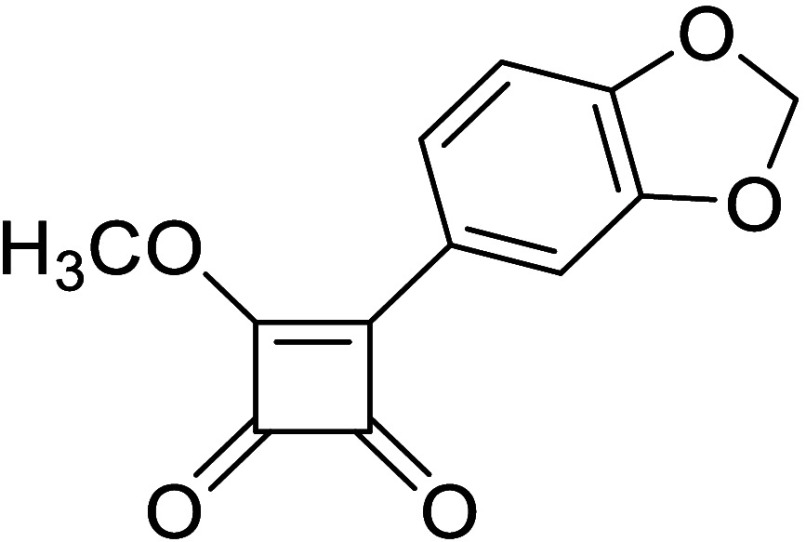



Product **5u** was obtained as a pale-yellow solid. Yield: 55 mg (40%). ^1^H NMR (400 MHz, CDCl_3_) δ 7.69 (dd, *J* = 8.1, 1.6 Hz, 1H), 7.47 (d, *J* = 1.6 Hz, 1H), 6.93 (d, *J* = 8.1 Hz, 1H), 6.07 (s, 2H), 4.58 (s, 3H). ^13^C NMR (126 MHz, CDCl_3_) δ 193.5, 192.8, 191.7, 173.4, 151.8, 148.5, 124.3, 121.9, 109.3, 107.4, 102.1, 61.7. HRMS: *m*/*z*: calcd. for C_12_H_9_O_5_^+^: 233.0444 [M + H]^+^; found: 233.0447.

#### 3-(2,3-Dihydrobenzo[*b*][1,4]dioxin-6-yl)-4-methoxycyclobut-3-ene-1,2-dione **5v**

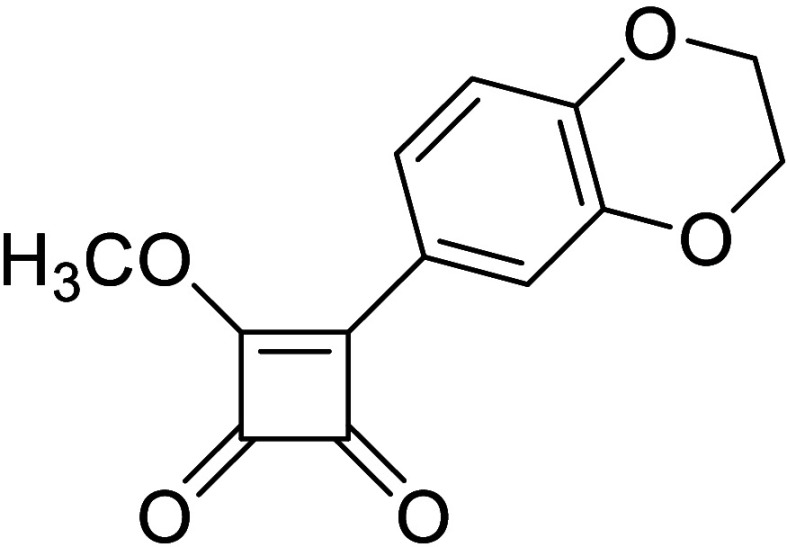



Product **5v** was obtained as a pale-yellow solid. Yield: 68 mg (46%). ^1^H NMR (400 MHz, CDCl_3_) δ 7.60–7.55 (m, 2H), 6.95 (d, *J* = 8.2 Hz, 1H), 4.56 (s, 3H), 4.36–4.25 (m, 4H). ^13^C NMR (126 MHz, CDCl_3_) δ 193.6, 192.8, 191.9, 173.6, 148.0, 144.0, 122.4, 121.2, 118.2, 116.9, 64.9, 64.1, 61.6. HRMS: *m*/*z*: calcd. for C_13_H_11_O_5_^+^: 247.0601 [M + H]^+^; found: 247.0605.

#### 3-(6-Ethoxynaphthalen-2-yl)-4-methoxycyclobut-3-ene-1,2-dione **5w**

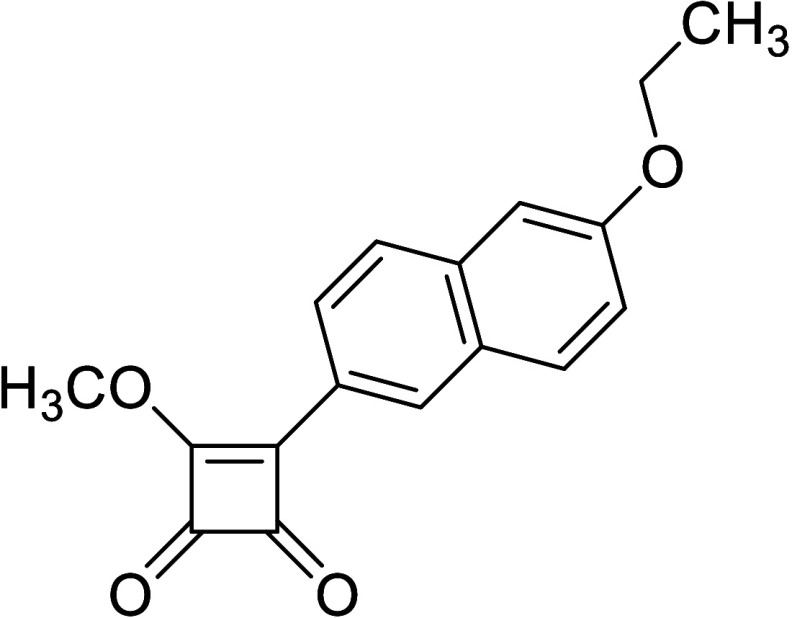



Product **5w** was obtained as a pale-yellow solid. Yield: 97 mg (57%). ^1^H NMR (400 MHz, CDCl_3_) δ 8.56 (d, *J* = 1.1 Hz, 1H), 7.94 (dd, *J* = 8.5, 1.7 Hz, 1H), 7.85 (d, *J* = 9.0 Hz, 1H), 7.77 (d, *J* = 8.6 Hz, 1H), 7.21 (dd, *J* = 9.0, 2.5 Hz, 1H), 7.13 (d, *J* = 2.4 Hz, 1H), 4.63 (s, 3H), 4.18 (q, *J* = 7.0 Hz, 2H), 1.50 (t, *J* = 6.9 Hz, 3H). ^13^C NMR (126 MHz, CDCl_3_) δ 194.2, 193.2, 192.1, 174.2, 159.6, 137.1, 131.3, 129.1, 128.3, 127.7, 124.3, 123.0, 120.4, 106.9, 63.9, 61.8, 14.9. HRMS: *m*/*z*: calcd. for C_17_H_15_O_4_^+^: 283.0965 [M + H]^+^; found: 283.0963.

#### 3-(Furan-3-yl)-4-methoxycyclobut-3-ene-1,2-dione **5x**

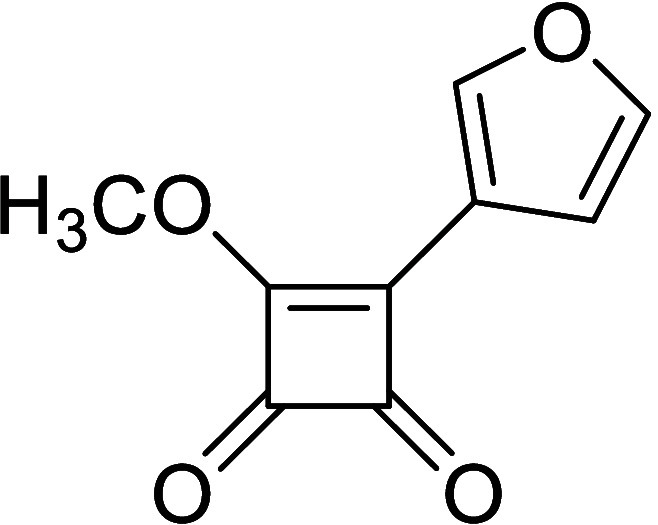



Product **5x** was obtained as a white solid. Yield: 94 mg (88%). ^1^H NMR (400 MHz, CDCl_3_) δ 8.24 (dd, *J* = 1.3, 0.8 Hz, 1H), 7.58 (t, *J* = 1.6 Hz, 1H), 6.82 (dd, *J* = 1.9, 0.6 Hz, 1H), 4.55 (s, 3H). ^13^C NMR (101 MHz, CDCl_3_) δ 199.5, 193.4, 191.8, 191.3, 168.7, 145.0, 113.6, 107.7, 61.6. HRMS: *m*/*z*: calcd. for C_9_H_7_O_4_^+^: 179.0339 [M + H]^+^; found: 179.0341.

#### 3-Methoxy-4-(thiophen-3-yl)cyclobut-3-ene-1,2-dione **5y**

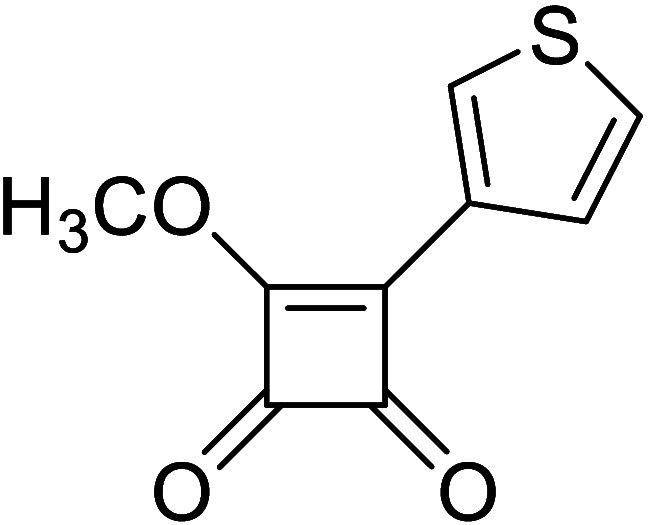



Product **5y** was obtained as a yellow solid. Yield: 69 mg (59%). ^1^H NMR (400 MHz, CDCl_3_) δ 8.28 (dd, *J* = 2.9, 1.1 Hz, 1H), 7.60 (dd, *J* = 5.0, 1.2 Hz, 1H), 7.47 (dd, *J* = 5.1, 2.9 Hz, 1H), 4.58 (s, 3H). ^13^C NMR (126 MHz, CDCl_3_) δ 193.0, 192.1, 169.6, 130.5, 128.2, 127.5, 125.8, 61.7. HRMS: *m*/*z*: calcd. for C_9_H_7_O_3_S^+^: 195.0110 [M + H]^+^; found: 195.0114.

#### 3-Methoxy-4-(5-methylthiophen-2-yl)cyclobut-3-ene-1,2-dione **5z**

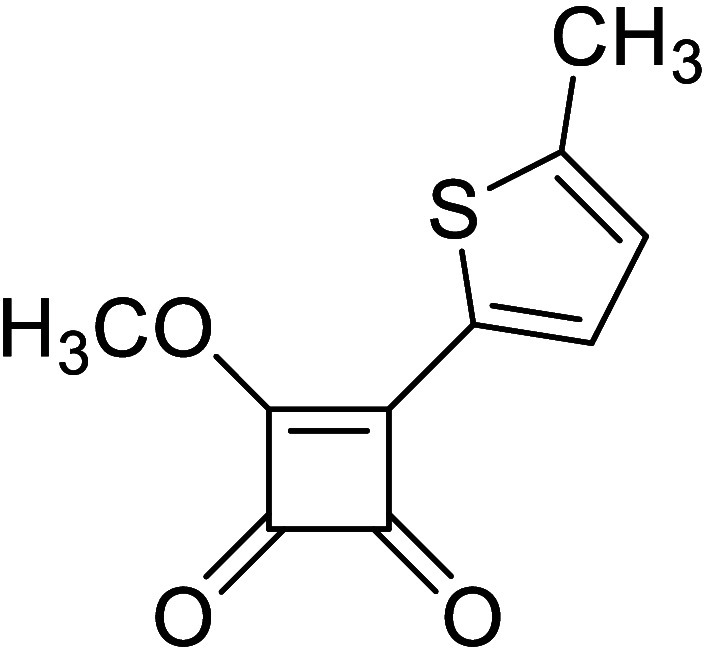



Product **5z** was obtained as a pale-yellow solid. Yield: 52 mg (42%). ^1^H NMR (400 MHz, CDCl_3_) δ 7.72 (d, *J* = 3.7 Hz, 1H), 6.93 (dd, *J* = 3.9, 1.2 Hz, 1H), 4.57–4.55 (m, 3H), 2.60 (s, 3H). ^13^C NMR (101 MHz, CDCl_3_) δ 190.9, 190.6, 190.0, 168.4, 150.8, 132.6, 128.0, 125.6, 61.6, 16.1. HRMS: *m*/*z*: calcd. for C_10_H_9_O_3_S^+^: 209.0267 [M + H]^+^; found: 209.0266.

#### Benzyl 4-(2-methoxy-3,4-dioxocyclobut-1-en-1-yl)benzoate **5aa**

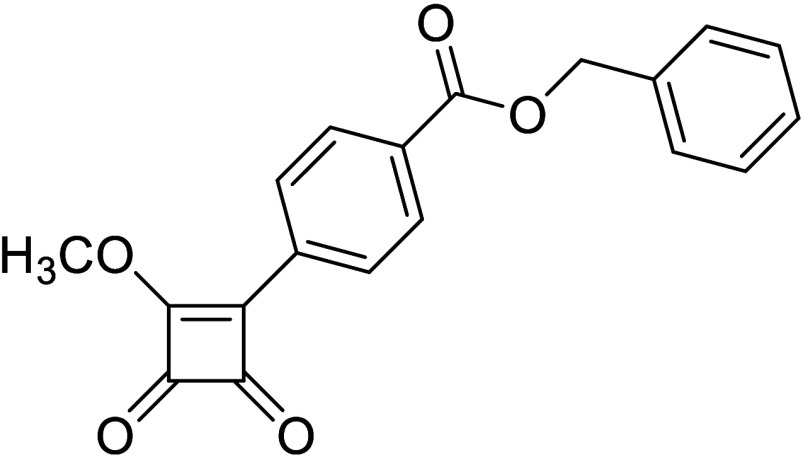



Product **5aa** was obtained as a yellow solid. Yield: 2 mg (1%). ^1^H NMR (400 MHz, CDCl_3_) δ 8.21–8.16 (m, 2H), 8.11–8.05 (m, 2H), 7.48–7.33 (m, 5H), 5.39 (s, 2H), 4.63 (s, 3H). ^13^C NMR was not measured due to the small amount of the compound. HRMS: *m*/*z*: calcd. for C_19_H_15_O_5_^+^: 323.0914 [M + H]^+^; found: 323.0914.

#### 3-(1*H*-Indol-5-yl)-4-methoxycyclobut-3-ene-1,2-dione **5ab**

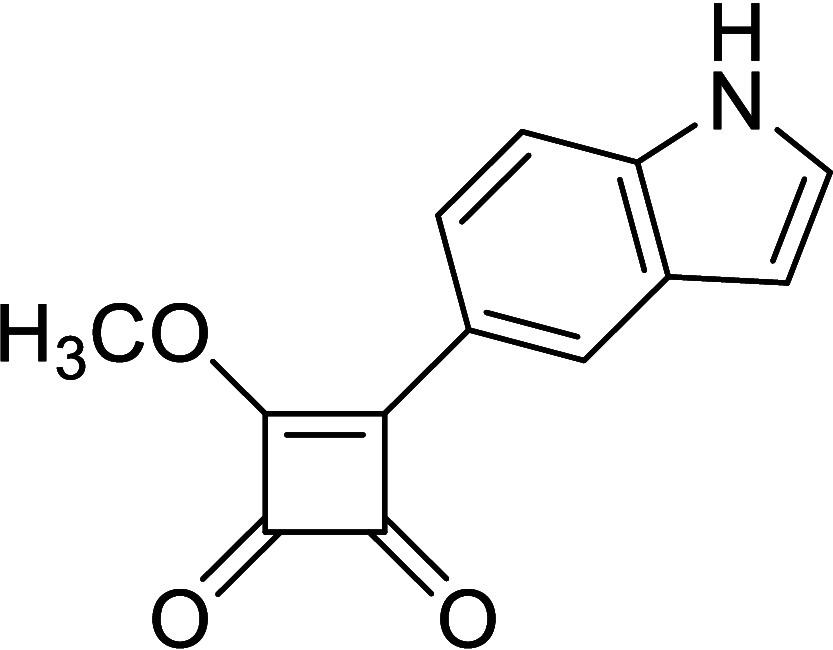



Product **5ab** was obtained as a white solid. Yield: 3 mg (2%). ^1^H NMR (400 MHz, DMSO-*d*_6_) δ 11.60 (br s, 1H), 8.28 (s, 1H), 7.71 (dd, *J* = 8.5, 1.6 Hz, 1H), 7.59 (d, *J* = 8.5 Hz, 1H), 7.50 (t, *J* = 2.8 Hz, 1H), 6.65 (t, *J* = 2.1 Hz, 1H), 4.54 (s, 3H). ^13^C NMR was not measured due to the small amount of the compound. HRMS: *m*/*z*: calcd. for C_13_H_10_NO_3_^+^: 228.0655 [M + H]^+^; found: 228.0656.

### General Procedure for the Synthesis of **6**

Precursor **5** (25 mg) was dissolved in MeCN. Then, 2-(aminomethyl)pyridine was added dropwisely (1 equiv). The reaction mixture was stirred at rt for 60 min. Product **6** was isolated by filtration when the precipitate was formed, and the solid was washed with MeCN. Otherwise, after the completion of the reaction, the solvent was evaporated under reduced pressure, and product **6** was purified by column chromatography using DCM/MeOH (grad.).

#### 3-((Pyridin-2-ylmethyl)amino)-4-(*p*-tolyl)cyclobut-3-ene-1,2-dione **6a**

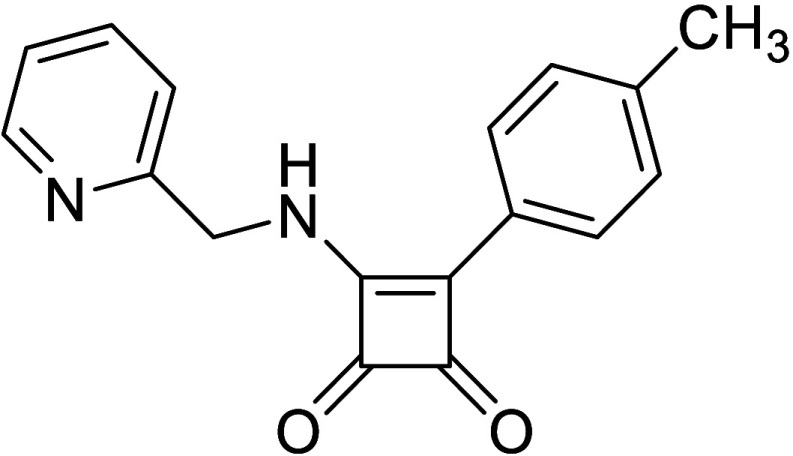



Product **6a** was obtained as a white solid. Yield: 31 mg (90%). ^1^H NMR (400 MHz, DMSO-*d*_6_) δ 9.56 (t, *J* = 6.0 Hz, 1H), 8.55 (ddd, *J* = 4.8, 1.6, 0.8 Hz, 1H), 7.95 (d, *J* = 8.2 Hz, 2H), 7.81 (td, *J* = 7.6, 1.8 Hz, 1H), 7.45 (d, *J* = 7.8 Hz, 1H), 7.37 (d, *J* = 8.0 Hz, 2H), 7.32 (ddd, *J* = 7.5, 4.8, 1.2 Hz, 1H), 5.02 (d, *J* = 6.1 Hz, 2H), 2.38 (s, 3H). ^13^C NMR (101 MHz, DMSO-*d*_6_) δ 193.1, 188.9, 179.0, 162.0, 157.1, 149.3, 140.1, 137.0, 129.6, 126.6, 126.2, 122.7, 121.6, 48.9, 21.3. HRMS: *m*/*z*: calcd. for C_17_H_15_N_2_O_2_^+^: 279.1128 [M + H]^+^; found: 279.1125.

#### 3-(4-Bromophenyl)-4-((pyridin-2-ylmethyl)amino)cyclobut-3-ene-1,2-dione **6b**

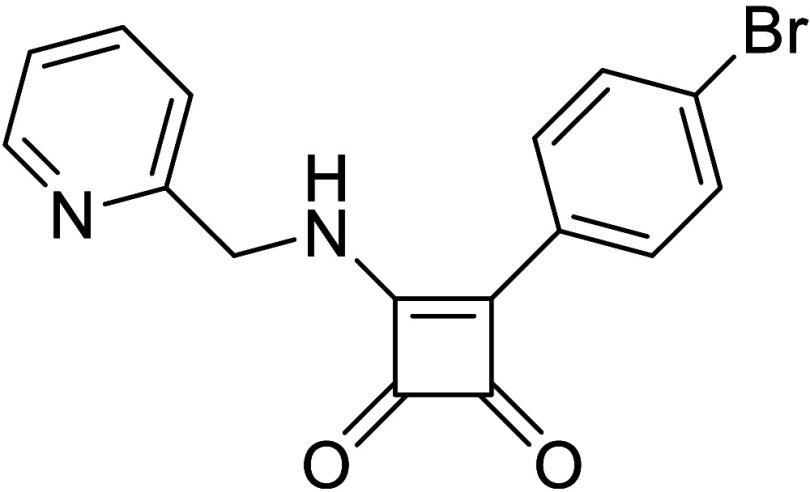



Product **6b** was obtained as a white solid. Yield: 28 mg (87%). ^1^H NMR (400 MHz, DMSO-*d*_6_) δ 9.70 (br s, 1H), 8.54 (d, *J* = 4.6 Hz, 1H), 7.99 (d, *J* = 8.5 Hz, 2H), 7.80 (td, *J* = 7.7, 1.9 Hz, 1H), 7.73 (d, *J* = 8.5 Hz, 2H), 7.46 (d, *J* = 7.8 Hz, 1H), 7.31 (dd, *J* = 7.6, 4.9 Hz, 1H), 5.01 (s, 2H). ^13^C NMR (101 MHz, DMSO-*d*_6_) δ 195.6, 188.4, 178.6, 159.7, 157.8, 149.1, 136.9, 131.9, 128.9, 127.6, 123.0, 122.5, 121.7, 50.1. HRMS: *m*/*z*: calcd. for C_16_H_12_BrN_2_O_2_^+^: 343.0077 [M + H]^+^; found: 343.0075.

#### 3-(4-Hydroxyphenyl)-4-((pyridin-2-ylmethyl)amino)cyclobut-3-ene-1,2-dione **6c**

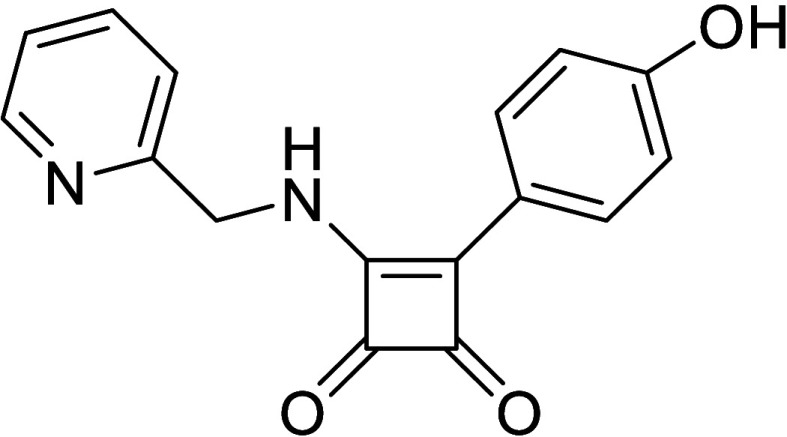



Product **6c** was obtained as a white solid. Yield: 30 mg (87%). ^1^H NMR (400 MHz, DMSO-*d*_6_) δ 10.24 (br s, 1H), 9.39 (br s, 1H), 8.55 (dd, *J* = 4.8, 0.8 Hz, 1H), 7.97–7.90 (m, 1H), 7.81 (td, *J* = 7.7, 1.8 Hz, 1H), 7.43 (d, *J* = 7.8 Hz, 0H), 7.32 (dd, *J* = 7.4, 4.8 Hz, 0H), 6.95–6.89 (m, 1H), 5.00 (d, *J* = 5.2 Hz, 1H). ^13^C NMR (101 MHz, DMSO-*d*_6_) δ 192.2, 188.9, 178.3, 162.8, 160.0, 157.3, 149.2, 137.0, 128.6, 122.7, 121.6, 120.7, 115.9, 48.8. HRMS: *m*/*z*: calcd. for C_16_H_13_N_2_O_3_^+^: 281.0921 [M + H]^+^; found: 281.0920.

#### 3-((Pyridin-2-ylmethyl)amino)-4-(4-(trifluoromethoxy)phenyl)cyclobut-3-ene-1,2-dione **6d**

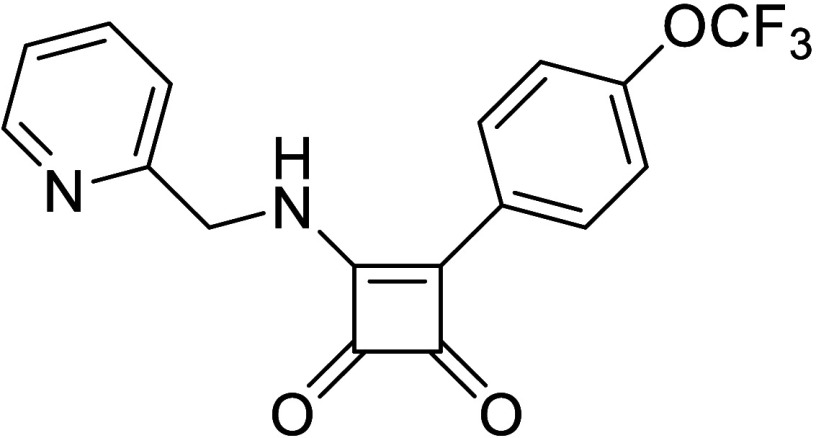



Product **6d** was obtained as a pale-yellow solid. Yield: 29 mg (91%). ^1^H NMR (400 MHz, DMSO-*d*_6_) δ 9.77 (br s, 1H), 8.60–8.51 (m, 1H), 8.15 (d, *J* = 8.8 Hz, 2H), 7.82 (td, *J* = 7.7, 1.7 Hz, 1H), 7.56 (d, *J* = 8.2 Hz, 2H), 7.47 (d, *J* = 7.8 Hz, 1H), 7.33 (dd, *J* = 7.1, 1.9 Hz, 1H), 5.03 (s, 2H). ^13^C NMR (126 MHz, DMSO-*d*_6_) δ 193.4, 188.6, 179.2, 159.7, 156.9, 149.3, 149.1, 137.1, 128.3, 128.2, 122.8, 121.7, 121.5, 120.0 (q, *J* = 257.3 Hz), 48.9. HRMS: *m*/*z*: calcd. for C_17_H_12_F_3_N_2_O_2_^+^: 349.0795 [M + H]^+^; found: 349.0792.

#### 3-(4-(Methylthio)phenyl)-4-((pyridin-2-ylmethyl)amino)cyclobut-3-ene-1,2-dione **6e**

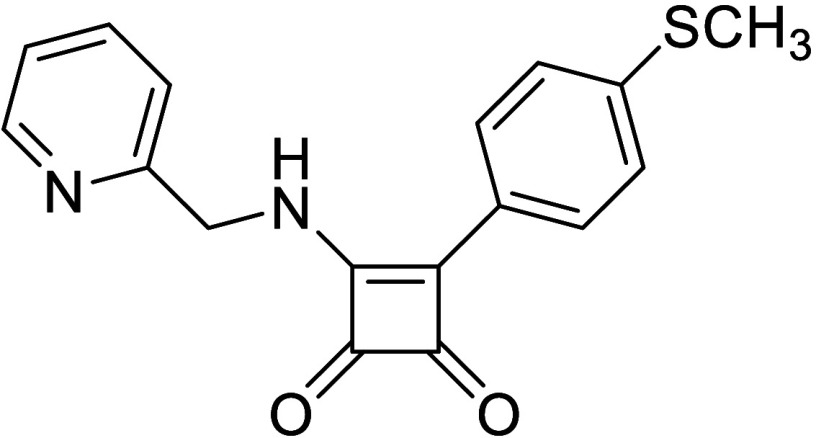



Product **6e** was obtained as a pale-yellow solid. Yield: 30 mg (91%). ^1^H NMR (400 MHz, DMSO-*d*_6_) δ 9.59 (br s, 1H), 8.56 (d, *J* = 4.4 Hz, 1H), 7.98 (d, *J* = 8.5 Hz, 2H), 7.81 (td, *J* = 7.6, 1.8 Hz, 1H), 7.45 (d, *J* = 7.8 Hz, 1H), 7.40 (d, *J* = 8.5 Hz, 2H), 7.32 (dd, *J* = 7.5, 2.0 Hz, 1H), 5.02 (s, 2H), 2.54 (s, 3H). ^13^C NMR (126 MHz, DMSO-*d*_6_) δ 192.8, 188.8, 178.8, 161.4, 157.1, 149.2, 142.2, 137.0, 126.5, 125.5, 125.4, 122.7, 121.6, 48.9, 14.1. HRMS: *m*/*z*: calcd. for C_17_H_15_N_2_O_2_S^+^: 311.0849 [M + H]^+^; found: 311.0846.

#### 3-(4-(*tert*-Butoxy)phenyl)-4-((pyridin-2-ylmethyl)amino)cyclobut-3-ene-1,2-dione **6f**

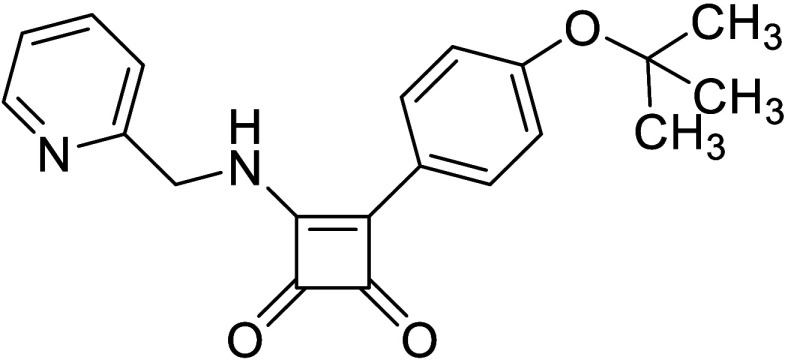



Product **6f** was obtained as a white solid. Yield: 28 mg (86%). ^1^H NMR (400 MHz, DMSO-*d*_6_) δ 9.54 (br s, 1H), 8.55 (ddd, *J* = 4.9, 1.1 Hz, 2H), 8.05–7.94 (m, 2H), 7.81 (td, *J* = 7.6, 1.8 Hz, 1H), 7.45 (d, *J* = 7.8 Hz, 1H), 7.32 (ddd, *J* = 7.6, 4.9, 1.1 Hz, 1H), 7.17–7.11 (m, 2H), 5.01 (s, 2H), 1.37 (s, 9H). ^13^C NMR (101 MHz, DMSO-*d*_6_) δ 192.8, 188.8, 178.8, 161.9, 157.6, 157.2, 149.2, 137.0, 127.6, 123.8, 123.0, 122.7, 121.6, 79.2, 48.9, 28.5. HRMS: *m*/*z*: calcd. for C_20_H_21_N_2_O_3_^+^: 337.1547 [M + H]^+^; found: 337.1538.

#### 3-(4-(2-Methoxyethoxy)phenyl)-4-((pyridin-2-ylmethyl)amino)cyclobut-3-ene-1,2-dione **6g**

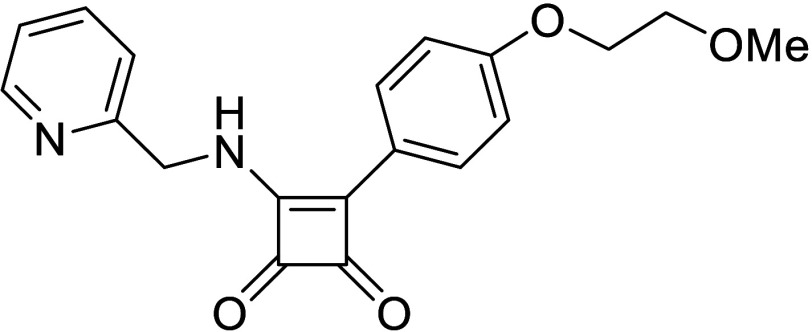



Product **6g** was obtained as a white solid. Yield: 30 mg (94%). ^1^H NMR (400 MHz, DMSO-*d*_6_) δ 9.50 (br s, 1H), 8.55 (ddd, *J* = 4.8, 1.6, 0.8 Hz, 1H), 8.06–7.97 (m, 2H), 7.81 (td, *J* = 7.7, 1.8 Hz, 1H), 7.45 (d, *J* = 7.9 Hz, 1H), 7.32 (ddd, *J* = 7.4, 4.9, 0.9 Hz, 1H), 7.15–7.10 (m, 2H), 5.01 (s, 2H), 4.22–4.16 (m, 2H), 3.71–3.65 (m, 2H), 3.31 (s, 3H). ^13^C NMR (101 MHz, DMSO-*d*_6_) δ ^13^C NMR (101 MHz, DMSO-*d*_6_) δ 192.5, 188.8, 178.5, 162.1, 160.4, 157.2, 149.2, 137.0, 128.3, 122.7, 122.1, 121.6, 115.0, 70.2, 67.2, 58.2, 48.8. HRMS: *m*/*z*: calcd. for C_19_H_19_N_2_O_4_^+^: 339.1339 [M + H]^+^; found: 339.1342.

#### 3-(4-Phenoxyphenyl)-4-((pyridin-2-ylmethyl)amino)cyclobut-3-ene-1,2-dione **6h**

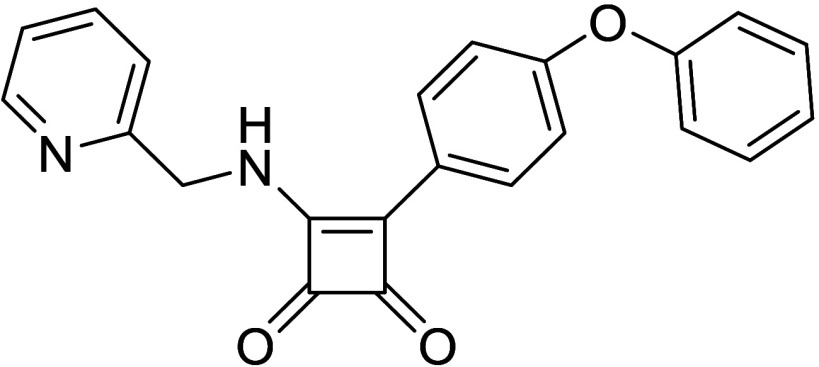



Product **6h** was obtained as a white solid. Yield: 30 mg (96%). ^1^H NMR (400 MHz, DMSO-*d*_6_) δ 9.59 (t, *J* = 6.3 Hz, 1H), 8.56 (d, *J* = 4.3 Hz, 1H), 8.10–8.05 (m, 2H), 7.81 (td, *J* = 7.7, 1.8 Hz, 1H), 7.47–7.41 (m, 3H), 7.32 (ddd, *J* = 7.6, 4.9, 1.1 Hz, 1H), 7.24–7.19 (m, 1H), 7.17–7.13 (m, 2H), 7.12–7.07 (m, 2H), 5.02 (d, *J* = 6.2 Hz, 2H). ^13^C NMR (101 MHz, DMSO-*d*_6_) δ 192.9, 188.7, 178.8, 161.3, 158.6, 157.1, 155.6, 149.3, 137.0, 130.2, 128.4, 124.4, 124.3, 122.7, 121.6, 119.3, 118.5, 48.9. HRMS: *m*/*z*: calcd. for C_22_H_17_N_2_O_3_^+^: 357.1234 [M + H]^+^; found: 357.1234.

#### 3-(4-(Benzyloxy)phenyl)-4-((pyridin-2-ylmethyl)amino)cyclobut-3-ene-1,2-dione **6i**

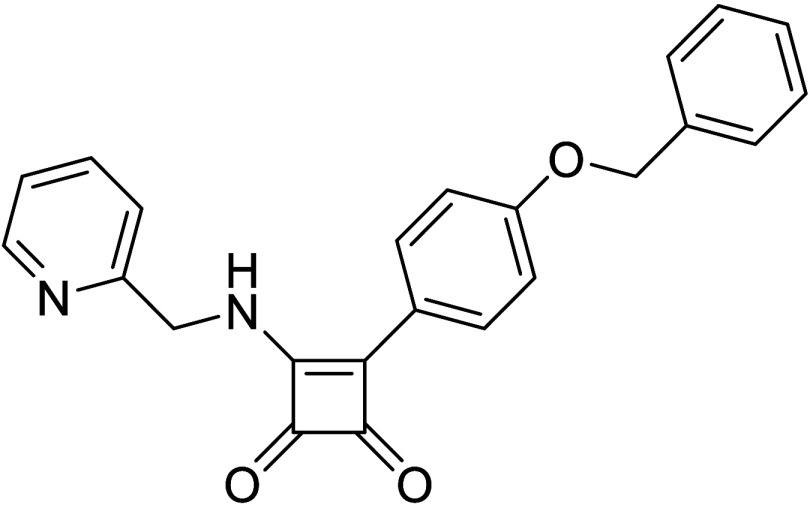



Product **6i** was obtained as a white solid. Yield: 27 mg (87%). ^1^H NMR (400 MHz, DMSO-*d*_6_) δ 9.50 (br s, 1H), 8.55 (ddd, *J* = 4.7, 1.8, 0.9 Hz, 1H), 8.07–8.00 (m, 2H), 7.81 (td, *J* = 7.7, 1.8 Hz, 1H), 7.49–7.38 (m, 5H), 7.37–7.30 (m, 2H), 7.23–7.17 (m, 2H), 5.21 (s, 2H), 5.01 (d, *J* = 5.0 Hz, 2H). ^13^C NMR (101 MHz, DMSO-*d*_6_) δ 192.5, 188.8, 178.6, 162.0, 160.2, 157.2, 149.2, 137.0, 136.5, 128.5, 128.2, 128.0, 127.8, 122.7, 122.3, 121.6, 115.4, 69.4, 48.8. HRMS: *m*/*z*: calcd. for C_23_H_19_N_2_O_3_^+^: 371.1390 [M + H]^+^; found: 371.1366.

#### 3-(4-((4-Fluorobenzyl)oxy)phenyl)-4-((pyridin-2-ylmethyl)amino)cyclobut-3-ene-1,2-dione **6j**

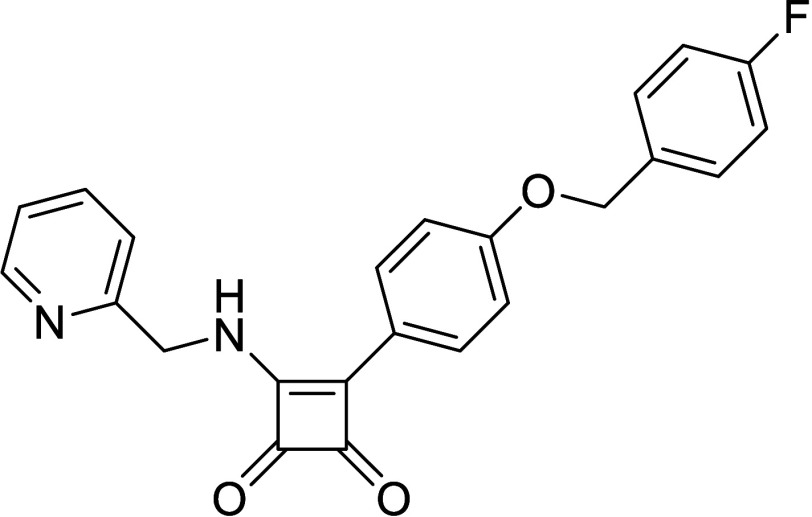



Product **6j** was obtained as a pale-yellow solid. Yield: 28 mg (90%). ^1^H NMR (400 MHz, DMSO-*d*_6_) δ 9.51 (t, *J* = 5.3 Hz, 1H), 8.55 (d, *J* = 4.1 Hz, 1H), 8.03 (d, *J* = 8.7 Hz, 2H), 7.85–7.77 (m, 1H), 7.53 (dd, *J* = 8.0, 2.7 Hz, 2H), 7.44 (d, *J* = 7.7 Hz, 1H), 7.35–7.30 (m, 1H), 7.29–7.14 (m, 4H), 5.19 (s, 2H), 5.01 (d, *J* = 5.9 Hz, 2H). ^13^C NMR (126 MHz, DMSO-*d*_6_) δ 192.5, 188.8, 178.6, 162.0, 161.8 (d, *J* = 243.9 Hz), 160.1, 157.2, 149.2, 137.0, 132.8 (d, *J* = 2.8 Hz), 130.1 (d, *J* = 8.3 Hz), 128.2, 122.7, 122.3, 121.6, 115.4, 115.3 (d, *J* = 21.5 Hz), 68.7, 48.8. HRMS: *m*/*z*: calcd. for C_23_H_18_FN_2_O_3_^+^: 389.1296 [M + H]^+^; found: 389.1294.

#### 3-(4-(Benzyloxy)-3-chlorophenyl)-4-((pyridin-2-ylmethyl)amino)cyclobut-3-ene-1,2-dione **6k**

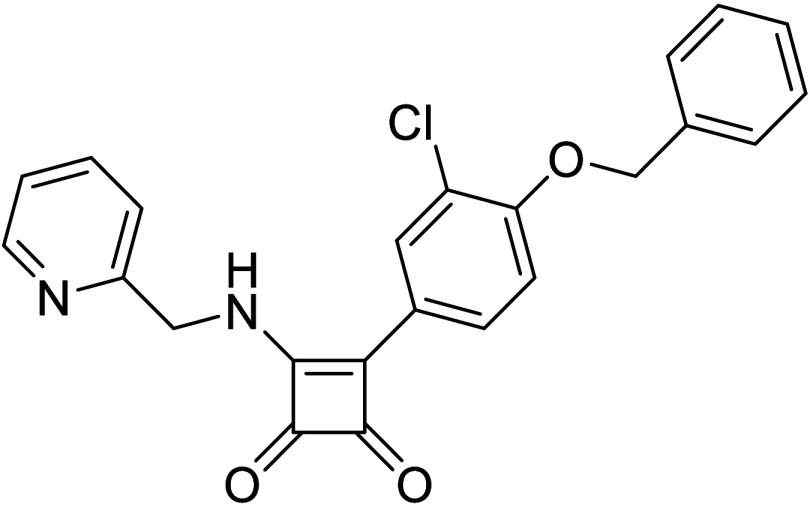



Product **6k** was obtained as a pale-yellow solid. Yield: 28 mg (92%). ^1^H NMR (400 MHz, DMSO-*d*_6_) δ 9.68 (br s, 1H), 8.56 (ddd, *J* = 4.9, 1.5, 0.8 Hz, 1H), 8.14 (d, *J* = 2.1 Hz, 1H), 8.03 (dd, *J* = 8.7, 2.1 Hz, 1H), 7.82 (td, *J* = 7.7, 1.8 Hz, 1H), 7.54–7.28 (m, 8H), 5.32 (s, 2H), 5.01 (s, 2H). ^13^C NMR (126 MHz, DMSO-*d*_6_) δ 193.8, 192.6, 188.6, 178.5, 160.1, 157.0, 155.0, 149.3, 137.1, 136.1, 128.5, 128.1, 127.6, 127.4, 126.7, 123.0, 122.7, 122.4, 121.7, 70.3, 48.9. HRMS: *m*/*z*: calcd. for C_23_H_18_ClN_2_O_3_^+^: 405.1000 [M + H]^+^; found: 405.1002.

#### 3-(3-Chloro-4-methoxyphenyl)-4-((pyridin-2-ylmethyl)amino)cyclobut-3-ene-1,2-dione **6l**

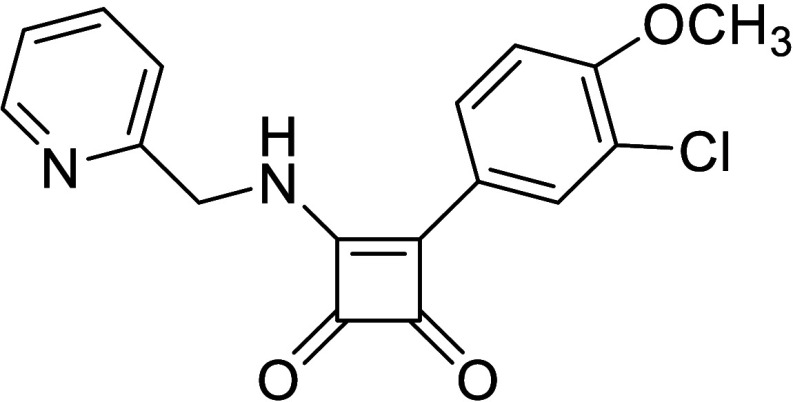



Product **6l** was obtained as a pale-yellow solid. Yield: 31 mg (96%). ^1^H NMR (400 MHz, DMSO-*d*_6_) δ 9.68 (t, *J* = 6.1 Hz, 1H), 8.56 (ddd, *J* = 4.8, 1.6, 0.9 Hz, 1H), 8.12 (d, *J* = 2.1 Hz, 1H), 8.07 (dd, *J* = 8.6, 2.1 Hz, 1H), 7.82 (td, *J* = 7.7, 1.8 Hz, 1H), 7.46 (d, *J* = 7.9 Hz, 1H), 7.35–7.30 (m, 2H), 5.02 (d, *J* = 6.1 Hz, 2H), 3.95 (s, 3H). ^13^C NMR (126 MHz, DMSO-*d*_6_) δ 193.2, 189.2, 179.0, 160.8, 157.6, 156.6, 149.8, 137.6, 127.9, 127.5, 123.3, 122.5, 122.3, 113.8, 57.0, 49.4. HRMS: *m*/*z*: calcd. for C_17_H_14_ClN_2_O_3_^+^: 329.0687 [M + H]^+^; found: 329.0686.

#### 3-(4-Chloro-2-(trifluoromethyl)phenyl)-4-((pyridin-2-ylmethyl)amino)cyclobut-3-ene-1,2-dione **6m**

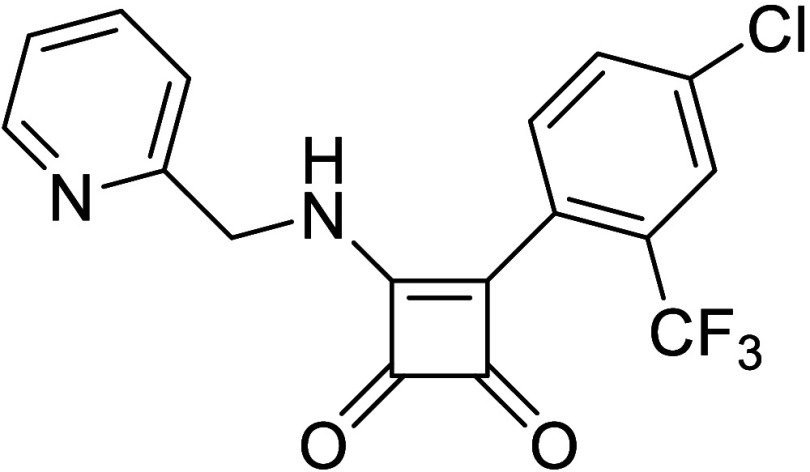



Product **6m** was obtained as a brown oil. Yield: 29 mg (91%). ^1^H NMR (400 MHz, DMSO-*d*_6_), 53: 47 mixture of two rotamers. Major rotamer: δ 9.59 (br s, 1H), 8.55 (ddd, *J* = 4.5, 1.5, 0.8 Hz, 1H), 7.98 (d, *J* = 2.0 Hz, 1H), 7.91 (dd, *J* = 8.3, 2.1 Hz, 1H), 7.83–7.79 (m, 1H), 7.54 (d, *J* = 8.3 Hz, 1H), 7.39 (d, J = 7.9 Hz, 1H) 7.34–7.30 (m, 1H), 4.89 (s, 2H). Minor rotamer, characteristic signals: δ 10.03 (br s, 1H), 8.36 (ddd, *J* = 4.5, 1.4, 0.8 Hz, 1H), 7.72 (dd, *J* = 8.3, 2.2 Hz, 1H), 7.66 (td, *J* = 7.7, 1.8 Hz, 1H), 7.06 (d, *J* = 7.9 Hz, 1H), 4.30 (s, 2H). ^13^C NMR (126 MHz, DMSO-*d*_6_), 53:47 mixture of two rotamers. Signals are not specified for major and minor rotamer. δ 194.0, 193.4, 187.4, 187.1, 181.5, 181.1, 161.9, 161.6, 156.6, 155.5, 149.3, 149.0, 137.1, 136.8, 134.3, 134.2, 132.7, 132.3, 131.5, 131.4, 128.2 (q, *J* = 31.4 Hz), 128.1 (q, *J* = 30.9 Hz), 126.5, 126.2, 125.7, 125.7, 122.8 (q, *J* = 273.9 Hz), 122.8, 122.7 (q, *J* = 275.1 Hz), 122.6, 121.5, 121.3, 49.0, 48.8. HRMS: *m*/*z*: calcd. for C_17_H_11_ClF_3_N_2_O_2_^+^: 367.0456 [M + H]^+^; found: 367.0463.

#### 3-(3,4-Difluorophenyl)-4-((pyridin-2-ylmethyl)amino)cyclobut-3-ene-1,2-dione **6n**

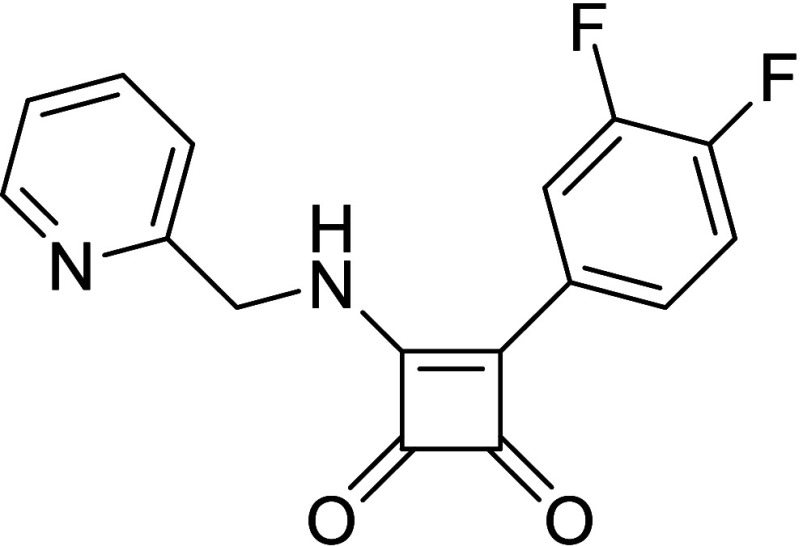



Product **6n** was obtained as a white solid. Yield: 30 mg (89%). ^1^H NMR (400 MHz, DMSO-*d*_6_) δ 9.79 (br s, 1H), 8.61–8.47 (m, 1H), 8.08–8.00 (m, 1H), 7.97–7.90 (m, 1H), 7.82 (td, *J* = 7.7, 1.8 Hz, 1H), 7.64 (dt, *J* = 10.7, 8.6 Hz, 1H), 7.47 (d, *J* = 7.9 Hz, 1H), 7.33 (ddd, *J* = 7.5, 4.8, 1.1 Hz, 1H), 5.02 (s, 2H). ^13^C NMR (101 MHz, DMSO-*d*_6_) δ 193.2, 188.5, 178.9, 158.8, 156.8, 150.2 (dd, *J* = 251.6, 12.6 Hz), 149.7 (dd, *J* = 246.9, 13.1 Hz), 149.3, 137.1, 126.6 (dd, *J* = 6.8, 3.8 Hz), 123.8 (dd, *J* = 6.5, 3.3 Hz), 122.8, 121.79, 118.5 (d, *J* = 17.6 Hz), 114.8 (d, *J* = 18.1 Hz), 49.0. HRMS: *m*/*z*: calcd. for C_16_H_11_F_2_N_2_O_2_^+^: 301.0783 [M + H]^+^; found: 301.0782.

#### 3-(2,3-Difluoro-4-methoxyphenyl)-4-((pyridin-2-ylmethyl)amino)cyclobut-3-ene-1,2-dione **6o**

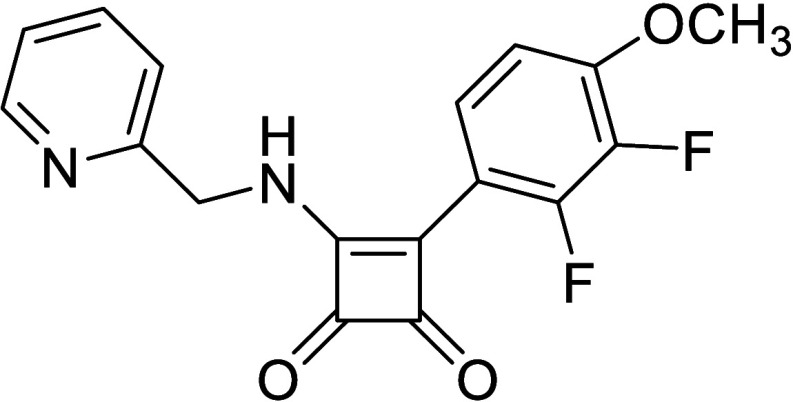



Product **6o** was obtained as a white solid. Yield: 30 mg (93%). ^1^H NMR (400 MHz, DMSO-*d*_6_), 95: 5 mixture of two rotamers. Major rotamer: δ 9.02 (br s, 1H), 8.55 (ddd, *J* = 4.8, 1.7, 0.9 Hz, 1H), 7.85–7.79 (m, 2H), 7.44–7.40 (m, 1H), 7.32 (ddd, *J* = 7.4, 4.8, 0.8 Hz, 1H), 7.25–7.20 (m, 1H), 4.99 (s, 2H), 3.96 (s, 3H). Minor rotamer, characteristic signals: δ 9.89 (br s, 1H), 8.42 (d, *J* = 4.6 Hz, 1H), 7.70 (td, *J* = 7.7, 1.8 Hz, 1H), 7.09–7.03 (m, 1H), 4.57 (s, 2H), 3.90 (s, 3H). ^13^C NMR (101 MHz, DMSO-*d*_6_), 95: 5 mixture of two rotamers. Major rotamer: δ 192.9, 187.6, 178.8, 156.9, 156.1, 150.4 (dd, *J* = 7.5, 2.8 Hz), 149.2, 146.4 (dd, *J* = 250.8, 10.8 Hz), 140.3 (dd, *J* = 246.4, 14.3 Hz), 137.0, 122.6, 122.5, 121.4, 111.1 (d, *J* = 13.4 Hz), 109.8, 57.0, 48.9–48.4 (m). Minor rotamer was not detected. HRMS: *m*/*z*: calcd. for C_17_H_13_F_2_N_2_O_3_^+^: 331.0889 [M + H]^+^; found: 331.0890.

#### 3-(2,3-Difluoro-4-propoxyphenyl)-4-((pyridin-2-ylmethyl)amino)cyclobut-3-ene-1,2-dione **6p**

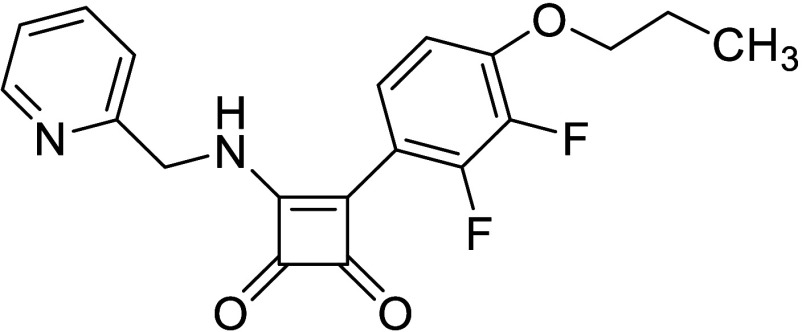



Product **6p** was obtained as a white solid. Yield: 26 mg (83%). ^1^H NMR (400 MHz, DMSO-*d*_6_), 95: 5 mixture of two rotamers. Major rotamer: δ 9.01 (br s, 1H), 8.55 (d, *J* = 4.4 Hz, 1H), 7.85–7.77 (m, 2H), 7.42 (d, *J* = 7.8 Hz, 1H), 7.32 (dd, *J* = 7.4, 4.8 Hz, 1H), 7.25–7.18 (m, 1H), 4.99 (d, *J* = 4.9 Hz, 2H), 4.14 (t, *J* = 6.5 Hz, 2H), 1.78 (h, *J* = 6.9 Hz, 2H), 0.99 (t, *J* = 7.4 Hz, 3H). Minor rotamer, characteristic signals: δ 9.89 (br s, 1H), 8.42 (d, *J* = 4.7 Hz, 1H), 7.70 (td, *J* = 7.8, 1.9 Hz, 1H), 7.05 (t, *J* = 8.2 Hz, 1H), 4.57 (d, *J* = 4.6 Hz, 2H). ^13^C NMR (101 MHz, DMSO-*d*_6_), 95: 5 mixture of two rotamers. Major rotamer: δ 192.8, 187.6, 178.8, 156.9, 156.1, 149.9 (dd, *J* = 7.3, 3.0 Hz), 149.2, 146.5 (dd, *J* = 251.0, 11.0 Hz), 140.4 (dd, *J* = 246.1, 13.9 Hz), 137.0, 122.6, 122.4 (t, *J* = 4.2 Hz), 121.4, 111.0 (d, *J* = 13.1 Hz), 110.5, 71.0, 48.7, 21.8, 10.1. Minor rotamer was not detected. HRMS: *m*/*z*: calcd. for C_19_H_17_F_2_N_2_O_3_^+^: 359.1202 [M + H]^+^; found: 359.1199.

#### 3-(4-Butoxy-2,3-difluorophenyl)-4-((pyridin-2-ylmethyl)amino)cyclobut-3-ene-1,2-dione **6q**

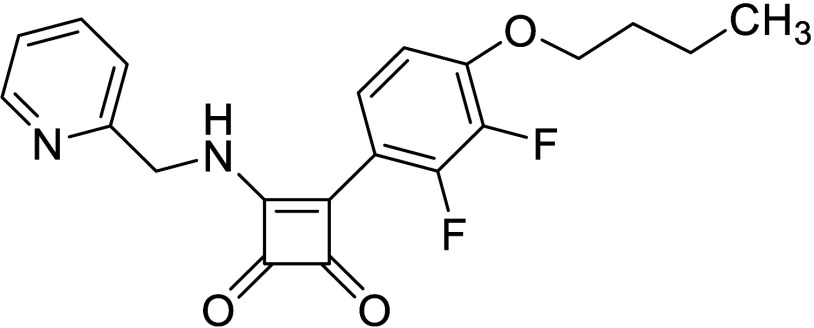



Product **6q** was obtained as a white solid. Yield: 28 mg (89%). ^1^H NMR (400 MHz, DMSO-*d*_6_), 95: 5 mixture of two rotamers. Major rotamer: δ 9.05–8.96 (m, 1H), 8.55 (ddd, *J* = 4.6, 1.5, 0.7 Hz, 1H), 7.84–7.78 (m, 2H), 7.42 (d, *J* = 7.8 Hz, 1H), 7.32 (ddd, *J* = 7.4, 4.7, 1.1 Hz, 1H), 7.25–7.20 (m, 1H), 4.98 (d, *J* = 6.2 Hz, 2H), 4.18 (t, *J* = 6.5 Hz, 2H), 1.75 (p, *J* = 7.2 Hz, 2H), 1.45 (dd, *J* = 116.7, 7.7 Hz, 2H), 0.94 (t, *J* = 7.4 Hz, 3H). Minor rotamer, characteristic signals: δ 9.89 (t, *J* = 6.0 Hz, 1H), 8.42 (d, *J* = 5.2 Hz, 1H), 7.70 (td, *J* = 7.7, 1.8 Hz, 1H), 4.57 (d, *J* = 6.1 Hz, 2H), 4.12 (t, *J* = 6.5 Hz, 2H). ^13^C NMR (101 MHz, DMSO-*d*_6_), 95: 5 mixture of two rotamers. Major rotamer: δ 230.5, 225.2, 216.5, 194.5, 193.8, 187.5 (dd, *J* = 7.6, 2.7 Hz), 186.8 (d, *J* = 11.2 Hz), 184.2 (dd, *J* = 251.7, 11.3 Hz), 178.0 (dd, *J* = 245.3, 14.0 Hz), 174.6 (t, *J* = 10.0 Hz), 160.2 (t, *J* = 15.6 Hz), 160.0, 159.1 (d, *J* = 19.5 Hz), 148.6 (d, *J* = 13.3 Hz), 148.2 (d, *J* = 15.3 Hz), 107.0, 86.3, 68.0, 56.2, 51.2 (d, *J* = 9.5 Hz). Minor rotamer was not detected. HRMS: *m*/*z*: calcd. for C_20_H_19_F_2_N_2_O_3_^+^: 373.1358 [M + H]^+^; found: 373.1361.

#### 3-(4-Ethoxy-3,5-dimethylphenyl)-4-((pyridin-2-ylmethyl)amino)cyclobut-3-ene-1,2-dione **6r**

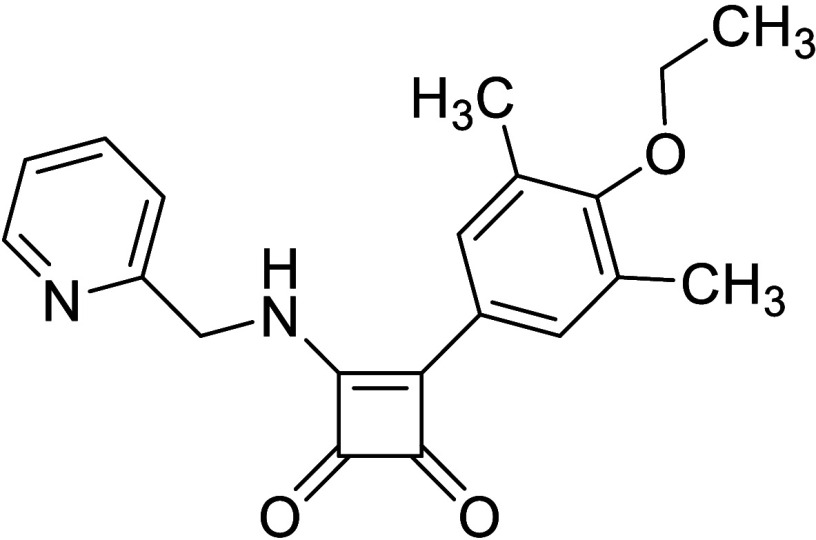



Product **6r** was obtained as a white solid. Yield: 24 mg (74%). ^1^H NMR (400 MHz, DMSO-*d*_6_) δ 9.49 (t, *J* = 6.2 Hz, 1H), 8.56 (ddd, J = 4.8, 1.7, 0.9 Hz, 1H), 7.82 (td, *J* = 7.7, 1.8 Hz, 1H), 7.75 (s, 2H), 7.45 (d, *J* = 7.9 Hz, 1H), 7.33 (ddd, *J* = 7.6, 4.9, 1.1 Hz, 1H), 5.02 (d, *J* = 6.3 Hz, 2H), 3.86 (q, *J* = 7.0 Hz, 2H), 2.27 (s, 6H), 1.35 (t, *J* = 7.0 Hz, 3H). ^13^C NMR (126 MHz, DMSO-*d*_6_) δ 193.0, 188.8, 177.0, 162.0, 157.8, 157.1, 149.3, 137.1, 131.5, 126.8, 124.7, 122.7, 121.6, 67.8, 48.8, 15.9, 15.6. HRMS: *m*/*z*: calcd. for C_20_H_21_N_2_O_3_^+^: 337.1547 [M + H]^+^; found: 337.1549.

#### 3-(Naphthalen-1-yl)-4-((pyridin-2-ylmethyl)amino)cyclobut-3-ene-1,2-dione **6s**

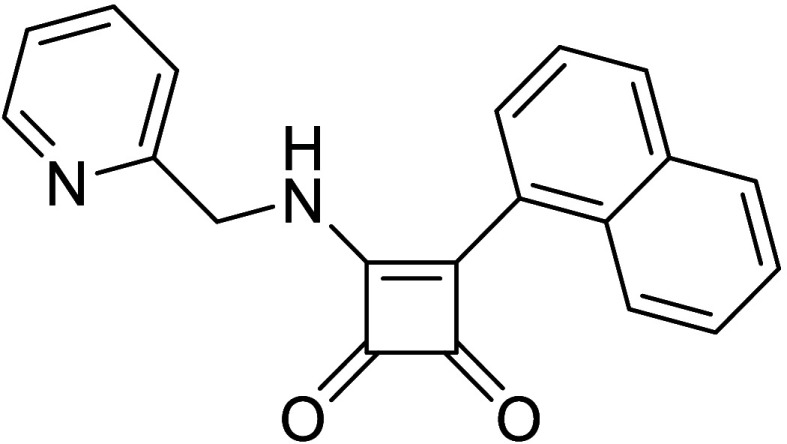



Product **6s** was obtained as a brown oil. Yield: 30 mg (89%). ^1^H NMR (400 MHz, DMSO-*d*_6_), 79: 21 mixture of two rotamers. Major rotamer: δ 9.56 (t, *J* = 5.3 Hz, 1H), 8.58 (ddd, *J* = 4.9, 1.5, 1.0 Hz, 1H), 8.15–8.10 (m, 1H), 8.07 (d, *J* = 7.9 Hz, 1H), 8.05–7.99 (m, 1H), 7.83 (td, *J* = 7.7, 1.8 Hz, 1H), 7.72–7.59 (m, 4H), 7.48 (d, *J* = 7.6 Hz, 1H), 7.33 (ddd, *J* = 7.6, 4.9, 1.1 Hz, 1H), 5.01 (d, *J* = 5.6 Hz, 2H). Minor rotamer, characteristic signals: δ 9.89 (t, *J* = 6.7 Hz, 1H), 8.27 (d, *J* = 4.1 Hz, 1H), 7.06 (dd, *J* = 7.1, 5.2 Hz, 1H), 6.97 (d, *J* = 7.9 Hz, 1H), 4.37 (d, *J* = 6.0 Hz, 2H). ^13^C NMR (126 MHz, DMSO-*d*_6_), 79: 21 mixture of two rotamers. Major rotamer δ 193.8, 188.3, 181.4, 164.3, 157.0, 149.2, 137.0, 133.3, 130.4, 129.4, 128.4, 126.7, 126.5, 126.5, 126.4, 125.9, 125.5, 122.7, 121.6, 48.9. Minor rotamer, characteristic signals: δ 189.1, 182.1, 155.6, 148.9, 136.5, 132.9, 128.1, 125.1, 122.3, 121.1, 49.0. HRMS: *m*/*z*: calcd. for C_20_H_15_N_2_O_2_^+^: 315.1128 [M + H]^+^; found: 315.1123.

#### 3-(4-Methoxynaphthalen-1-yl)-4-((pyridin-2-ylmethyl)amino)cyclobut-3-ene-1,2-dione **6t**

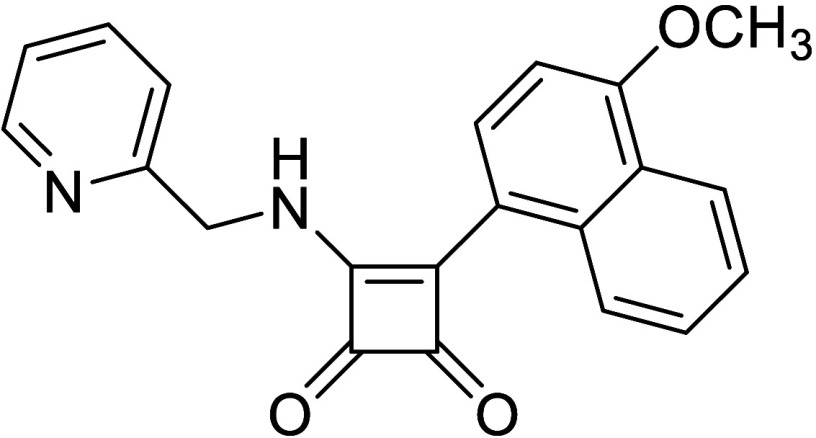



Product **6t** was obtained as a white solid. Yield: 30 mg (92%). ^1^H NMR (400 MHz, DMSO-*d*_6_), 83: 17 mixture of two rotamers. Major rotamer: δ 9.46 (br s, 1H), 8.57 (ddd, *J* = 4.8, 1.9, 1.1 Hz, 3H), 8.28–8.22 (m, 1H), 8.19–8.13 (m, 1H), 7.82 (td, *J* = 7.7, 1.8 Hz, 1H), 7.71 (d, *J* = 8.1 Hz, 1H), 7.66–7.56 (m, 2H), 7.47 (d, *J* = 7.8 Hz, 1H), 7.33 (ddd, *J* = 7.6, 4.9, 1.1 Hz, 1H), 7.14 (d, *J* = 8.2 Hz, 1H), 5.01 (s, 2H), 4.05 (s, 3H). Minor rotamer, characteristic signals: δ ^1^H NMR (400 MHz, DMSO-*d*_6_) δ 9.78 (br s, 1H), 8.32 (d, *J* = 4.2 Hz, 1H), 7.03 (d, *J* = 7.8 Hz, 1H), 6.99 (d, *J* = 8.0 Hz, 1H), 4.41 (s, 2H), 4.00 (s, 3H). ^13^C NMR (126 MHz, DMSO-*d*_6_), 83: 17 mixture of two rotamers. Major rotamer δ 193.4, 188.4, 181.0, 164.9, 157.2, 156.8, 149.2, 137.0, 130.6, 127.3, 127.2, 126.6, 126.0, 125.0, 122.7, 121.8, 121.6, 119.0, 104.2, 56.1, 48.9. Minor rotamer, characteristic signals: δ 182.1, 156.0, 155.8, 154.4, 148.9, 126.9, 125.8, 122.3, 121.2, 103.9, 55.9, 49.1. HRMS: *m*/*z*: calcd. for C_21_H_17_N_2_O_3_^+^: 345.1234 [M + H]^+^; found: 345.1233.

#### 3-(Benzo[*d*][1,3]dioxol-5-yl)-4-((pyridin-2-ylmethyl)amino)cyclobut-3-ene-1,2-dione **6u**

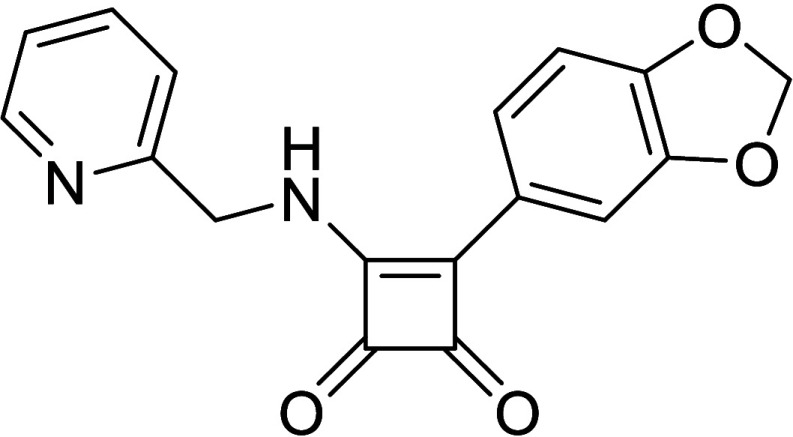



Product **6u** was obtained as a pale-yellow solid. Yield: 32 mg (95%). ^1^H NMR (400 MHz, DMSO-*d*_6_) δ 9.52 (t, *J* = 6.2 Hz, 1H), 8.56 (ddd, *J* = 4.8, 1.7, 0.9 Hz, 1H), 7.81 (td, *J* = 7.7, 1.8 Hz, 1H), 7.70 (dd, *J* = 8.1, 1.7 Hz, 1H), 7.59 (d, *J* = 1.6 Hz, 1H), 7.45 (d, *J* = 7.9 Hz, 1H), 7.32 (ddd, *J* = 7.5, 4.8, 1.0 Hz, 1H), 7.13 (d, *J* = 8.2 Hz, 1H), 6.13 (s, 2H), 5.01 (d, *J* = 6.1 Hz, 2H). ^13^C NMR (126 MHz, DMSO-*d*_6_) δ 192.5, 188.7, 178.4, 161.7, 157.1, 149.2, 137.0, 123.3, 122.7, 121.6, 109.1, 105.8, 101.8, 48.8. HRMS: *m*/*z*: calcd. for C_17_H_13_N_2_O_4_^+^: 309.0870 [M + H]^+^; found: 309.0870.

#### 3-(2,3-Dihydrobenzo[*b*][1,4]dioxin-6-yl)-4-((pyridin-2-ylmethyl)amino)cyclobut-3-ene-1,2-dione **6v**

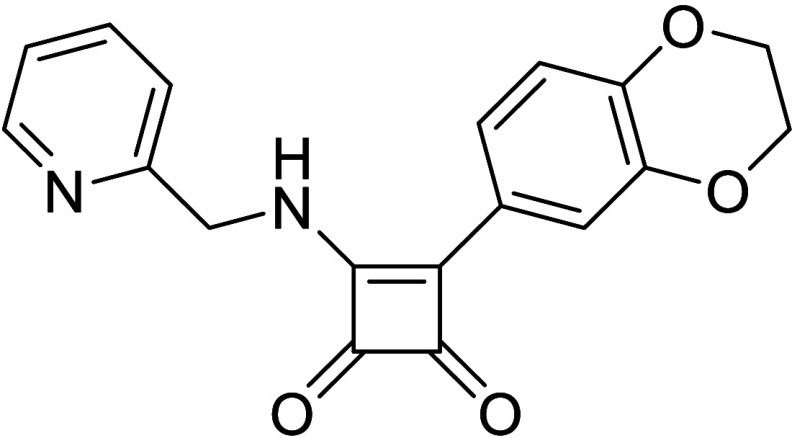



Product **6v** was obtained as a pale-yellow solid. Yield: 32 mg (98%). ^1^H NMR (400 MHz, DMSO-*d*_6_) δ ^1^H NMR (400 MHz, DMSO-*d*_6_) δ 9.52 (t, *J* = 6.3 Hz, 1H), 8.55 (ddd, *J* = 4.8, 1.6, 0.8 Hz, 1H), 7.81 (td, *J* = 7.7, 1.9 Hz, 1H), 7.63–7.58 (m, 2H), 7.43 (d, *J* = 7.8 Hz, 1H), 7.32 (ddd, *J* = 7.5, 4.8, 1.1 Hz, 1H), 7.06–6.99 (m, 1H), 5.00 (d, *J* = 6.3 Hz, 2H), 4.37–4.25 (m, 4H). ^13^C NMR (126 MHz, DMSO-*d*_6_) δ 192.6, 188.8, 178.5, 161.8, 157.2, 149.2, 145.7, 143.7, 137.0, 122.7, 122.6, 121.6, 120.4, 117.7, 114.9, 64.5, 64.0, 48.8. HRMS: *m*/*z*: calcd. for C_18_H_15_N_2_O_4_^+^: 323.1026 [M + H]^+^; found: 323.1025.

#### 3-(6-Ethoxynaphthalen-2-yl)-4-((pyridin-2-ylmethyl)amino)cyclobut-3-ene-1,2-dione **6w**

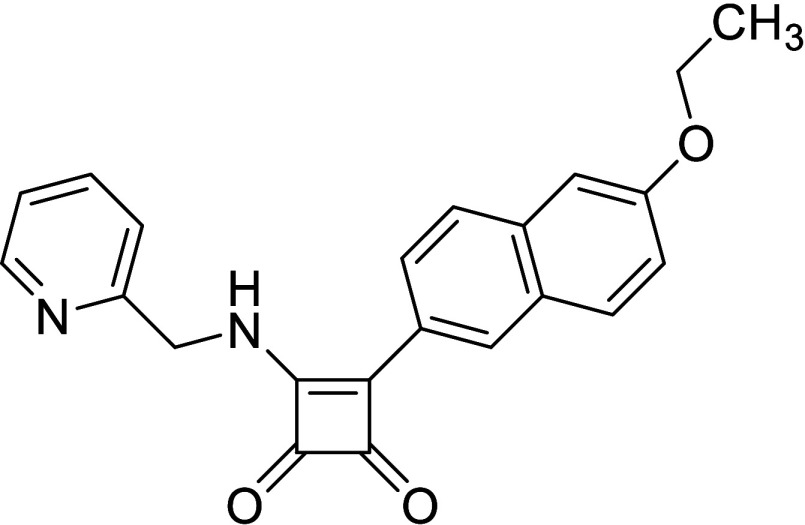



Product **6w** was obtained as a white solid. Yield: 28 mg (89%). ^1^H NMR (400 MHz, DMSO-*d*_6_) δ 9.67 (br s, 1H), 8.57 (ddd, *J* = 4.9, 1.5, 0.7 Hz, 1H), 8.53 (s, 1H), 8.10 (dd, *J* = 8.5, 1.8 Hz, 1H), 7.91 (dd, *J* = 10.8, 8.9 Hz, 2H), 7.83 (td, *J* = 7.7, 1.8 Hz, 1H), 7.49 (d, *J* = 7.9 Hz, 1H), 7.37 (d, *J* = 2.5 Hz, 1H), 7.33 (dd, *J* = 7.5, 4.8 Hz, 1H), 7.23 (dd, *J* = 8.9, 2.4 Hz, 1H), 5.07 (s, 2H), 4.18 (q, *J* = 6.9 Hz, 2H), 1.41 (t, *J* = 6.9 Hz, 3H). ^13^C NMR (101 MHz, DMSO-*d*_6_) δ 193.0, 189.0, 179.1, 162.2, 158.0, 157.1, 149.3, 137.1, 135.2, 130.3, 128.0, 127.4, 126.0, 124.5, 123.7, 122.7, 121.7, 119.8, 106.9, 63.3, 49.0, 14.6. HRMS: *m*/*z*: calcd. for C_22_H_19_N_2_O_3_^+^: 359.1390 [M + H]^+^; found: 359.1386.

#### 3-(Furan-3-yl)-4-((pyridin-2-ylmethyl)amino)cyclobut-3-ene-1,2-dione **6x**

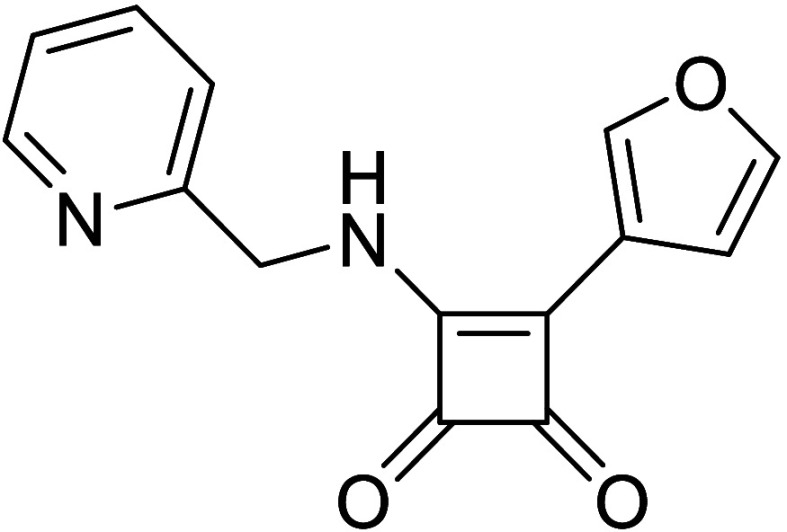



Product **6x** was obtained as a white solid. Yield: 30 mg (84%). ^1^H NMR (400 MHz, DMSO-*d*_6_) δ 9.40 (t, *J* = 5.5 Hz, 1H), 8.56 (d, *J* = 4.7 Hz, 1H), 8.49 (s, 1H), 7.96–7.88 (m, 1H), 7.82 (td, *J* = 7.7, 1.9 Hz, 1H), 7.45 (d, *J* = 7.9 Hz, 1H), 7.33 (dd, *J* = 7.6, 4.6 Hz, 1H), 7.15–7.11 (m, 1H), 4.99 (d, *J* = 6.1 Hz, 2H). ^13^C NMR (101 MHz, DMSO-*d*_6_) δ 192.2, 188.0, 178.4, 157.0, 156.7, 149.3, 145.1, 142.8, 137.1, 122.8, 121.7, 114.7, 107.5, 48.8. HRMS: *m*/*z*: calcd. for C_14_H_11_N_2_O_3_^+^: 255.0764 [M + H]^+^; found: 255.0764.

#### 3-((Pyridin-2-ylmethyl)amino)-4-(thiophen-3-yl)cyclobut-3-ene-1,2-dione **6y**

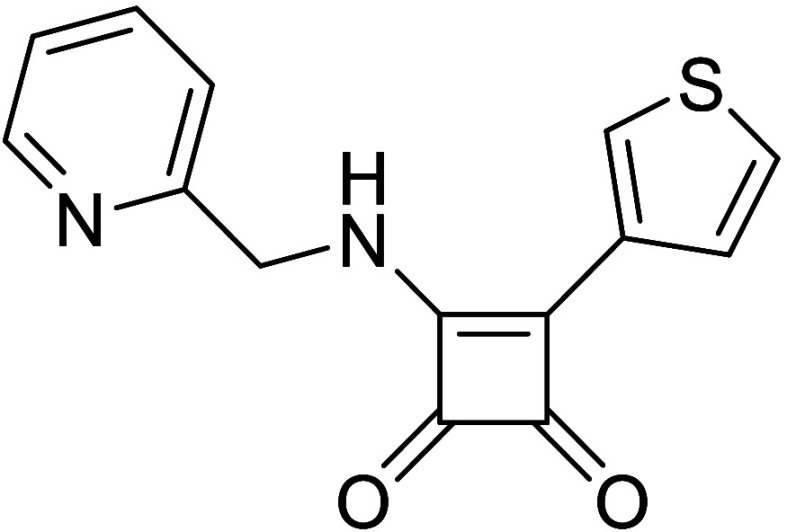



Product **6y** was obtained as a white solid. Yield: 34 mg (97%). ^1^H NMR (400 MHz, DMSO-*d*_6_) δ 9.53 (s, 1H), 8.60–8.51 (m, 1H), 8.38 (t, *J* = 2.0 Hz, 1H), 7.87–7.75 (m, 3H), 7.45 (d, *J* = 7.8 Hz, 1H), 7.33 (dd, *J* = 7.0, 5.2 Hz, 1H), 5.01 (s, 2H). ^13^C NMR (126 MHz, DMSO-*d*_6_) δ 192.9, 188.0, 178.1, 158.3, 157.0, 149.3, 137.1, 129.6, 127.9, 126.8, 125.2, 122.7, 121.7, 48.8. HRMS: *m*/*z*: calcd. for C_14_H_11_N_2_O_2_S^+^: 271.0536 [M + H]^+^; found: 271.0535.

#### 3-(5-Methylthiophen-2-yl)-4-((pyridin-2-ylmethyl)amino)cyclobut-3-ene-1,2-dione **6z**

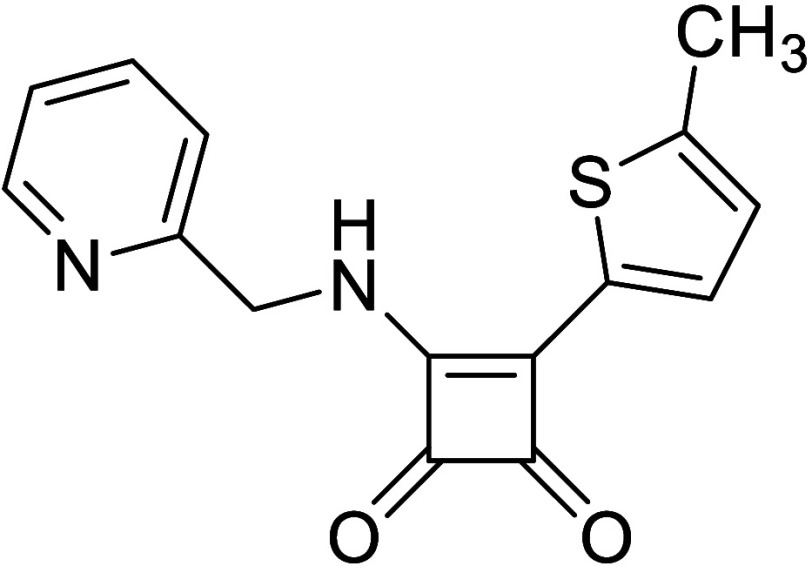



Product **6z** was obtained as a pale-yellow solid. Yield: 30 mg (89%). ^1^H NMR (400 MHz, DMSO-*d*_*6*_) δ 9.49 (s, 1H), 8.56 (ddd, *J* = 4.8, 1.7, 0.9 Hz, 1H), 7.82 (td, *J* = 7.7, 1.8 Hz, 1H), 7.71 (d, *J* = 3.9 Hz, 1H), 7.44 (d, *J* = 7.8 Hz, 1H), 7.33 (ddd, *J* = 7.5, 4.8, 1.2 Hz, 1H), 7.06 (dd, *J* = 3.7, 1.0 Hz, 1H), 5.00 (s, 2H), 2.55 (s, 3H). ^13^C NMR (101 MHz, DMSO-*d*_*6*_) δ 190.7, 186.7, 176.6, 157.1, 157.0, 149.3, 146.0, 137.1, 128.9, 127.4, 127.0, 122.7, 121.7, 48.9, 15.3. HRMS: *m*/*z*: calcd. for C_15_H_13_N_2_O_2_S^+^: 285.0692 [M + H]^+^; found: 285.0691.

### Cytotoxicity

*In vitro* cytotoxicity was evaluated on the MRC-5 *Homo sapiens* long fibroblast cell line (ATCC CCL-171). The MRC-5 cells were cultured in 75 cm^2^ sterile Dulbecco’s modified Eagle’s medium (DMEM) supplemented with 10% heat-inactivated fetal calf serum in a 5% CO_2_ atmosphere at 37 °C. When a semiconfluent layer of cells was formed, the cells were trypsinized, washed with sterile PBS, seeded into a transparent, flat-bottomed 96-well plate at a density of 4 × 10^4^ cells per well and left for recovery at 37 °C, 5% CO_2_ for at least 24 h. For each compound, a 2-fold serial dilution was made in complete DMEM with final concentrations ranging from 128 μM to 0.5 μM. Subsequently, the MRC-5 cells were exposed to the compounds by adding 100 μL of the serial dilutions to the wells. Test plates were incubated for 3 days in an atmosphere of 5% CO_2_ at 37 °C. For the resazurin assay, the cells were washed 2 times with 200 μL PBS, and 100 μL resazurin working solution was added per well. Subsequently, the plates were incubated at 37 °C, 5% CO_2_ for 3 h. To monitor the viable cell number after compound exposure, each well was analyzed using a microplate fluorometer equipped with a 560 nm excitation/590 nm emission filter set. Tamoxifen was included as a positive control (IC_50_ = 12.29 μM).

### Antimycobacterial Activity against *Mycobacterium tuberculosis*

*M. tuberculosis* H37Ra (ATCC 25117) harboring reporter plasmid pSMT1 encoding for *Vibrio harvei* luciferase (47) (H37Ra^lux^) was grown in 7H9 media supplemented with 10% oleic albumin dextrose catalase (Becton-Dickinson, cat. #212351), 0.2% glycerol, 0.05% tyloxapol and 100-μg/mL Hygromycin B (Roche, cat.# 10843555001) at 37 °C shaking (150 rpm).

The activity of the inhibitors was determined in a dose–response fashion. Nine 2-fold serial dilutions with a top concentration of 64 μM were spotted on black opaque 96-well plates (Greiner Bio-One, cat. #655077). An inoculum size of 10^4^ relative luminescence unit H37Ra^lux^ in 200 μL was added to each assay well. Columns 11 and 12 on each assay plate served as high (no growth inhibition) and low (100% growth inhibition) controls, respectively. To control evaporation and minimize the well-to-well variations, the outer wells of each plate were filled with sterile water and plates were kept in plastic bags inside a 37 °C and 5% CO_2_ incubator. The luminescence signal was measured at day 7 by injecting 1% decanal, which serves as the luciferase substrate, into each well (25 nL) using the Glomax Discover microplate reader (Promega). The relative luminescence units were normalized to percentages of cell survival. The dose–response curves and the IC_50_ values were generated in GraphPad Prism version 9 by a nonlinear regression model. Bedaquiline (Sigma) was included as a positive control (IC_50_ = 0.01 μM).

### Antimycobacterial Activity against *Mycobacterium abscessus*

Luminescent *Mycobacterium abscessus* (ATCC 25177) was grown until the exponential phase in 7H9 (Sigma) broth supplemented with 10% ADS (albumin-dextrose-saline), 0.2% glycerol, and 0.05% tyloxapol, and 1–5 × 10^5^ CFU/mL of bacteria were seeded in 96-well plates with 4-fold dilutions of the compounds starting from 64 μM. The plates were incubated at 37 °C in a humid atmosphere. After 3 days of incubation, *M. abscessus* viability was assessed by luminescence measurement, for this IVISbriteTM D-luciferin potassium salt (PerkinElmer) at 0.5 mg/mL was added to the wells (10% v/v) before measuring the luminescence using VantaStar (BMG). Moxifloxacin (VWR) was included as a reference compound and showed an IC_50_ value of 1.74 μM.

## Data Availability

The data that supports the findings of this study are available in the Supporting Information of this article.

## Supporting Information Available

NMR spectra (PDF)
NMR spectra of rotamers (Figures 4–7) (ZIP)
