# Supplementary material for: The Liebeskind–Srogl Cross-Coupling Reaction as a Crucial Step in the Synthesis of New Squaramide-Based Antituberculosis Agents
Source: ACS Omega. 2024 Jul 29;9(32):34808–28. doi: 10.1021/acsomega.4c04314 (PMC11325506; doi:10.1021/acsomega.4c04314)

**Supporting information**

# The Liebeskind–Srogl Cross-Coupling Reaction as a Crucial Step in the Synthesis of New Squaramide-based Antituberculosis Agents

Jan Chasák,[a] Laurence Van Moll[b], An Matheeussen[b], Linda De Vooght[b], Paul Cos,[b] Lucie Brulíková[a,*]

[a]Department of Organic Chemistry, Faculty of Science, Palacký University, 17. listopadu 12, 77146, Olomouc, Czech Republic

[b]Laboratory of Microbiology, Parasitology and Hygiene (LMPH), S7, Faculty of Pharmaceutical, Biomedical and Veterinary Sciences, University of Antwerp, Wilrijk, Belgium

*Corresponding author email: lucie.brulikova@upol.cz

**Content:**

**Fig S1.** $^{1}$H NMR spectra of **2** ........ 4
**Fig S2.** $^{1}$H NMR spectra of **4** ........ 5
**Fig S3.** $^{13}$C NMR spectra of **4** ........ 5
**Fig S4.** $^{1}$H NMR spectra of **5a** ........ 6
**Fig S5.** $^{13}$C NMR spectra of **5a** ........ 6
**Fig S6.** $^{1}$H NMR spectra of **6a** ........ 7
**Fig S7.** $^{13}$C NMR spectra of **6a** ........ 7
**Fig S8.** $^{1}$H NMR spectra of **5b** ........ 8
**Fig S9.** $^{13}$C NMR spectra of **5b** ........ 8
**Fig S10.** $^{1}$H NMR spectra of **6b** ........ 9
**Fig S11.** $^{13}$C NMR spectra of **6b** ........ 9
**Fig S12.** $^{1}$H NMR spectra of **5c** ........ 10
**Fig S13.** $^{13}$C NMR spectra of **5c** ........ 10
**Fig S14.** $^{1}$H NMR spectra of **6c** ........ 11
**Fig S15.** $^{13}$C NMR spectra of **6c** ........ 11
**Fig S16.** $^{1}$H NMR spectra of **5d** ........ 12
**Fig S17.** $^{13}$C NMR spectra of **5d** ........ 12
**Fig S18.** $^{1}$H NMR spectra of **6d** ........ 13
**Fig S19.** $^{13}$C NMR spectra of **6d** ........ 13
**Fig S20.** $^{1}$H NMR spectra of **5e** ........ 14
**Fig S21.** $^{13}$C NMR spectra of **5e** ........ 14
**Fig S22.** $^{1}$H NMR spectra of **6e** ........ 15
**Fig S23.** $^{13}$C NMR spectra of **6e** ........ 15
**Fig S24.** $^{1}$H NMR spectra of **5f** ........ 16
**Fig S25.** $^{13}$C NMR spectra of **5f** ........ 16
**Fig S26.** $^{1}$H NMR spectra of **6f** ........ 17
**Fig S27.** $^{13}$C NMR spectra of **6f** ........ 17
**Fig S28.** $^{1}$H NMR spectra of **5g** ........ 18
**Fig S29.** $^{13}$C NMR spectra of **5g** ........ 18
**Fig S30.** $^{1}$H NMR spectra of **6g** ........ 19
**Fig S31.** $^{13}$C NMR spectra of **6g** ........ 19
**Fig S32.** $^{1}$H NMR spectra of **5h** ........ 20

**Fig S33.** $^{13}C$ NMR spectra of **5h** ........ 20
**Fig S34.** $^{1}H$ NMR spectra of **6h** ........ 21
**Fig S35.** $^{13}C$ NMR spectra of **6h** ........ 21
**Fig S36.** $^{1}H$ NMR spectra of **5i** ........ 22
**Fig S37.** $^{13}C$ NMR spectra of **5i** ........ 22
**Fig S38.** $^{1}H$ NMR spectra of **6i** ........ 23
**Fig S39.** $^{13}C$ NMR spectra of **6i** ........ 23
**Fig S40.** $^{1}H$ NMR spectra of **5j** ........ 24
**Fig S41.** $^{13}C$ NMR spectra of **5j** ........ 24
**Fig S42.** $^{1}H$ NMR spectra of **6j** ........ 25
**Fig S43.** $^{13}C$ NMR spectra of **6j** ........ 25
**Fig S44.** $^{1}H$ NMR spectra of **5k** ........ 26
**Fig S45.** $^{13}C$ NMR spectra of **5k** ........ 26
**Fig S46.** $^{1}H$ NMR spectra of **6k** ........ 27
**Fig S47.** $^{13}C$ NMR spectra of **6k** ........ 27
**Fig S48.** $^{1}H$ NMR spectra of **5l** ........ 28
**Fig S49.** $^{13}C$ NMR spectra of **5l** ........ 28
**Fig S50.** $^{1}H$ NMR spectra of **6l** ........ 29
**Fig S51.** $^{13}C$ NMR spectra of **6l** ........ 29
**Fig S52.** $^{1}H$ NMR spectra of **5m** ........ 30
**Fig S53.** $^{13}C$ NMR spectra of **5m** ........ 30
**Fig S54.** $^{1}H$ NMR spectra of **6m** at 25 °C ........ 31
**Fig S55.** $^{1}H$ NMR spectra of **6m** at 45 °C ........ 31
**Fig S56.** $^{1}H$ NMR spectra of **6m** at 65 °C ........ 32
**Fig S57.** $^{1}H$ NMR spectra of **6m** at 85 °C ........ 32
**Fig S58.** $^{1}H$ NMR spectra of **6m** at 105 °C ........ 33
**Fig S59.** $^{19}F$ NMR spectra of **6m** at 25 °C ........ 33
**Fig S60.** $^{19}F$ NMR spectra of **6m** at 105 °C ........ 34
**Fig S61.** $^{13}C$ NMR spectra of **6m** ........ 34
**Fig S62.** $^{1}H$ NMR spectra of **5n** ........ 35
**Fig S63.** $^{13}C$ NMR spectra of **5n** ........ 35
**Fig S64.** $^{1}H$ NMR spectra of **6n** ........ 36
**Fig S65.** $^{13}C$ NMR spectra of **6n** ........ 36
**Fig S66.** $^{1}H$ NMR spectra of **5o** ........ 37
**Fig S67.** $^{13}C$ NMR spectra of **5o** ........ 37
**Fig S68.** $^{1}H$ NMR spectra of **6o** ........ 38
**Fig S69.** $^{13}C$ NMR spectra of **6o** ........ 38
**Fig S70.** $^{1}H$ NMR spectra of **5p** ........ 39
**Fig S71.** $^{13}C$ NMR spectra of **5p** ........ 39
**Fig S72.** $^{1}H$ NMR spectra of **6p** ........ 40
**Fig S73.** $^{13}C$ NMR spectra of **6p** ........ 40
**Fig S74.** $^{1}H$ NMR spectra of **5q** ........ 41
**Fig S75.** $^{13}C$ NMR spectra of **5q** ........ 41
**Fig S76.** $^{1}H$ NMR spectra of **6q** ........ 42
**Fig S77.** $^{13}C$ NMR spectra of **6q** ........ 42
**Fig S78.** $^{1}H$ NMR spectra of **5r** ........ 43
**Fig S79.** $^{13}C$ NMR spectra of **5r** ........ 43
**Fig S80.** $^{1}H$ NMR spectra of **6r** ........ 44
**Fig S81.** $^{13}C$ NMR spectra of **6r** ........ 44
**Fig S82.** $^{1}H$ NMR spectra of **5s** ........ 45
**Fig S83.** $^{13}C$ NMR spectra of **5s** ........ 45
**Fig S84.** $^{1}H$ NMR spectra of **6s** at 25 °C ........ 46
**Fig S85.** $^{1}H$ NMR spectra of **6s** at 45 °C ........ 46

**Fig S86.** $^{1}H$ NMR spectra of **6s** at 65 °C ........ 47
**Fig S87.** $^{1}H$ NMR spectra of **6s** at 85 °C ........ 47
**Fig S88.** $^{1}H$ NMR spectra of **6s** at 105 °C ........ 48
**Fig S89.** $^{13}C$ NMR spectra of **6s** ........ 48
**Fig S90.** $^{1}H$ NMR spectra of **5t** ........ 49
**Fig S91.** $^{13}C$ NMR spectra of **5t** ........ 49
**Fig S92.** $^{1}H$ NMR spectra of **6t** ........ 50
**Fig S93.** $^{13}C$ NMR spectra of **6t** ........ 50
**Fig S94.** $^{1}H$ NMR spectra of **5u** ........ 51
**Fig S95.** $^{13}C$ NMR spectra of **5u** ........ 51
**Fig S96.** $^{1}H$ NMR spectra of **6u** ........ 52
**Fig S97.** $^{13}C$ NMR spectra of **6u** ........ 52
**Fig S98.** $^{1}H$ NMR spectra of **5v** ........ 53
**Fig S99.** $^{13}C$ NMR spectra of **5v** ........ 53
**Fig S100.** $^{1}H$ NMR spectra of **6v** ........ 54
**Fig S101.** $^{13}C$ NMR spectra of **6v** ........ 54
**Fig S102.** $^{1}H$ NMR spectra of **5w** ........ 55
**Fig S103.** $^{13}C$ NMR spectra of **5w** ........ 55
**Fig S104.** $^{1}H$ NMR spectra of **6w** ........ 56
**Fig S105.** $^{13}C$ NMR spectra of **6w** ........ 56
**Fig S106.** $^{1}H$ NMR spectra of **5x** ........ 57
**Fig S107.** $^{13}C$ NMR spectra of **5x** ........ 57
**Fig S108.** $^{1}H$ NMR spectra of **6x** ........ 58
**Fig S109.** $^{13}C$ NMR spectra of **6x** ........ 58
**Fig S110.** $^{1}H$ NMR spectra of **5y** ........ 59
**Fig S111.** $^{13}C$ NMR spectra of **5y** ........ 59
**Fig S112.** $^{1}H$ NMR spectra of **6y** ........ 60
**Fig S113.** $^{13}C$ NMR spectra of **6y** ........ 60
**Fig S114.** $^{1}H$ NMR spectra of **5z** ........ 61
**Fig S115.** $^{13}C$ NMR spectra of **5z** ........ 61
**Fig S116.** $^{1}H$ NMR spectra of **6z** ........ 62
**Fig S117.** $^{13}C$ NMR spectra of **6z** ........ 62
**Fig S118.** $^{1}H$ NMR spectra of **5aa** ........ 63
**Fig S119.** $^{1}H$ NMR spectra of **5ab** ........ 63

**Fig S1.** $^{1}$H NMR spectra of **2**

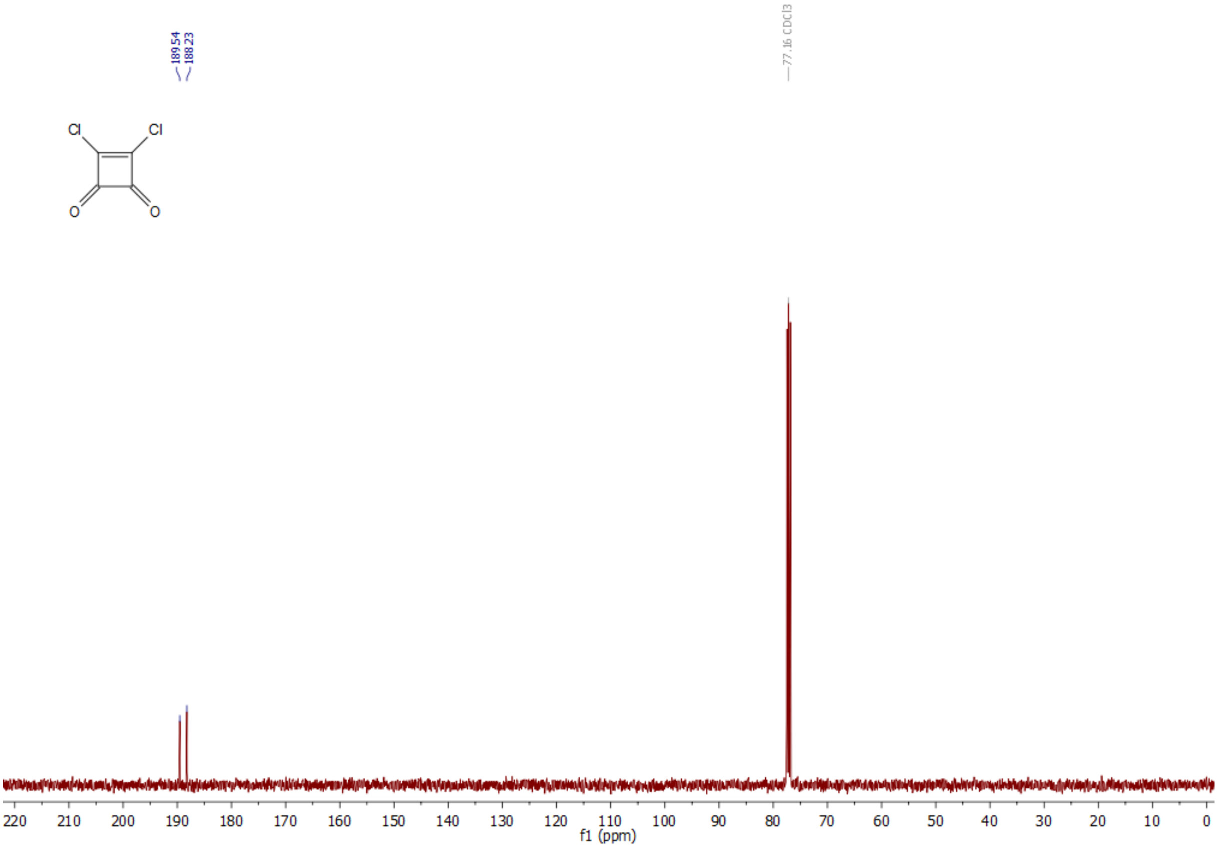

**Fig S2.** $^{1}$H NMR spectra of **4**

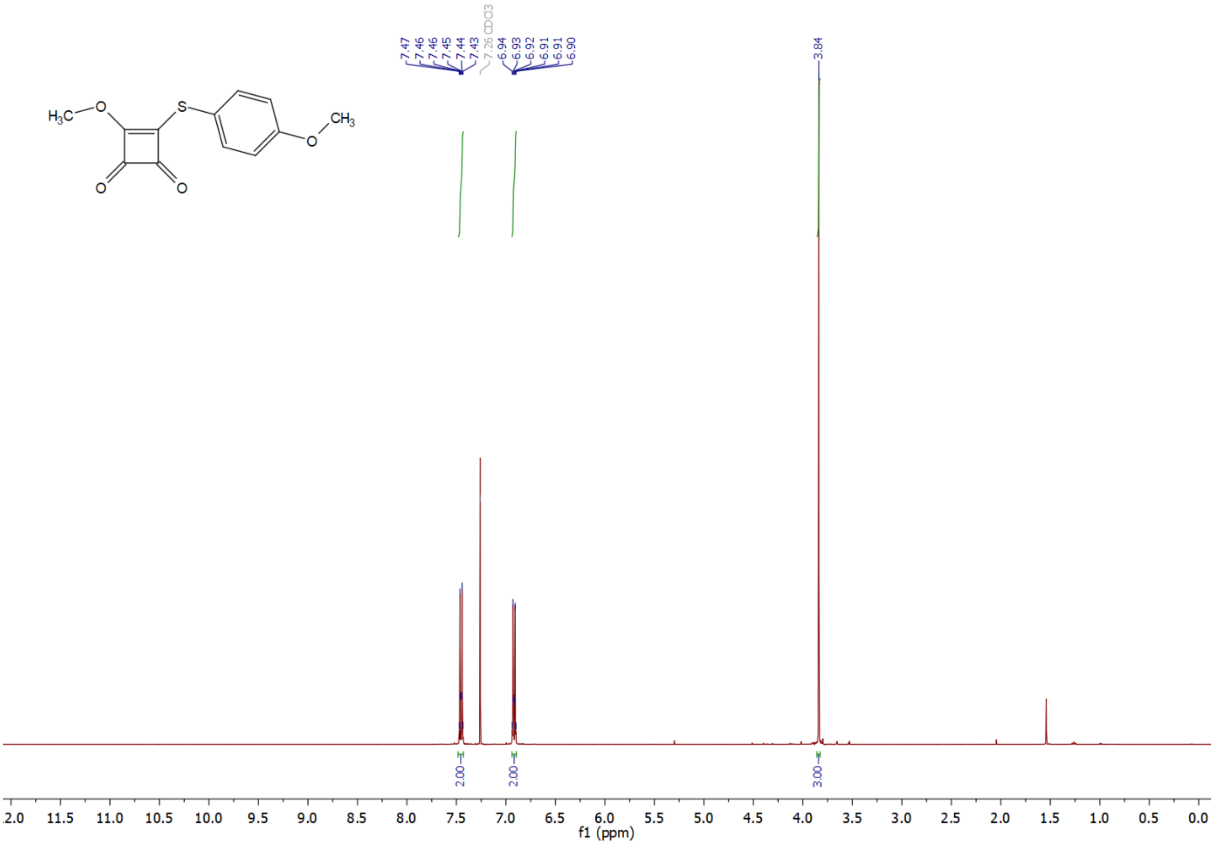


**Fig S3.** $^{13}$C NMR spectra of **4**

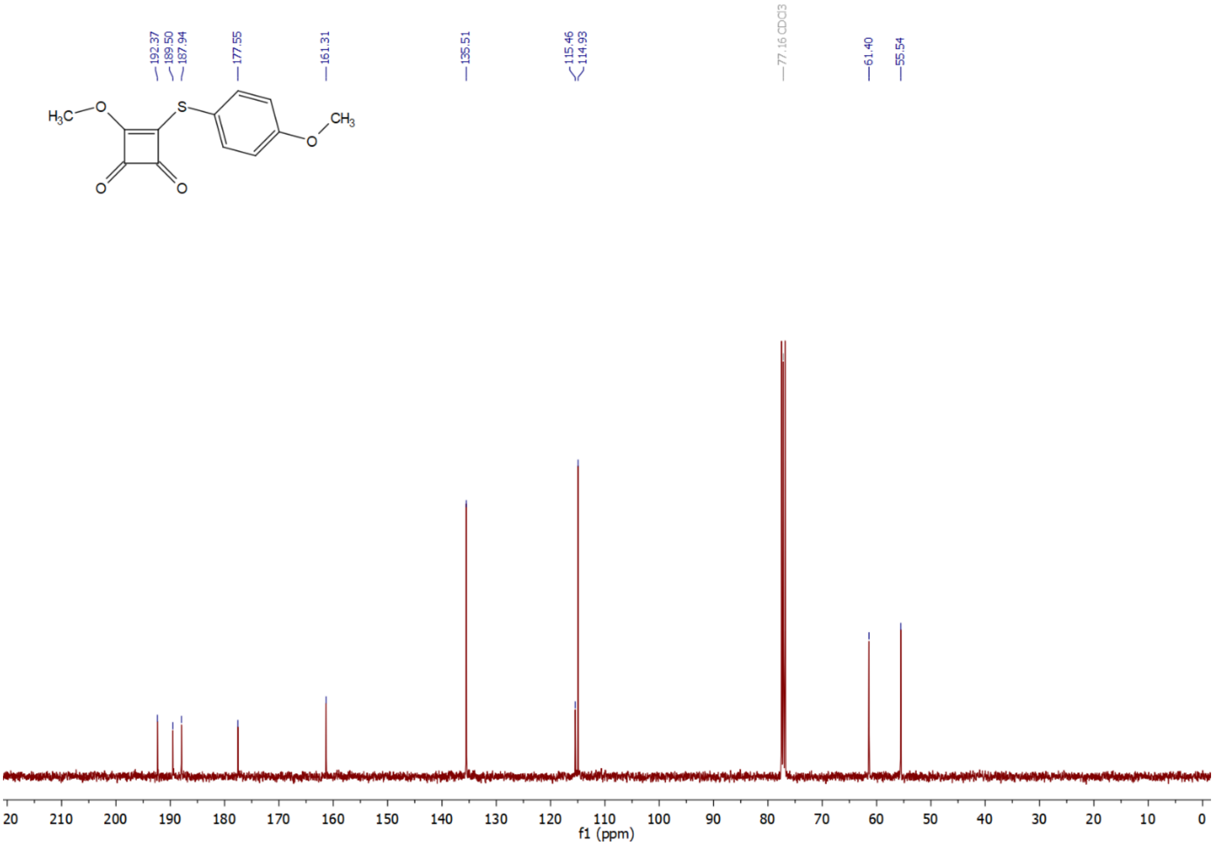

**Fig S4.** $^{1}$H NMR spectra of **5a**

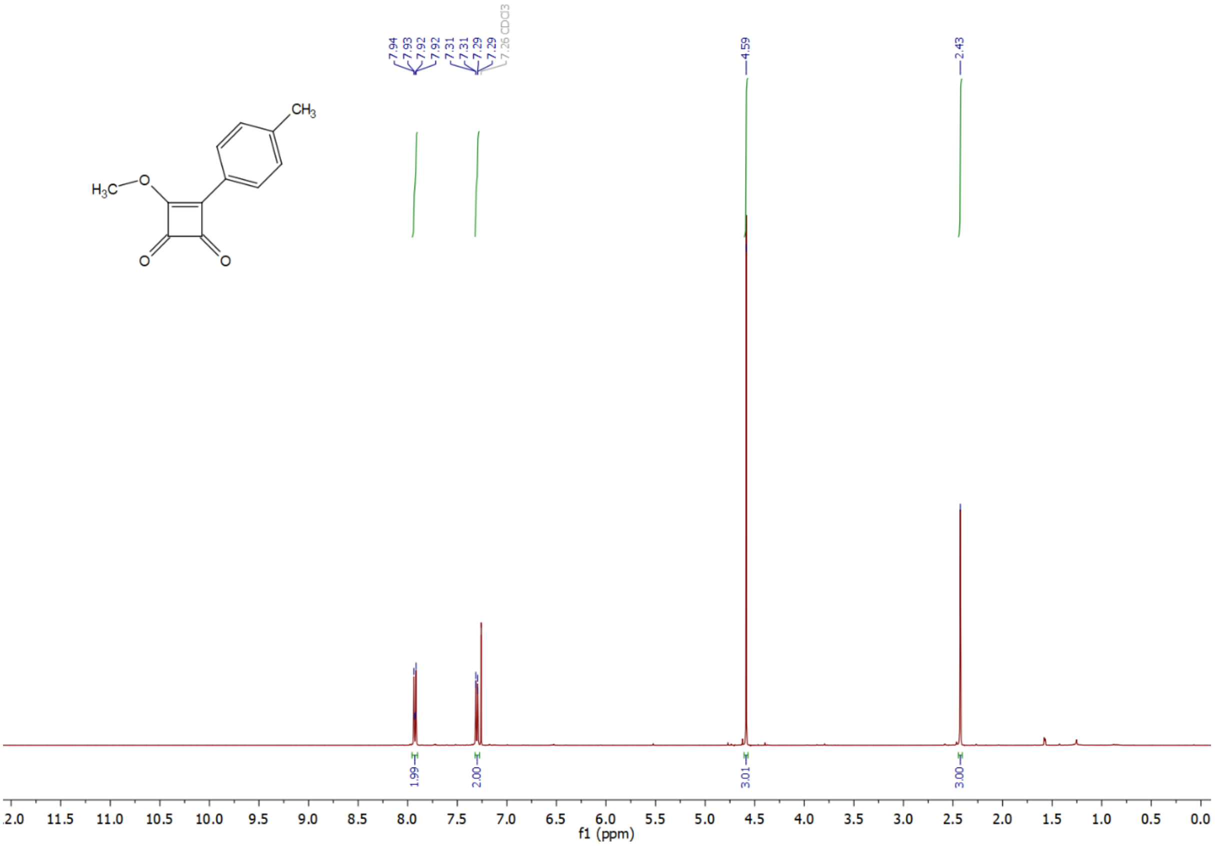


**Fig S5.** $^{13}$C NMR spectra of **5a**

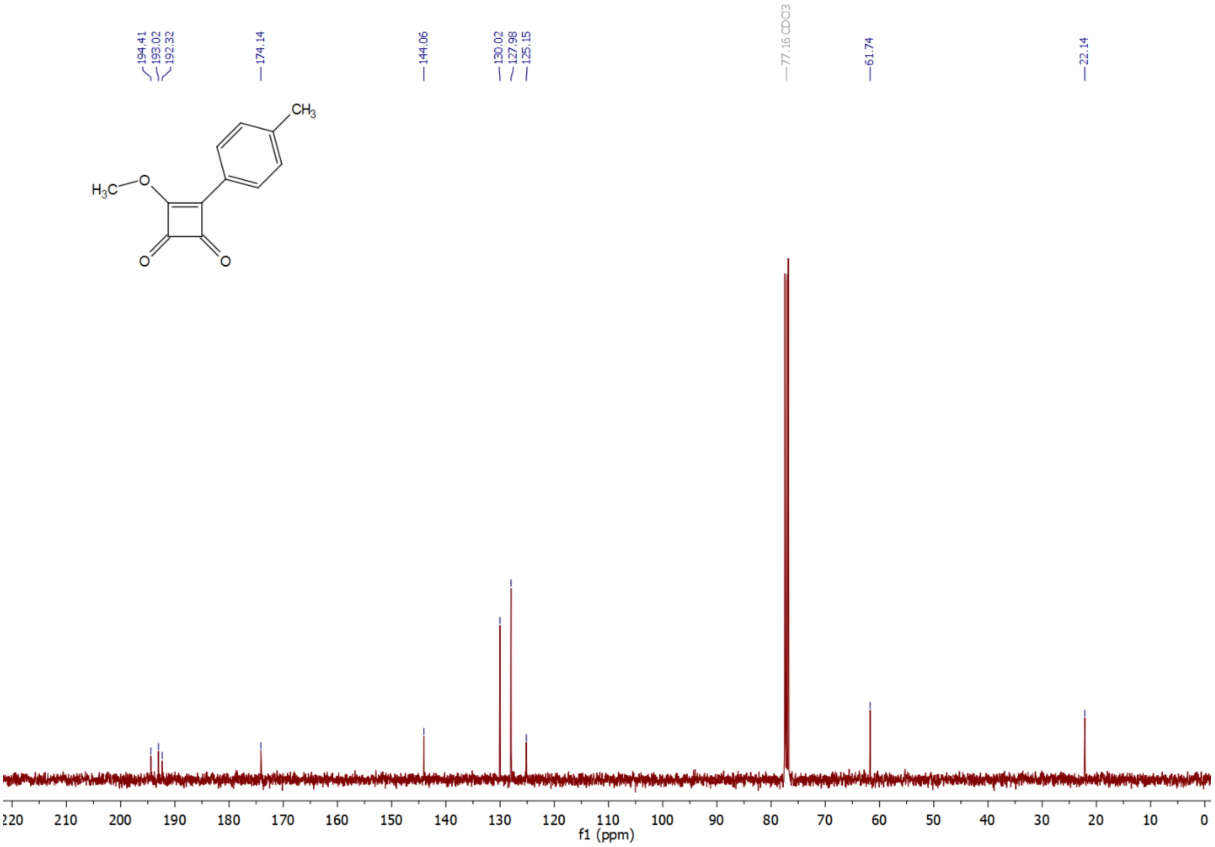

**Fig S6.** $^{1}$H NMR spectra of **6a**

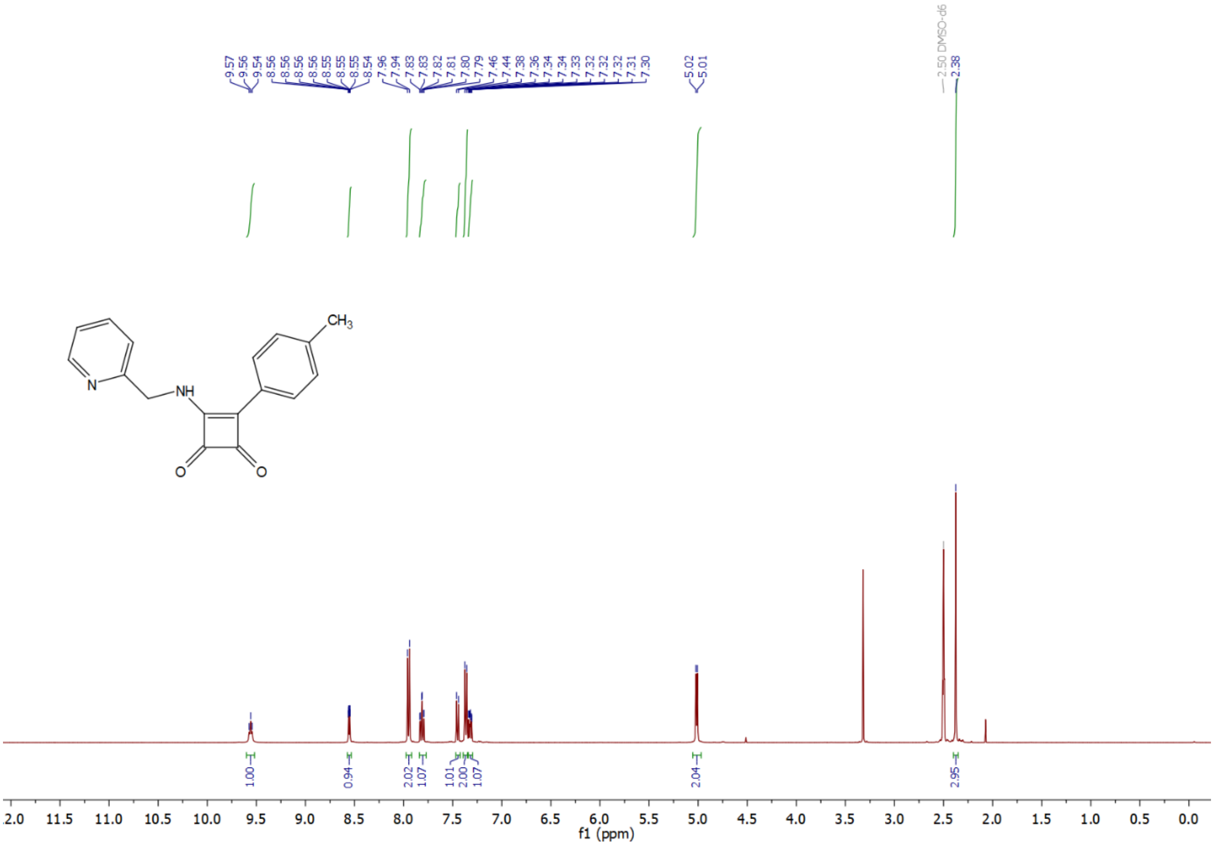

**Fig S7.** $^{13}$C NMR spectra of **6a**

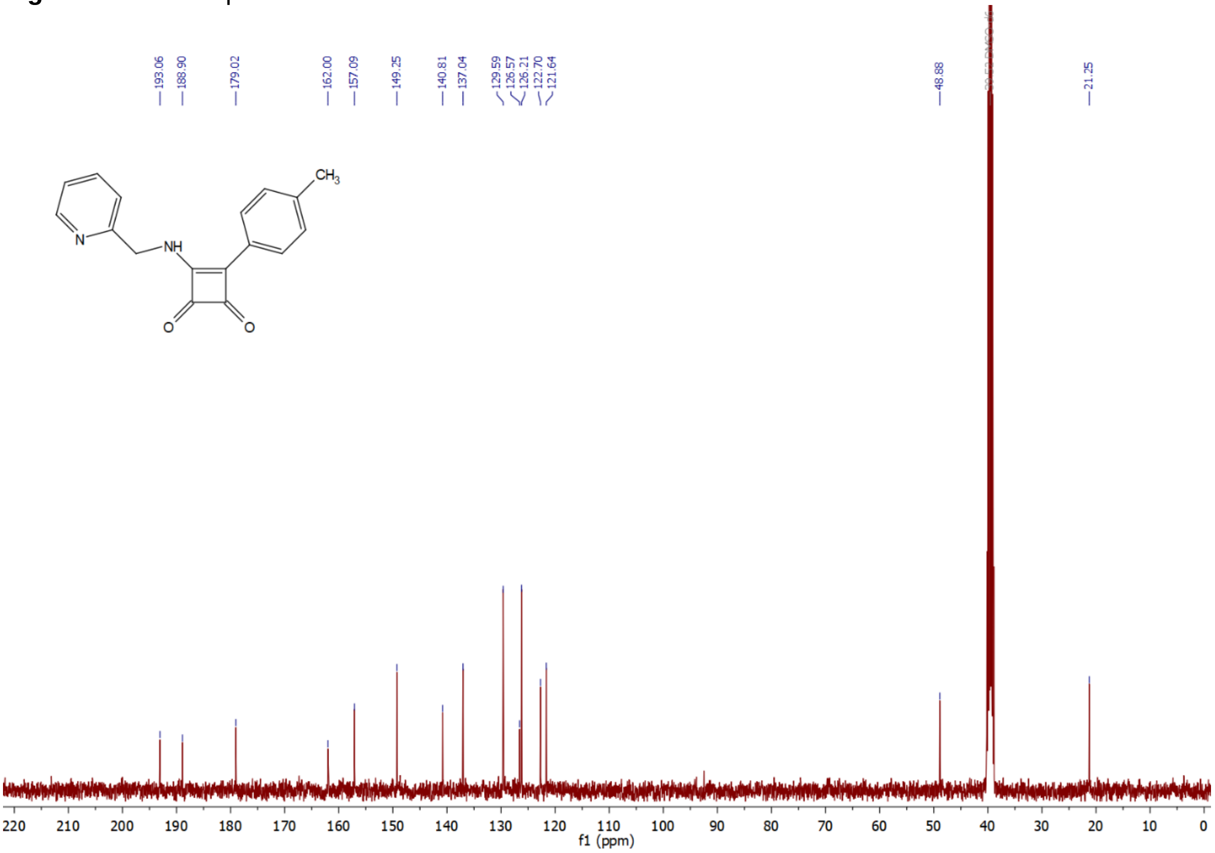

**Fig S8.** $^{1}$H NMR spectra of **5b**

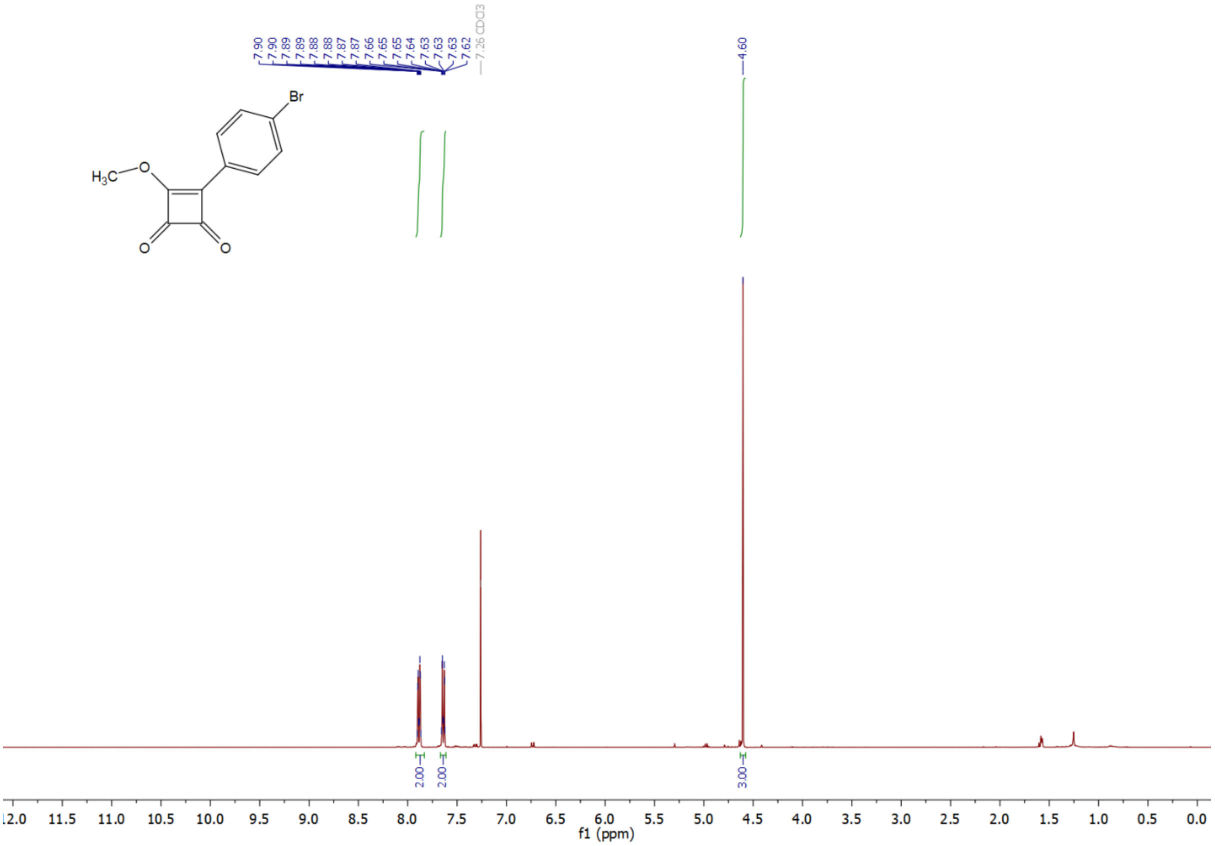


**Fig S9.** $^{13}$C NMR spectra of **5b**

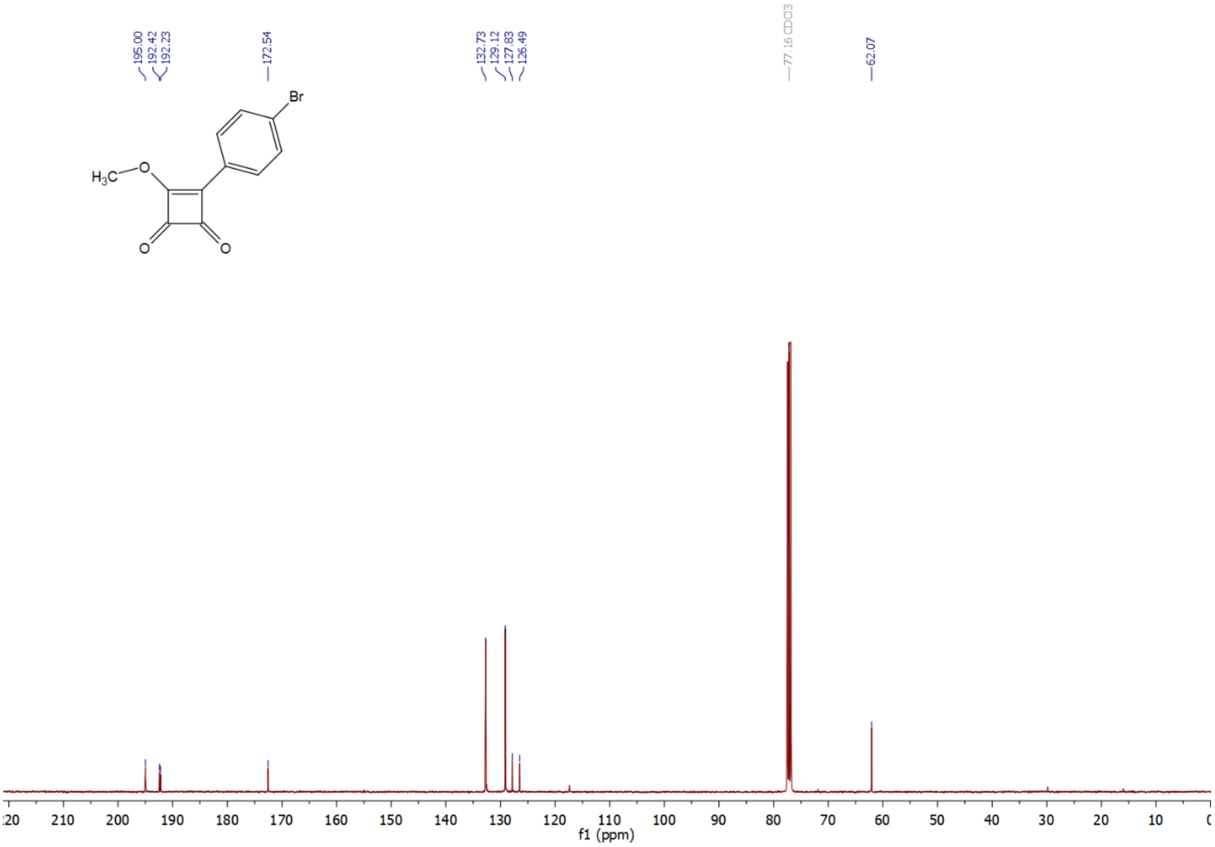

**Fig S10.** $^{1}H$ NMR spectra of **6b**

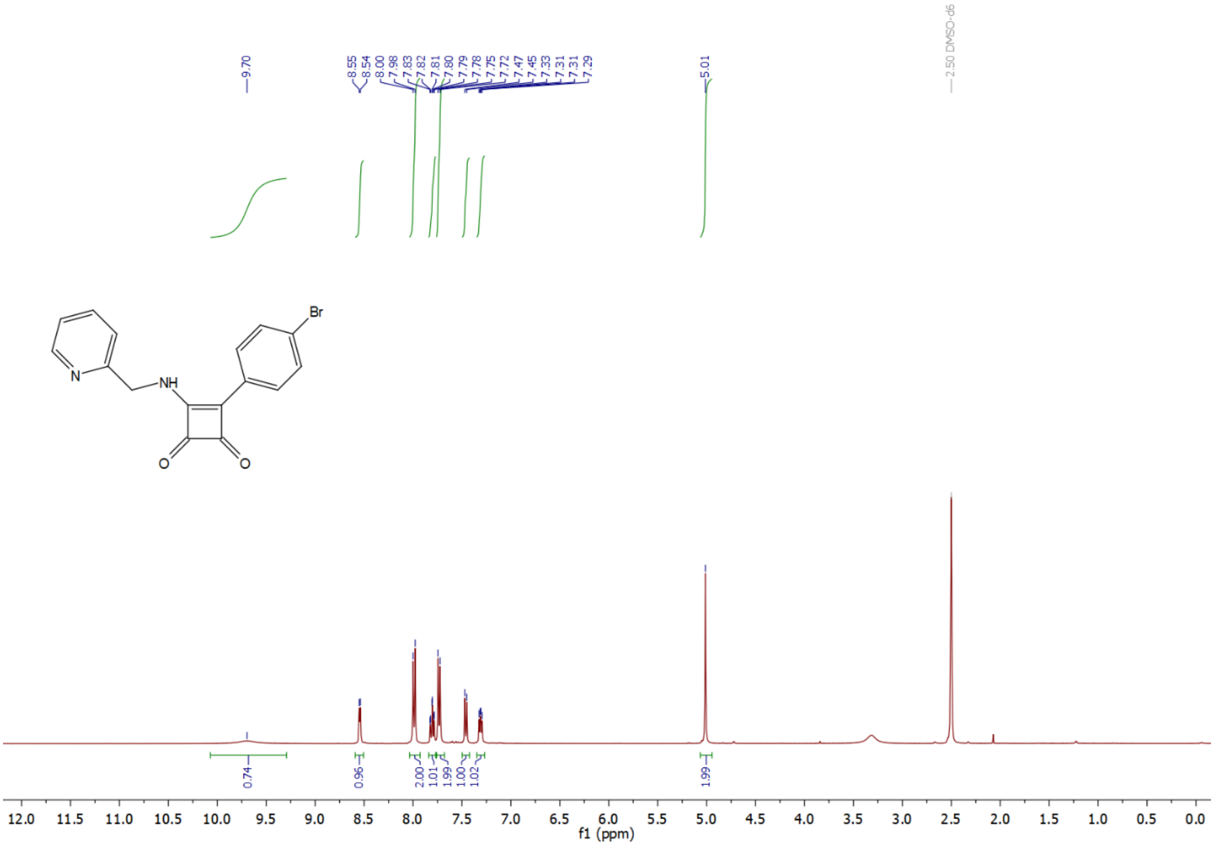

**Fig S11.** $^{13}C$ NMR spectra of **6b**

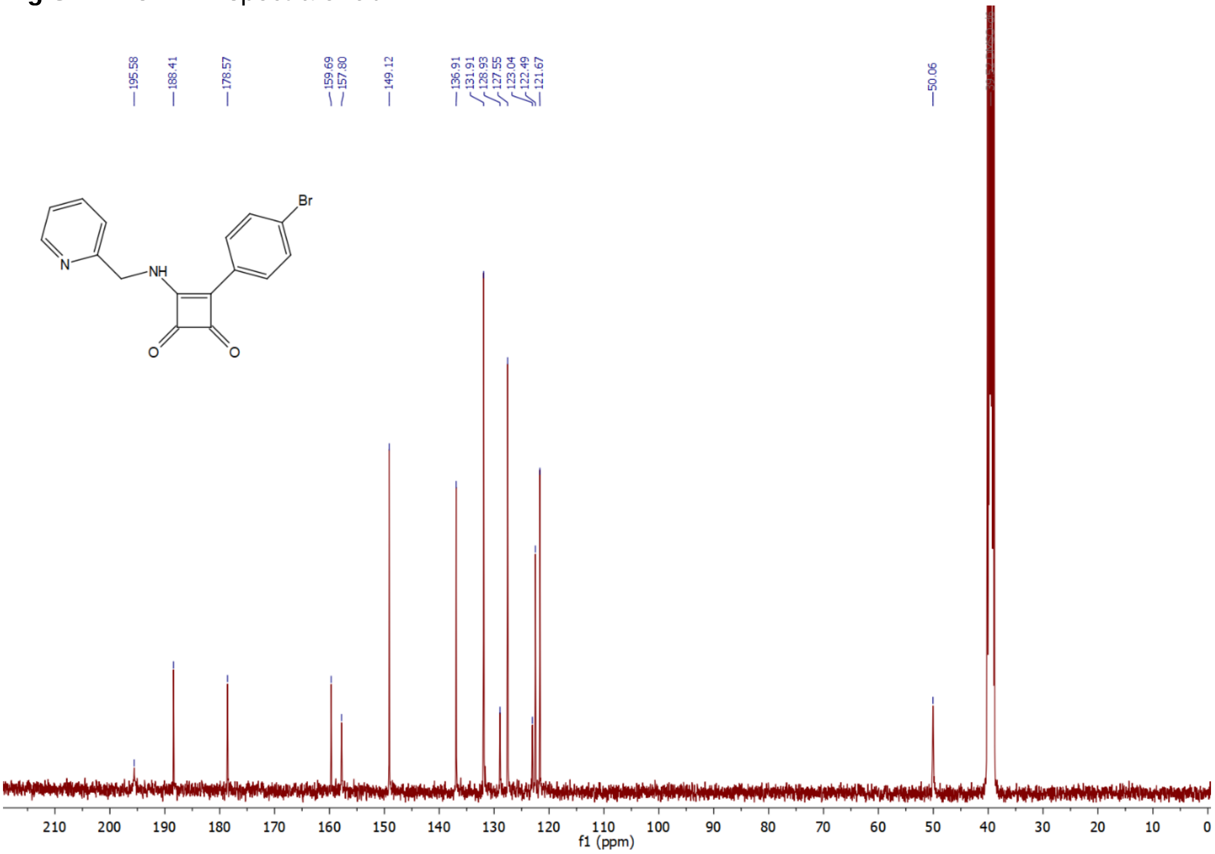

**Fig S12.** $^{1}H$ NMR spectra of **5c**

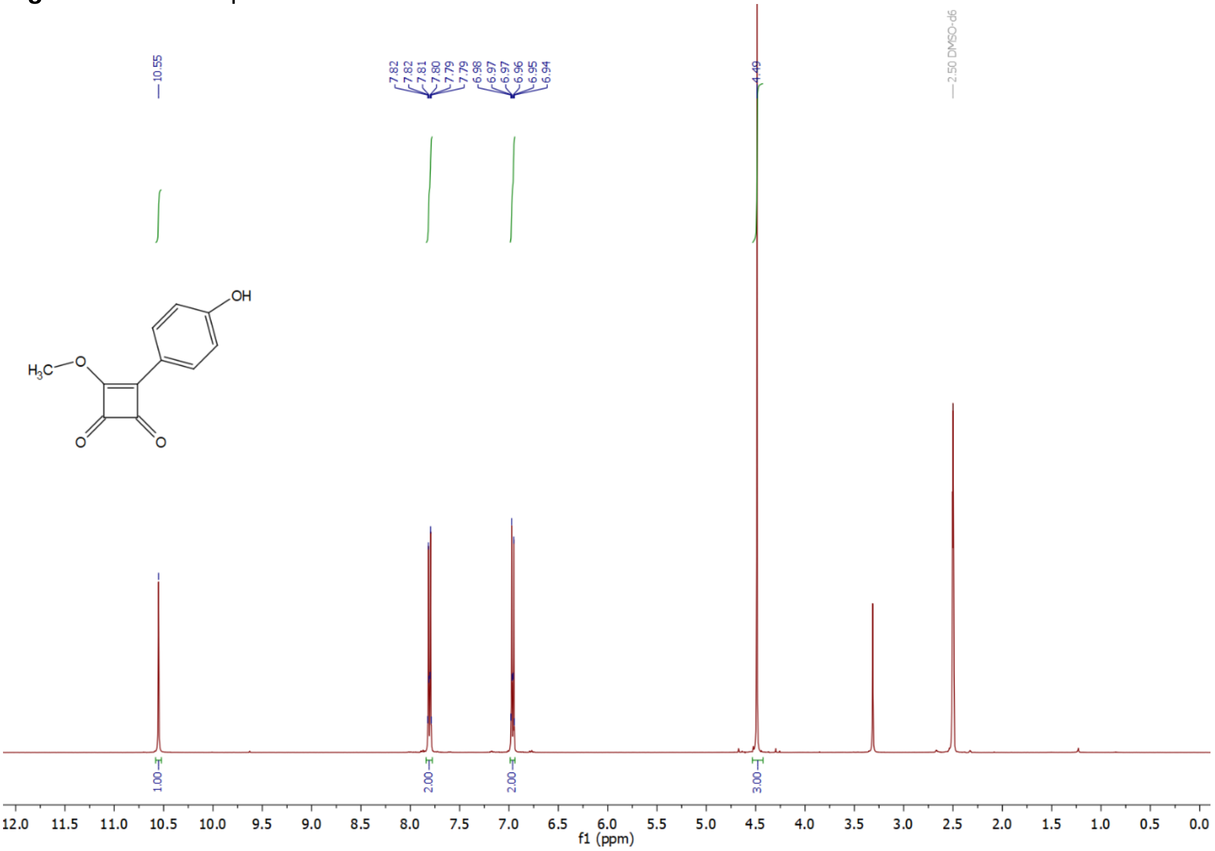


**Fig S13.** $^{13}C$ NMR spectra of **5c**

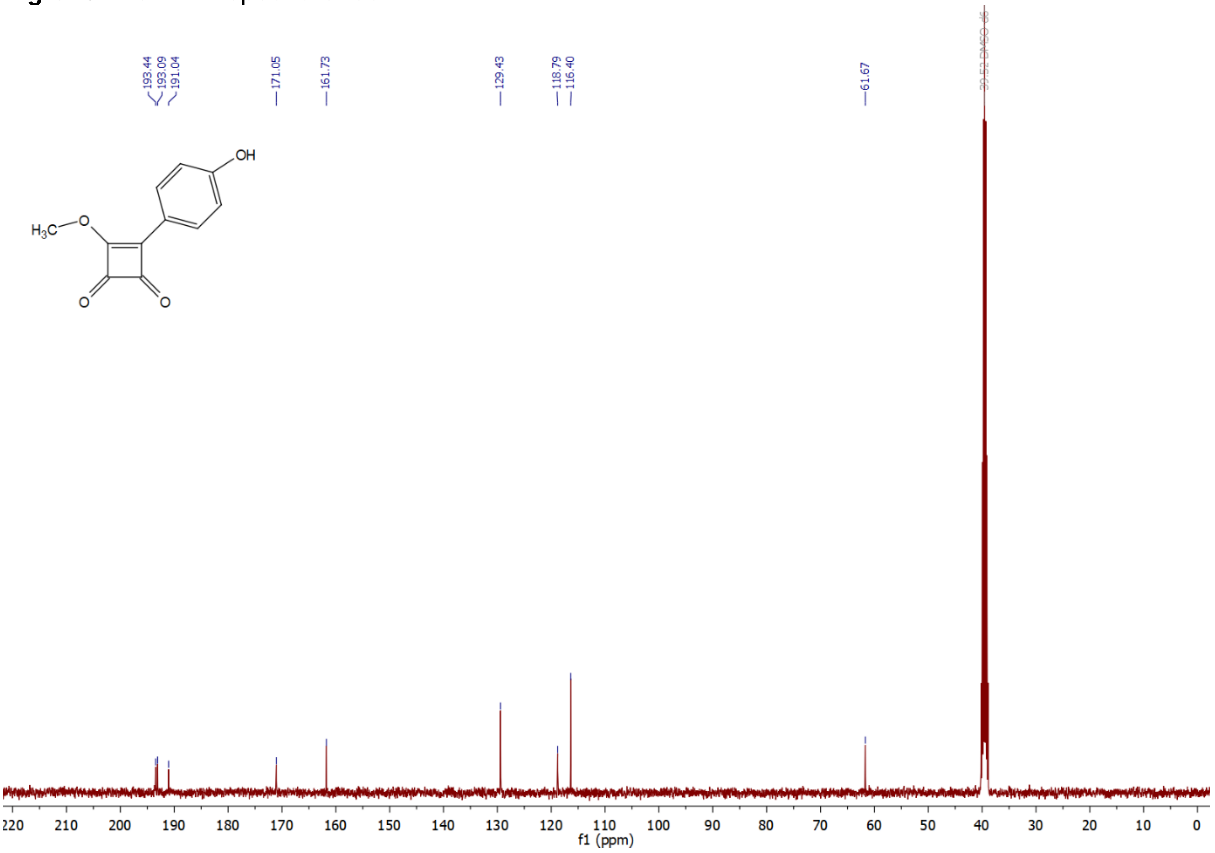

**Fig S14.** $^{1}$H NMR spectra of **6c**

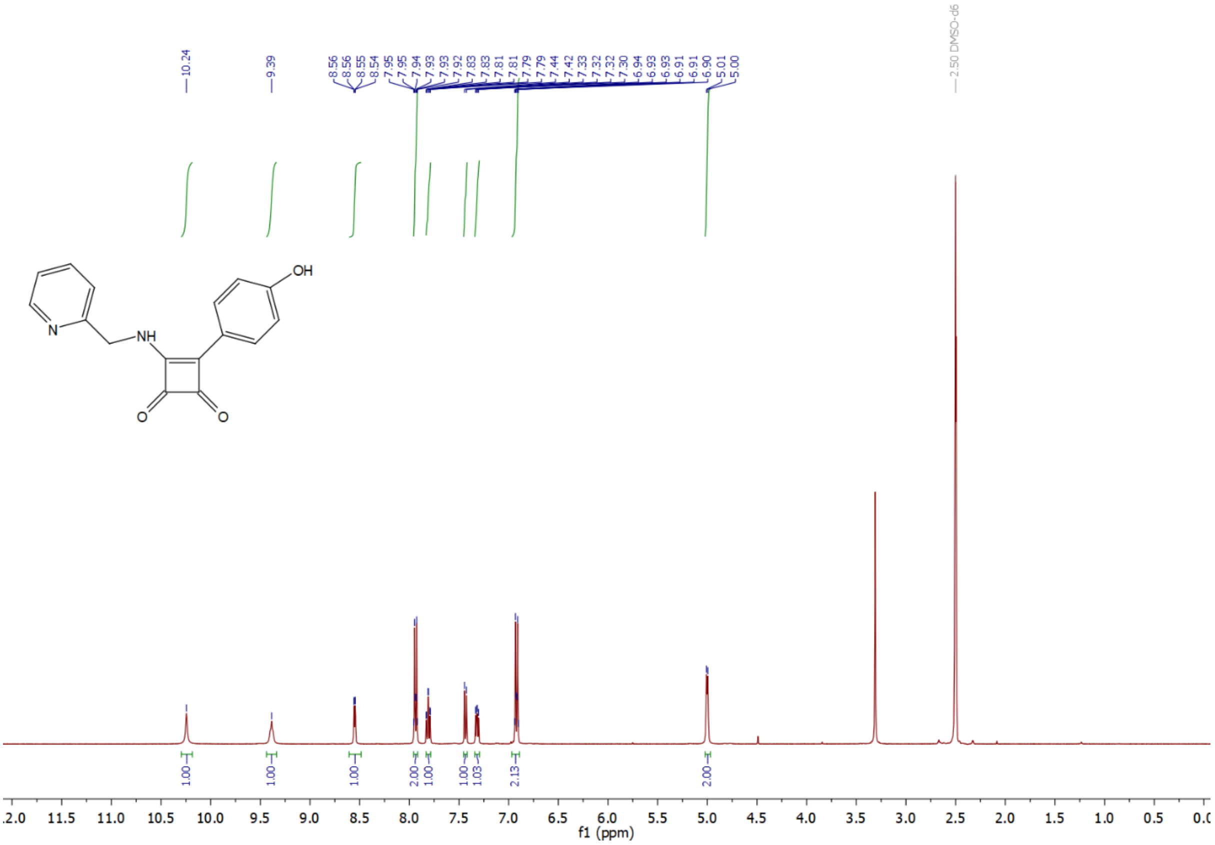

**Fig S15.** $^{13}$C NMR spectra of **6c**

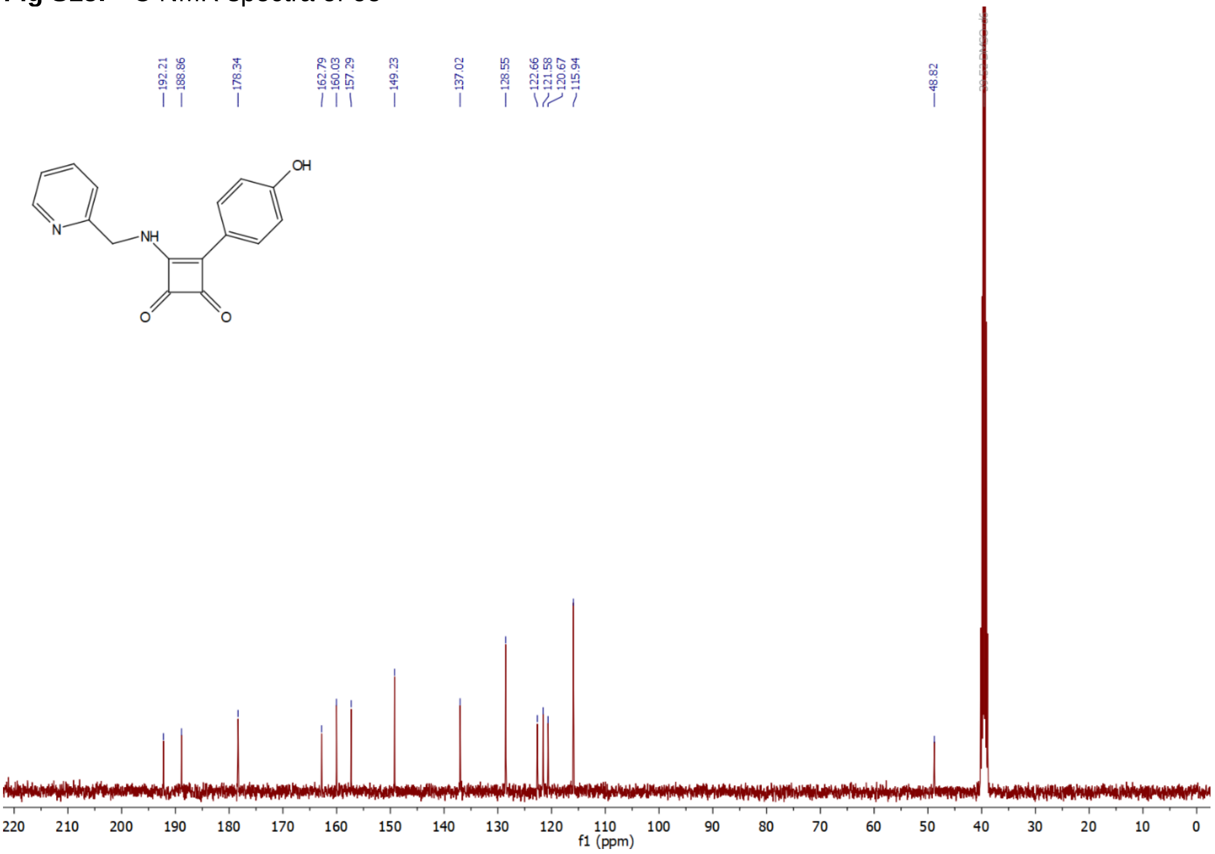

**Fig S16.** $^{1}$H NMR spectra of **5d**

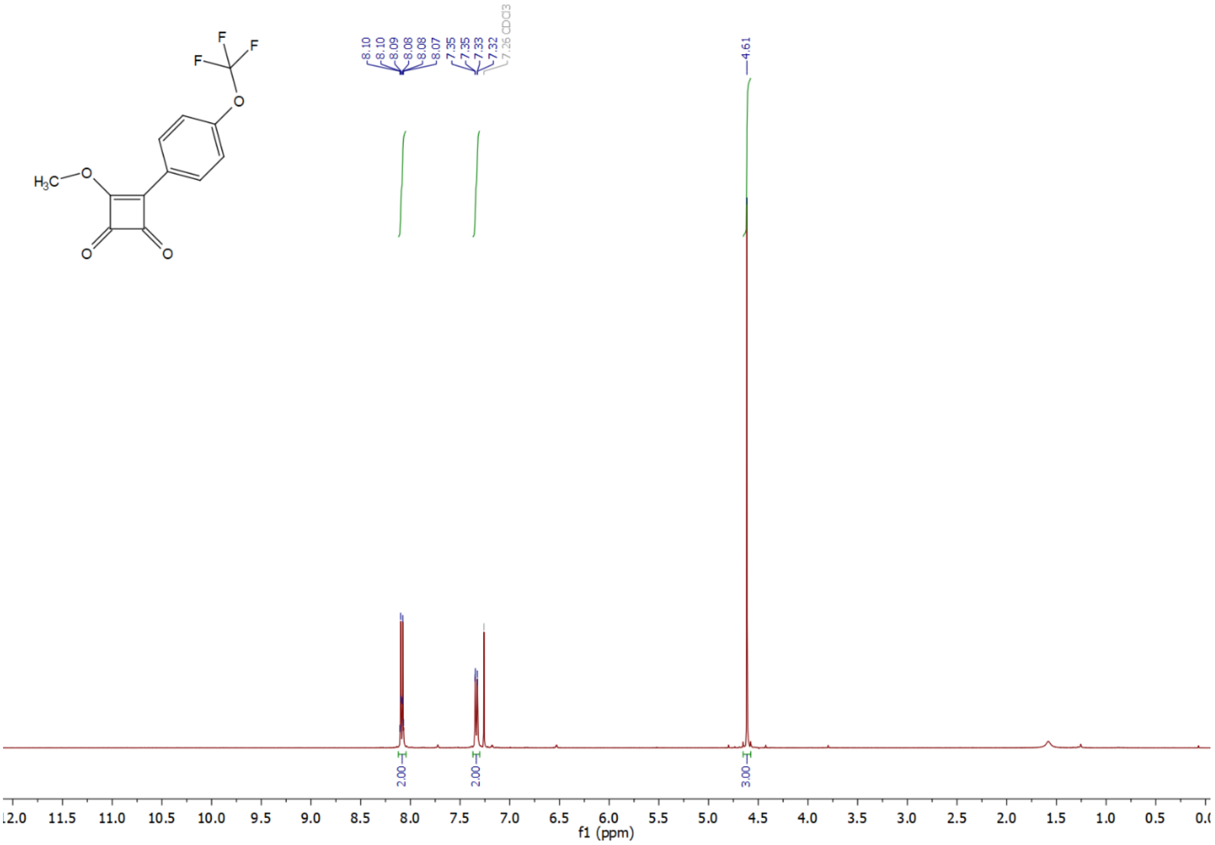


**Fig S17.** $^{13}$C NMR spectra of **5d**

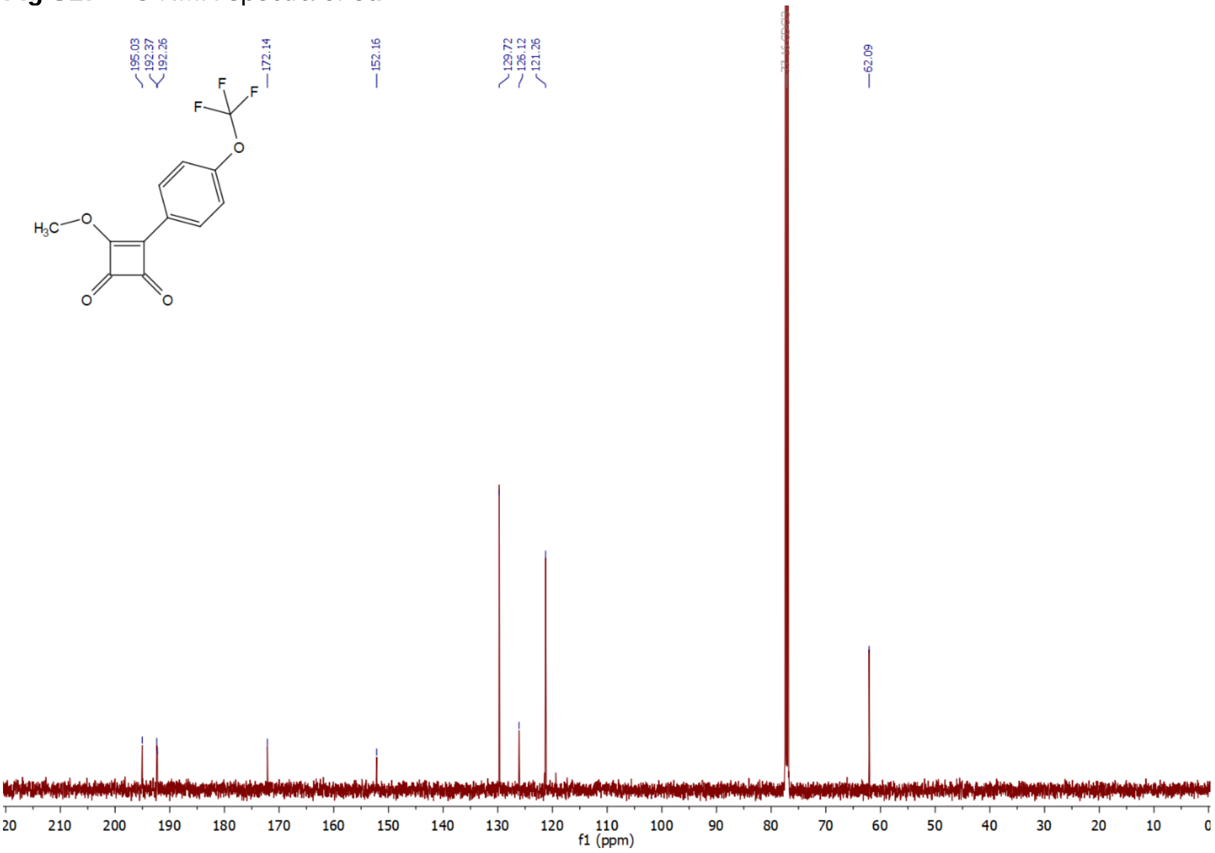

**Fig S18.** $^{1}$H NMR spectra of **6d**

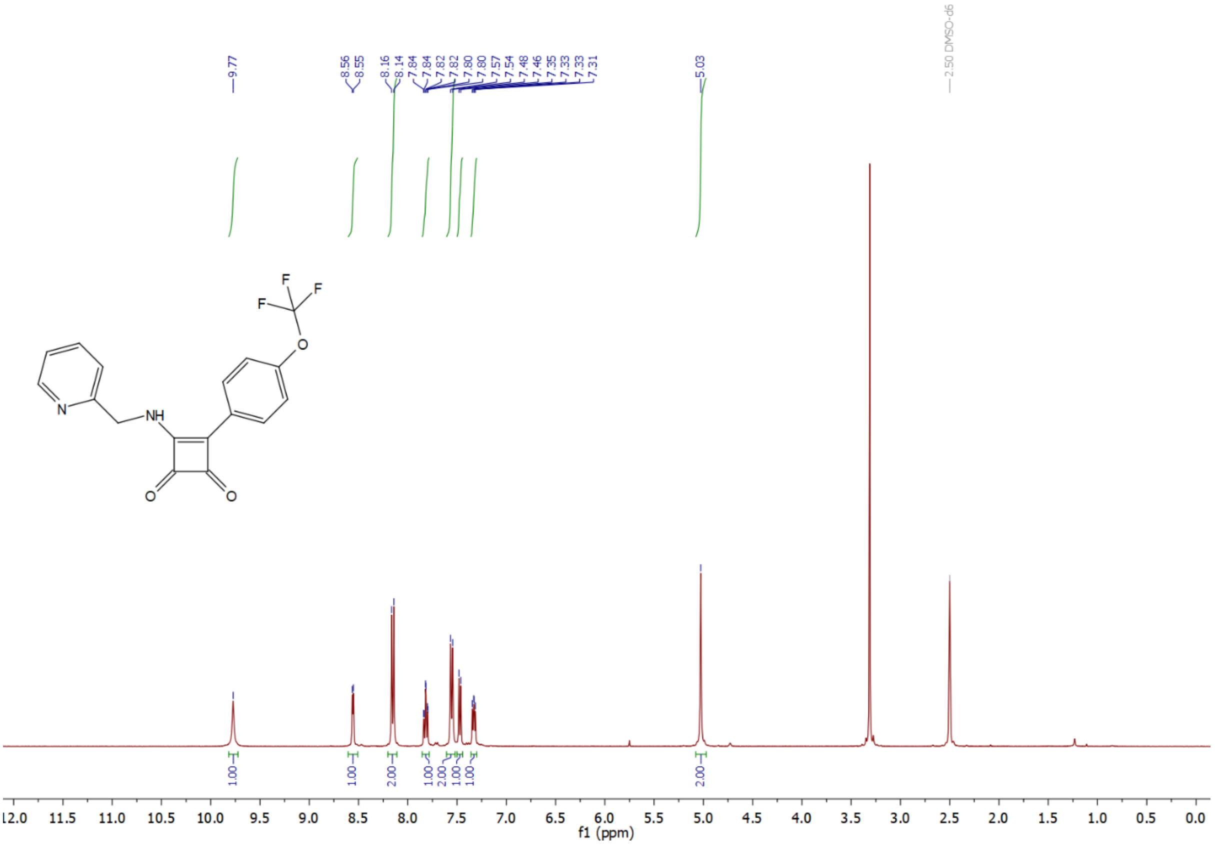


**Fig S19.** $^{13}$C NMR spectra of **6d**

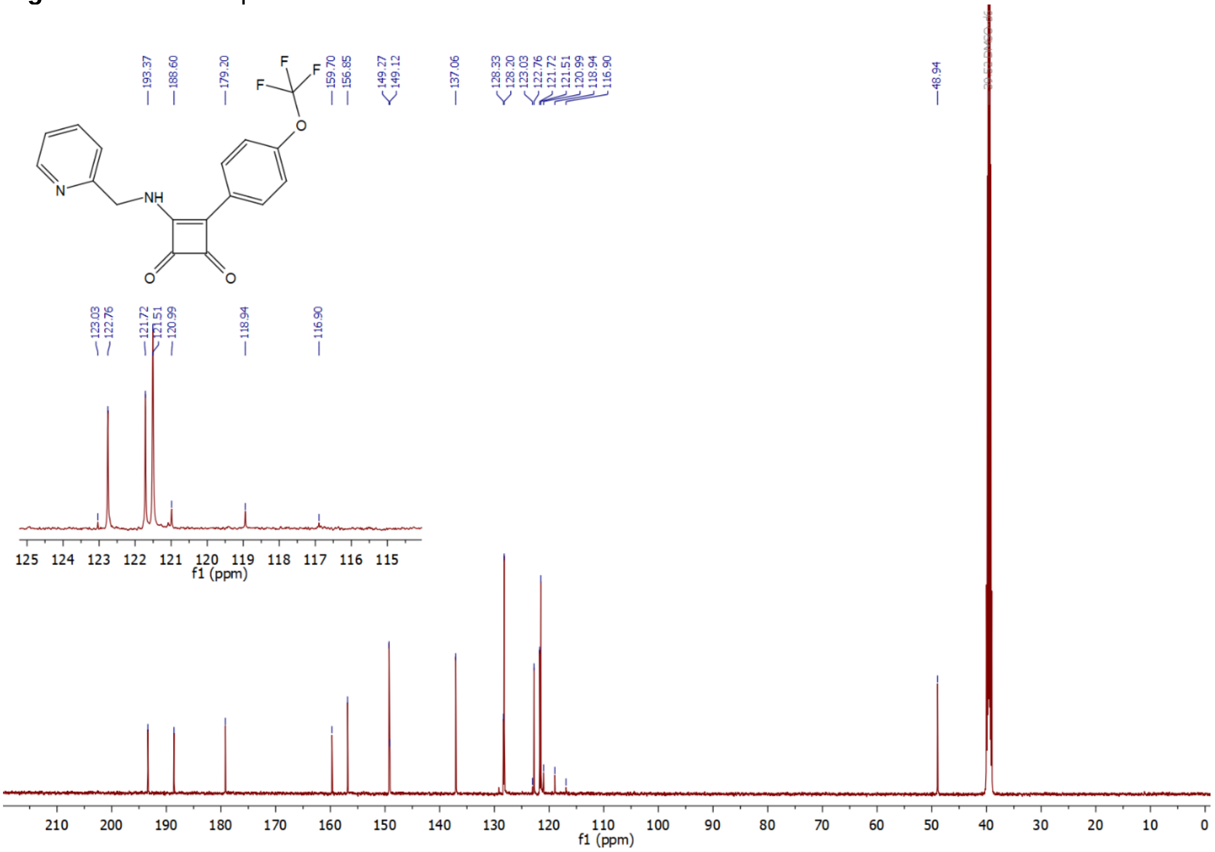

**Fig S20.** $^{1}$H NMR spectra of **5e**

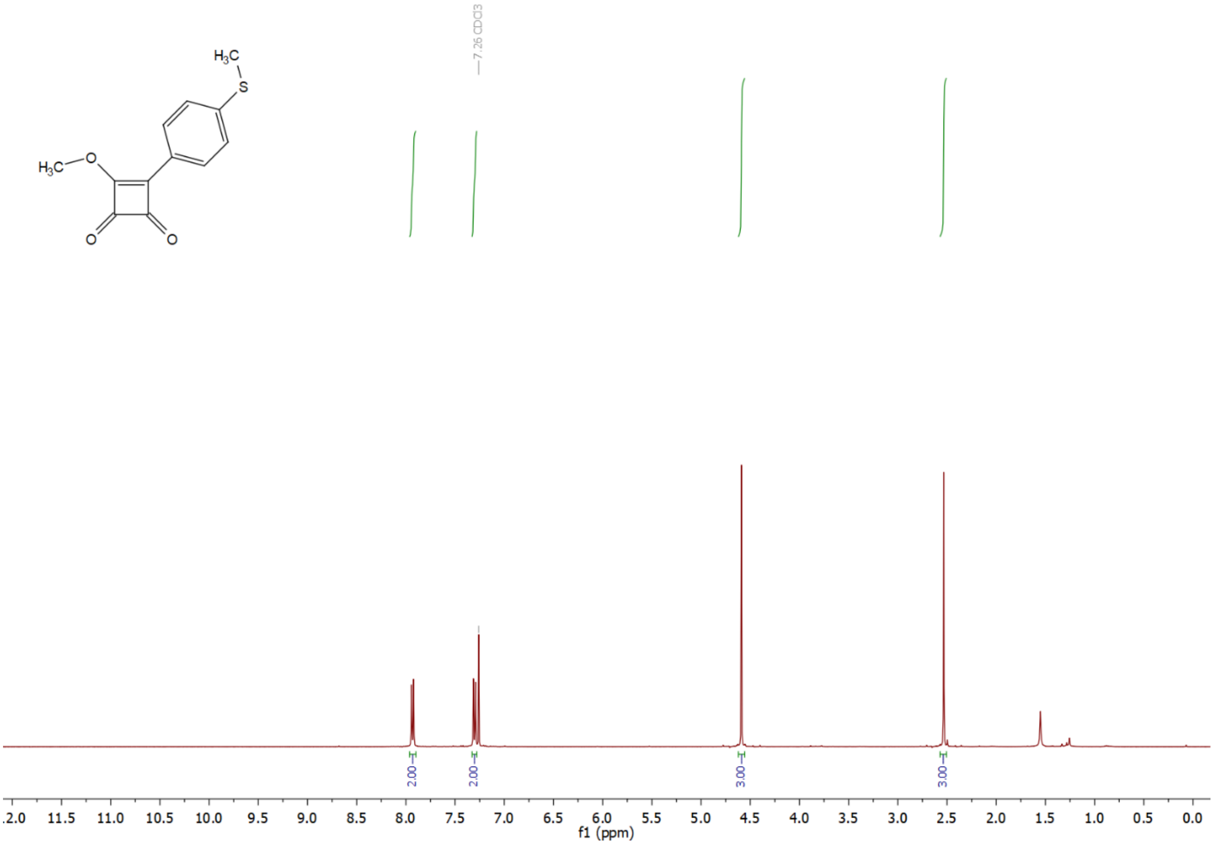


**Fig S21.** $^{13}$C NMR spectra of **5e**

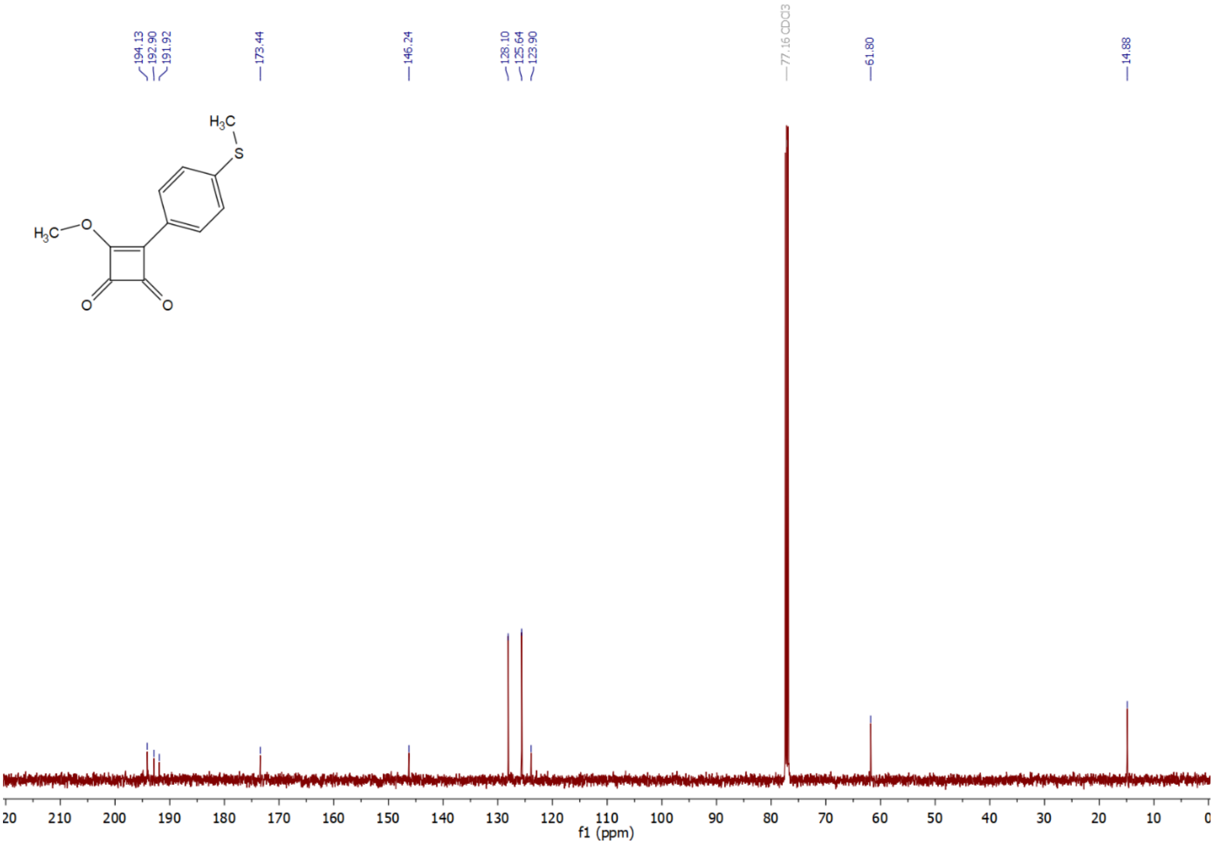

**Fig S22.** $^{1}$H NMR spectra of **6e**

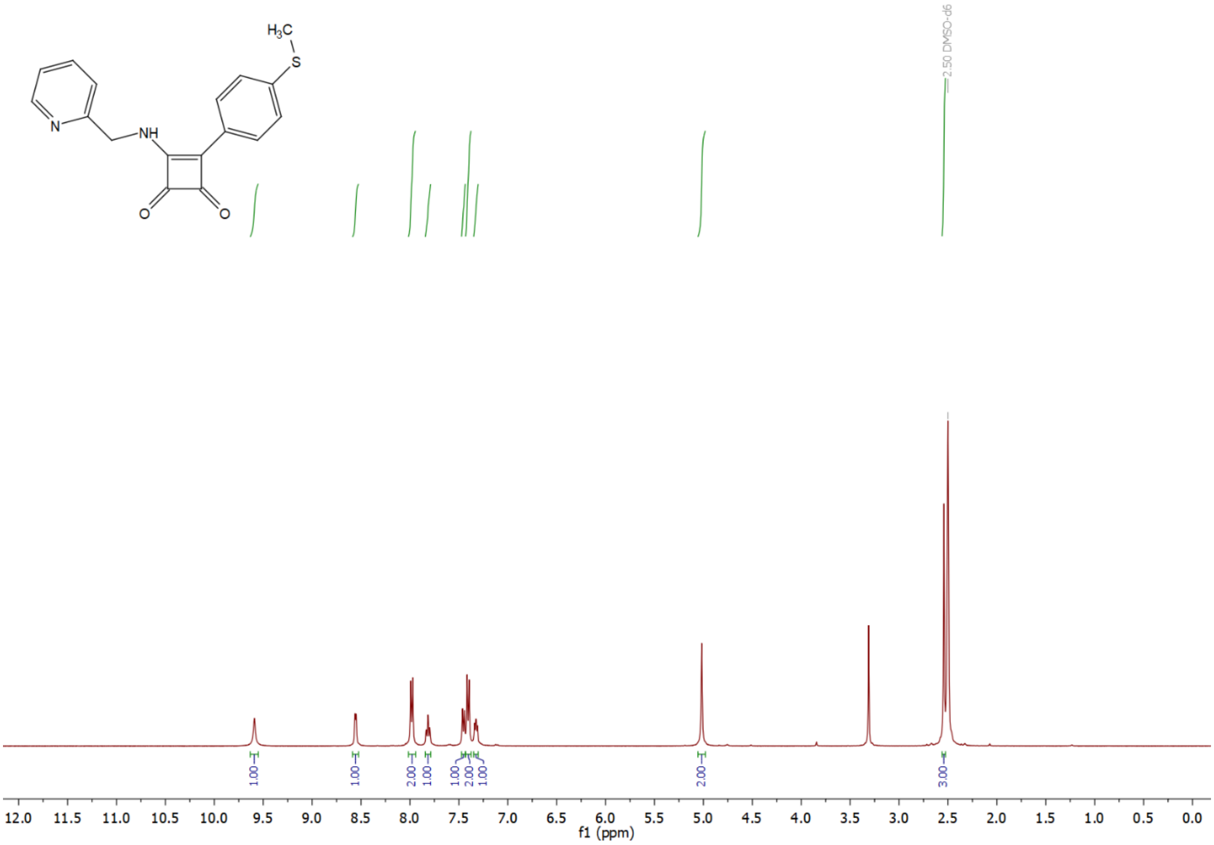


**Fig S23.** $^{13}$C NMR spectra of **6e**

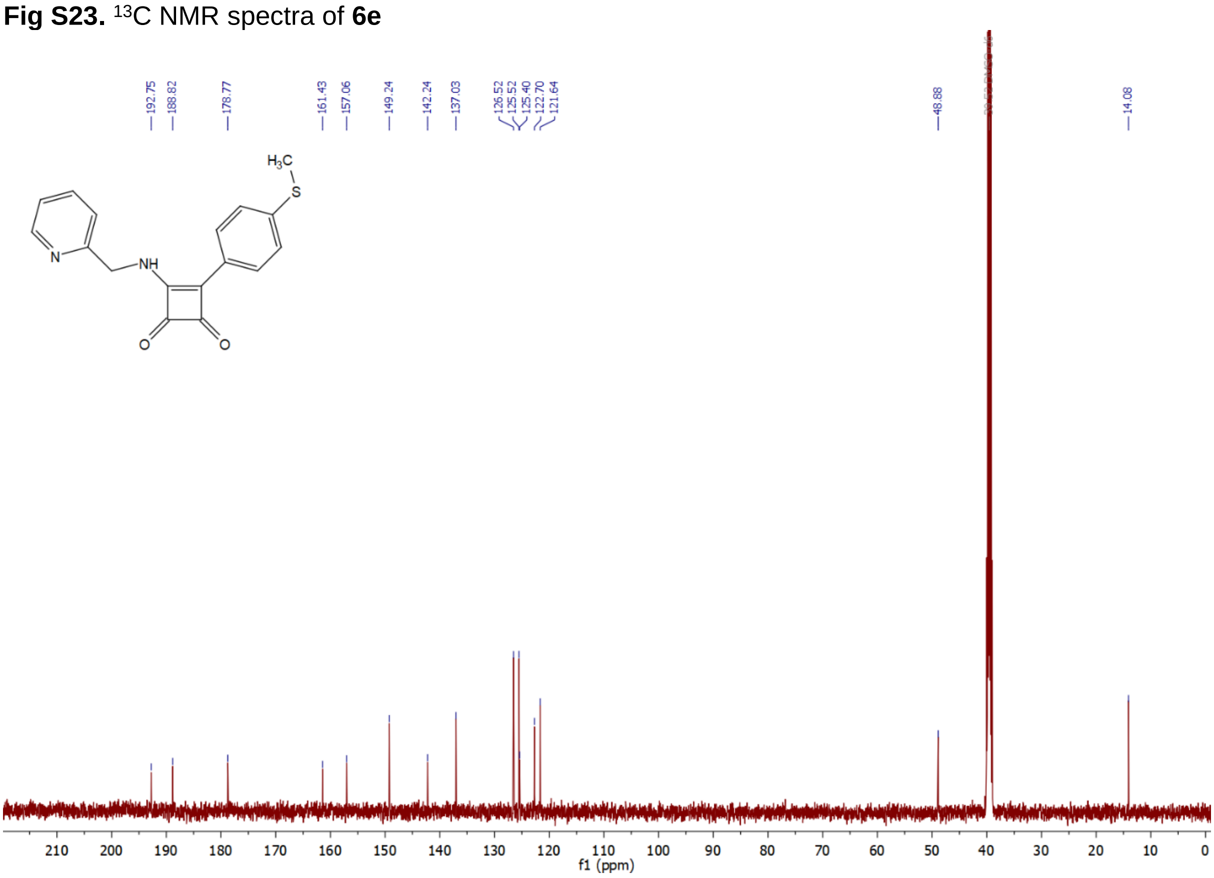

**Fig S24.** $^{1}$H NMR spectra of **5f**

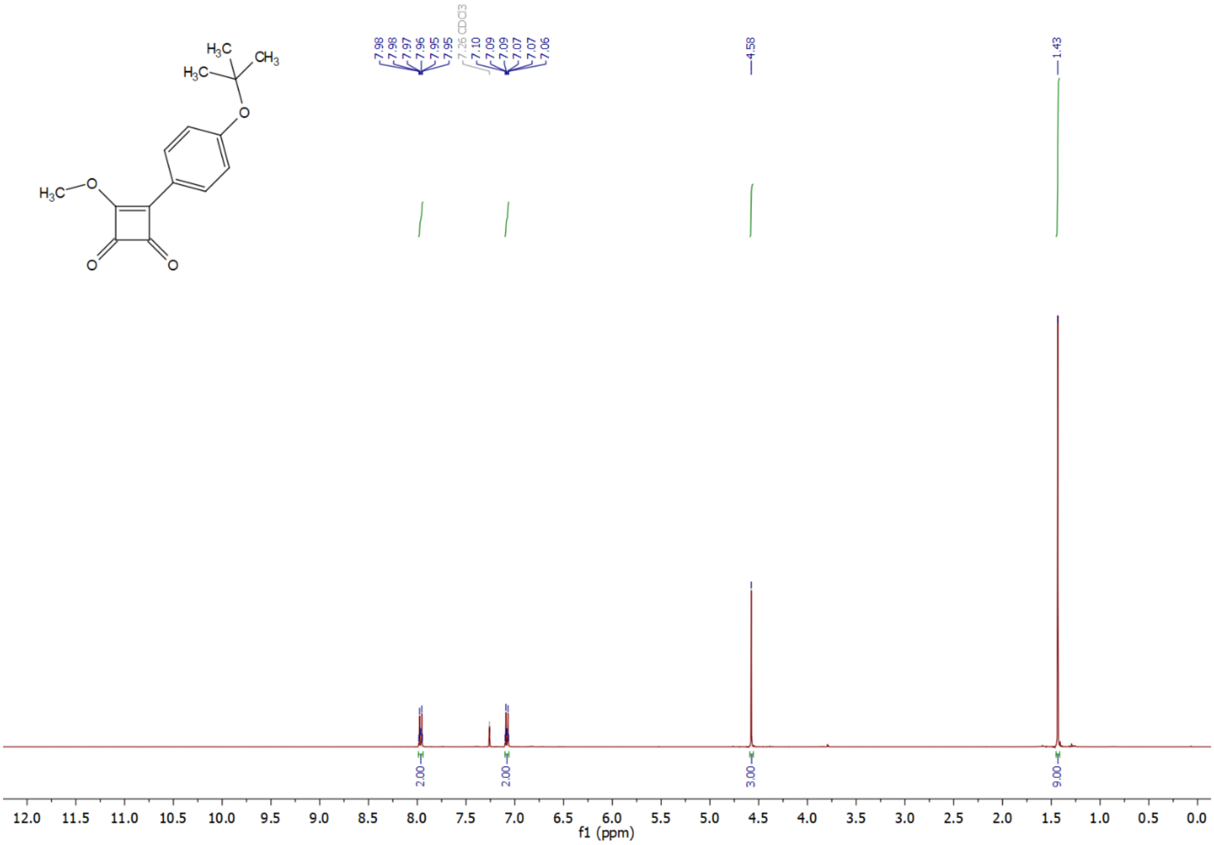


**Fig S25.** $^{13}$C NMR spectra of **5f**

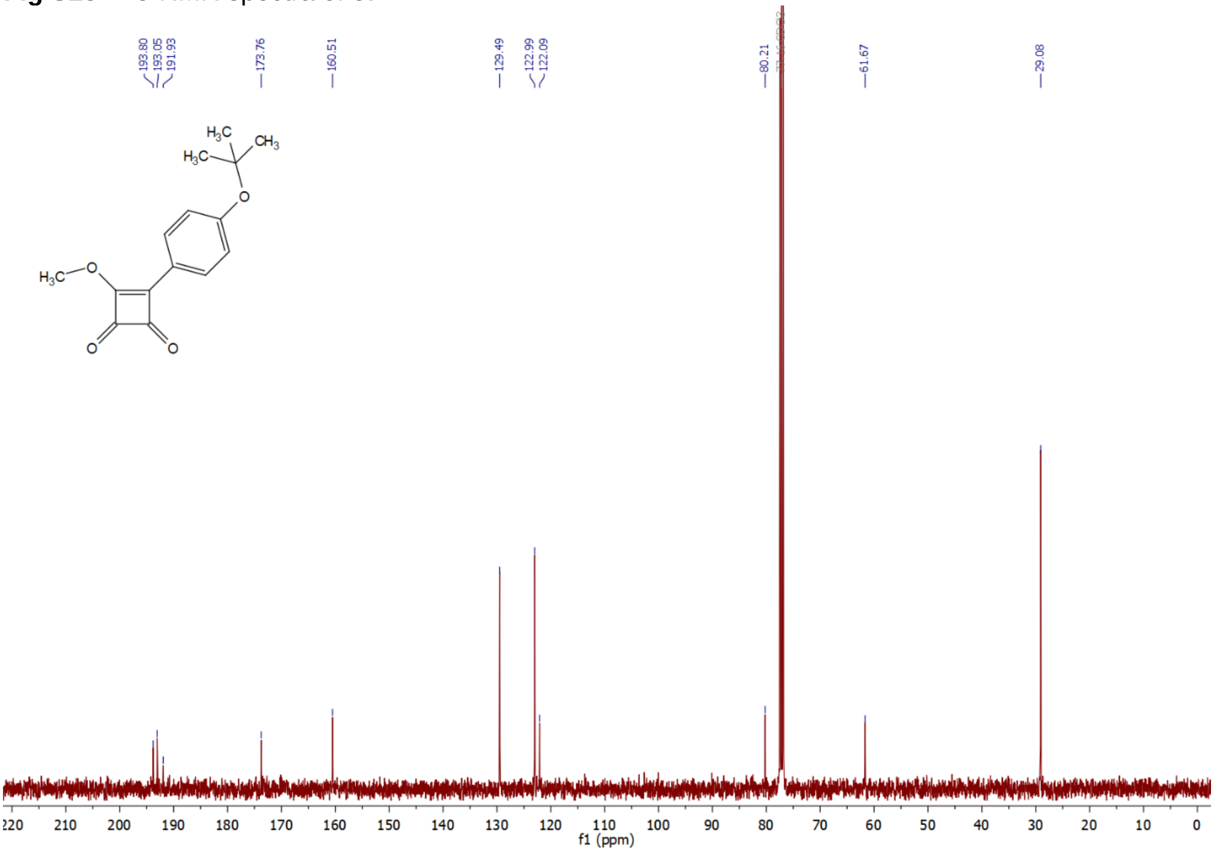

**Fig S26.** $^{1}$H NMR spectra of **6f**

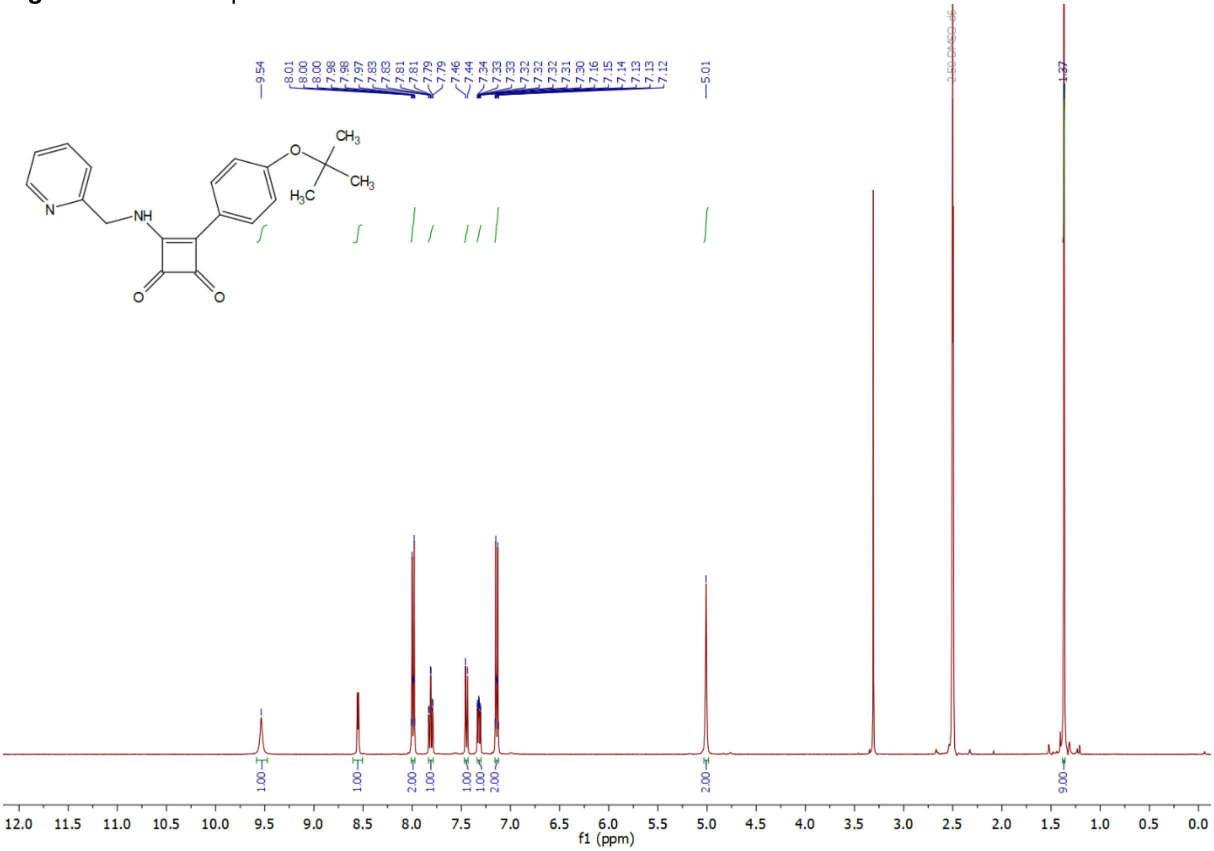

**Fig S27.** $^{13}$C NMR spectra of **6f**

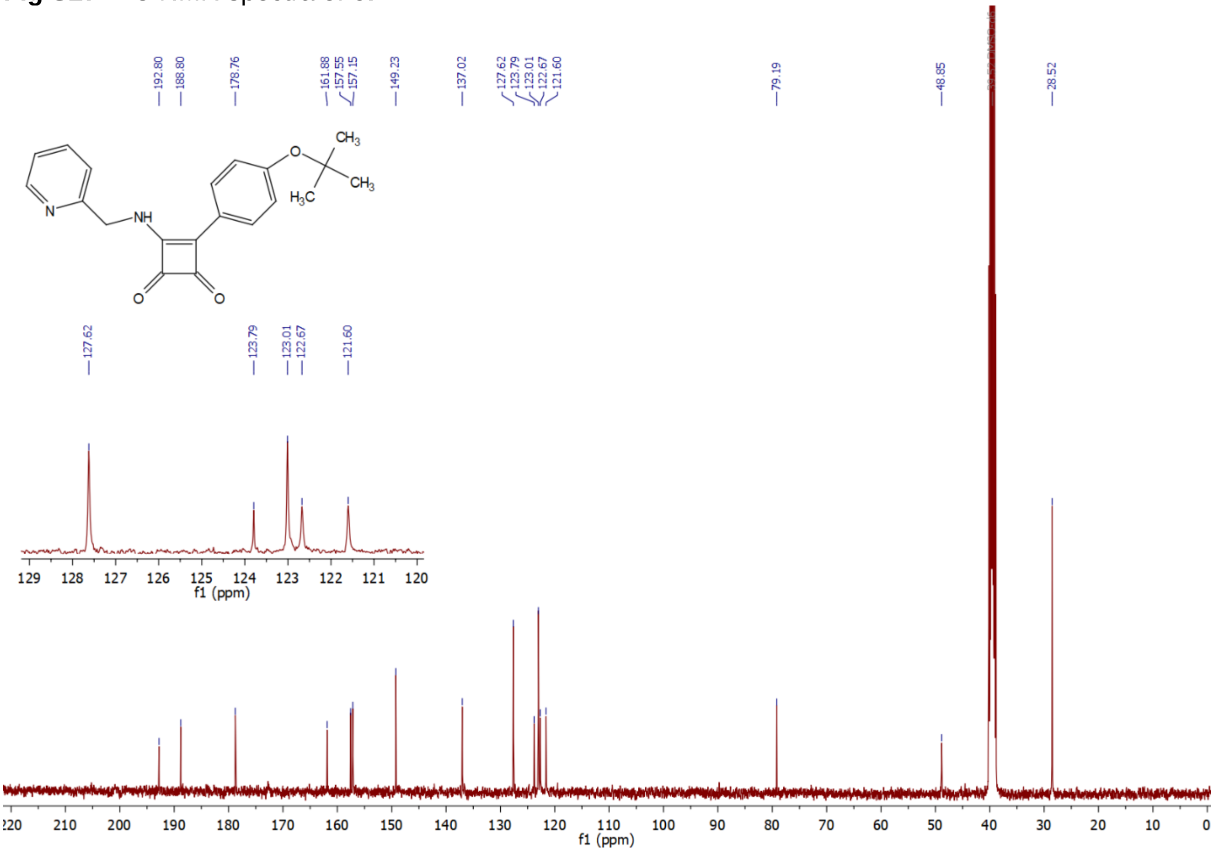

**Fig S28.** $^{1}H$ NMR spectra of **5g**

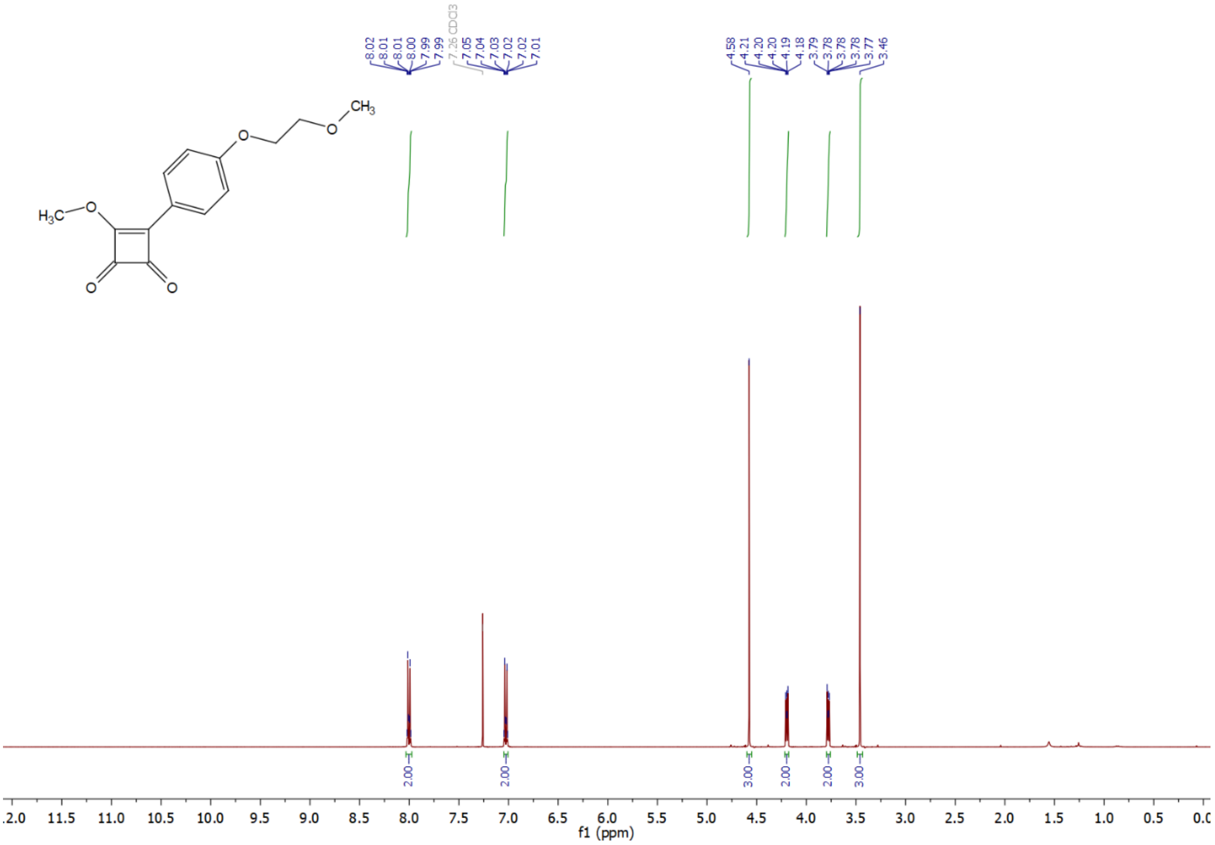


**Fig S29.** $^{13}C$ NMR spectra of **5g**

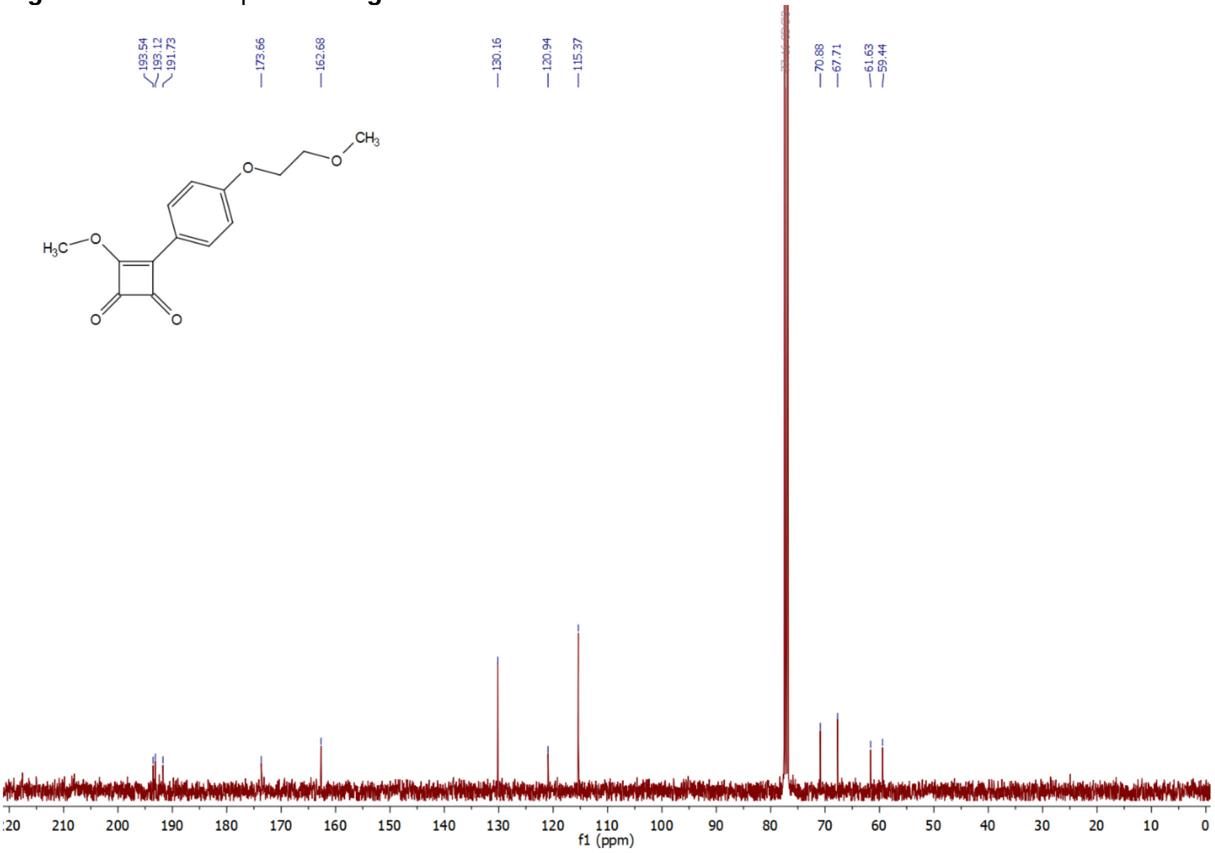

**Fig S30.** $^{1}H$ NMR spectra of **6g**

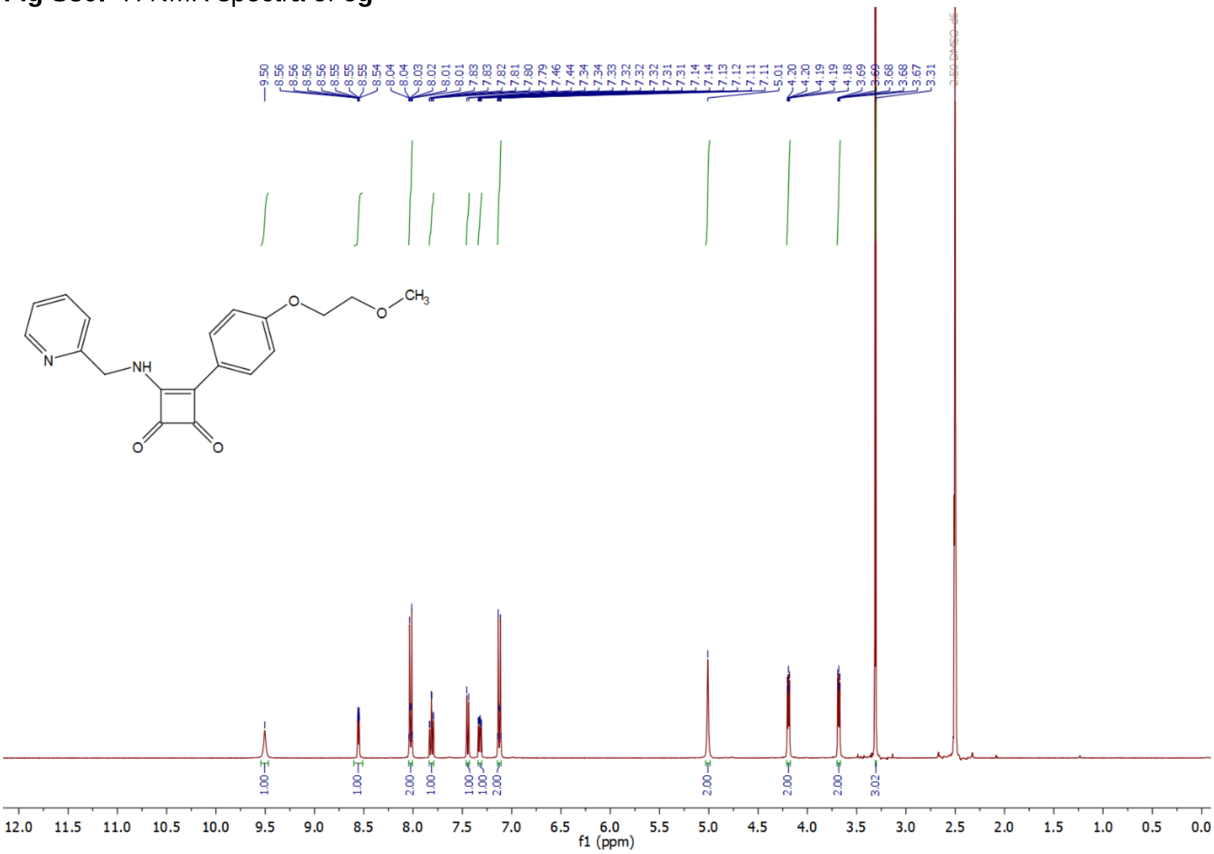


**Fig S31.** $^{13}C$ NMR spectra of **6g**

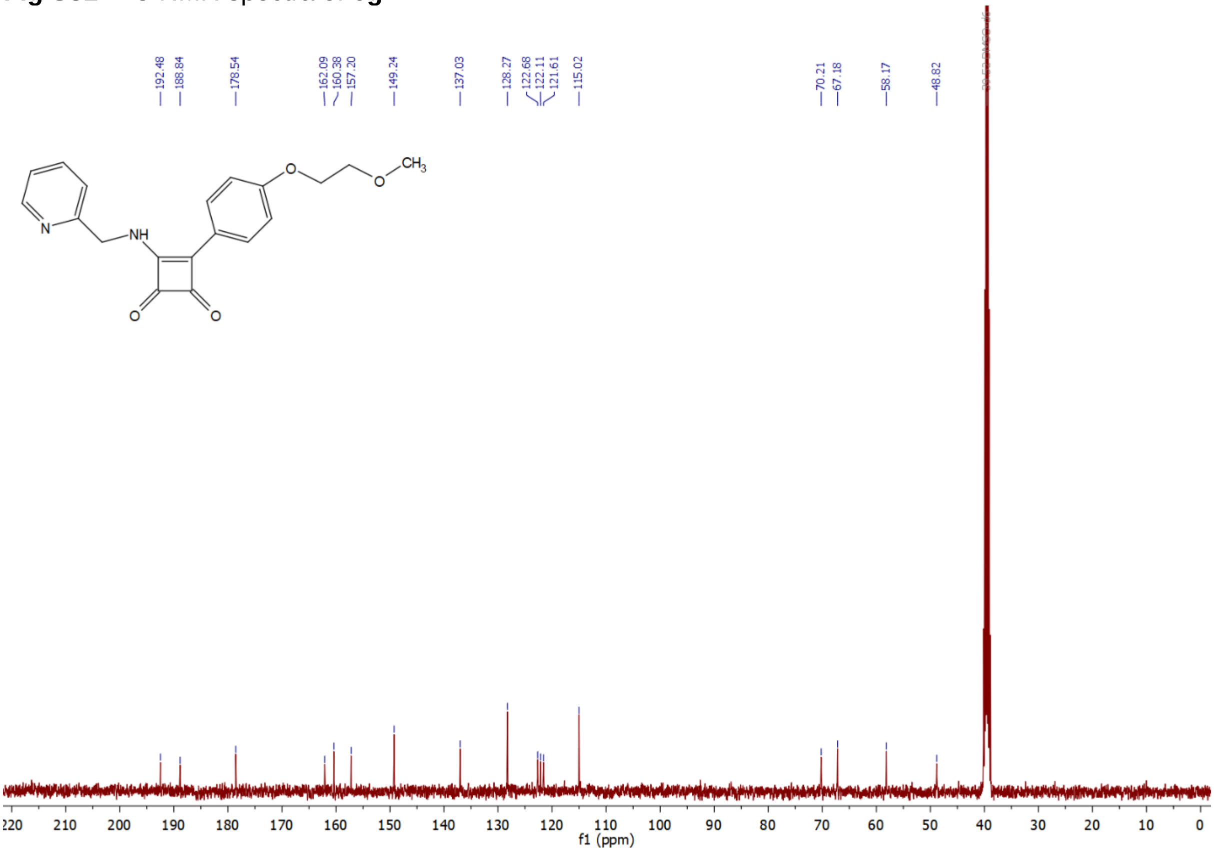

**Fig S32.** $^{1}$H NMR spectra of **5h**

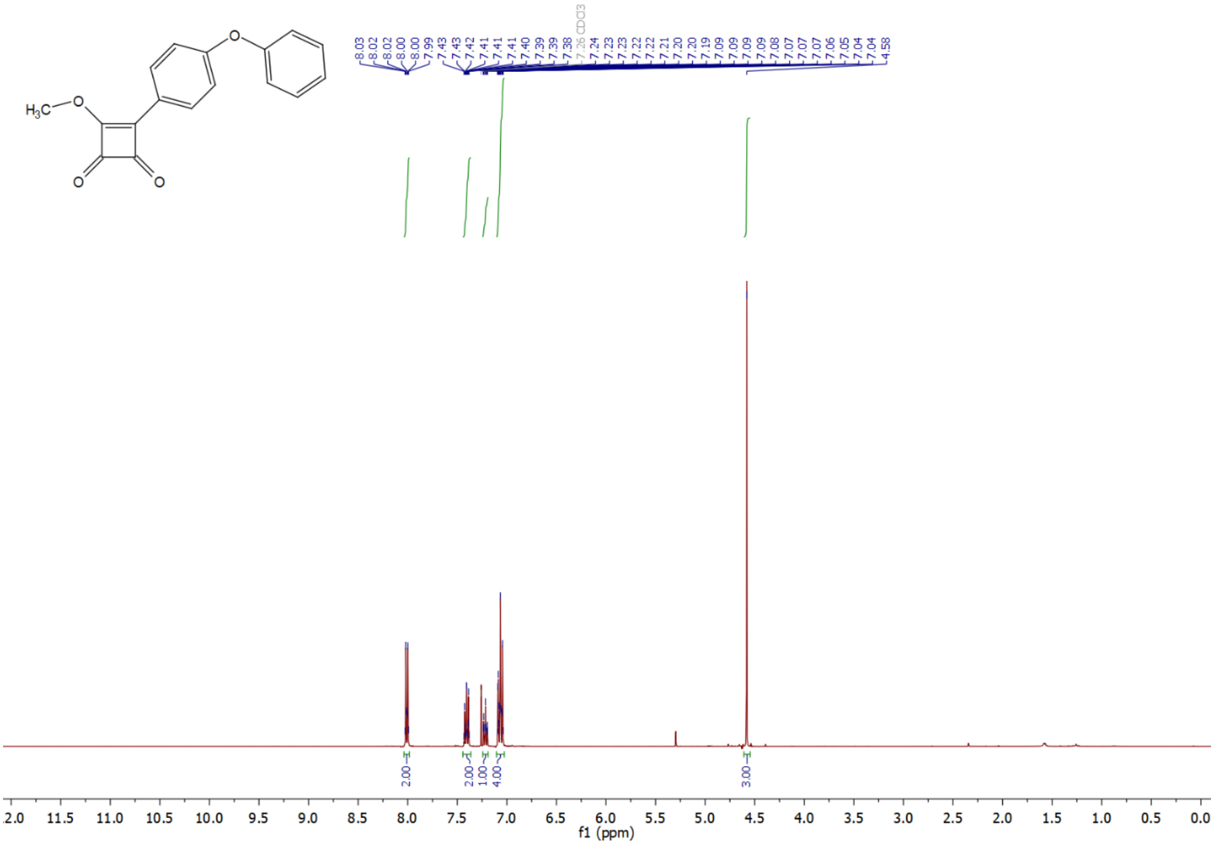


**Fig S33.** $^{13}$C NMR spectra of **5h**

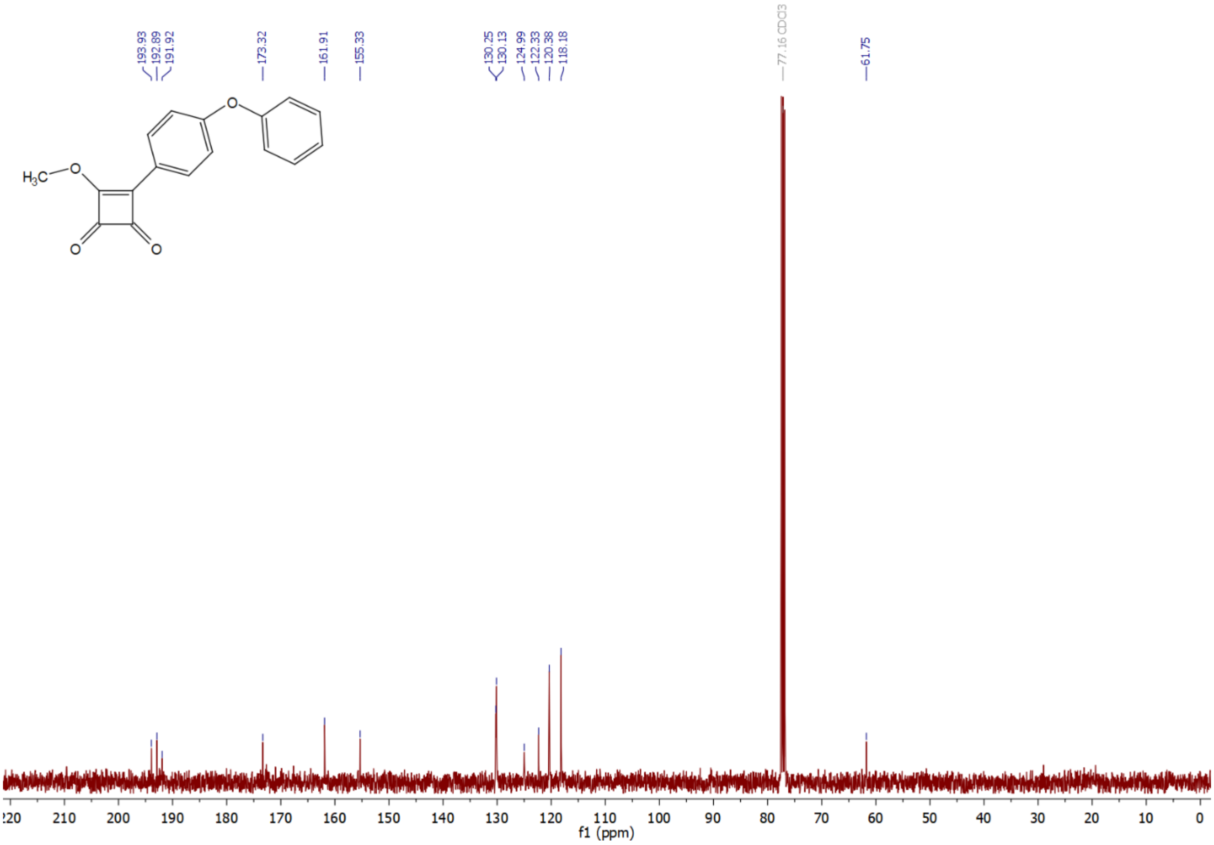

**Fig S34.** $^{1}H$ NMR spectra of **6h**

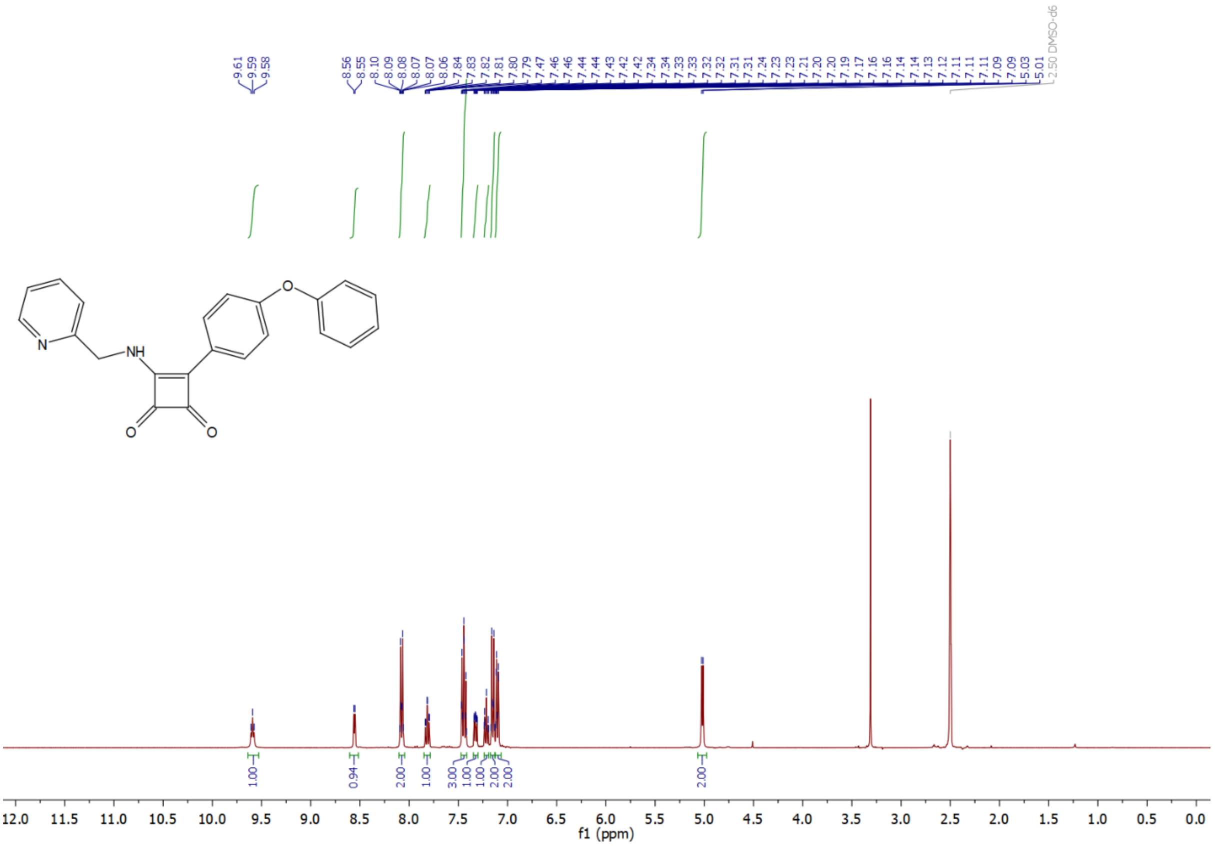


**Fig S35.** $^{13}C$ NMR spectra of **6h**

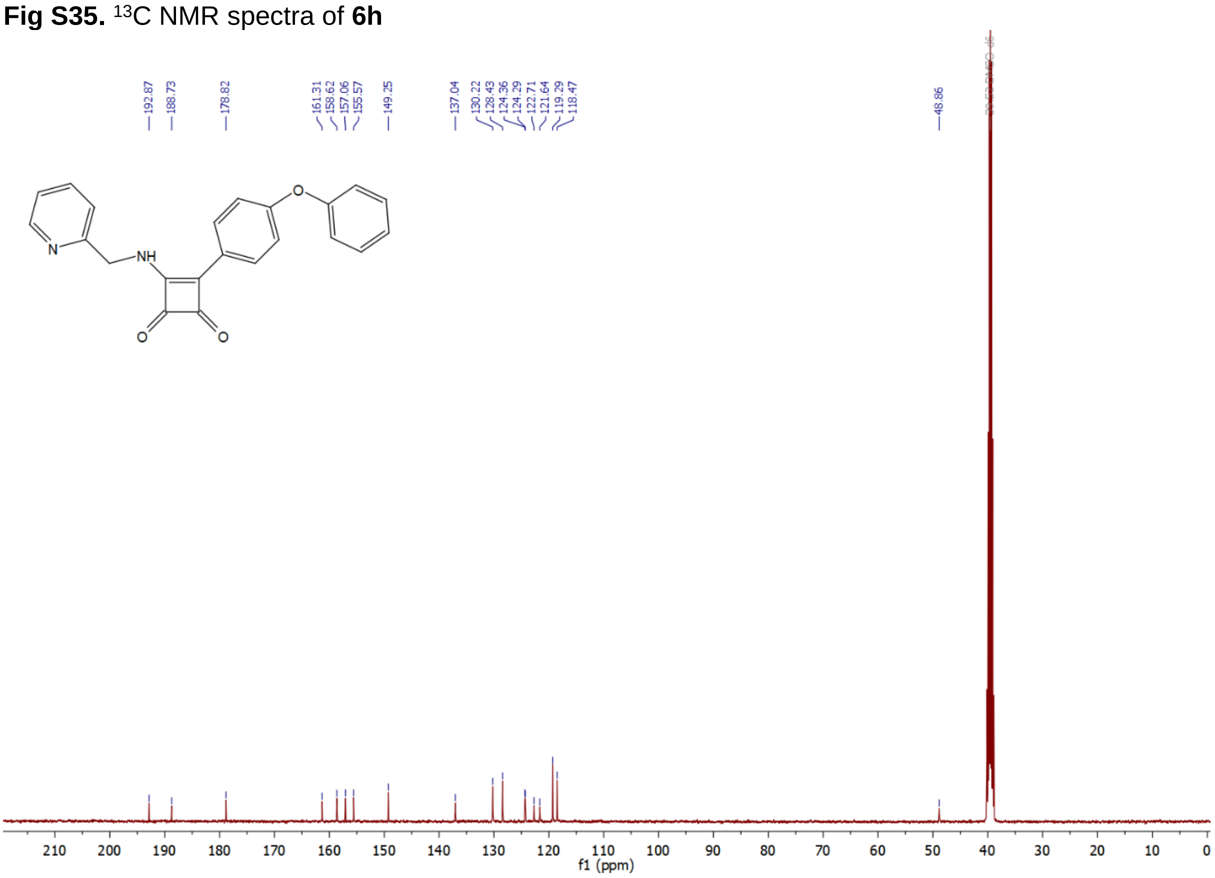

**Fig S36.** $^{1}$H NMR spectra of **5i**

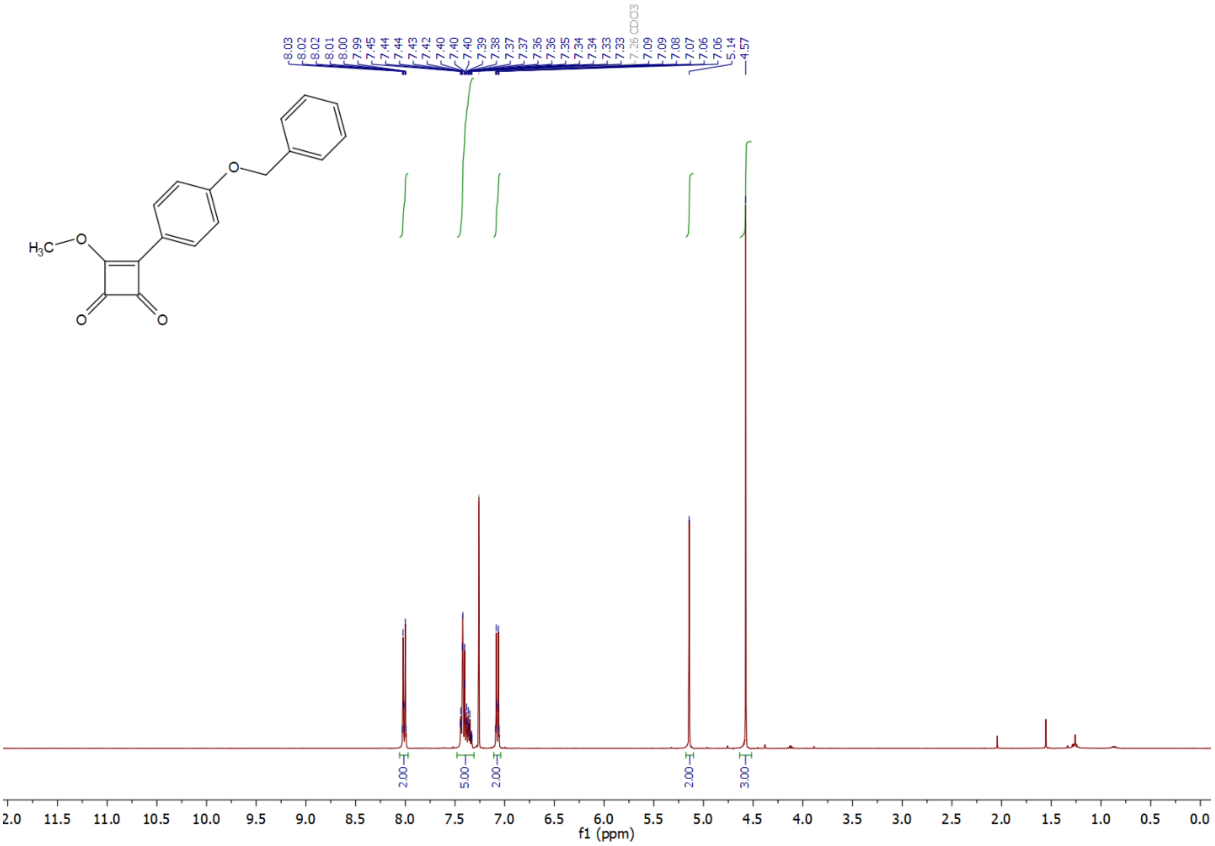


**Fig S37.** $^{13}$C NMR spectra of **5i**

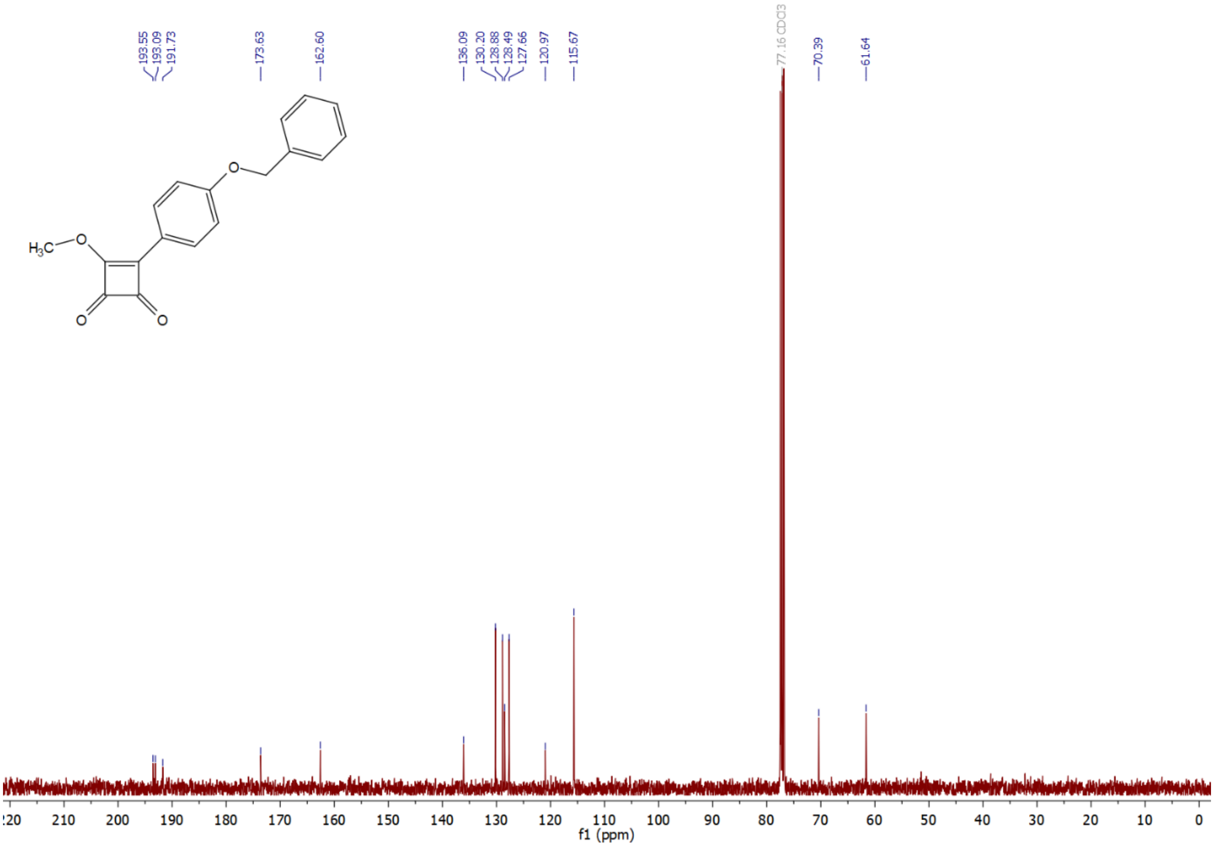

**Fig S38.** $^{1}H$ NMR spectra of **6i**

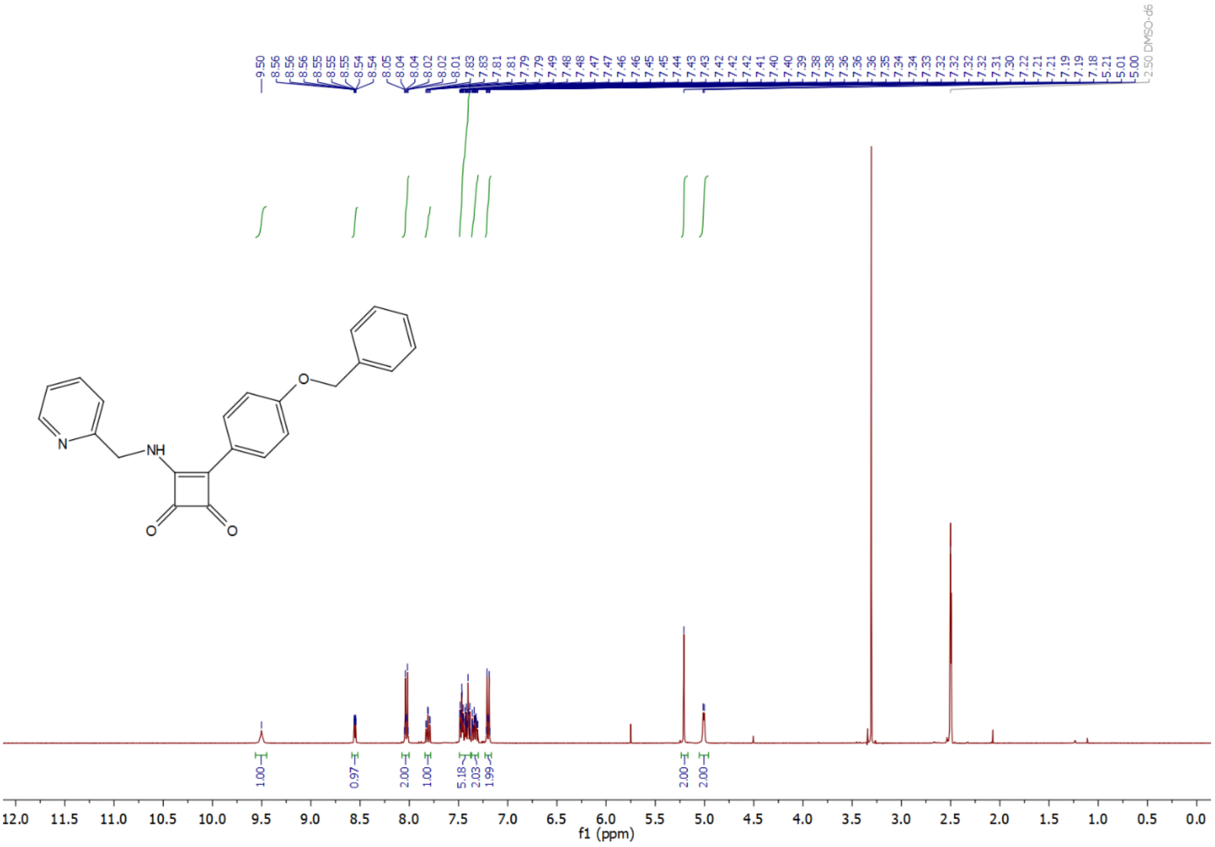


**Fig S39.** $^{13}C$ NMR spectra of **6i**

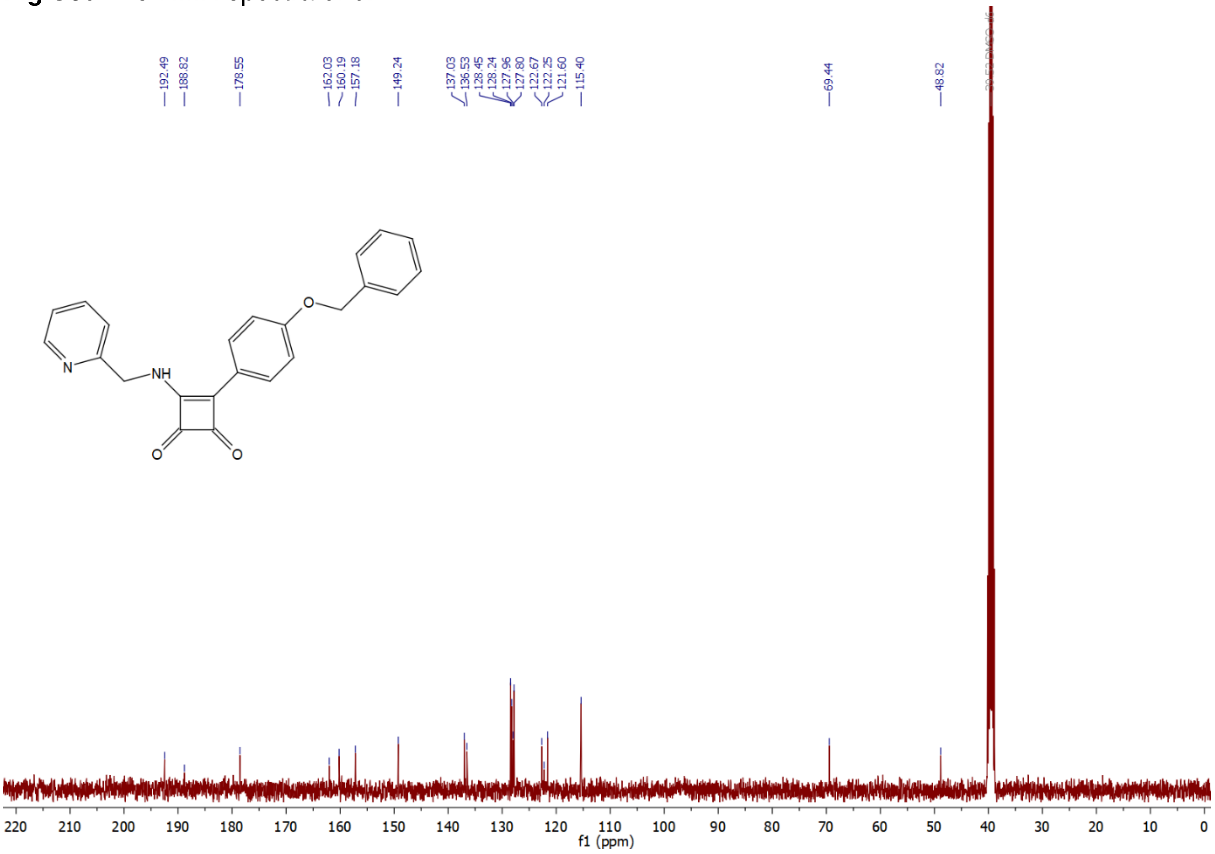

**Fig S40.** $^{1}$H NMR spectra of **5j**

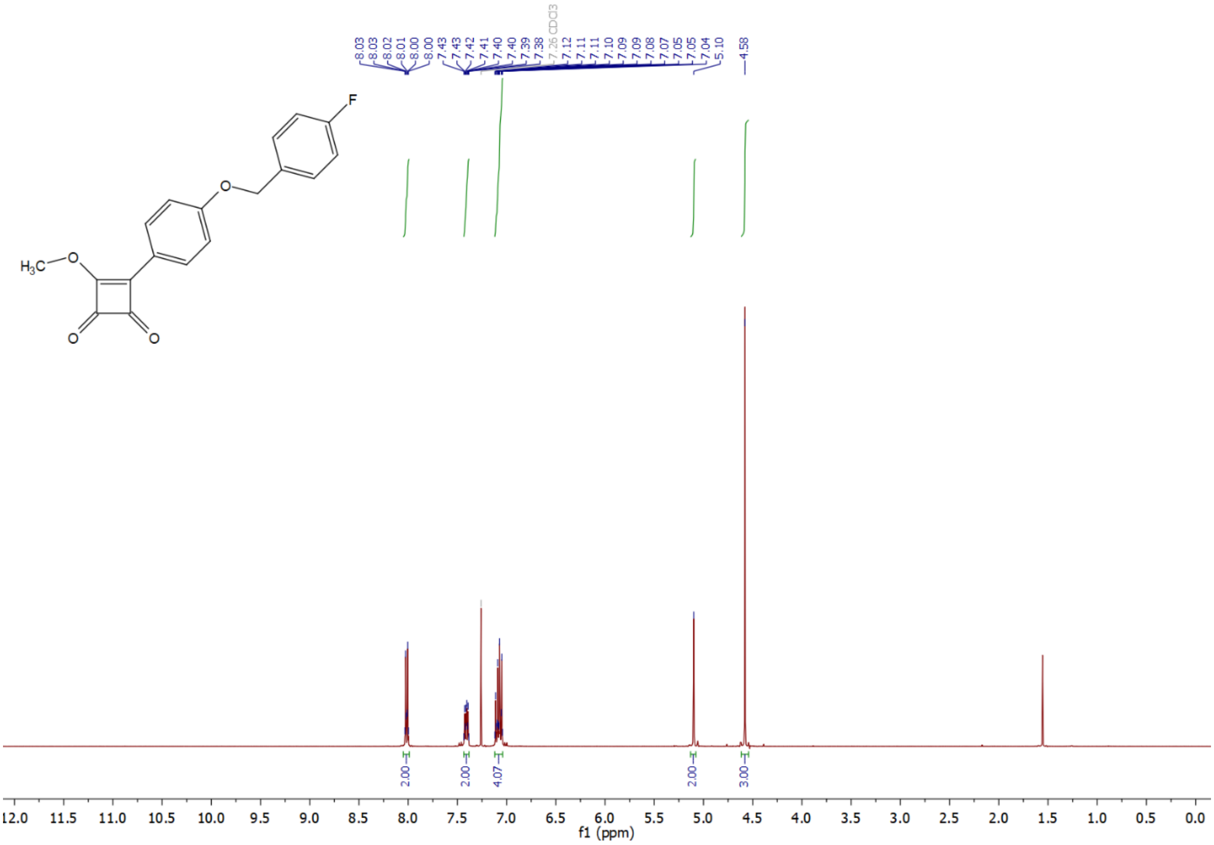

**Fig S41.** $^{13}$C NMR spectra of **5j**

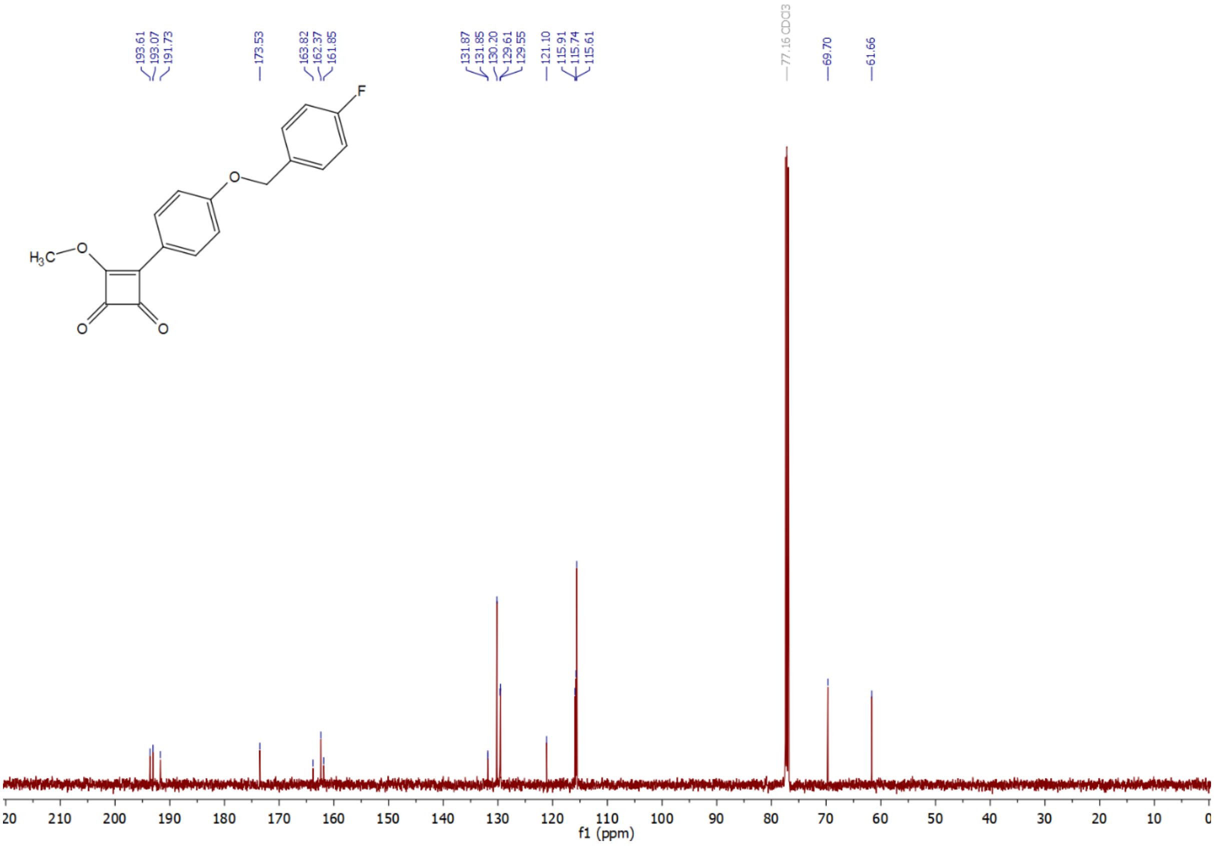

**Fig S42.** $^{1}$H NMR spectra of **6j**

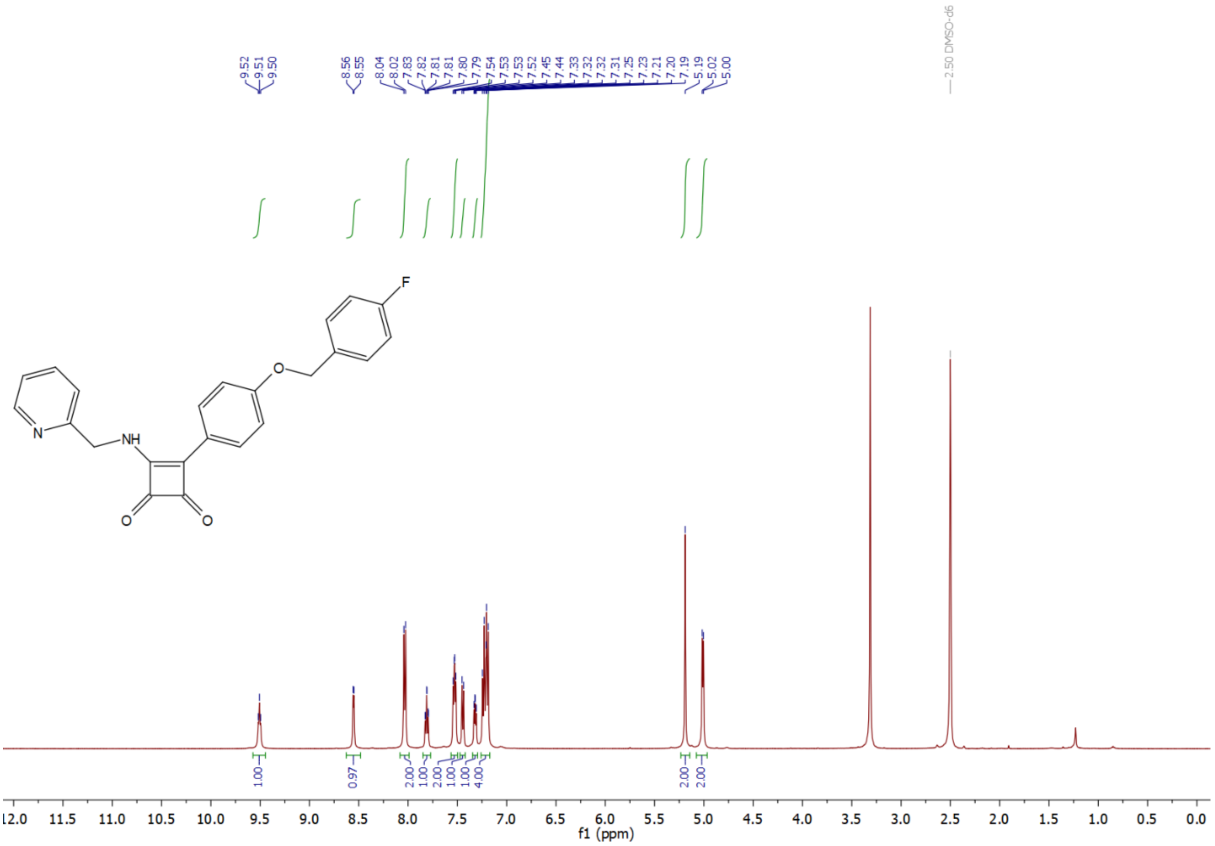

**Fig S43.** $^{13}$C NMR spectra of **6j**

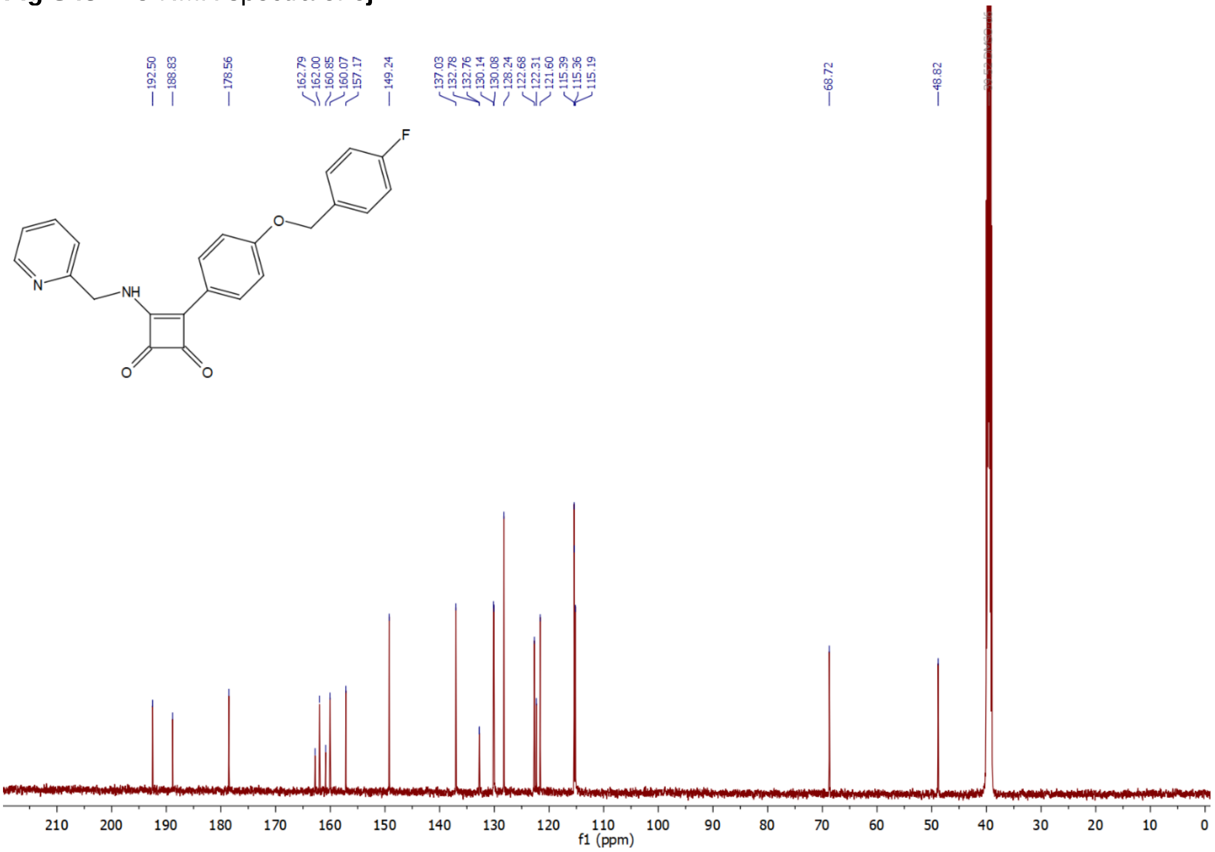

**Fig S44.** $^{1}$H NMR spectra of **5k**

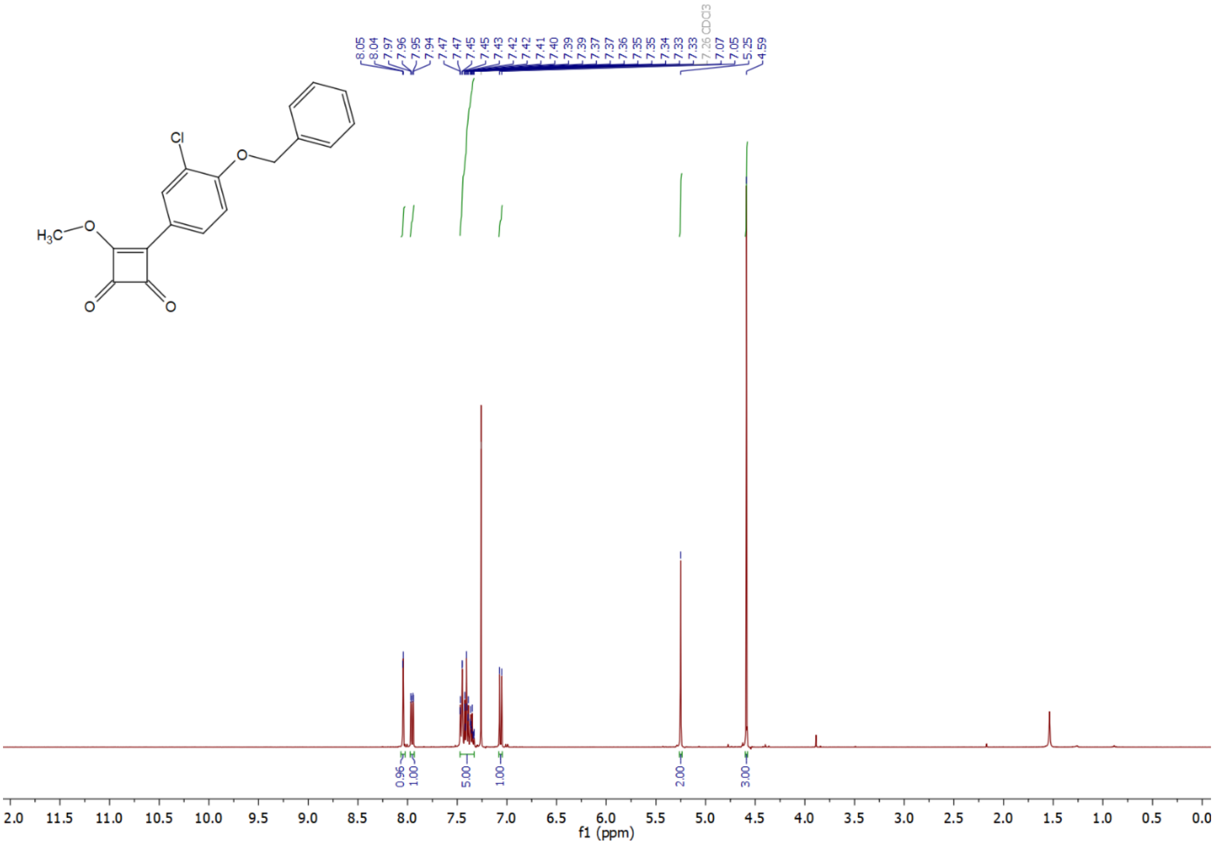


**Fig S45.** $^{13}$C NMR spectra of **5k**

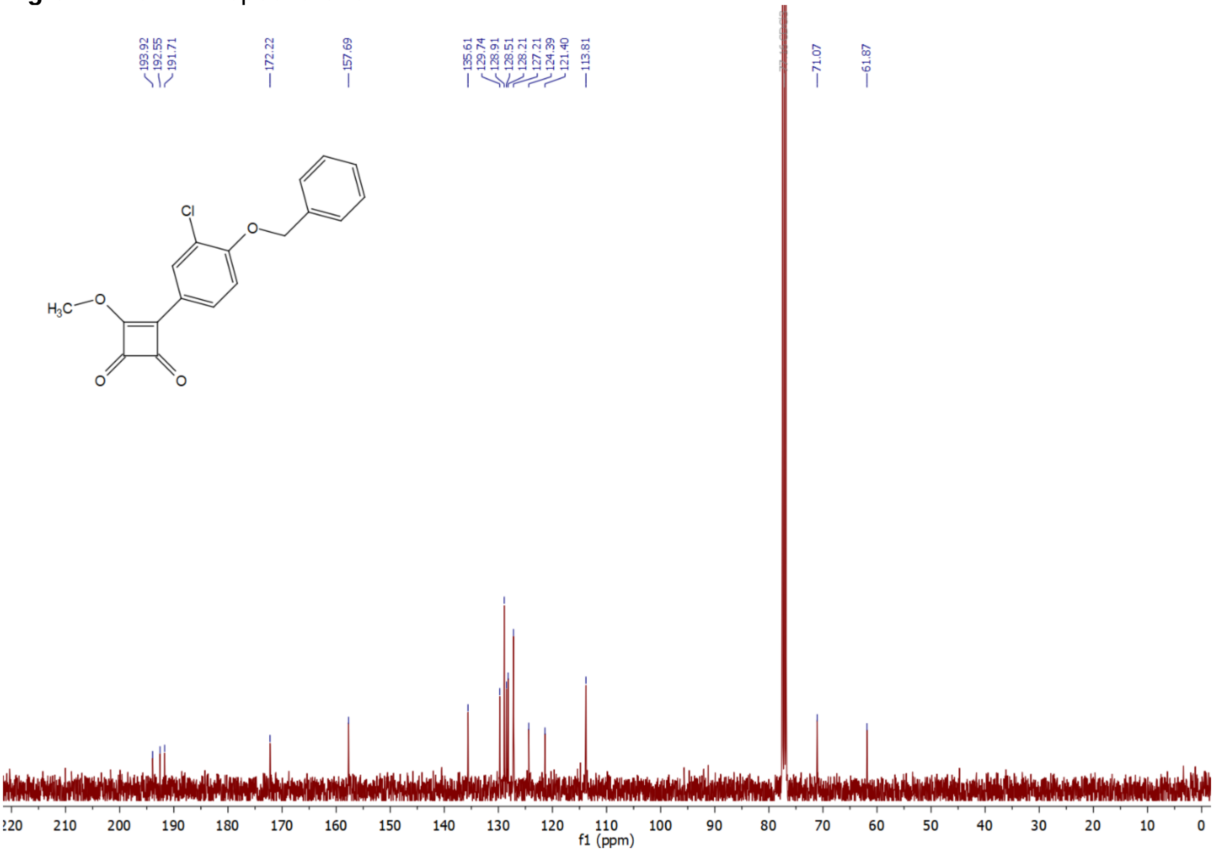

**Fig S46.** $^{1}H$ NMR spectra of **6k**

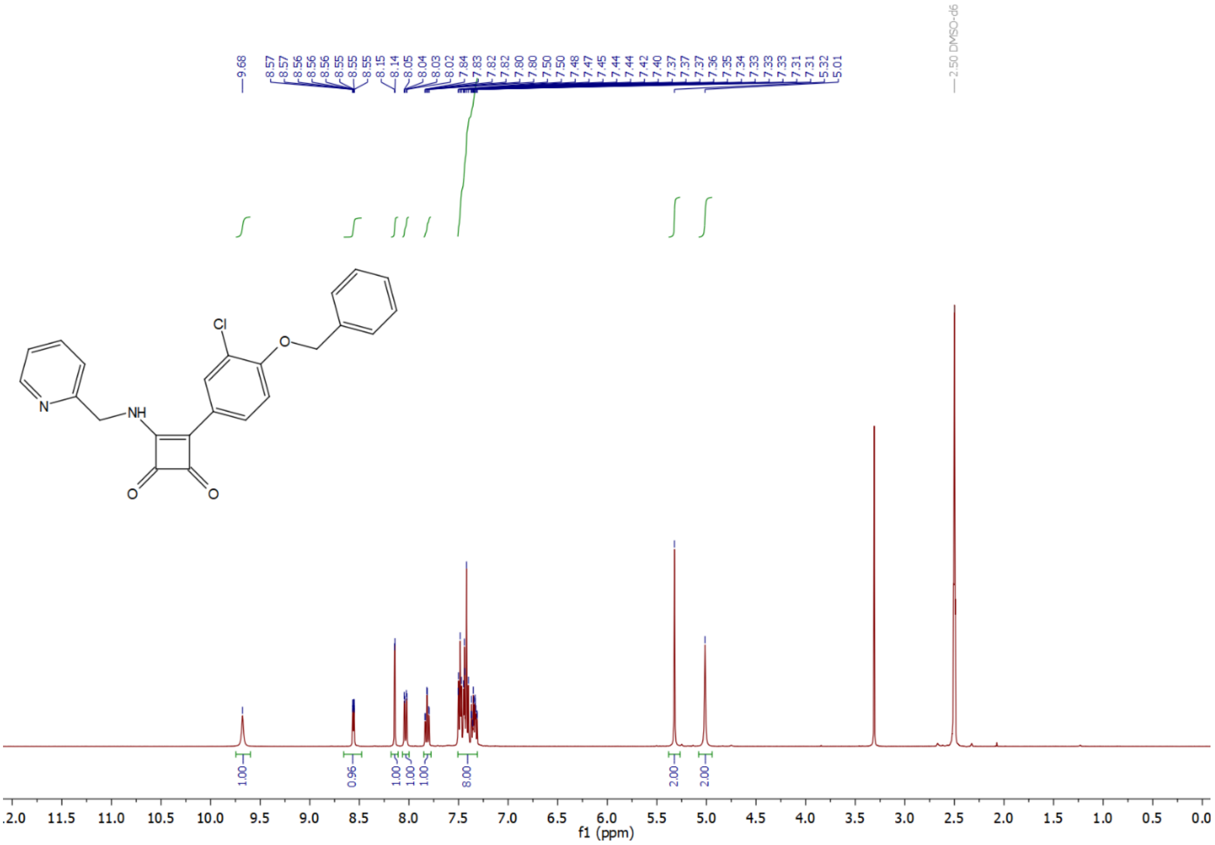

**Fig S47.** $^{13}C$ NMR spectra of **6k**

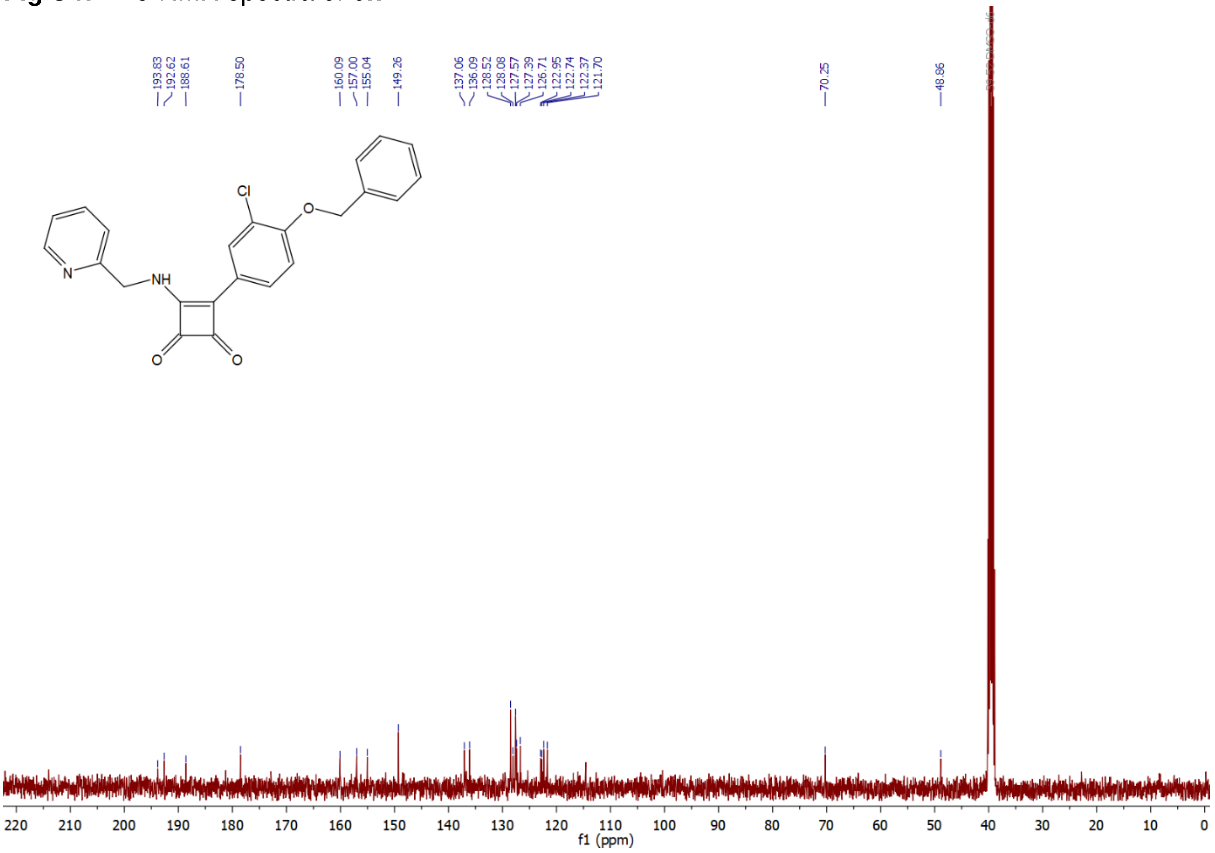

**Fig S48.** $^{1}$H NMR spectra of **5l**

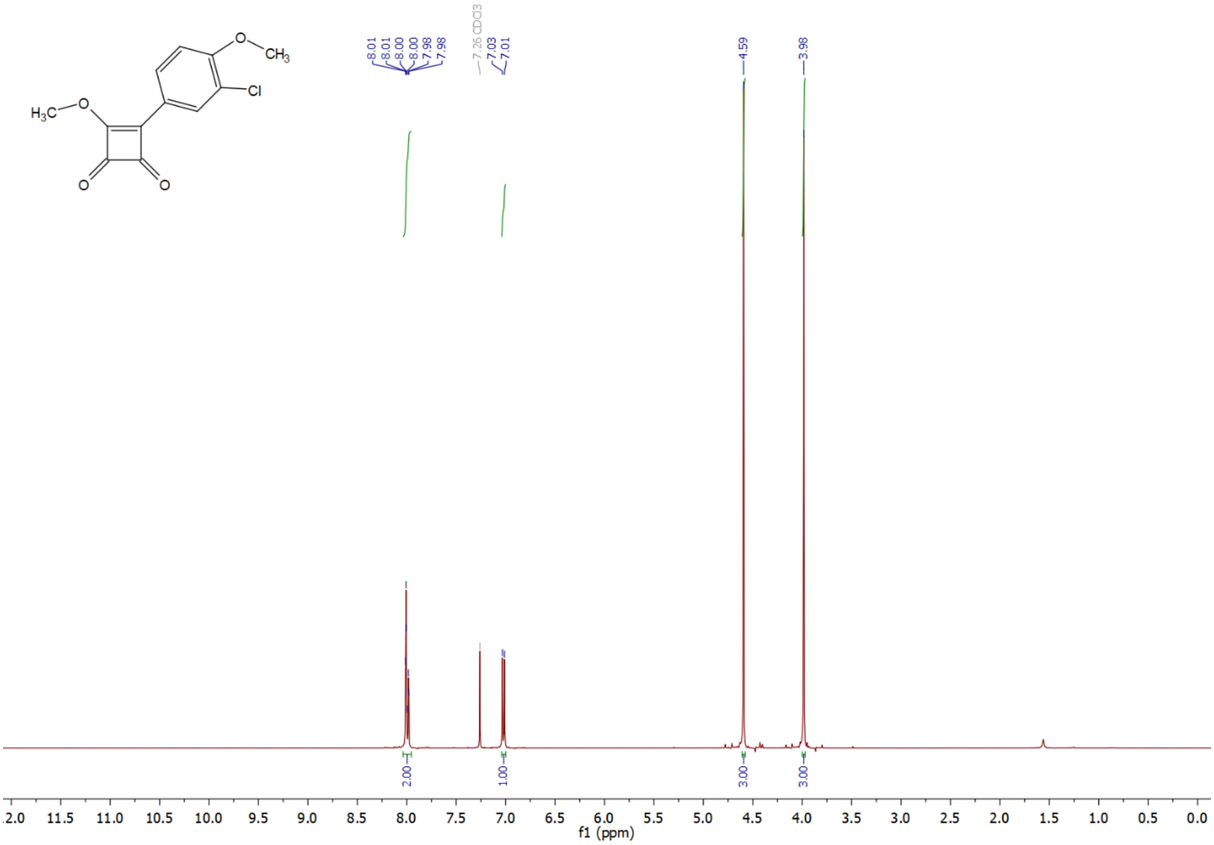

**Fig S49.** $^{13}$C NMR spectra of **5l**

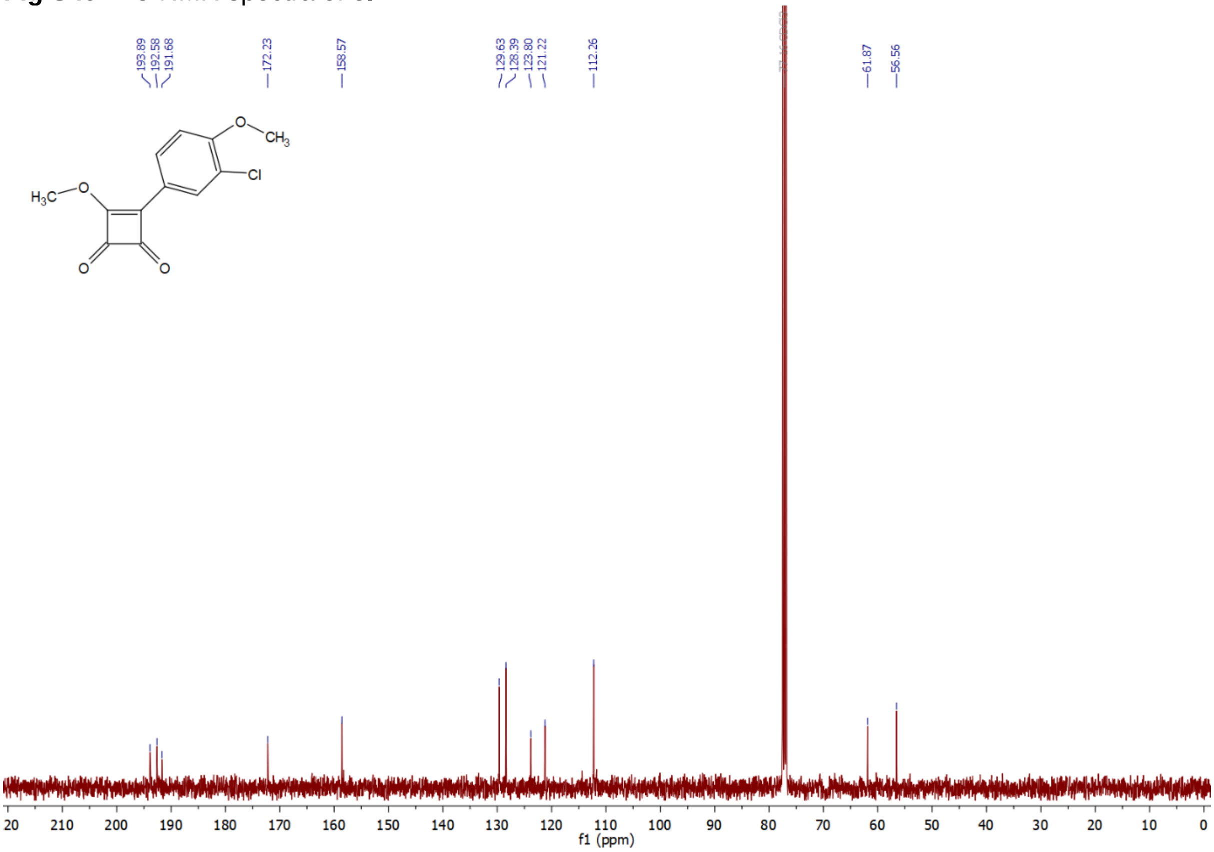

**Fig S50.** $^{1}$H NMR spectra of **6l**

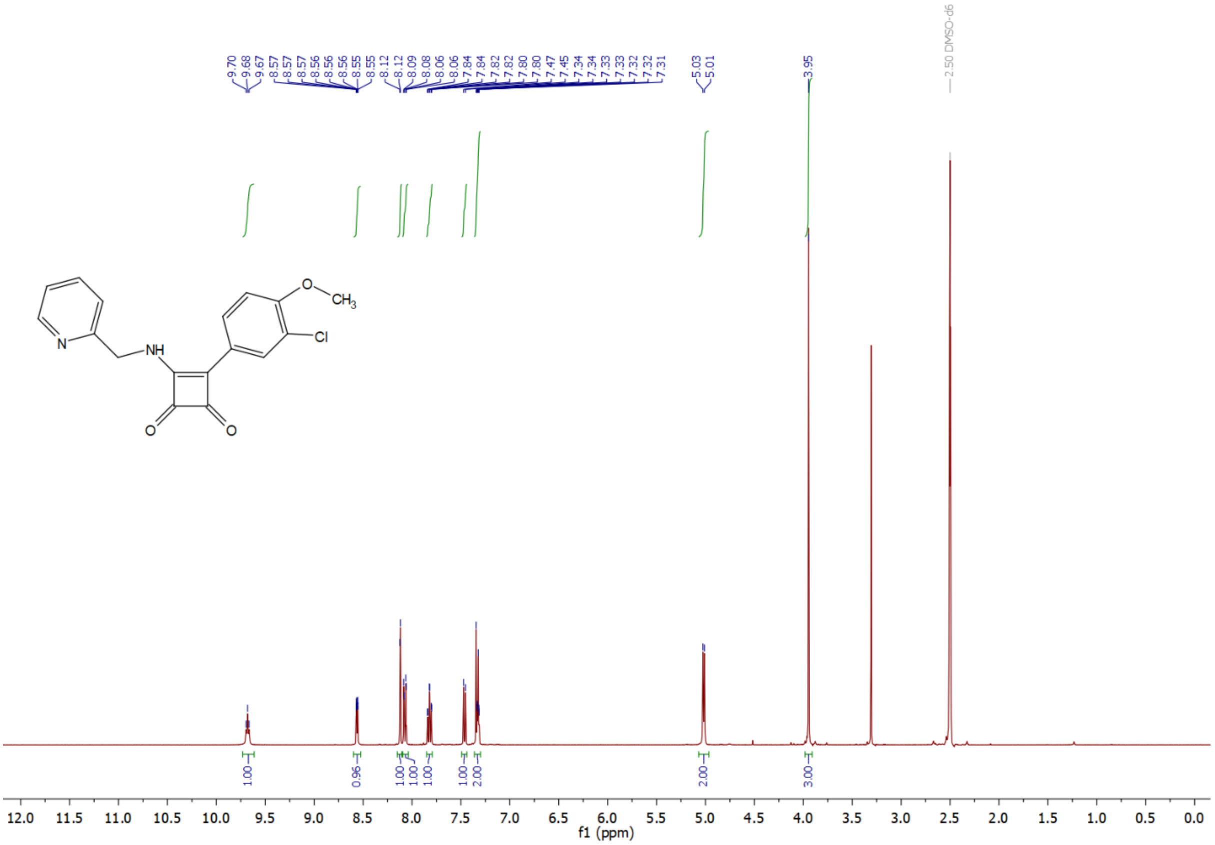


**Fig S51.** $^{13}$C NMR spectra of **6l**

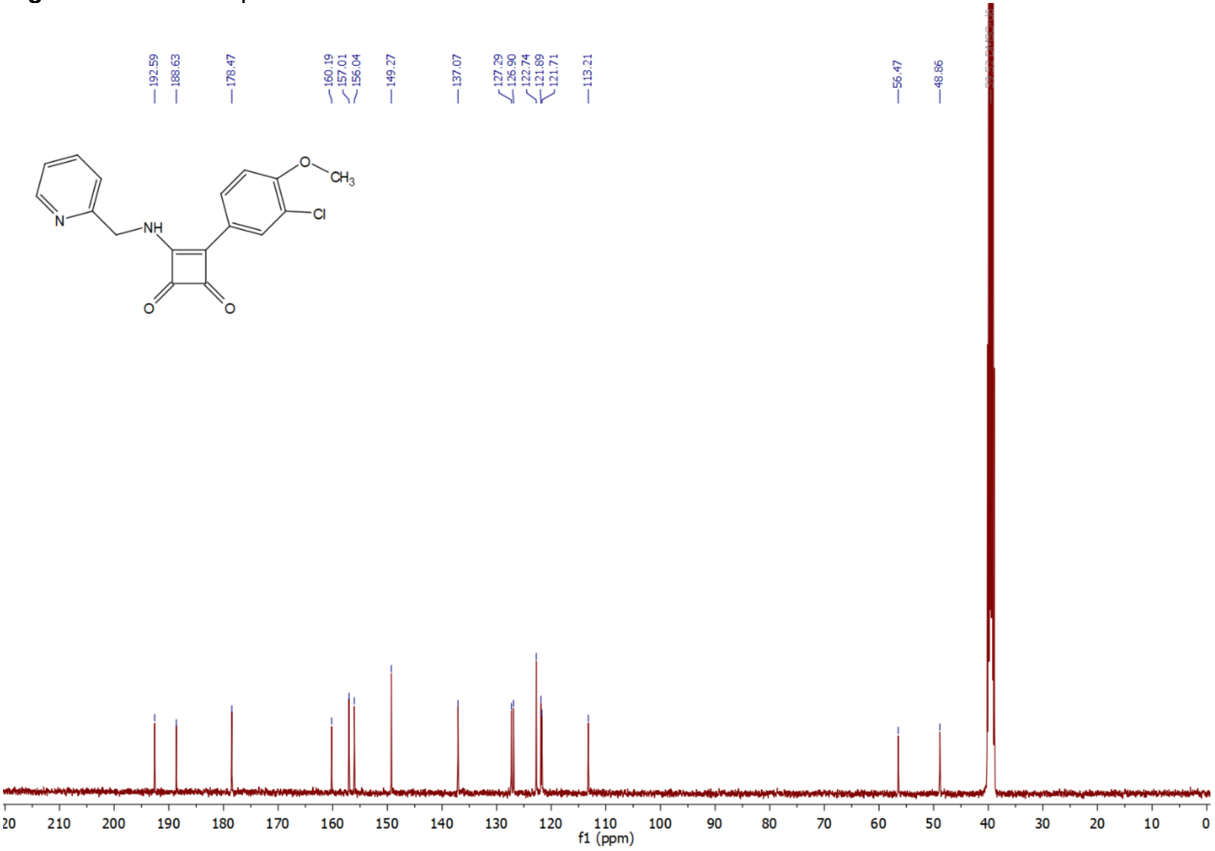

**Fig S52.** $^{1}$H NMR spectra of **5m**

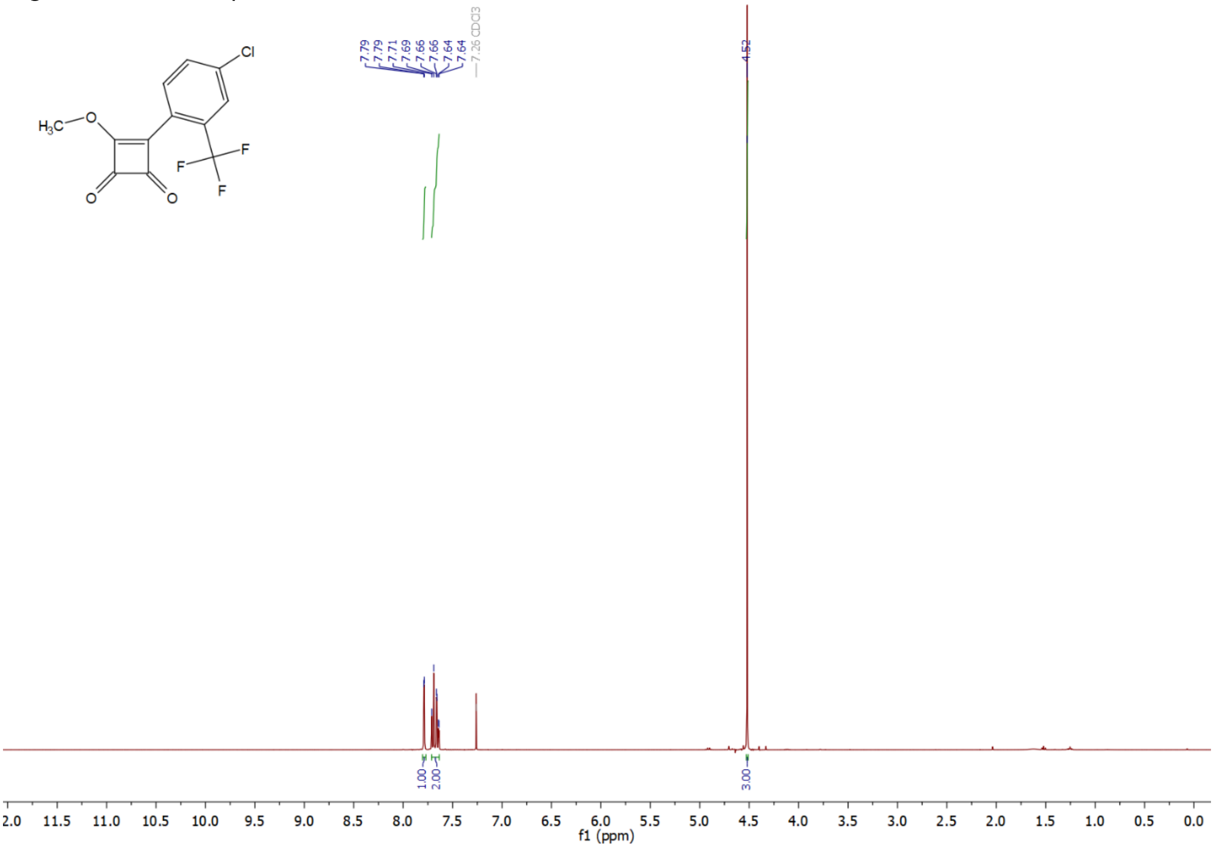


**Fig S53.** $^{13}$C NMR spectra of **5m**

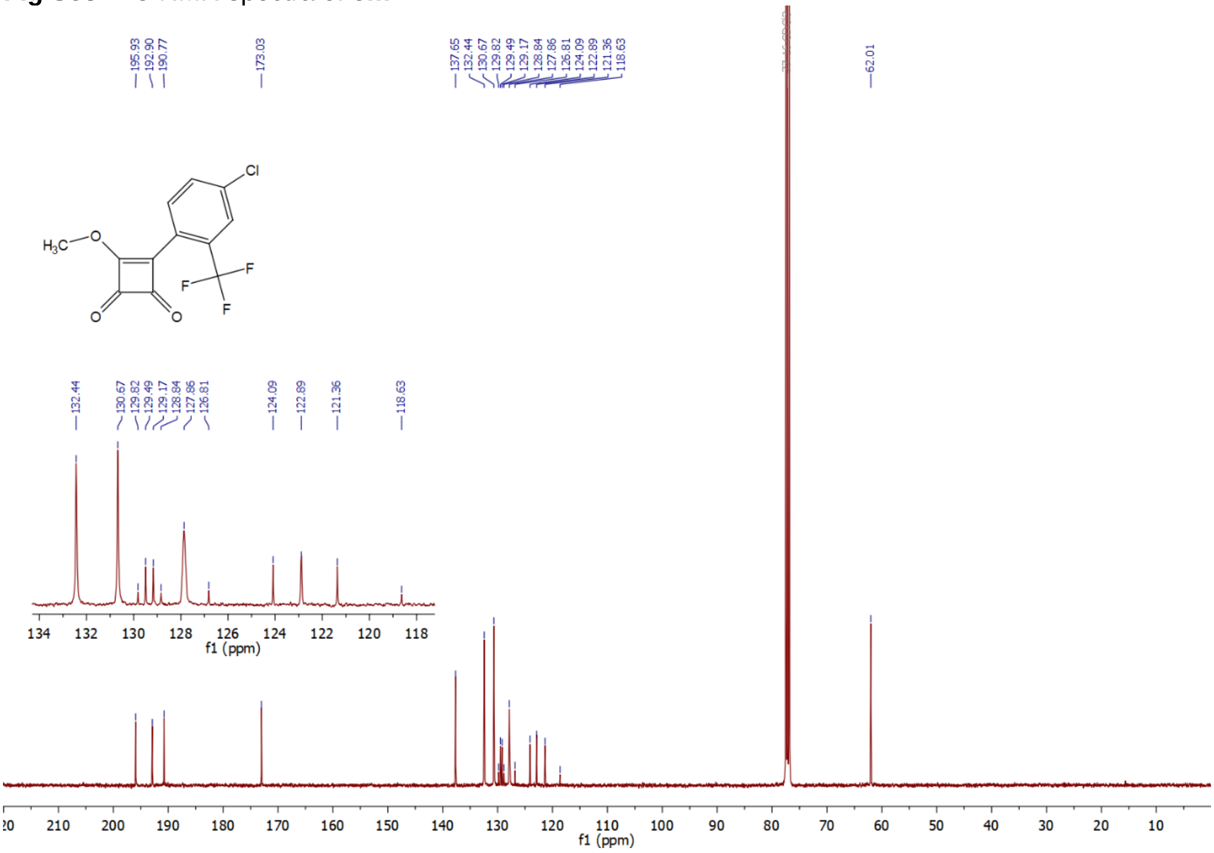

**Fig S54.** $^{1}$H NMR spectra of **6m** at 25 °C

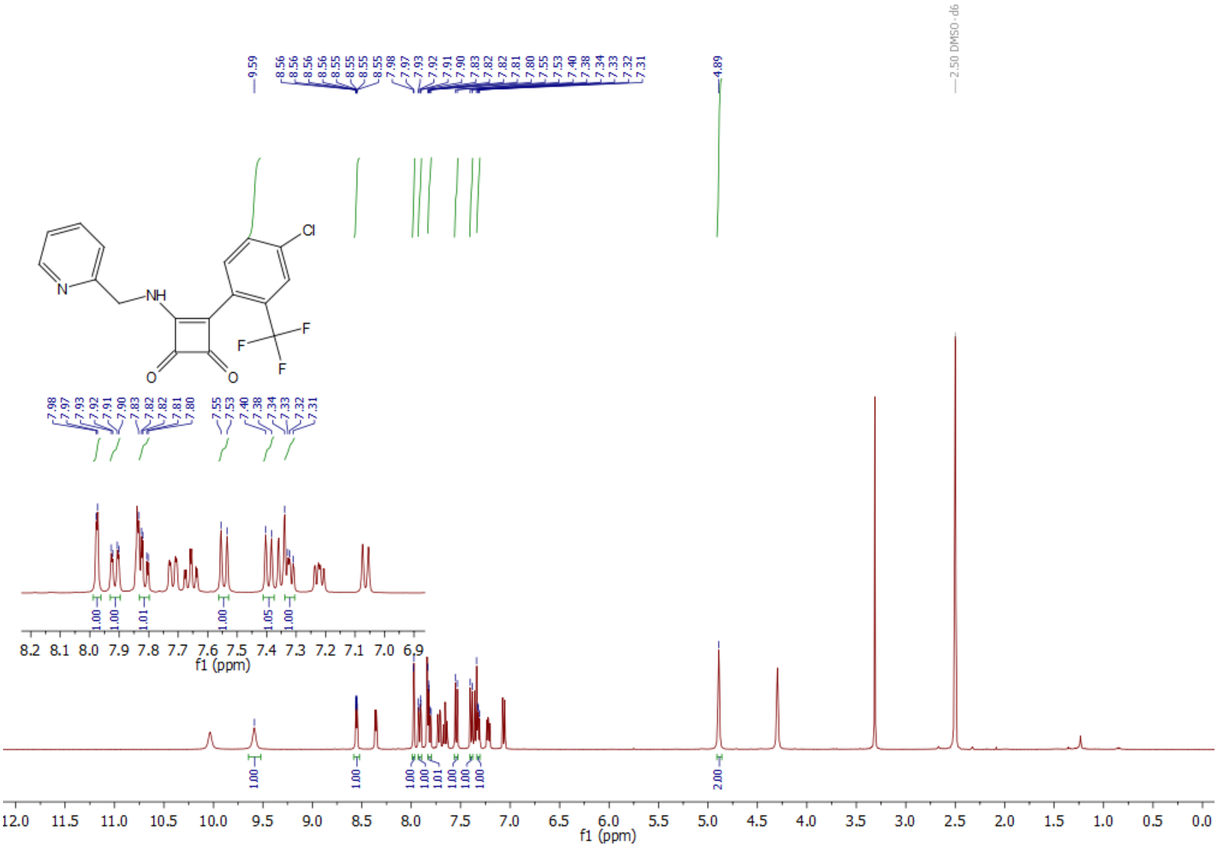

**Fig S55.** $^{1}$H NMR spectra of **6m** at 45 °C

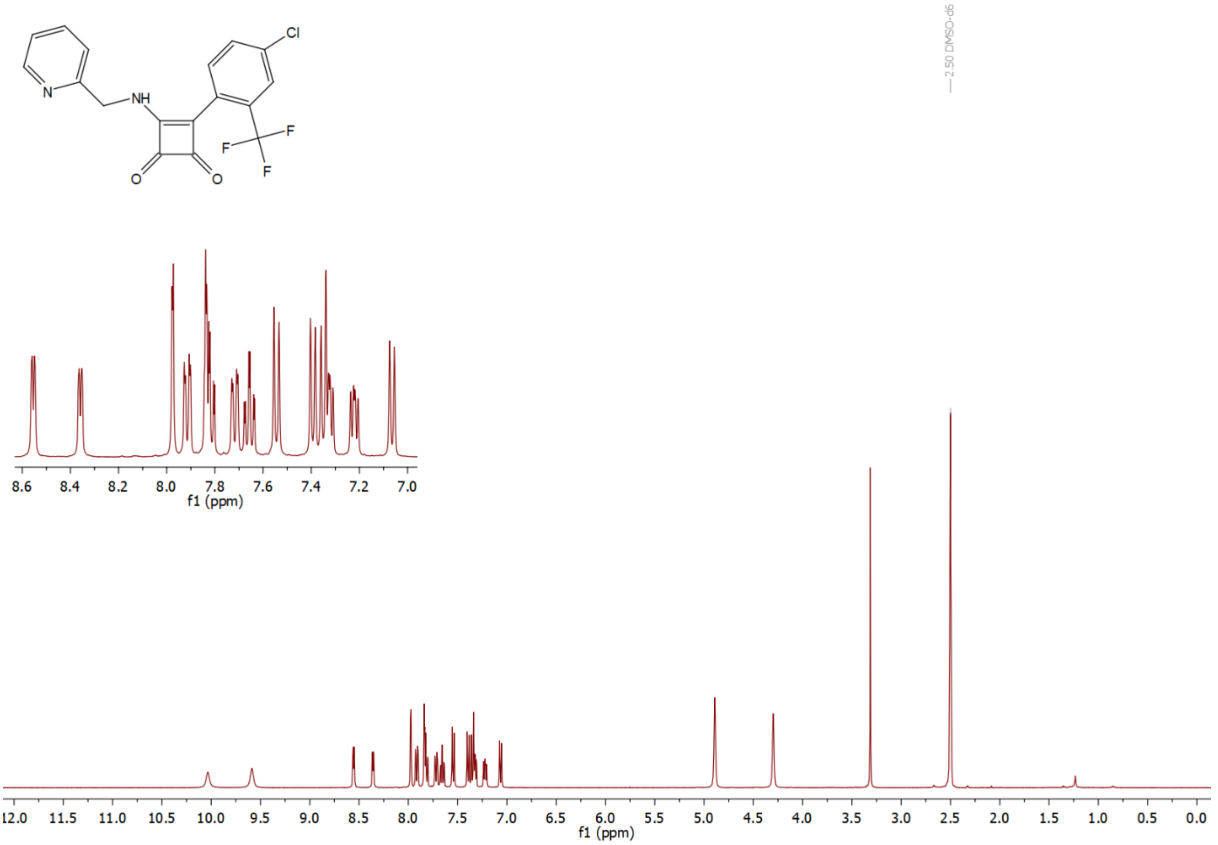

**Fig S56.** $^{1}$H NMR spectra of **6m** at 65 °C

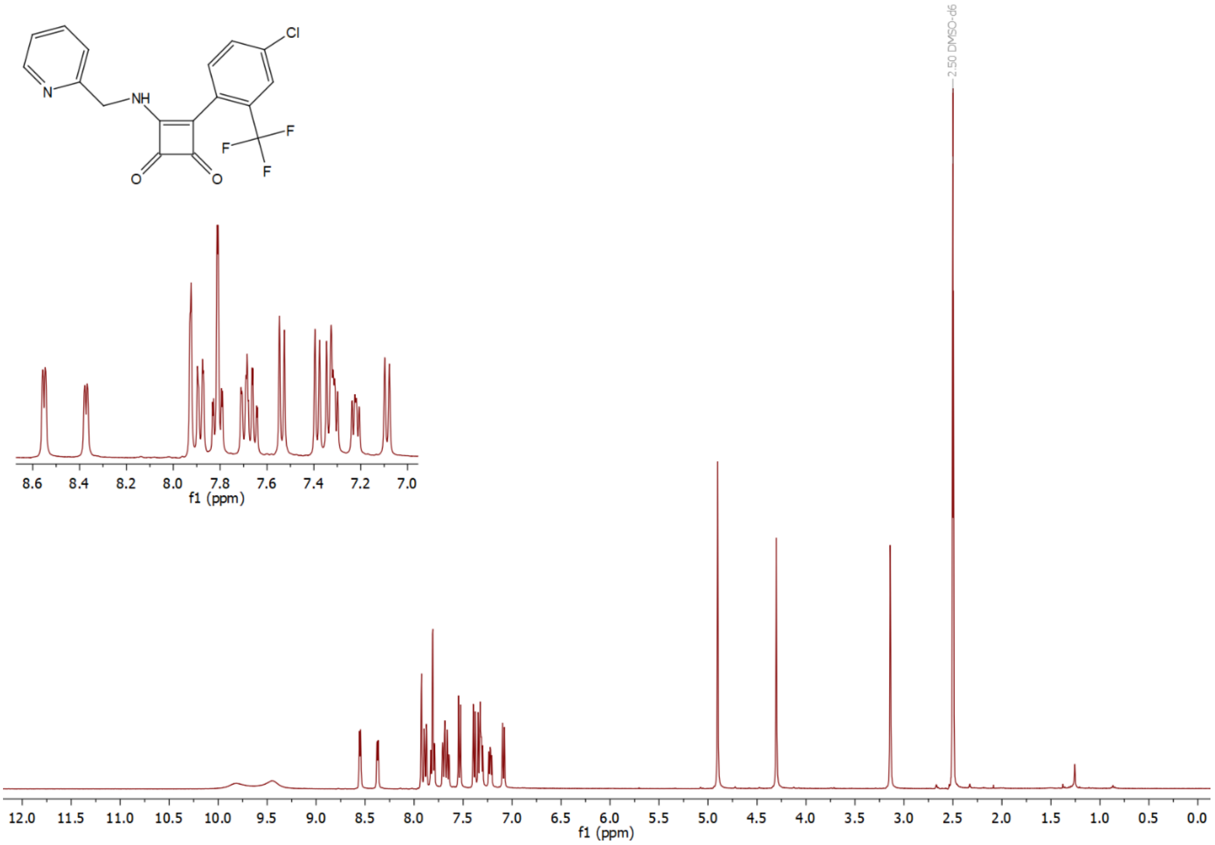


**Fig S57.** $^{1}$H NMR spectra of **6m** at 85 °C

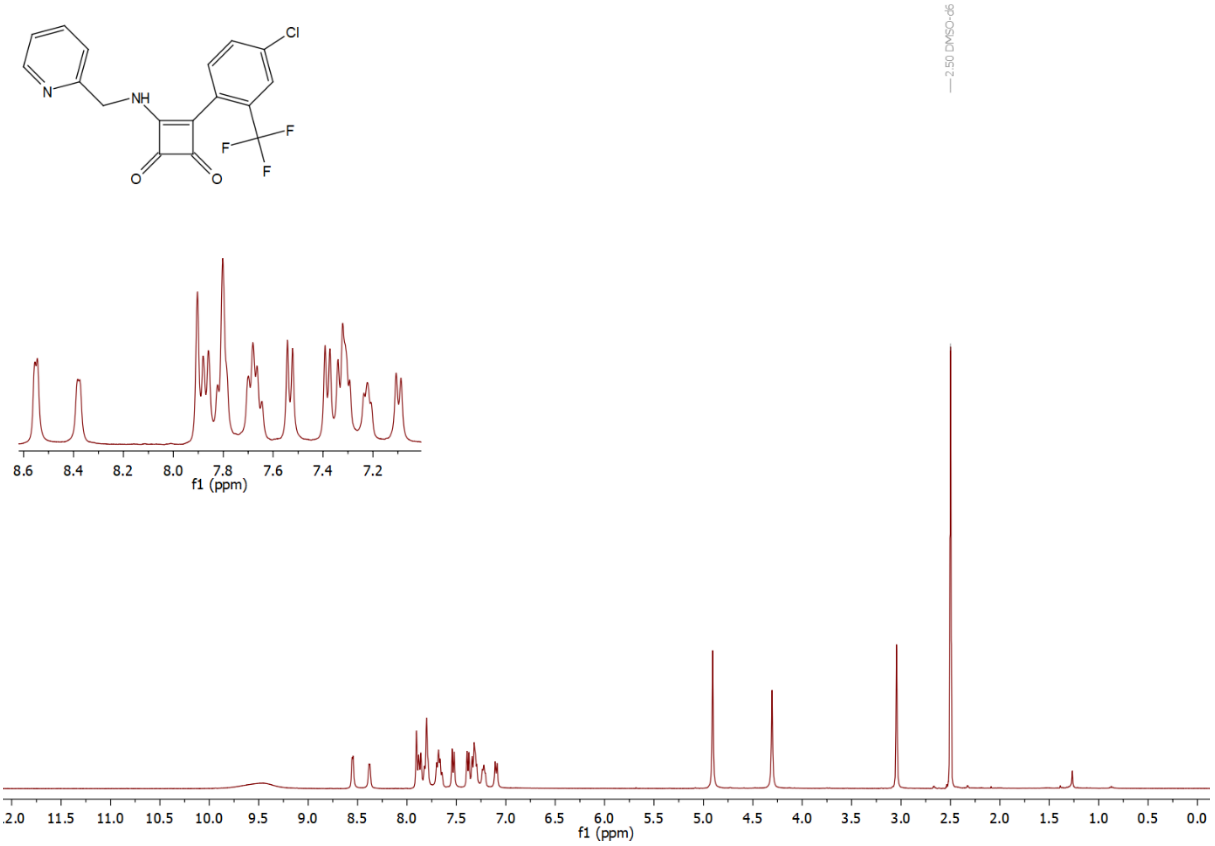

**Fig S58.** $^{1}$H NMR spectra of **6m** at 105 °C

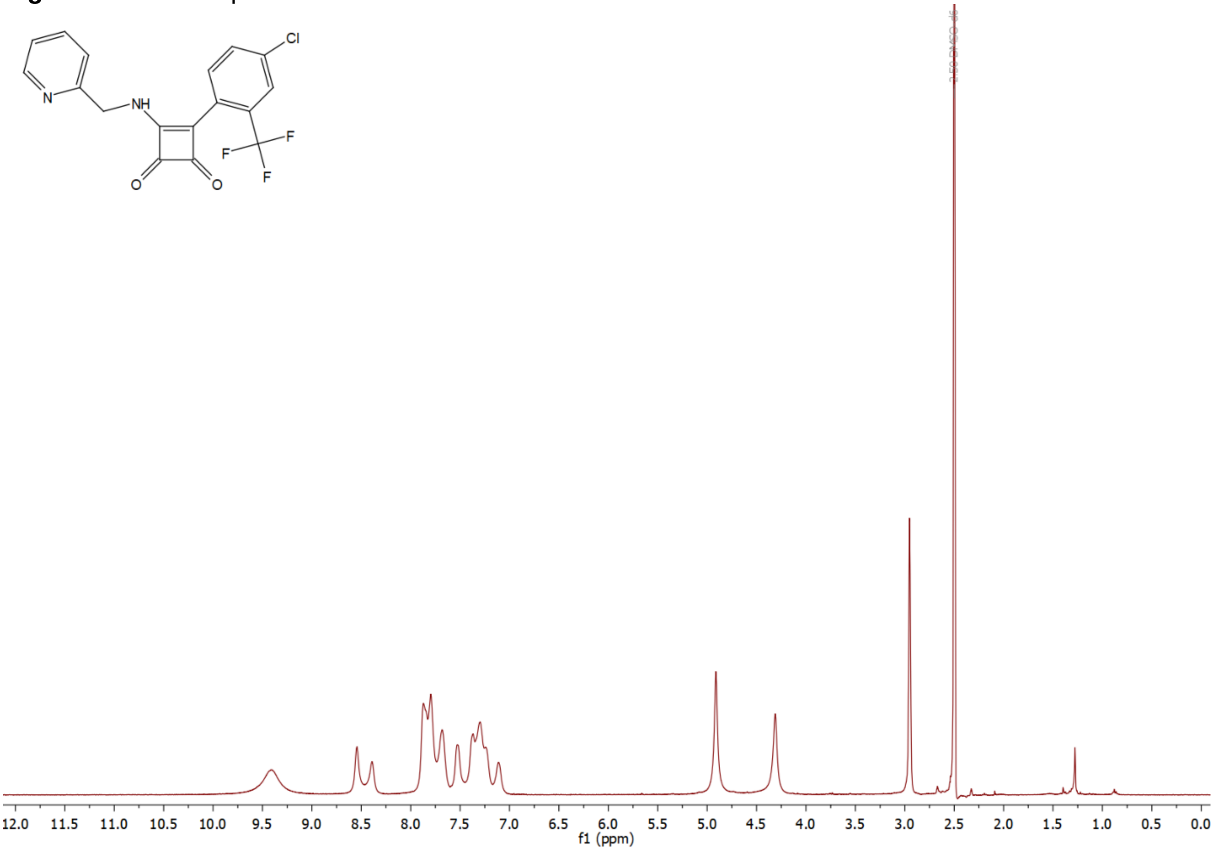


**Fig S59.** $^{19}$F NMR spectra of **6m** at 25 °C

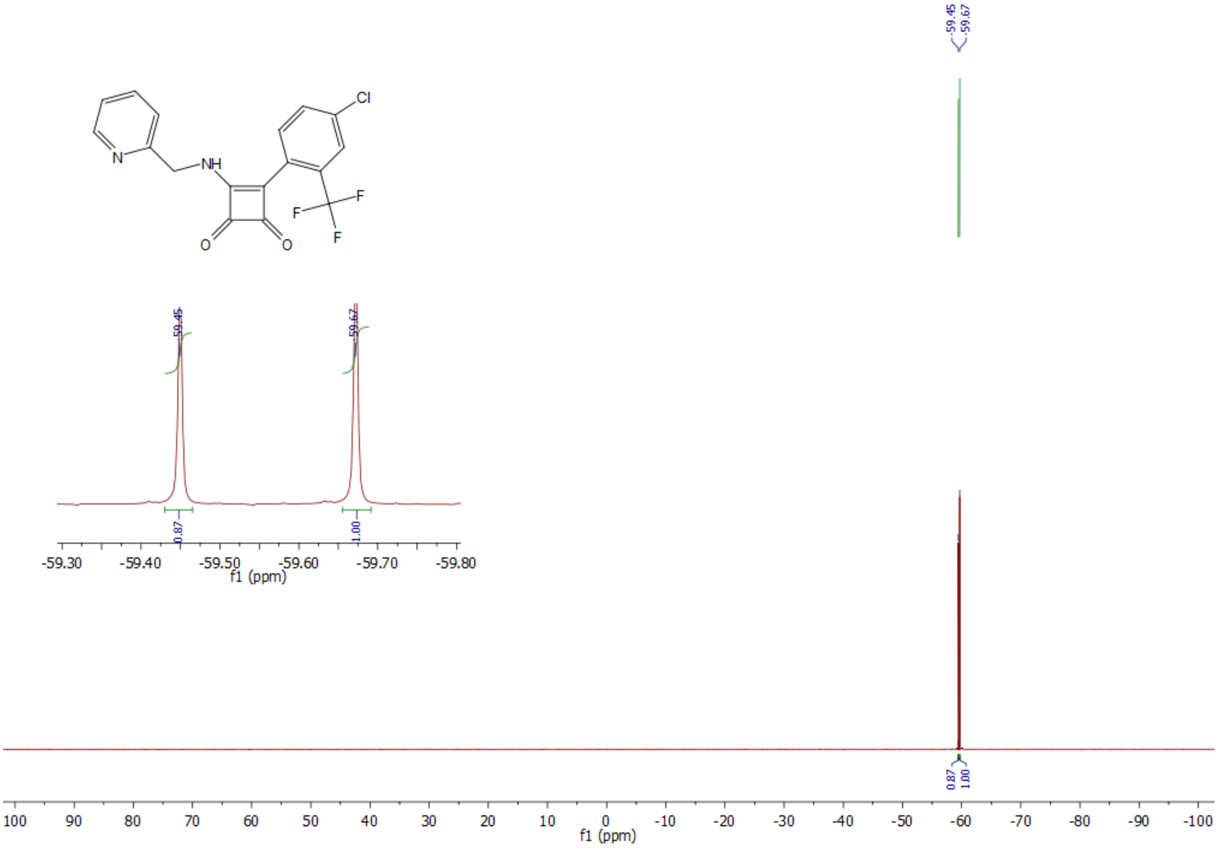

**Fig S60.** $^{19}$F NMR spectra of **6m** at 105 °C

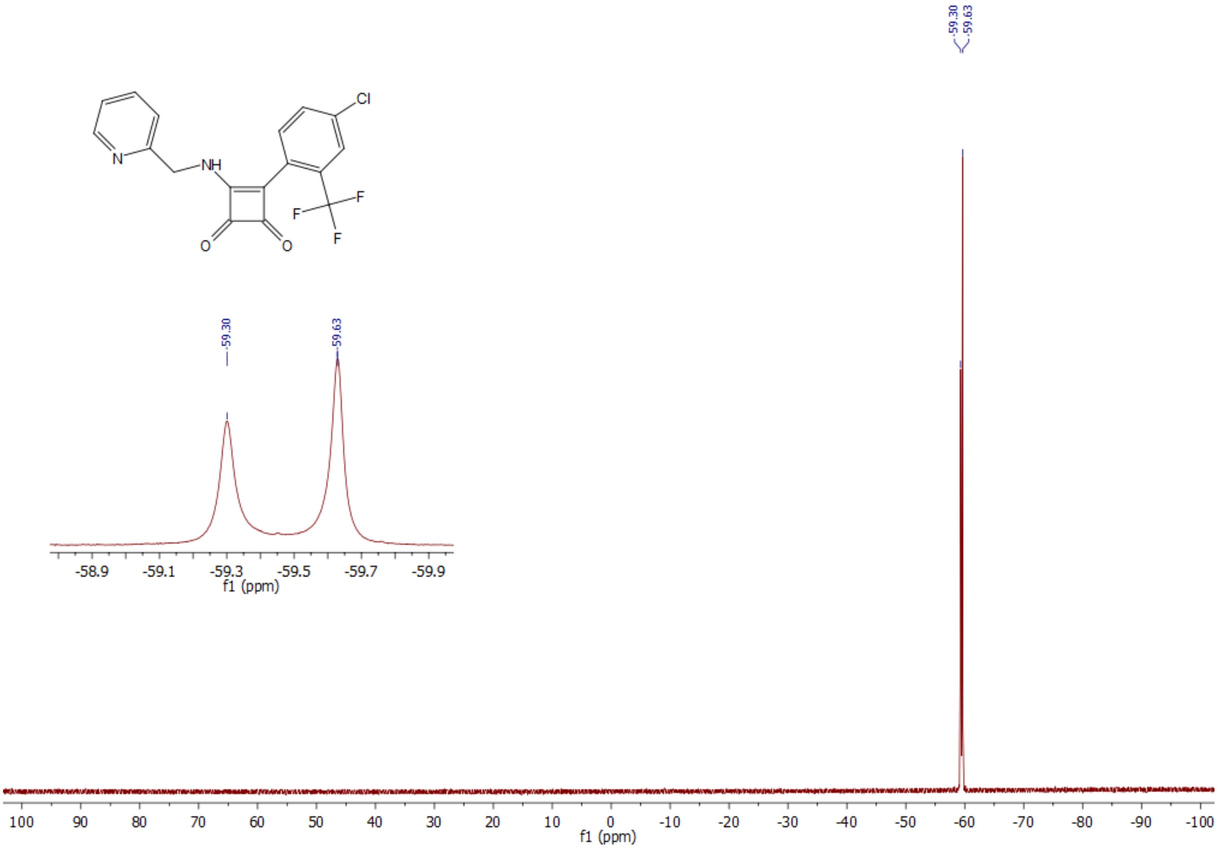

**Fig S61.** $^{13}$C NMR spectra of **6m**

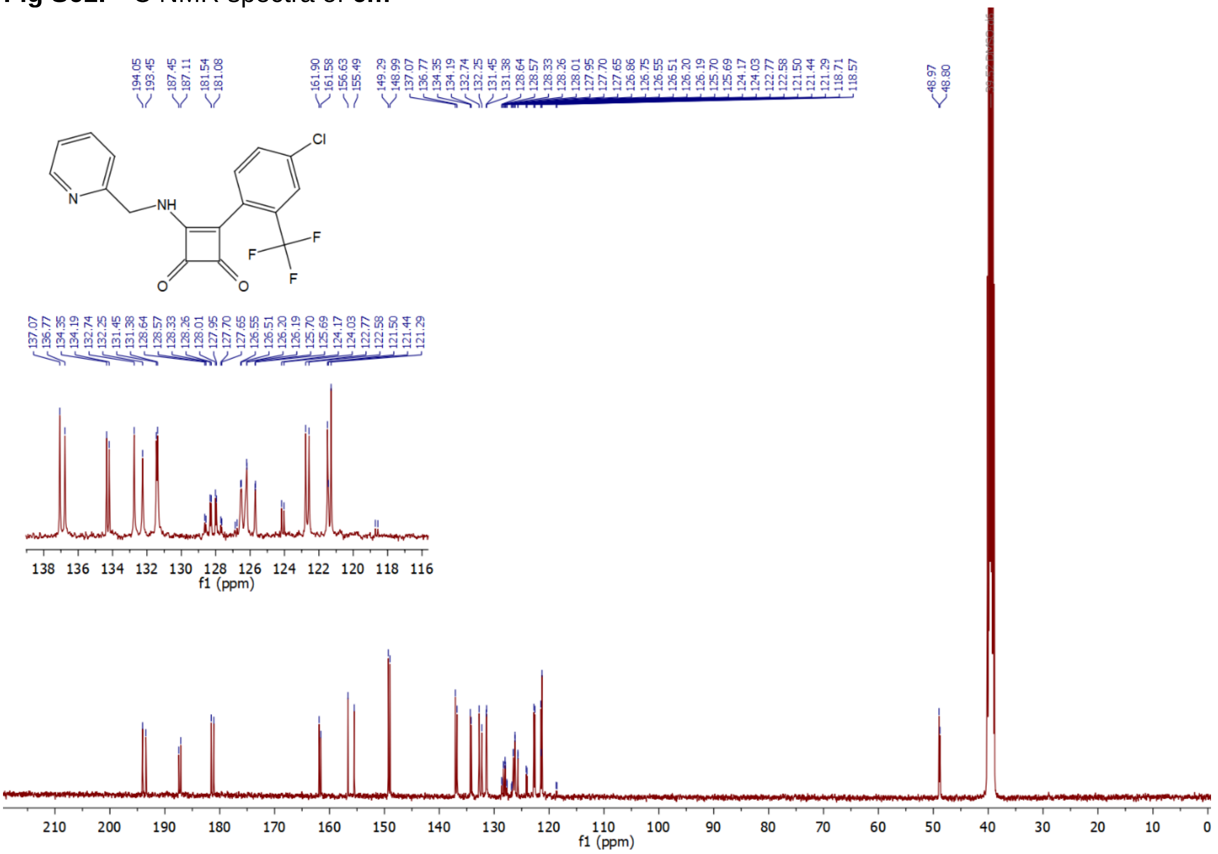

**Fig S62.** $^{1}$H NMR spectra of **5n**

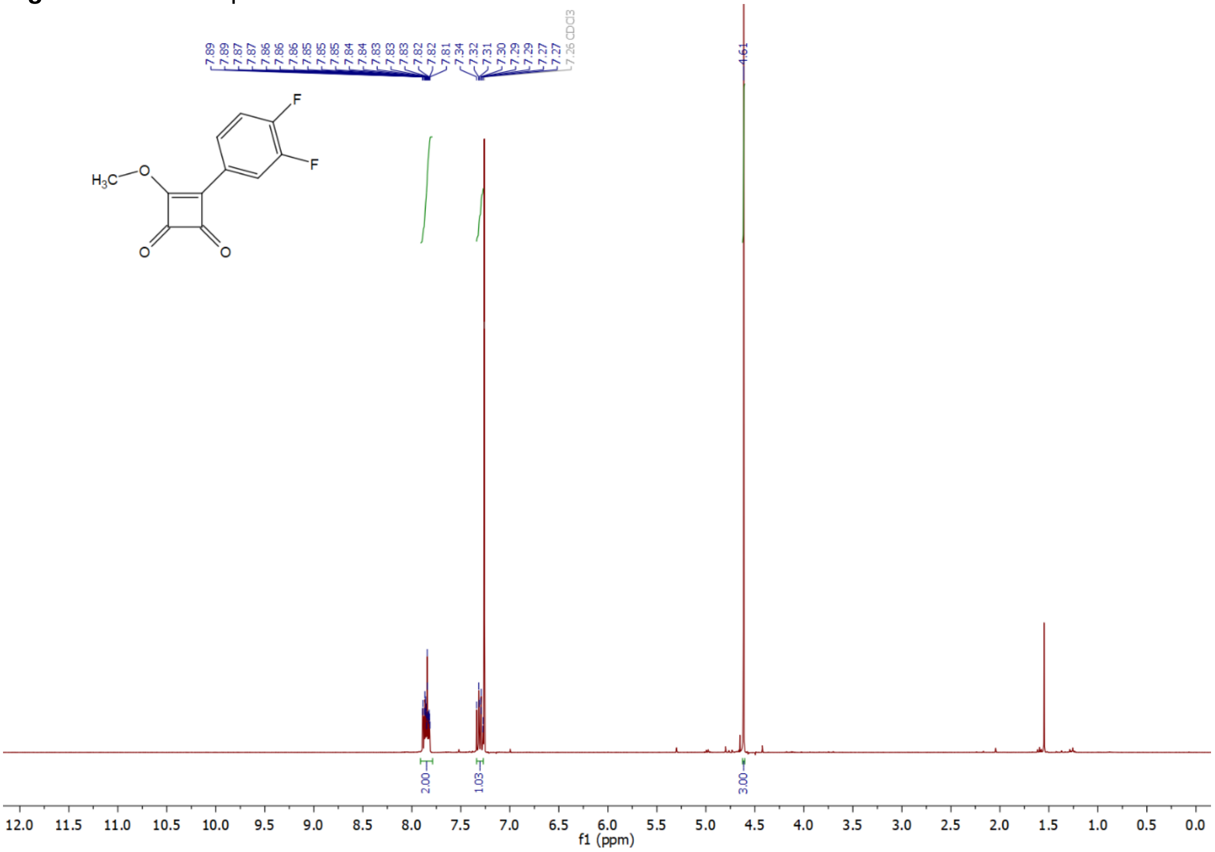


**Fig S63.** $^{13}$C NMR spectra of **5n**

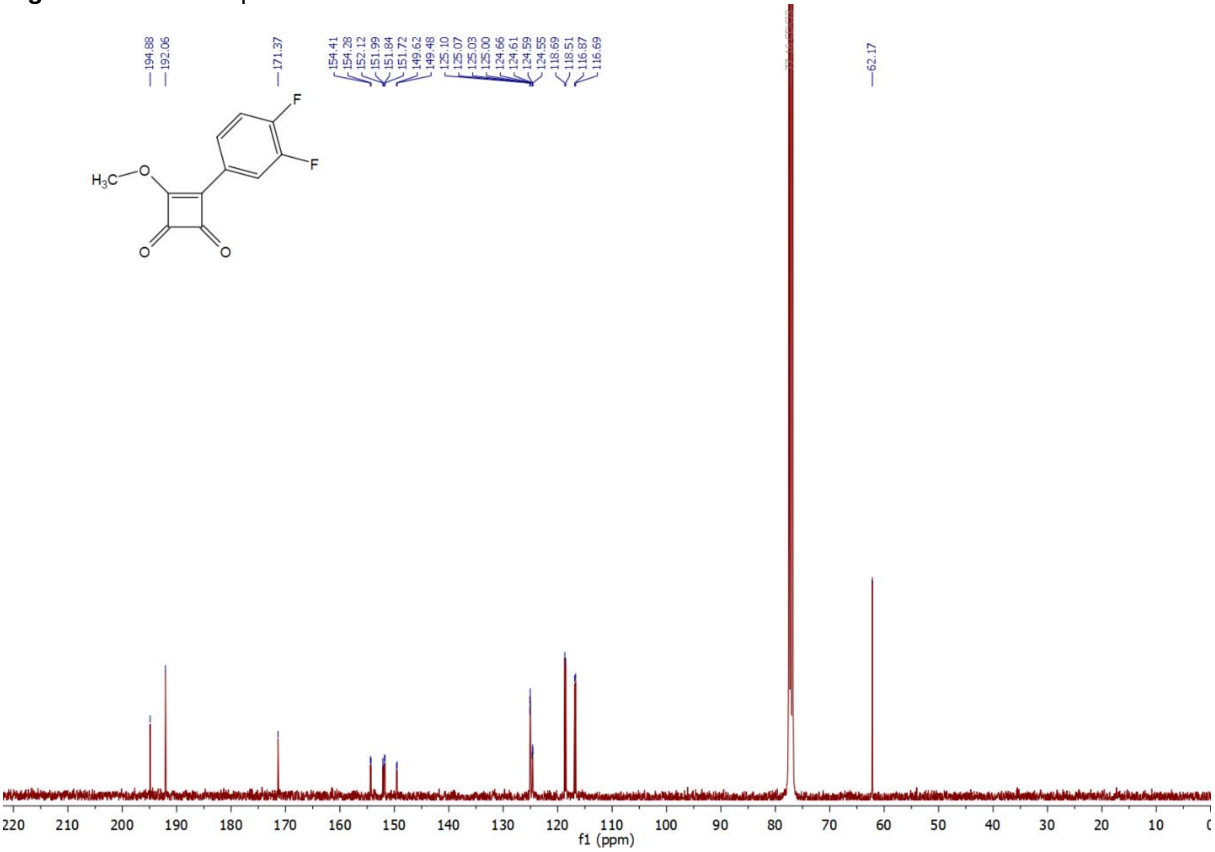

**Fig S64.** $^{1}$H NMR spectra of **6n**

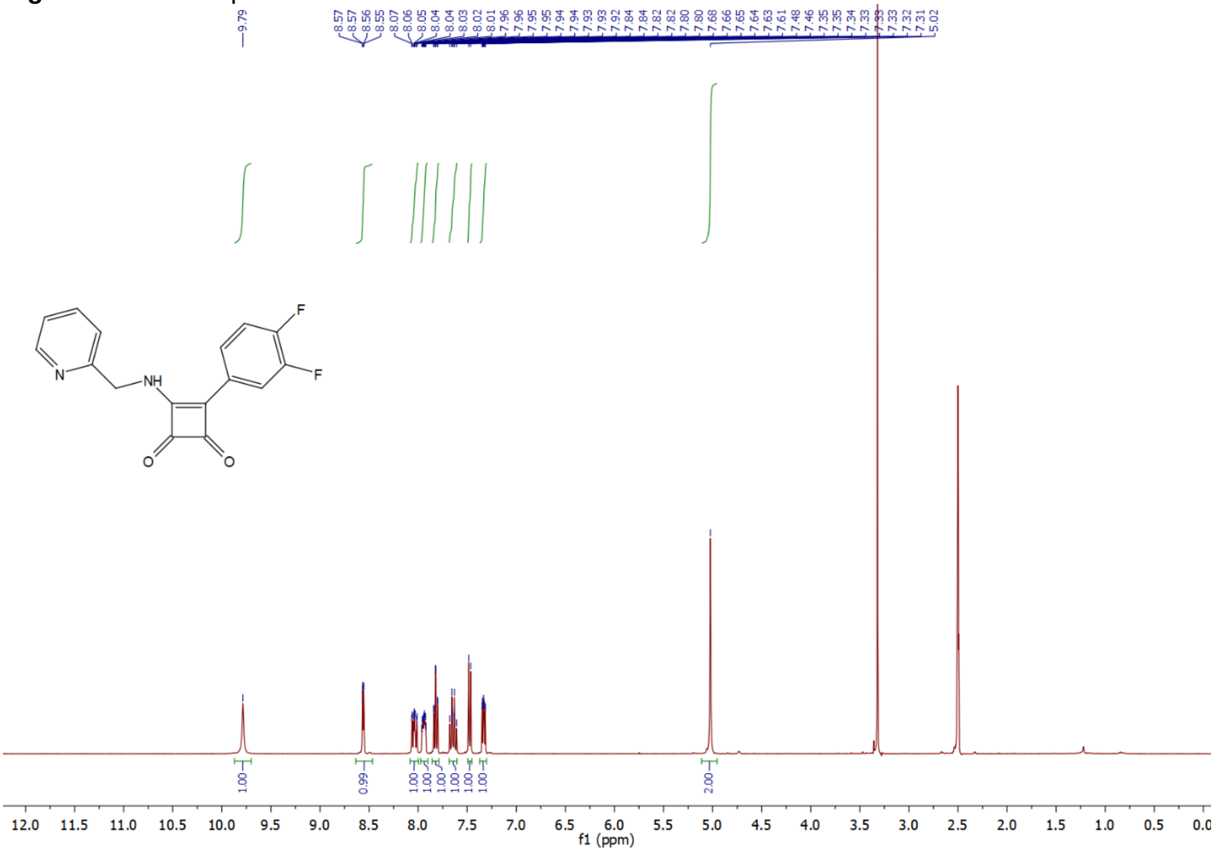

**Fig S65.** $^{13}$C NMR spectra of **6n**

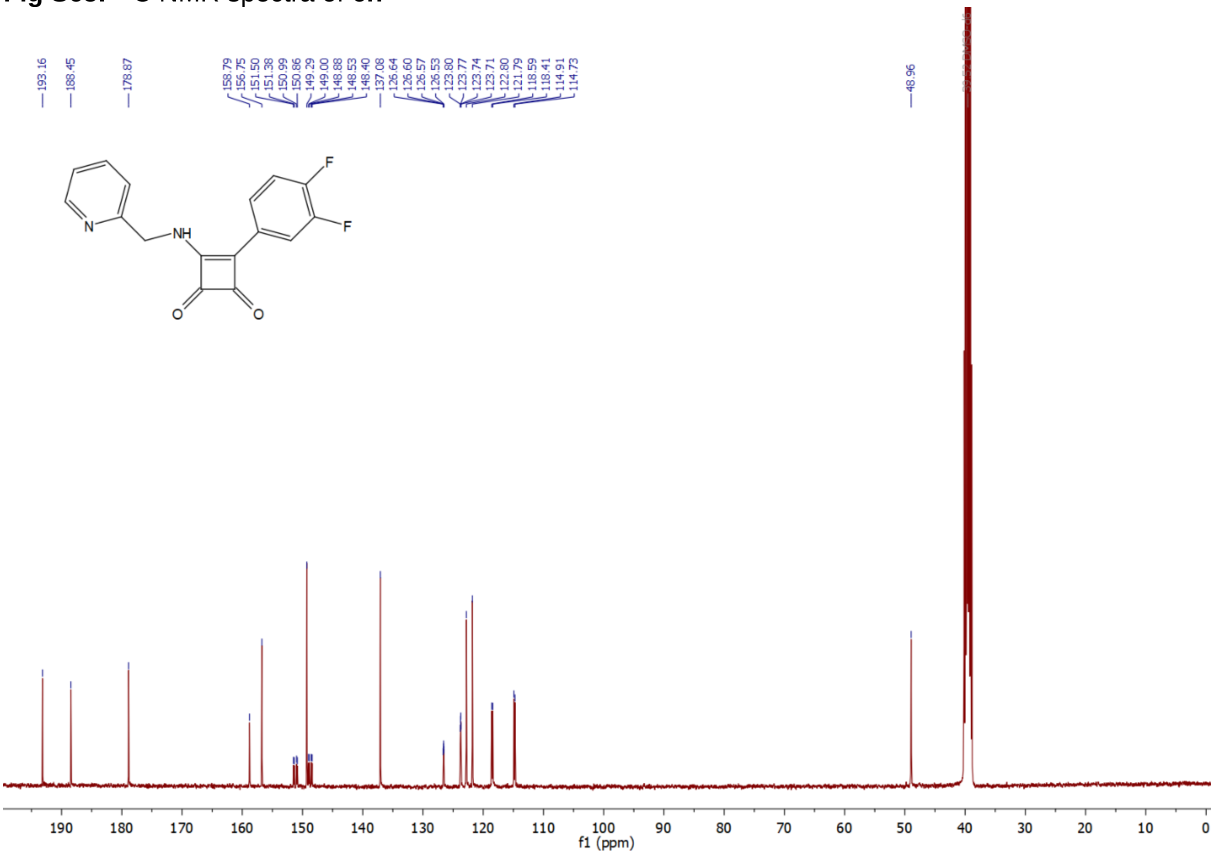

**Fig S66.** $^{1}$H NMR spectra of **5o**

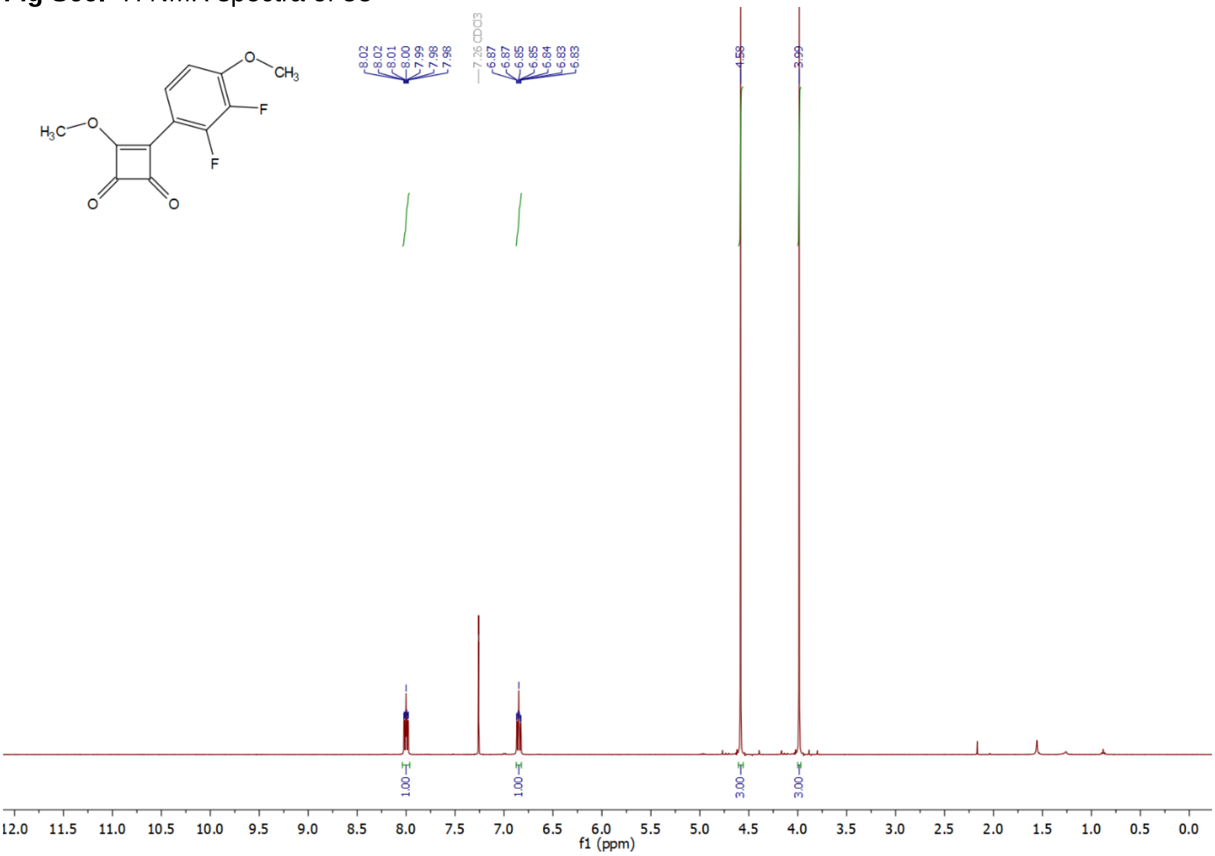


**Fig S67.** $^{13}$C NMR spectra of **5o**

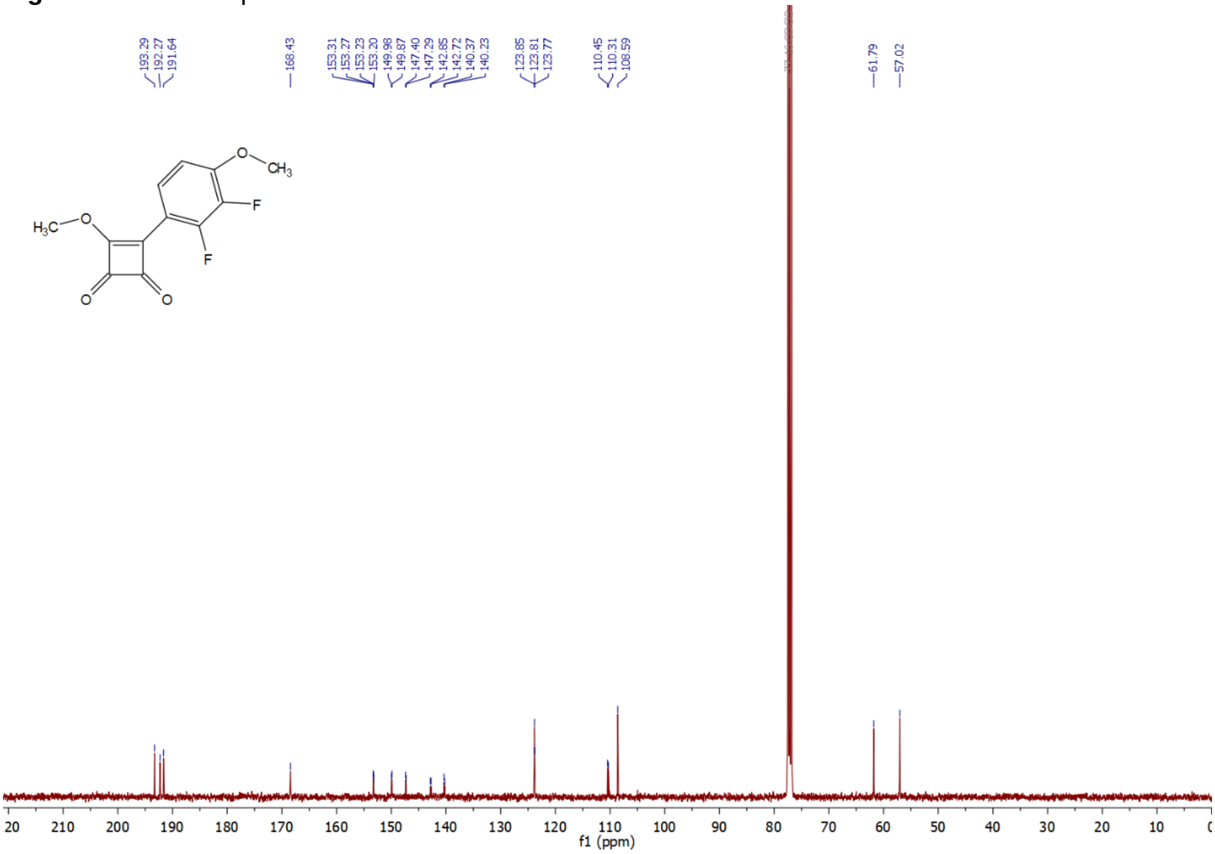

**Fig S68.** $^{1}$H NMR spectra of **6o**

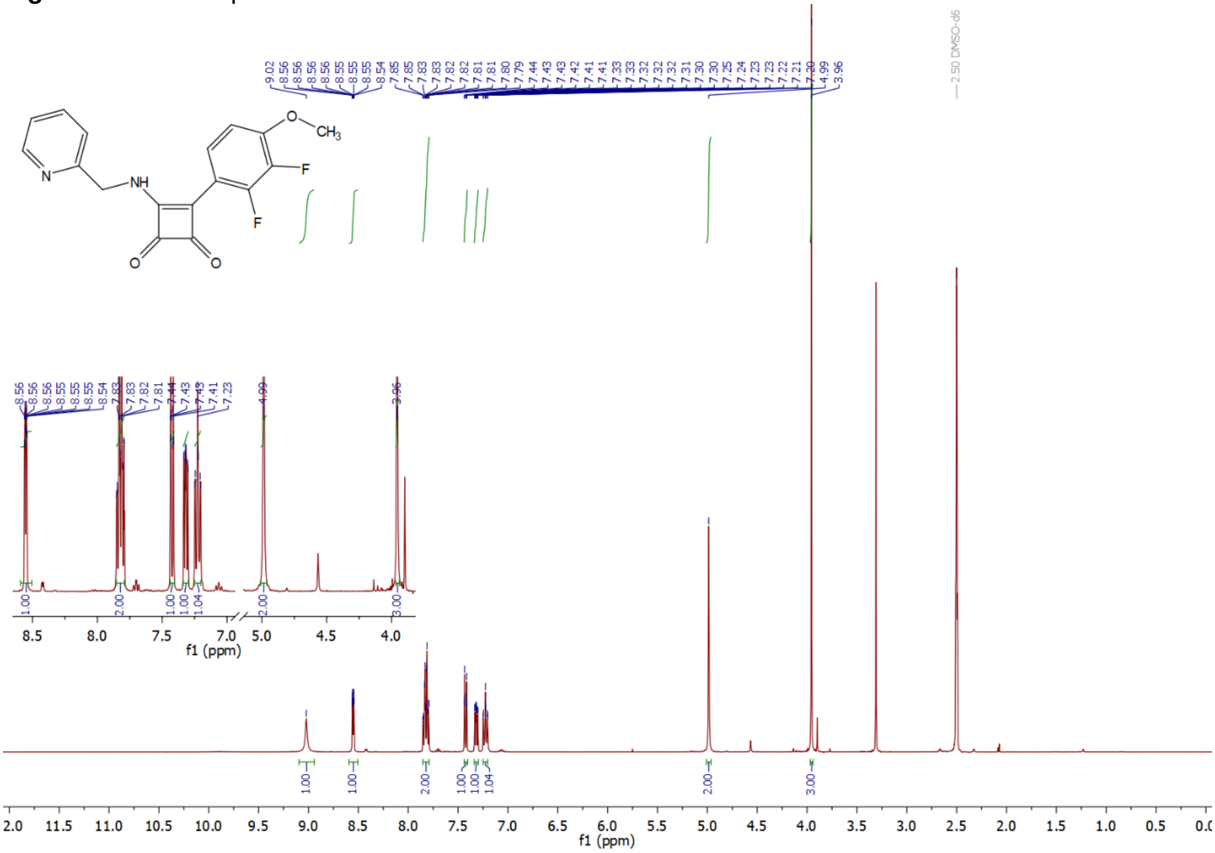


**Fig S69.** $^{13}$C NMR spectra of **6o**

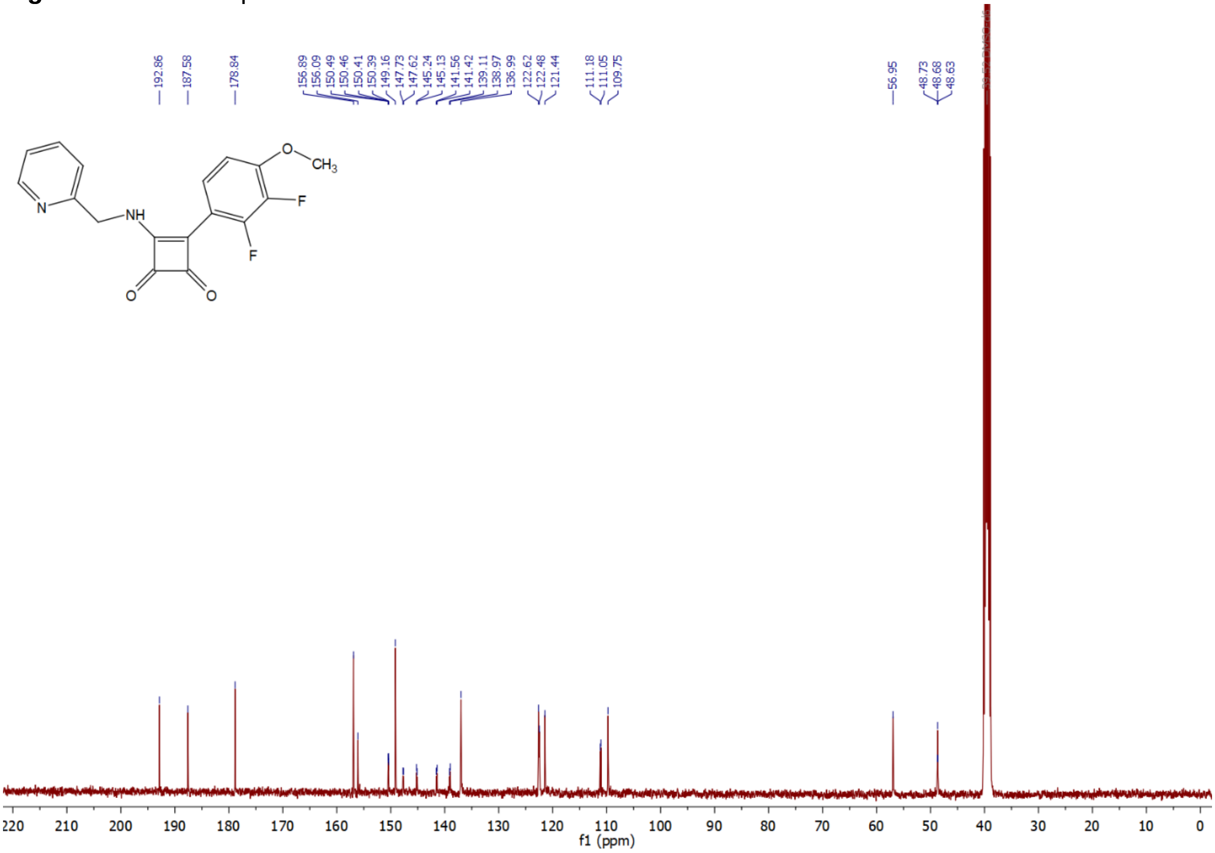

**Fig S70.** $^{1}$H NMR spectra of **5p**

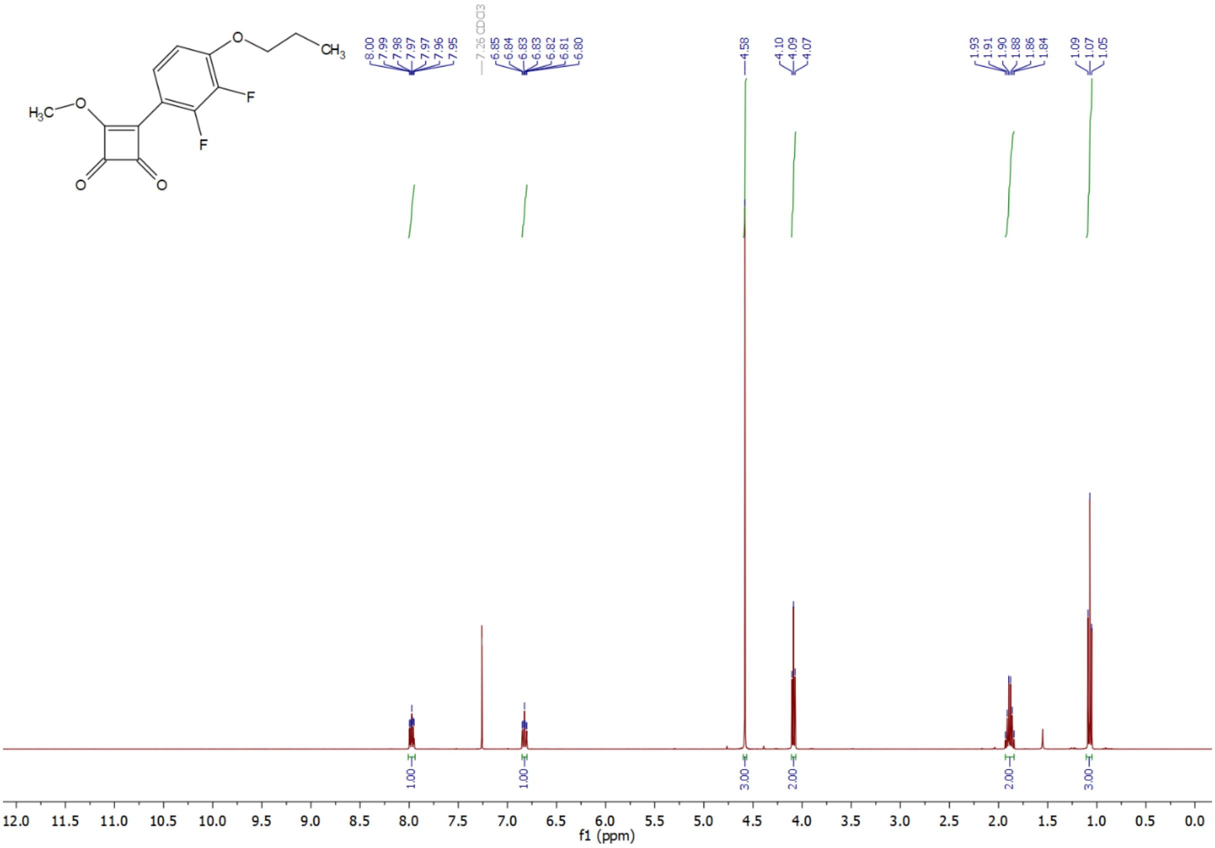

**Fig S71.** $^{13}$C NMR spectra of **5p**

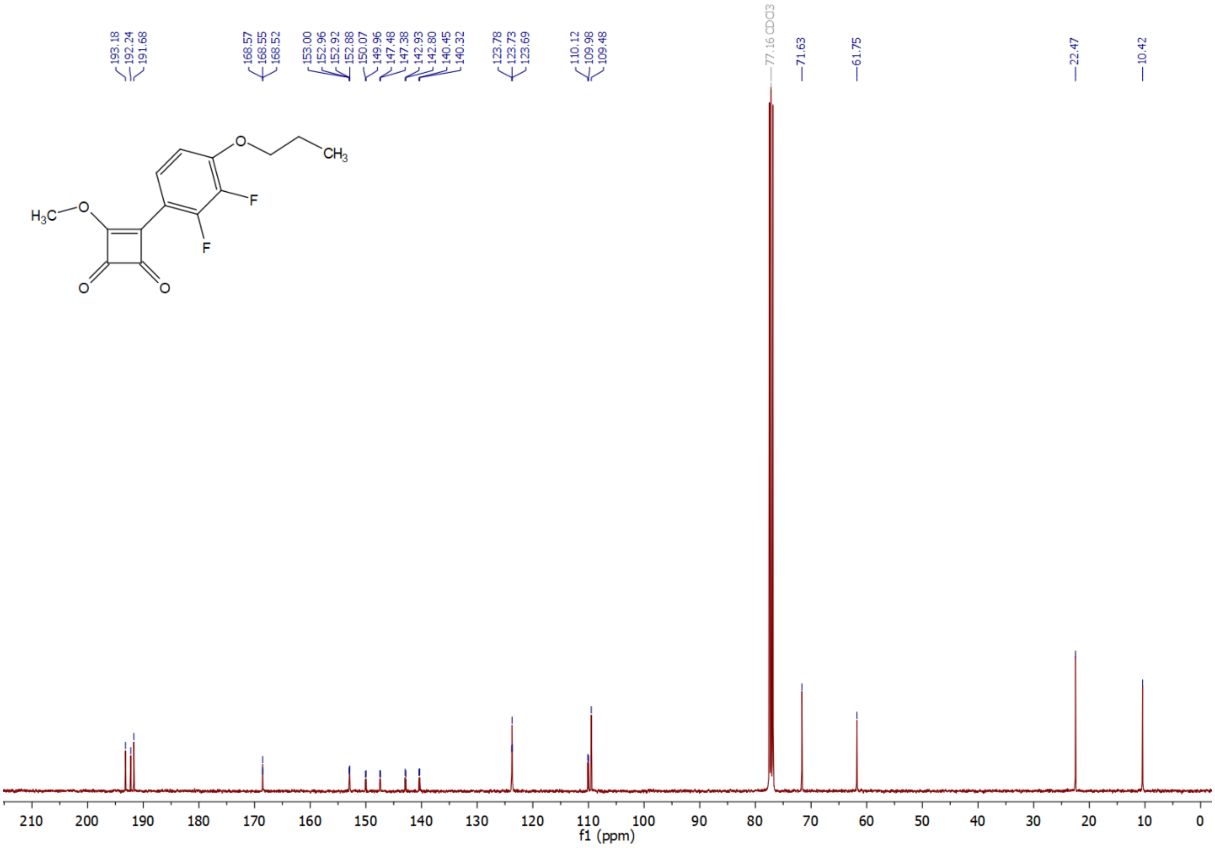

**Fig S72.** $^{1}$H NMR spectra of **6p**

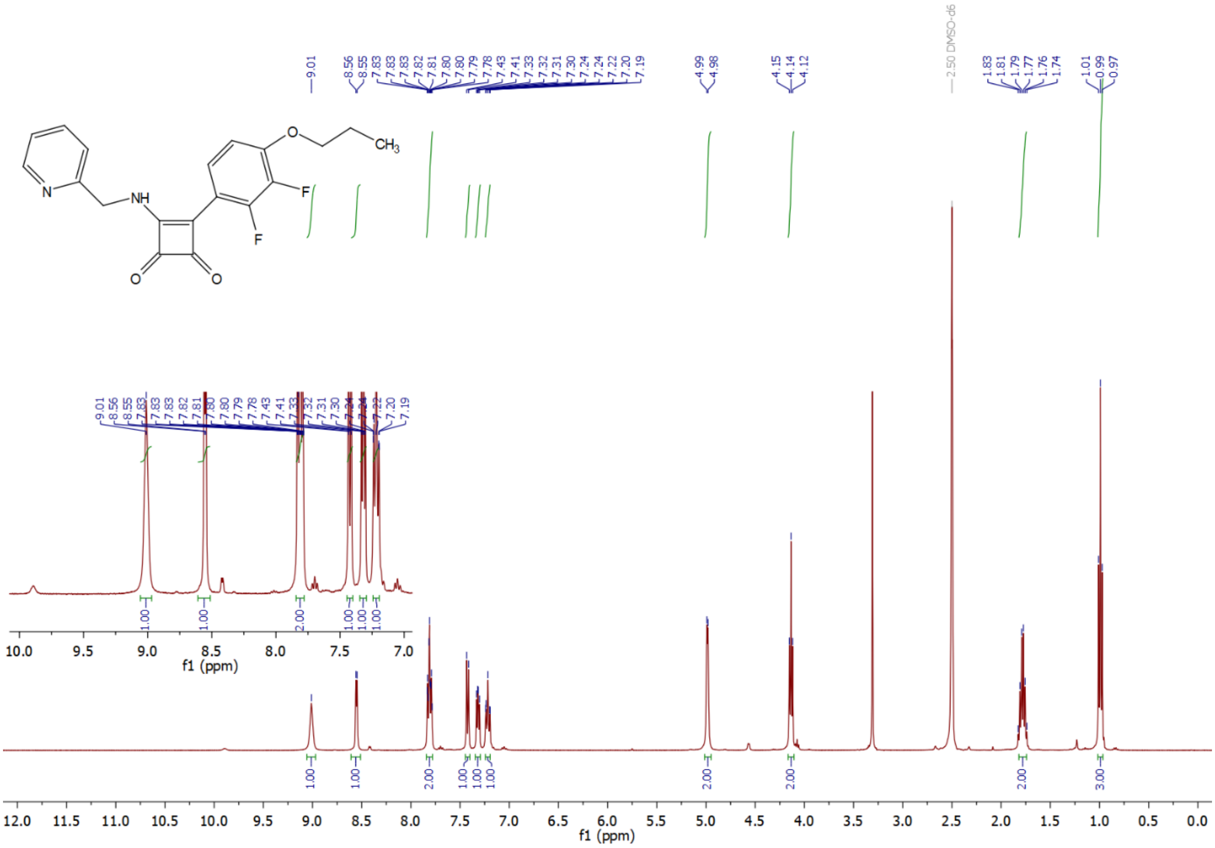


**Fig S73.** $^{13}$C NMR spectra of **6p**

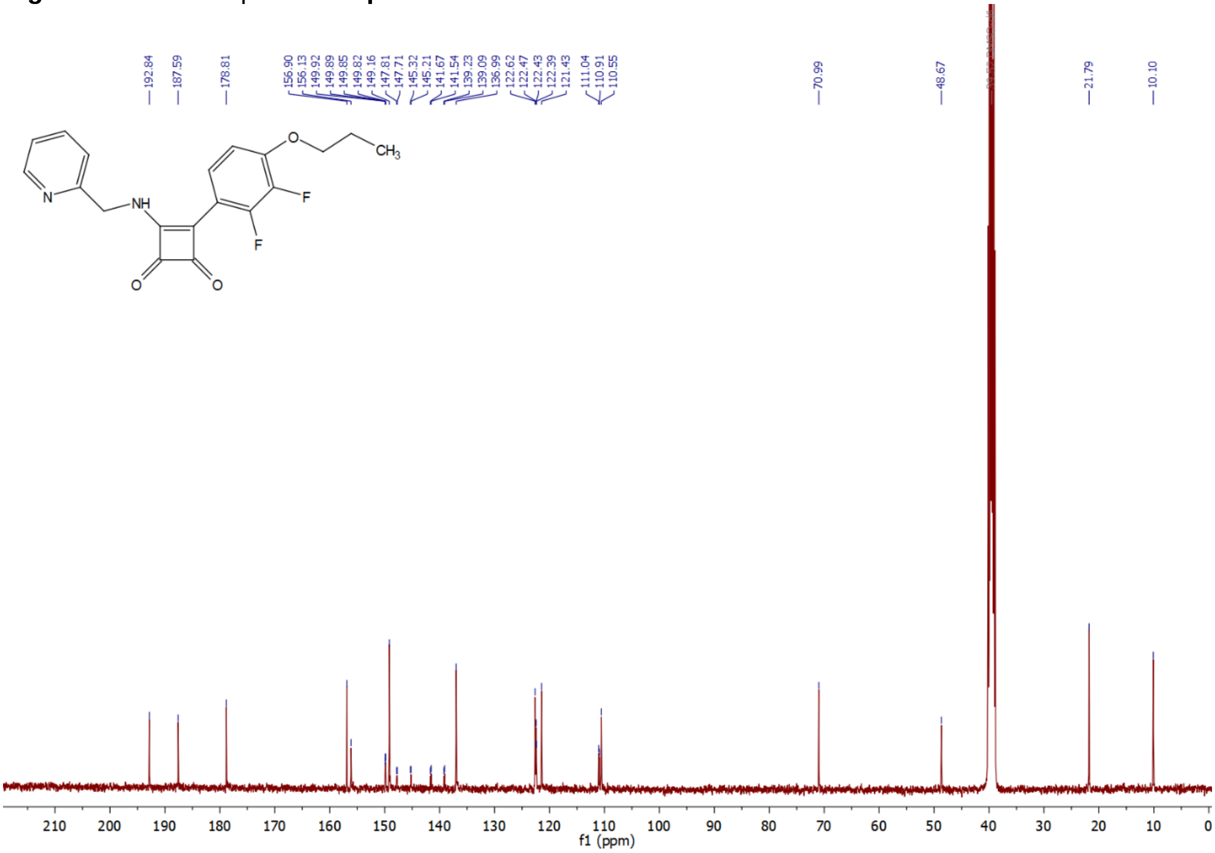

**Fig S74.** $^{1}H$ NMR spectra of **5q**

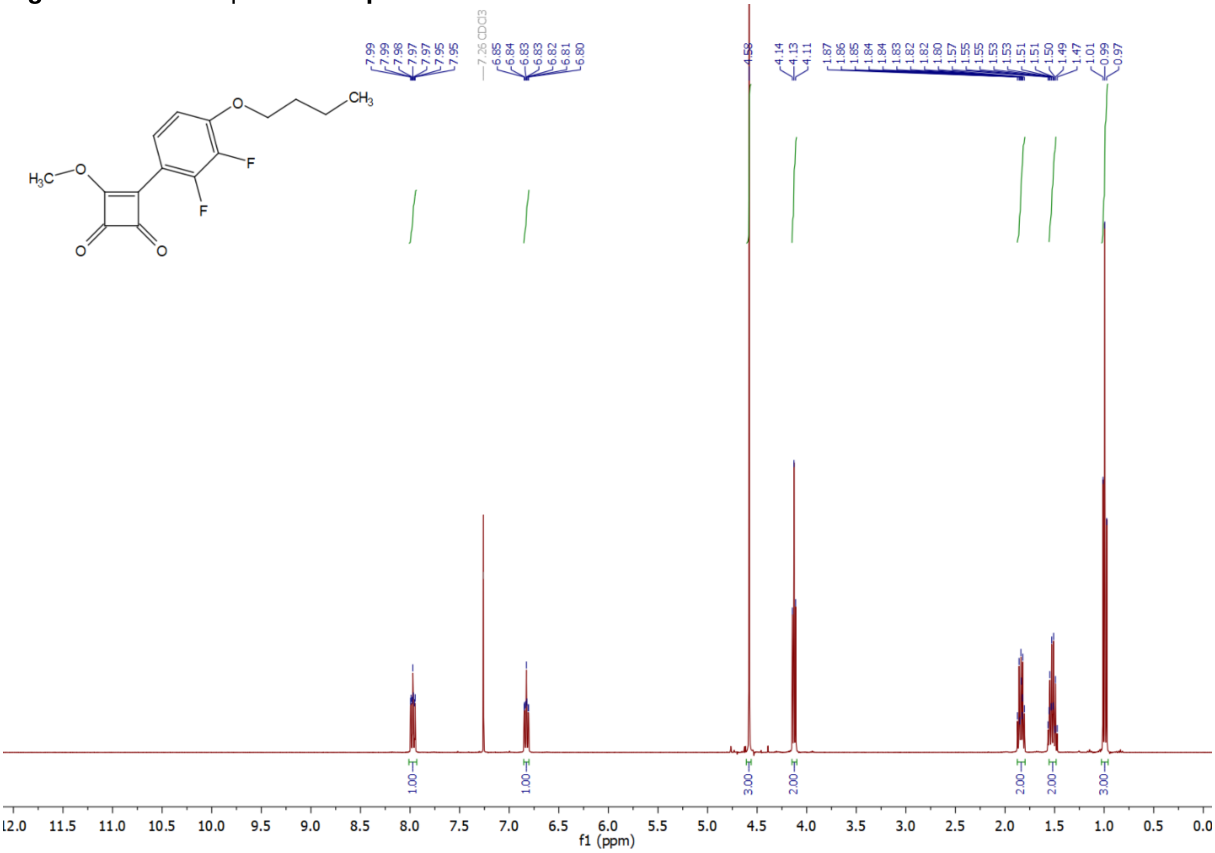

**Fig S75.** $^{13}C$ NMR spectra of **5q**

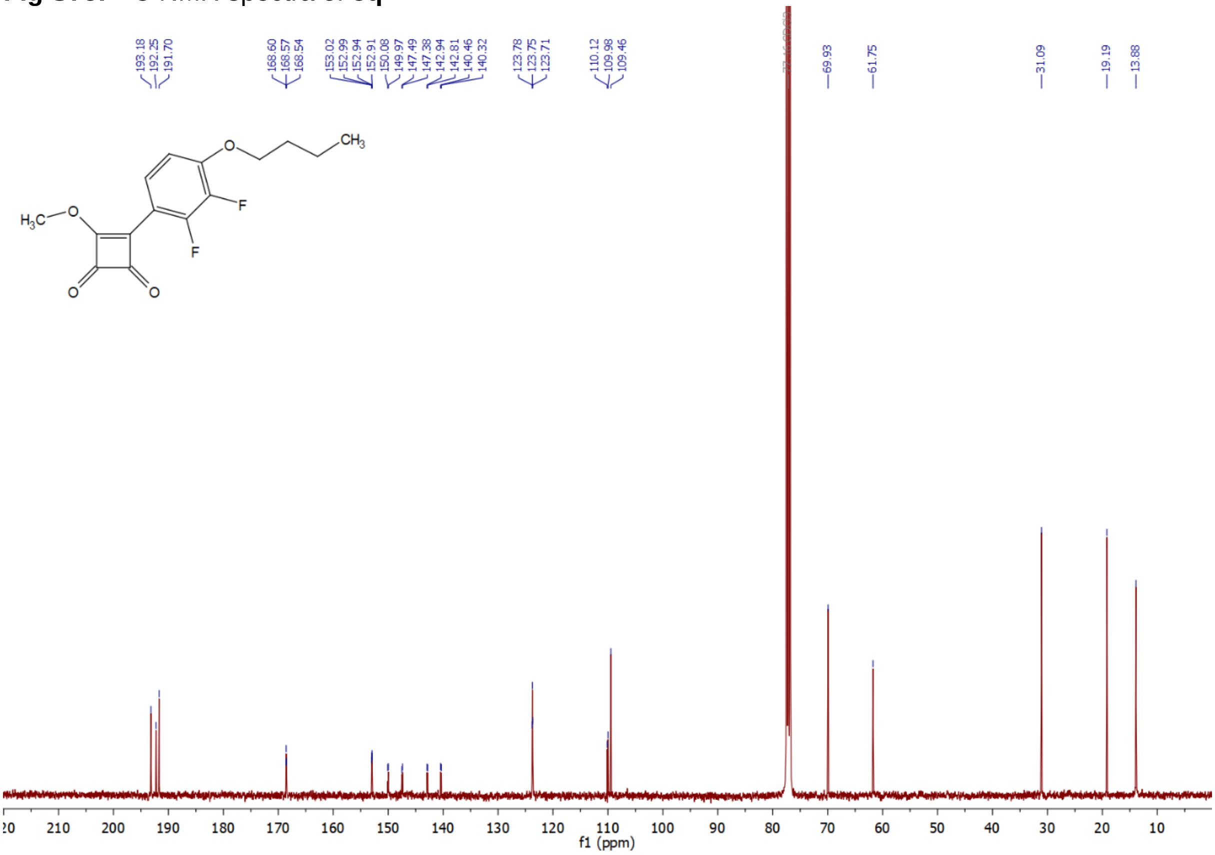

**Fig S76.** $^{1}H$ NMR spectra of **6q**

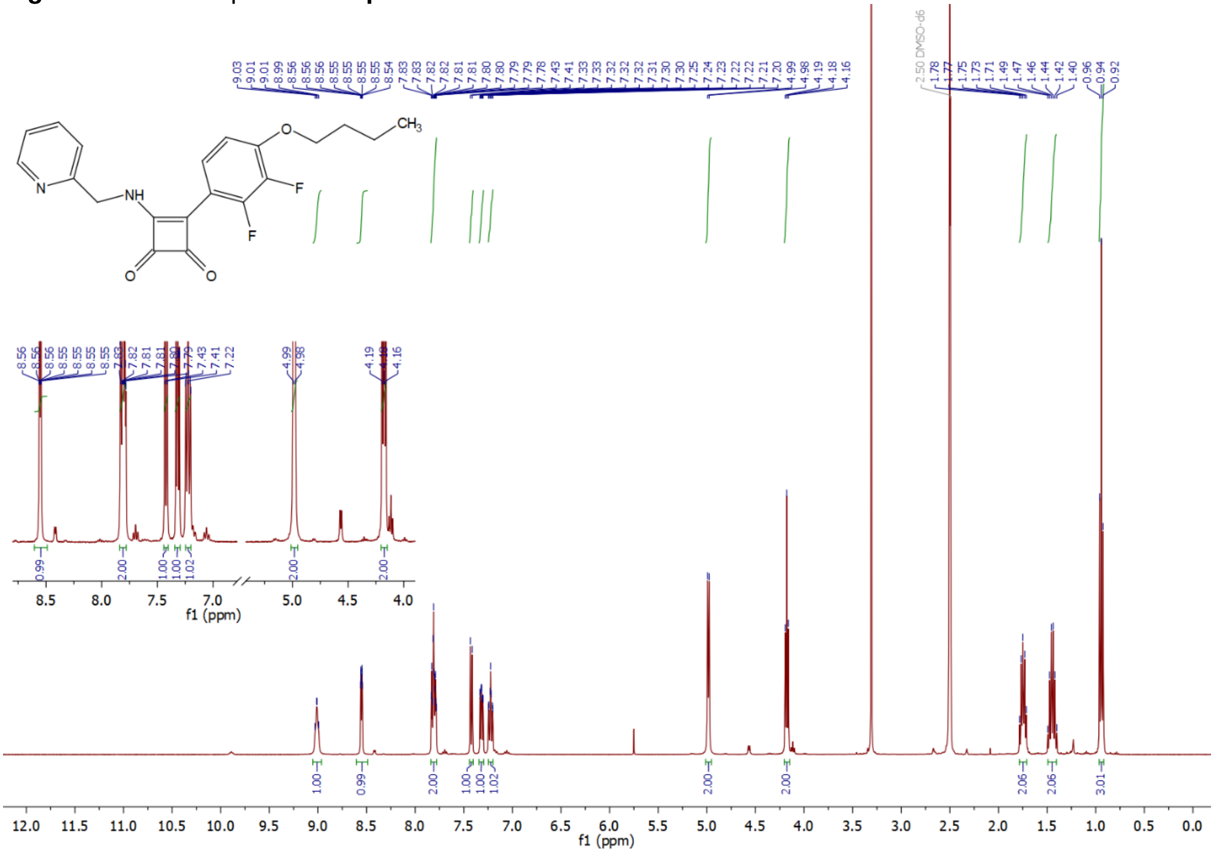


**Fig S77.** $^{13}C$ NMR spectra of **6q**

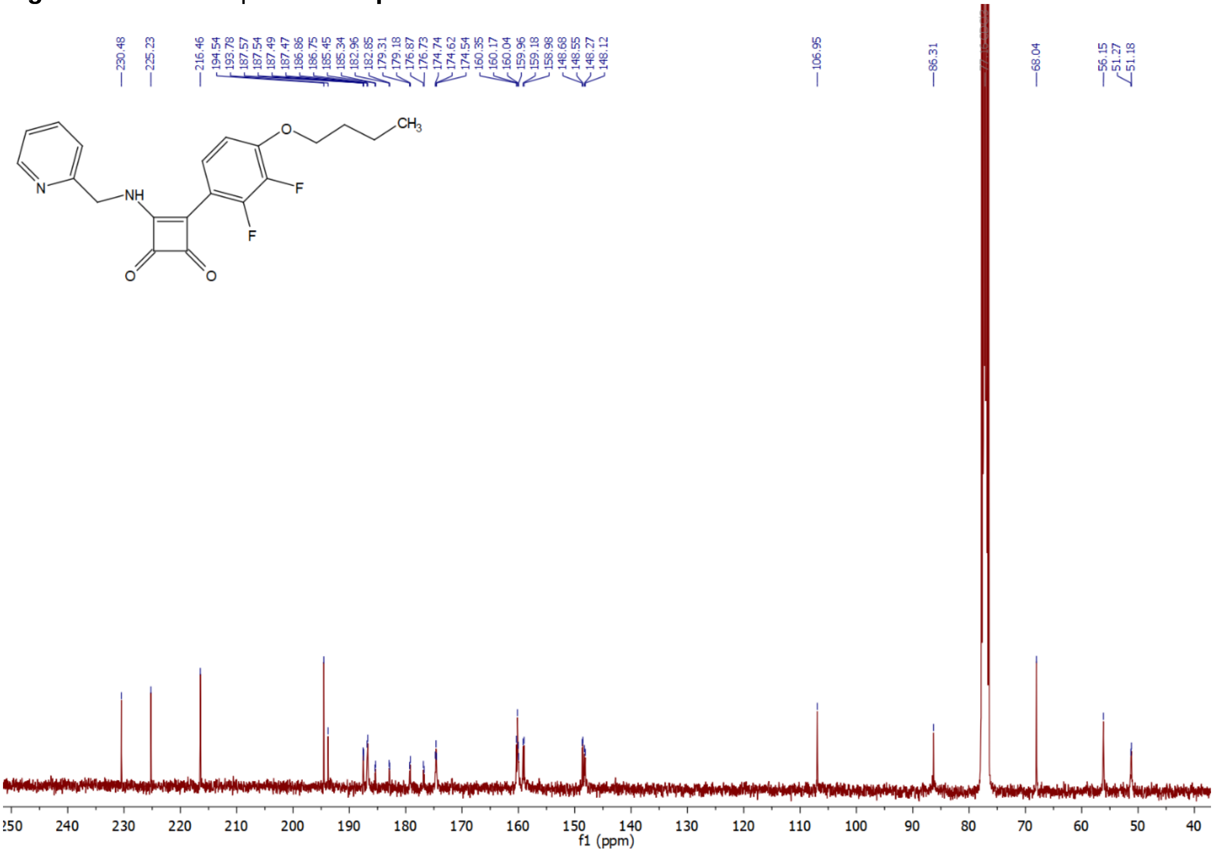

**Fig S78.** $^{1}H$ NMR spectra of **5r**

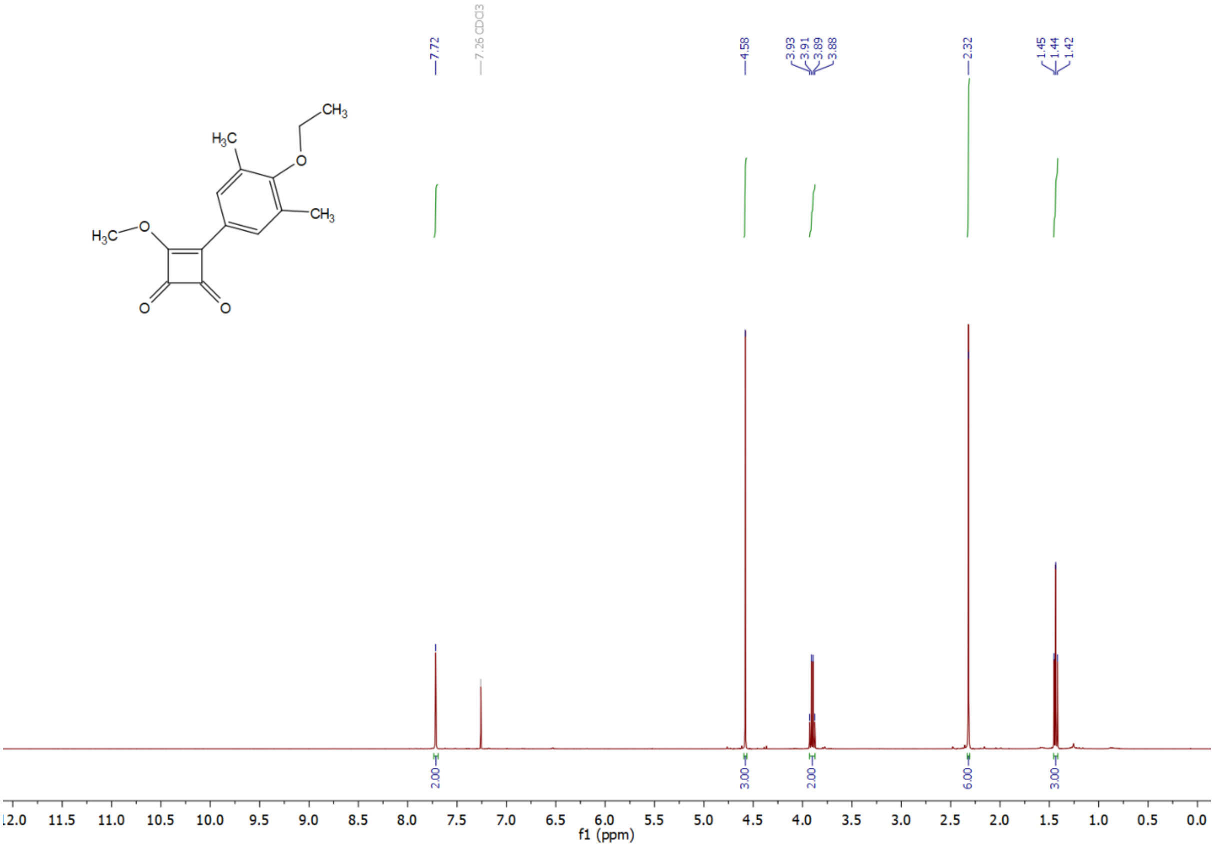


**Fig S79.** $^{13}C$ NMR spectra of **5r**

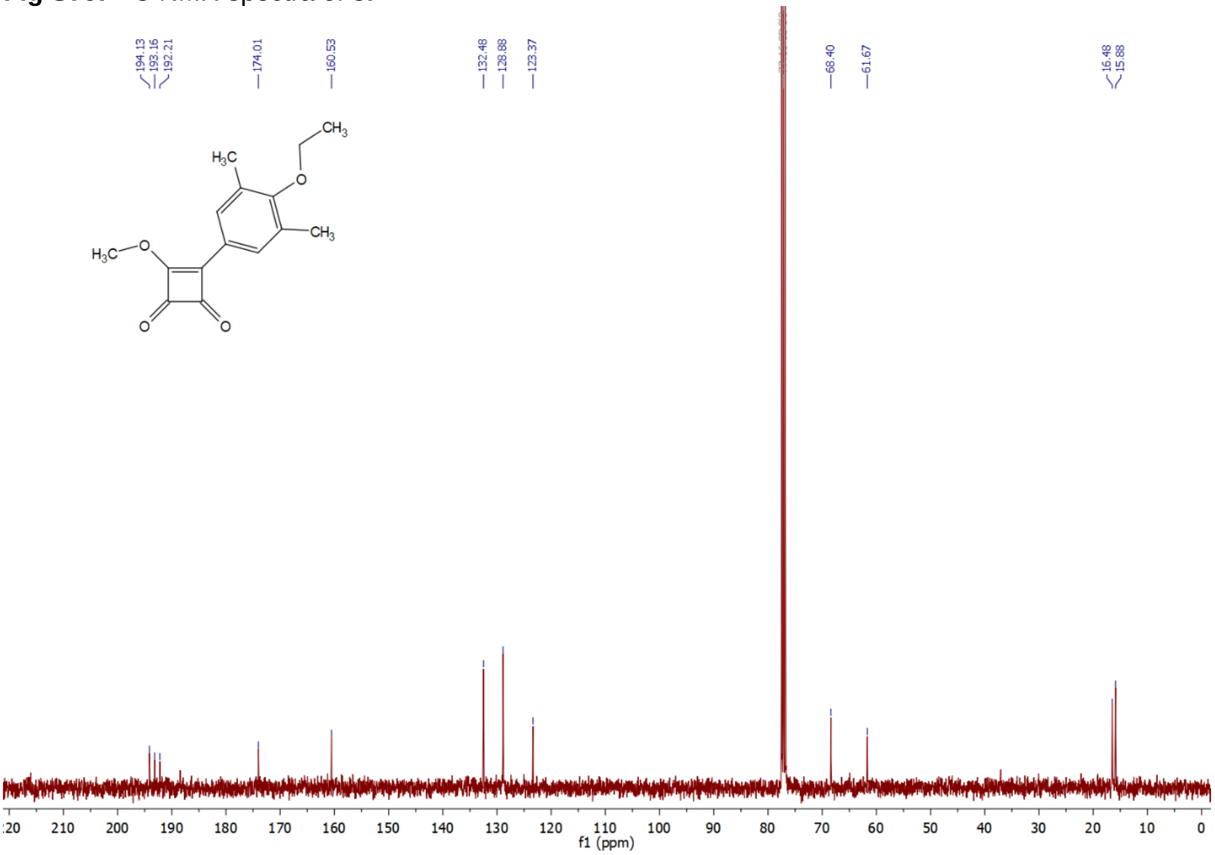

**Fig S80.** $^{1}H$ NMR spectra of **6r**

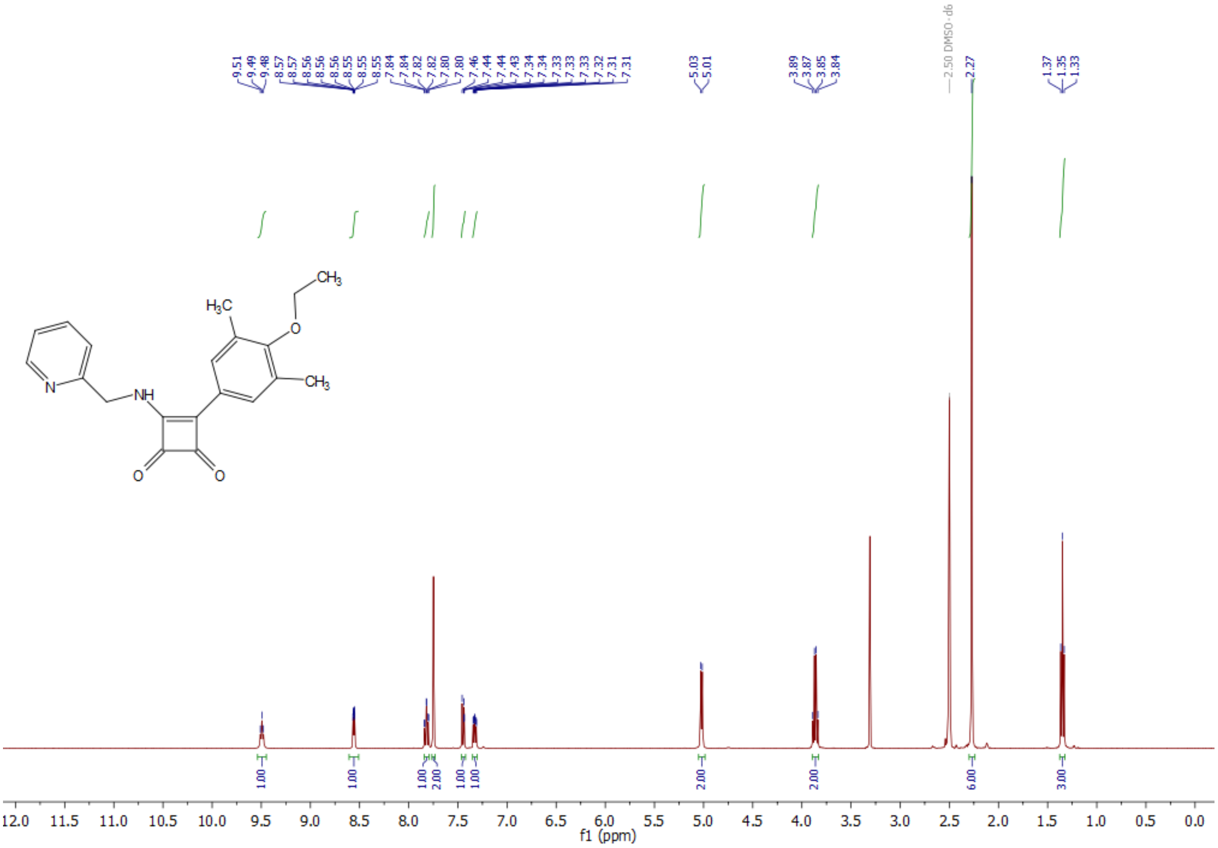

**Fig S81.** $^{13}C$ NMR spectra of **6r**

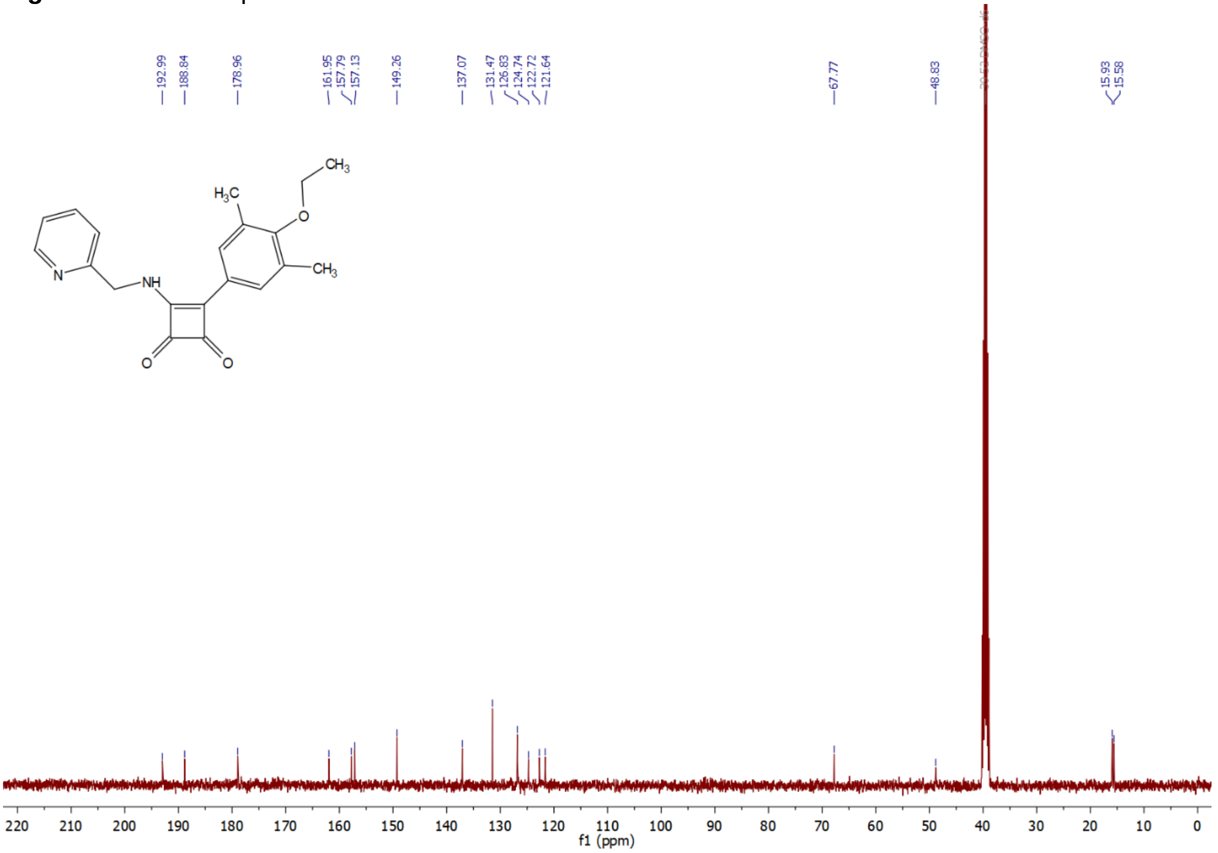

**Fig S82.** $^{1}$H NMR spectra of **5s**

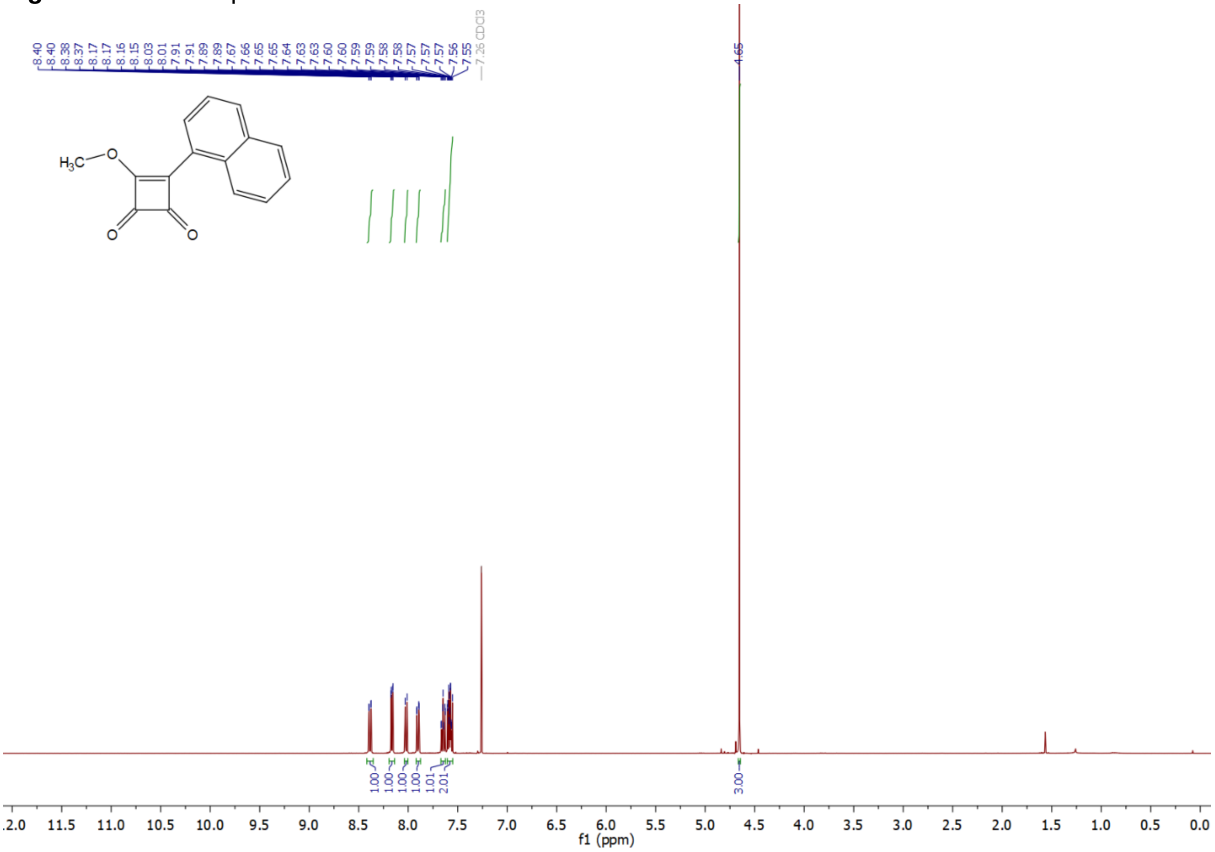

**Fig S83.** $^{13}$C NMR spectra of **5s**

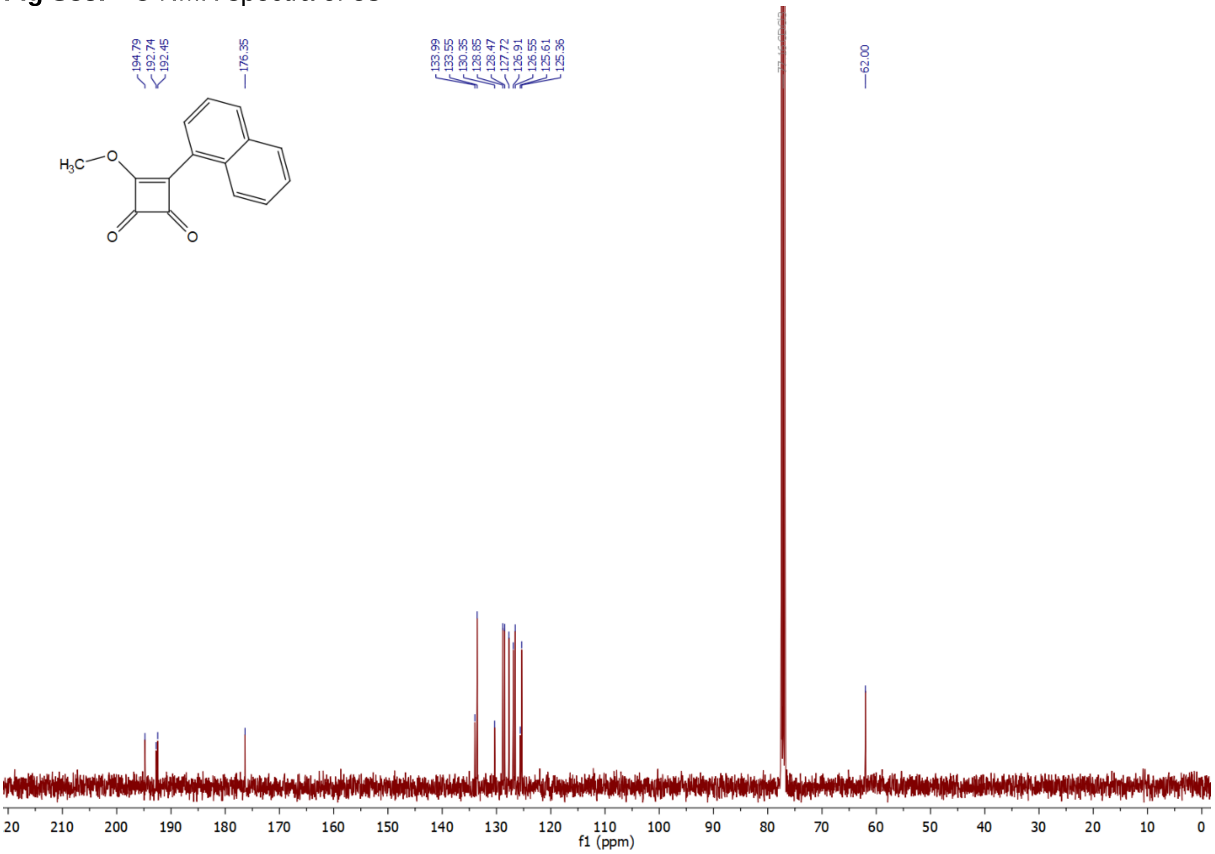

**Fig S84.** $^{1}$H NMR spectra of **6s** at 25 °C

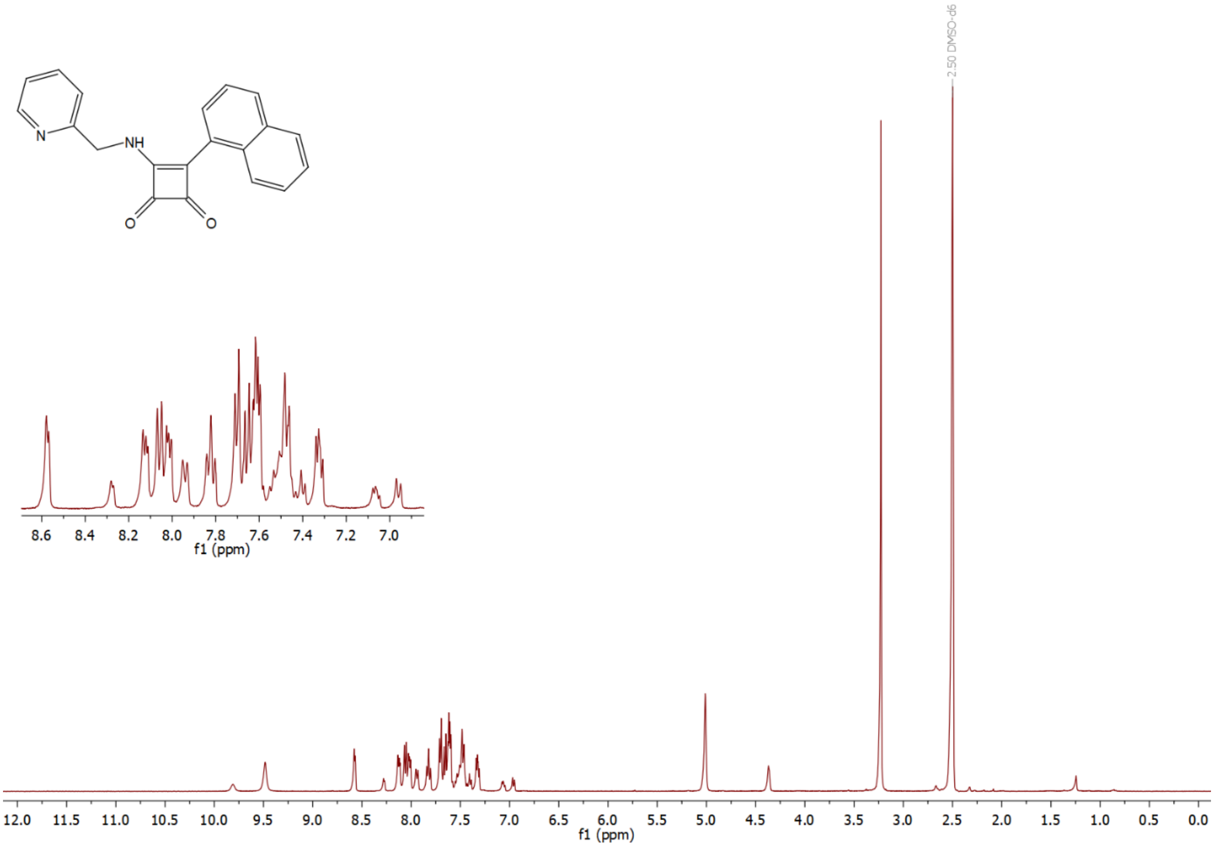


**Fig S85.** $^{1}$H NMR spectra of **6s** at 45 °C

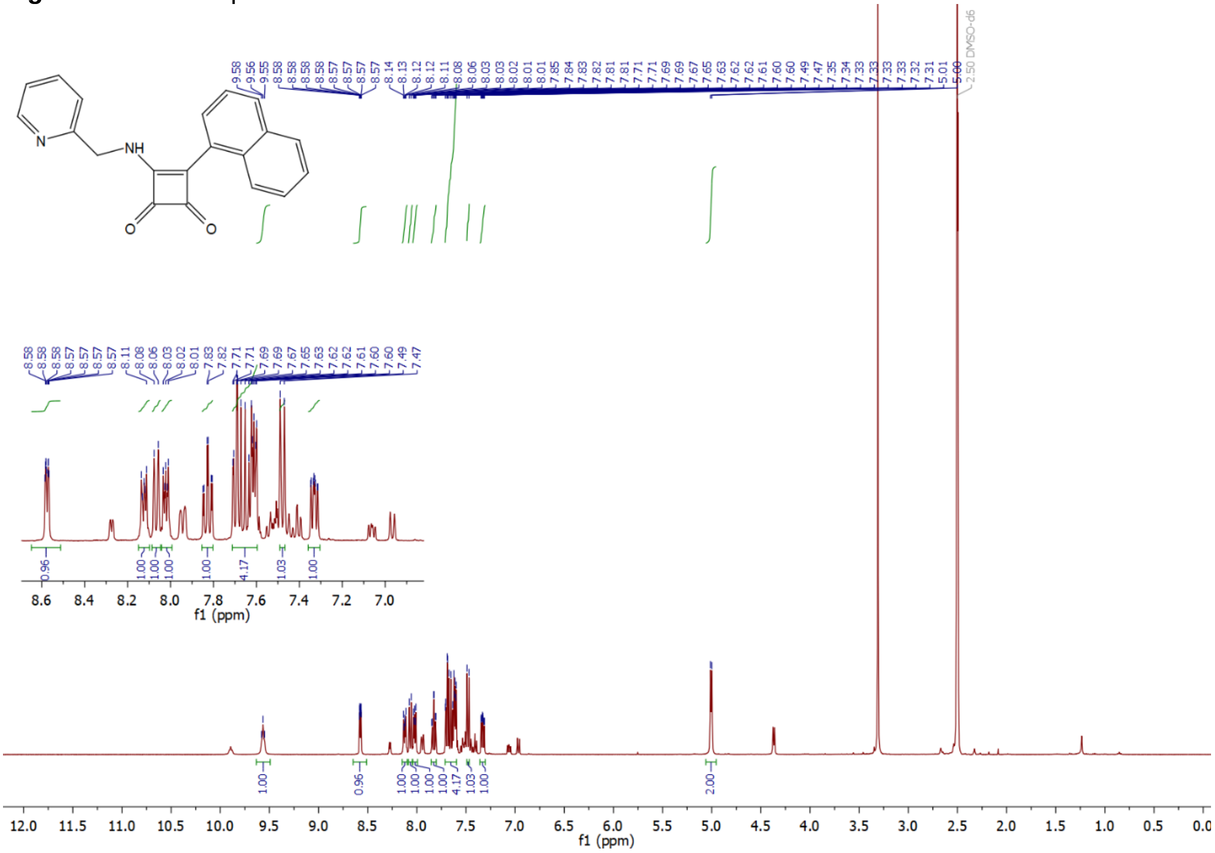

**Fig S86.** $^{1}H$ NMR spectra of **6s** at 65 °C

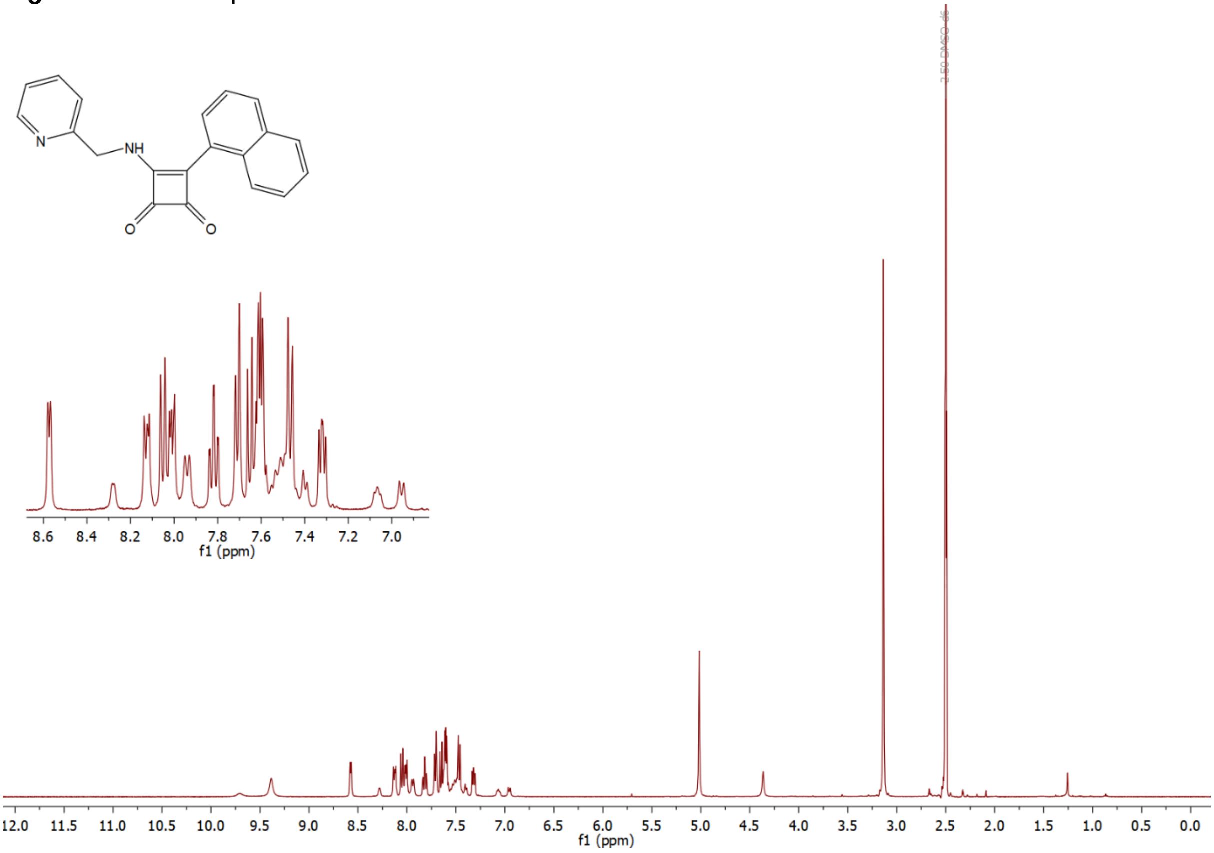


**Fig S87.** $^{1}H$ NMR spectra of **6s** at 85 °C

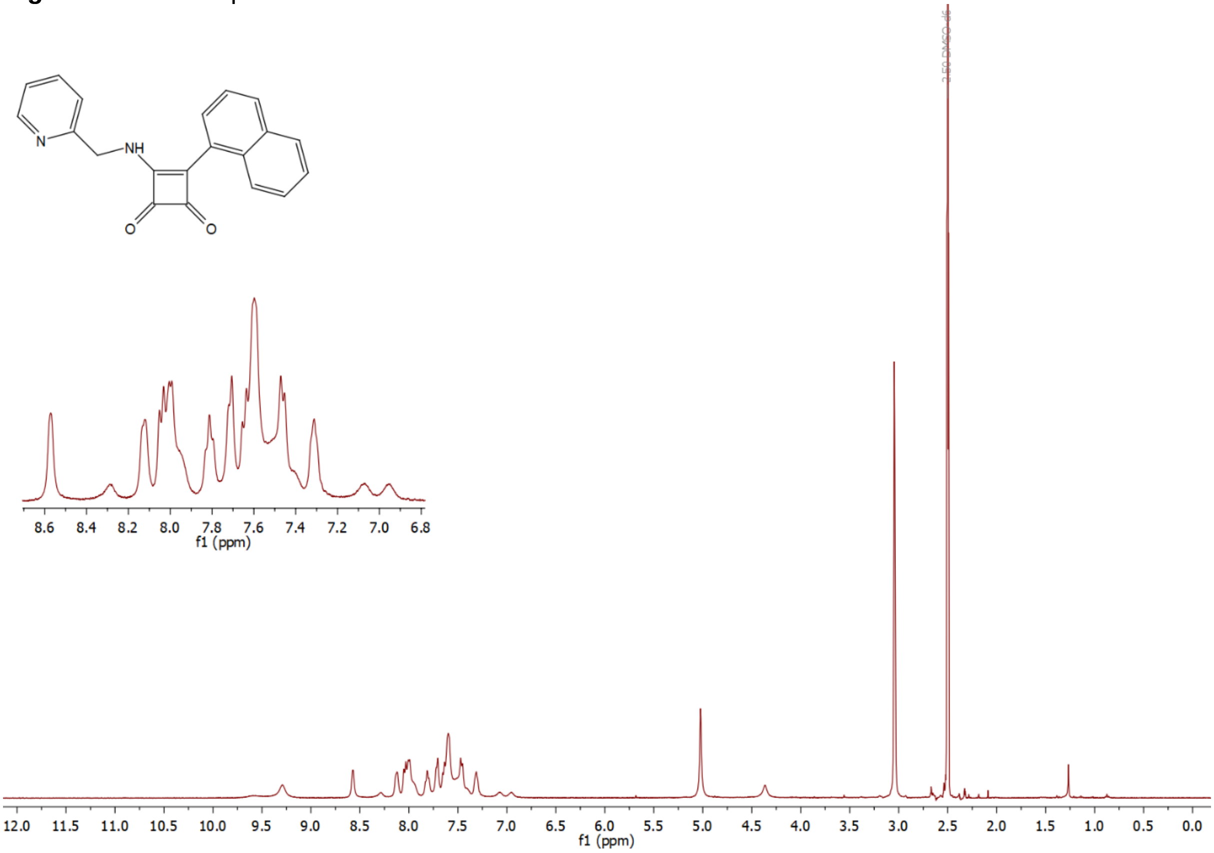

**Fig S88.** $^{1}$H NMR spectra of **6s** at 105 °C

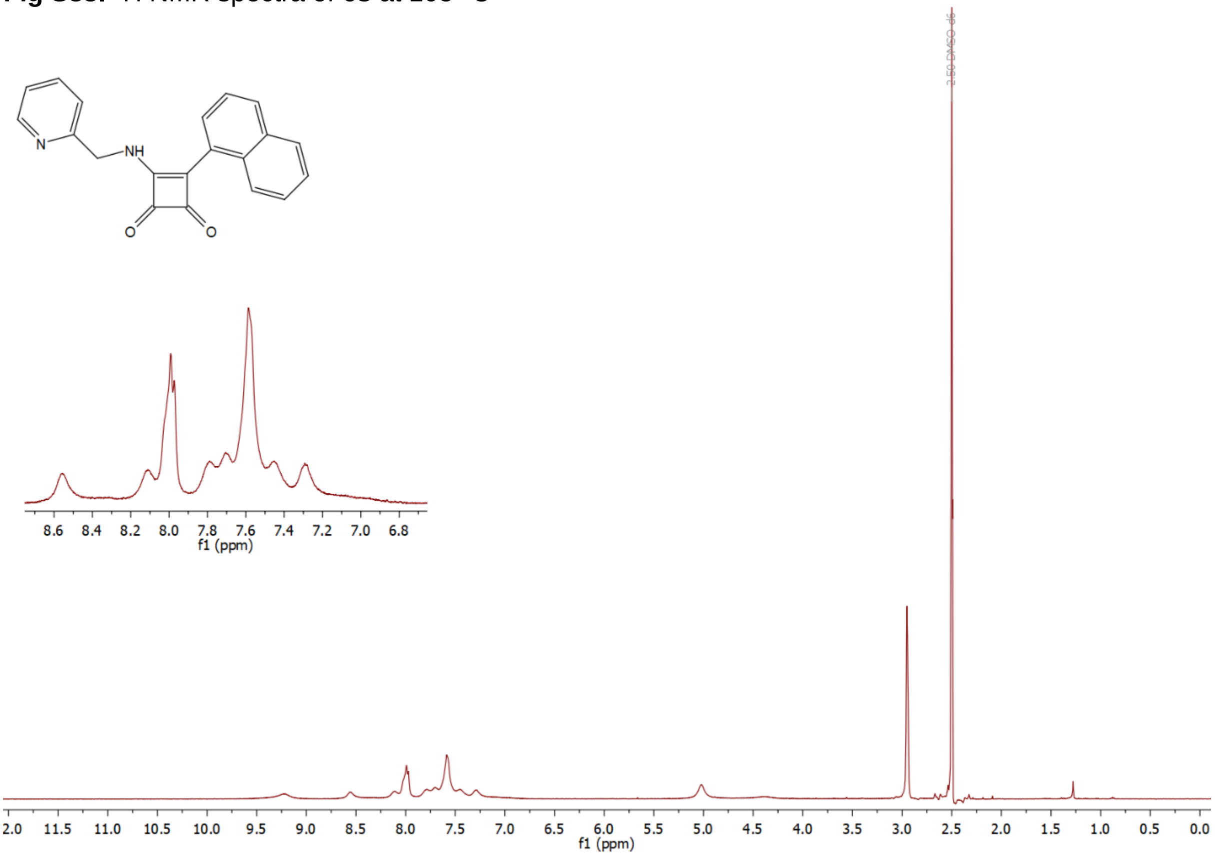


**Fig S89.** $^{13}$C NMR spectra of **6s**

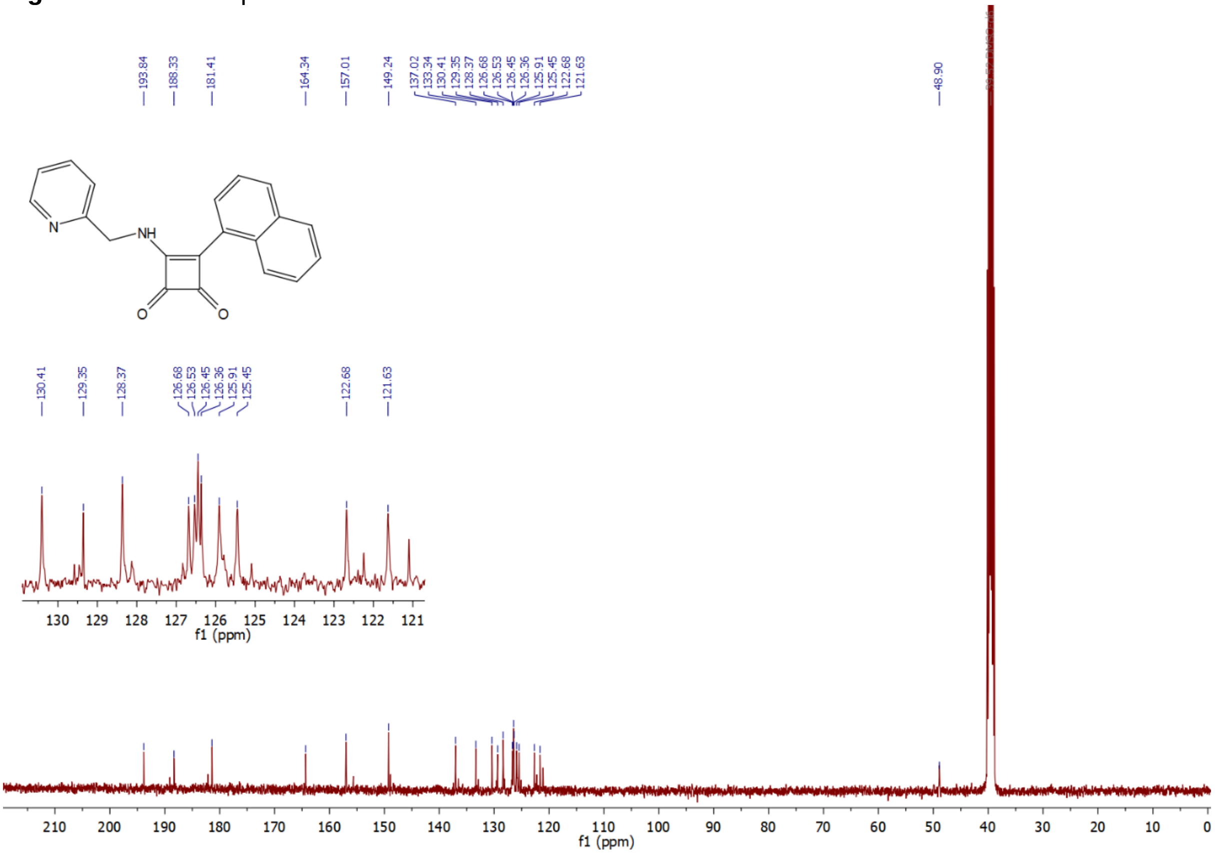

**Fig S90.** $^{1}$H NMR spectra of **5t**

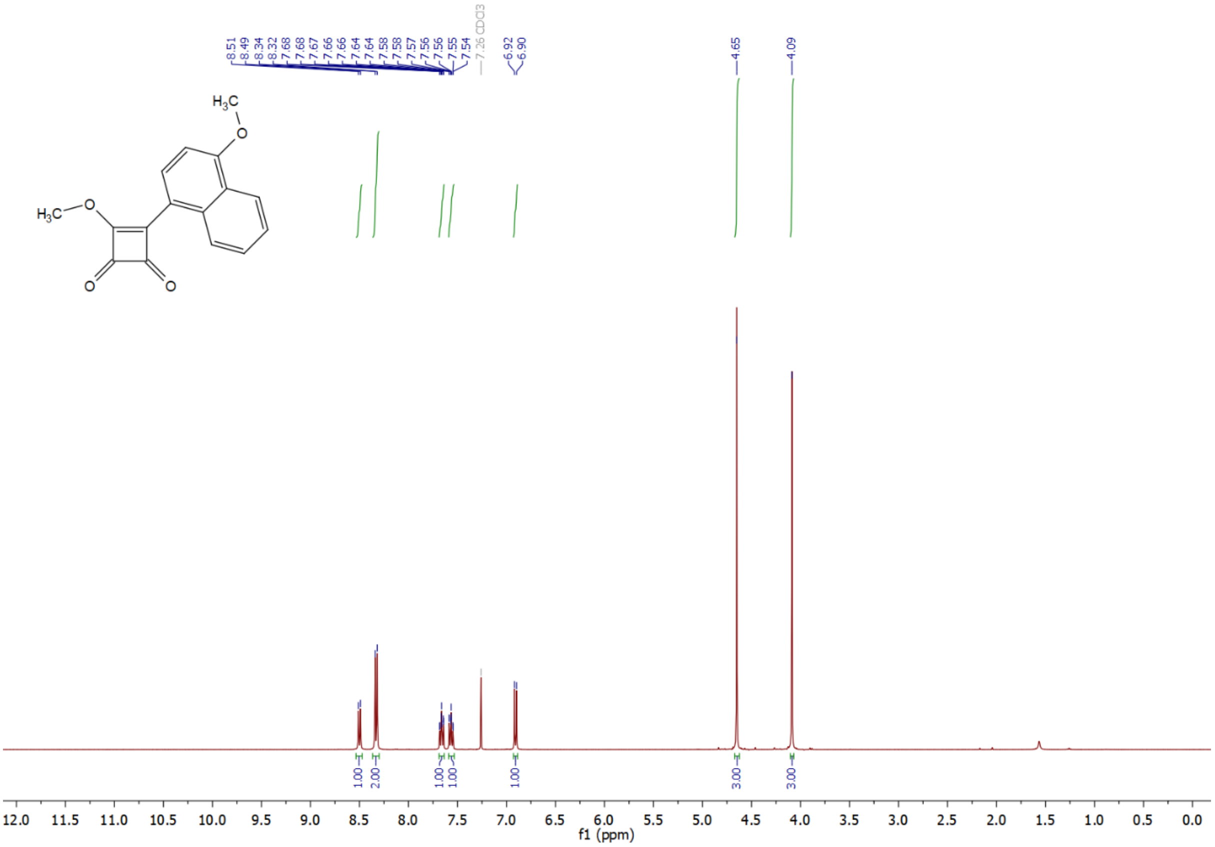


**Fig S91.** $^{13}$C NMR spectra of **5t**

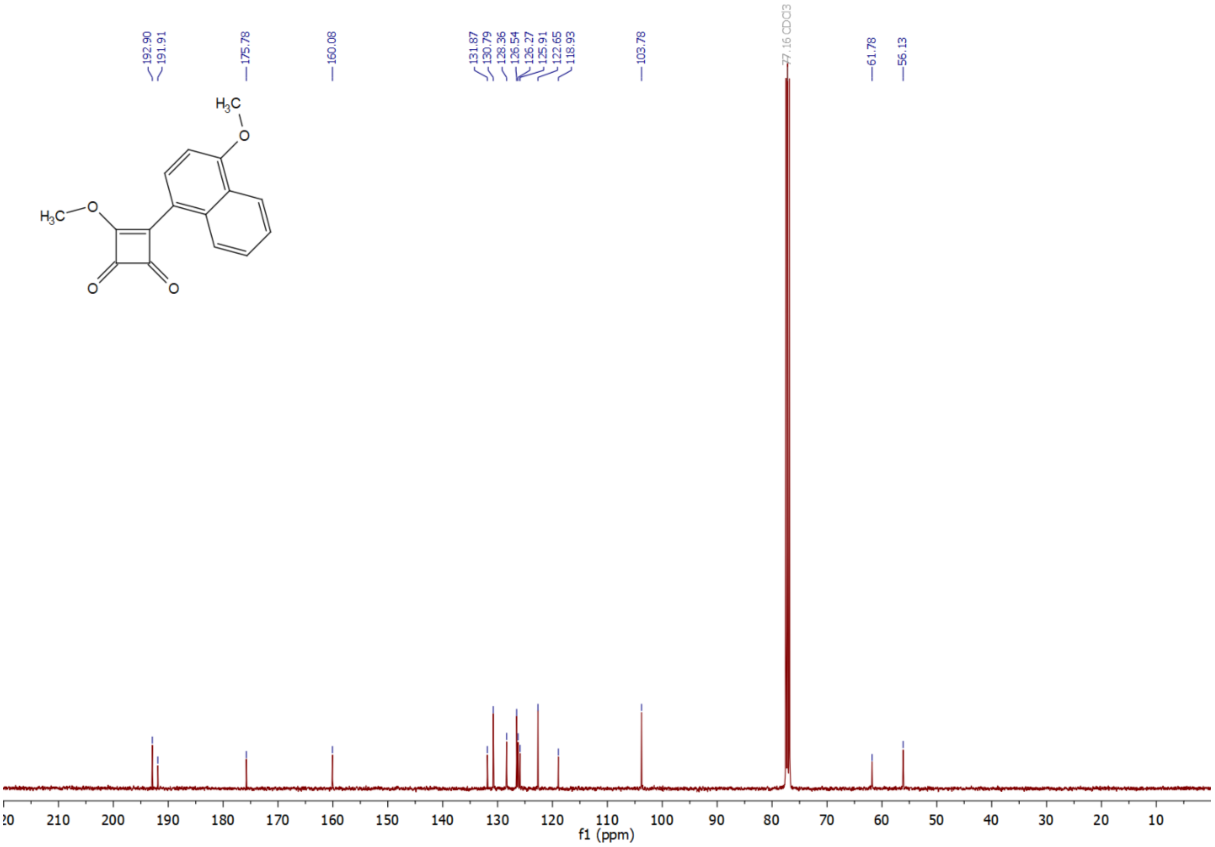

**Fig S92.** $^{1}$H NMR spectra of **6t**

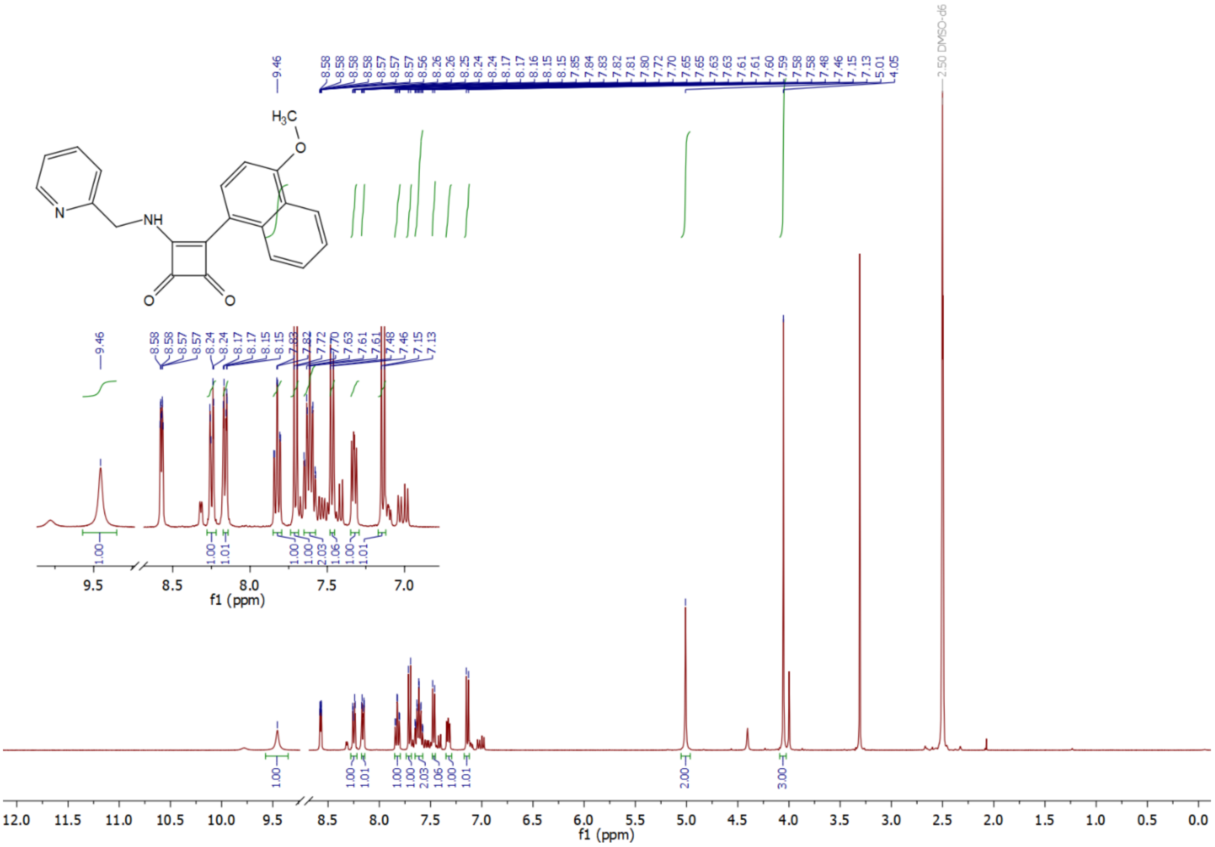

**Fig S93.** $^{13}$C NMR spectra of **6t**

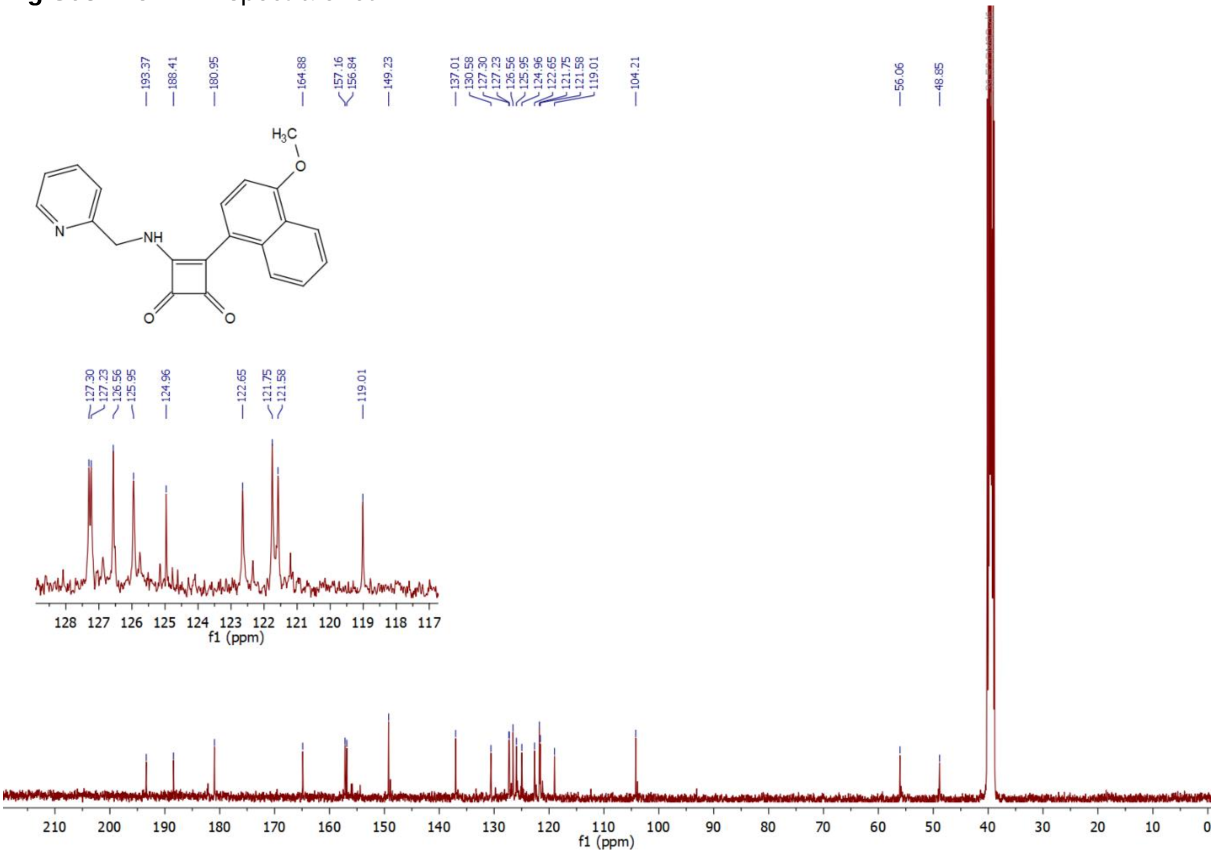

**Fig S94.** $^{1}$H NMR spectra of **5u**

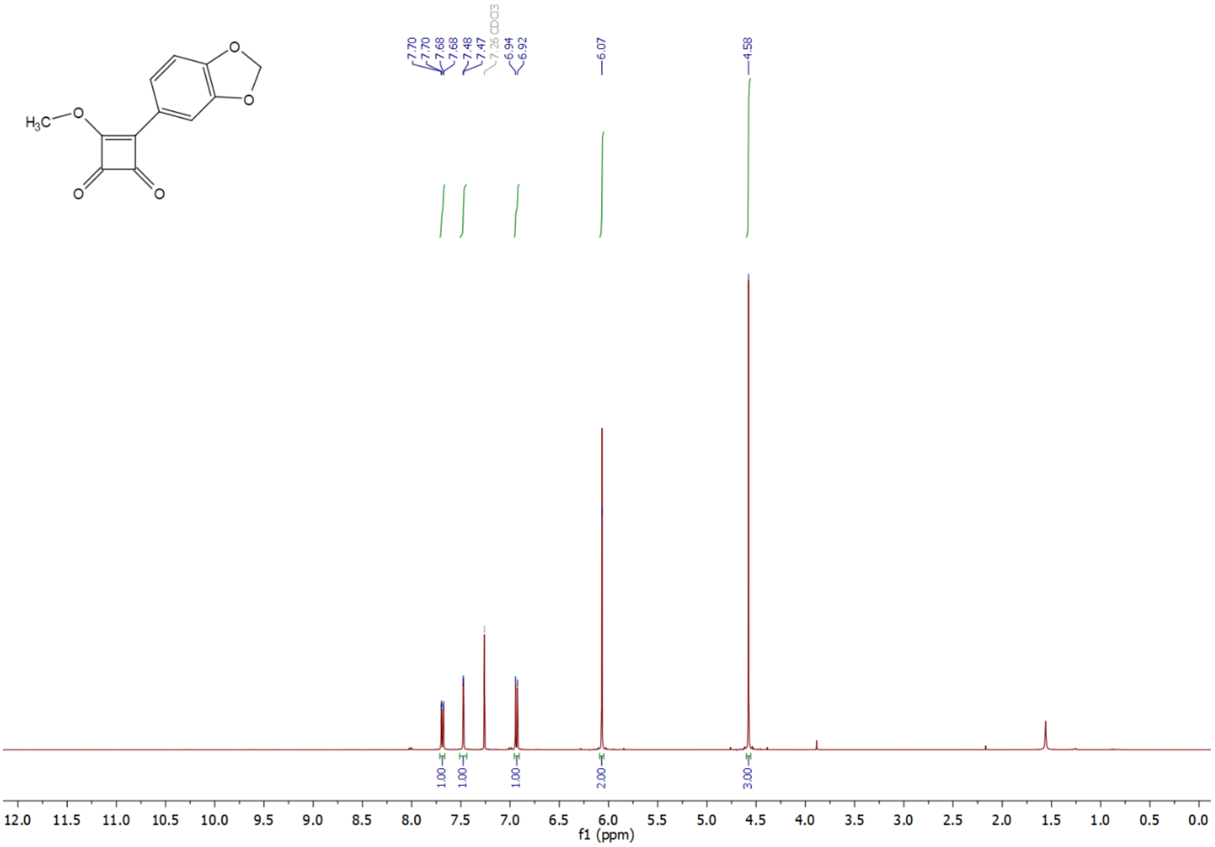


**Fig S95.** $^{13}$C NMR spectra of **5u**

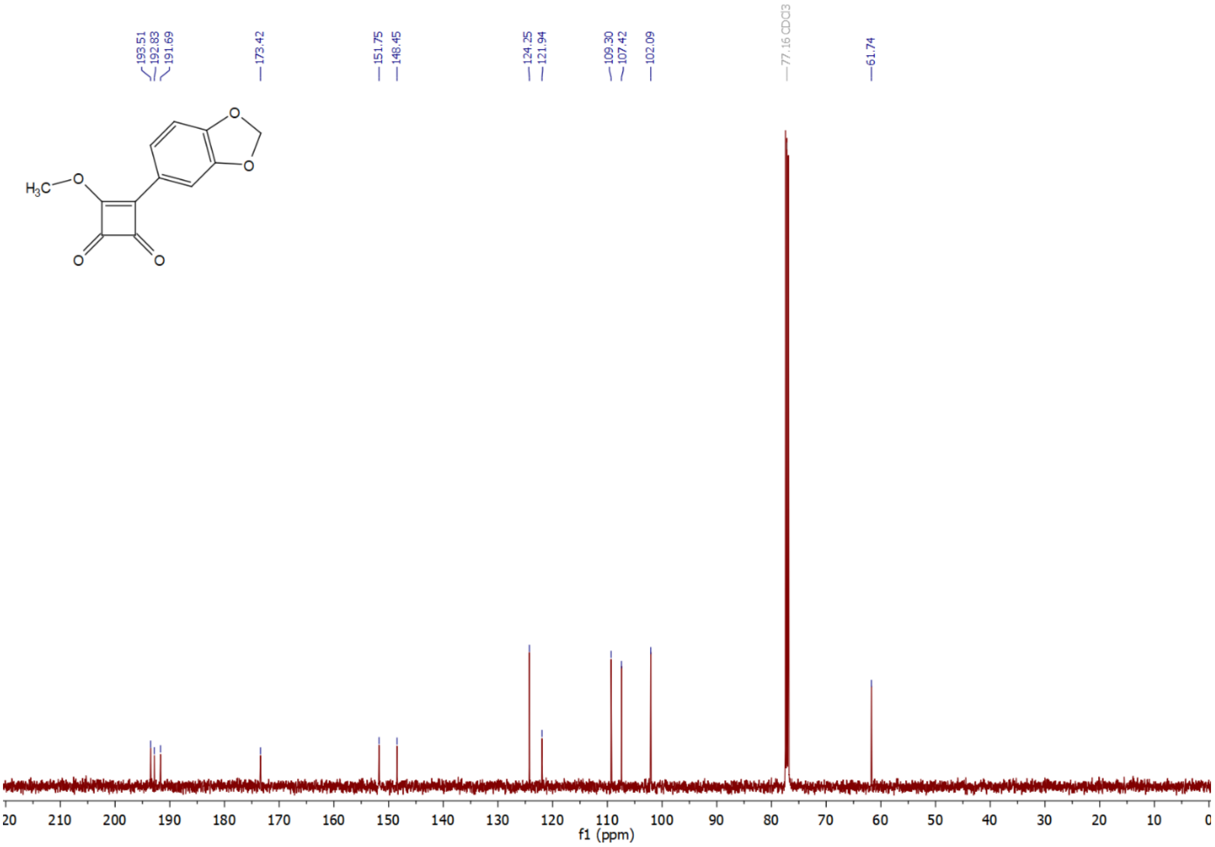

**Fig S96.** $^{1}$H NMR spectra of **6u**

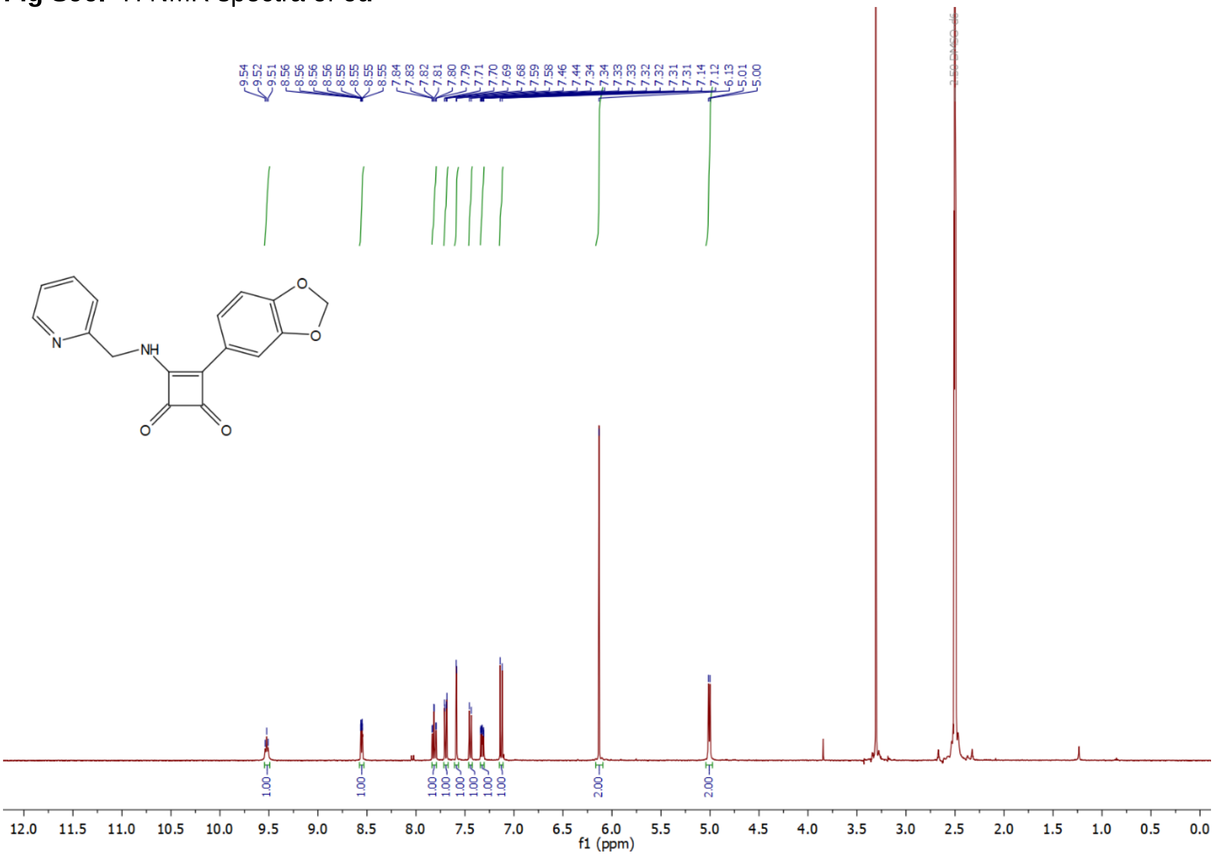


**Fig S97.** $^{13}$C NMR spectra of **6u**

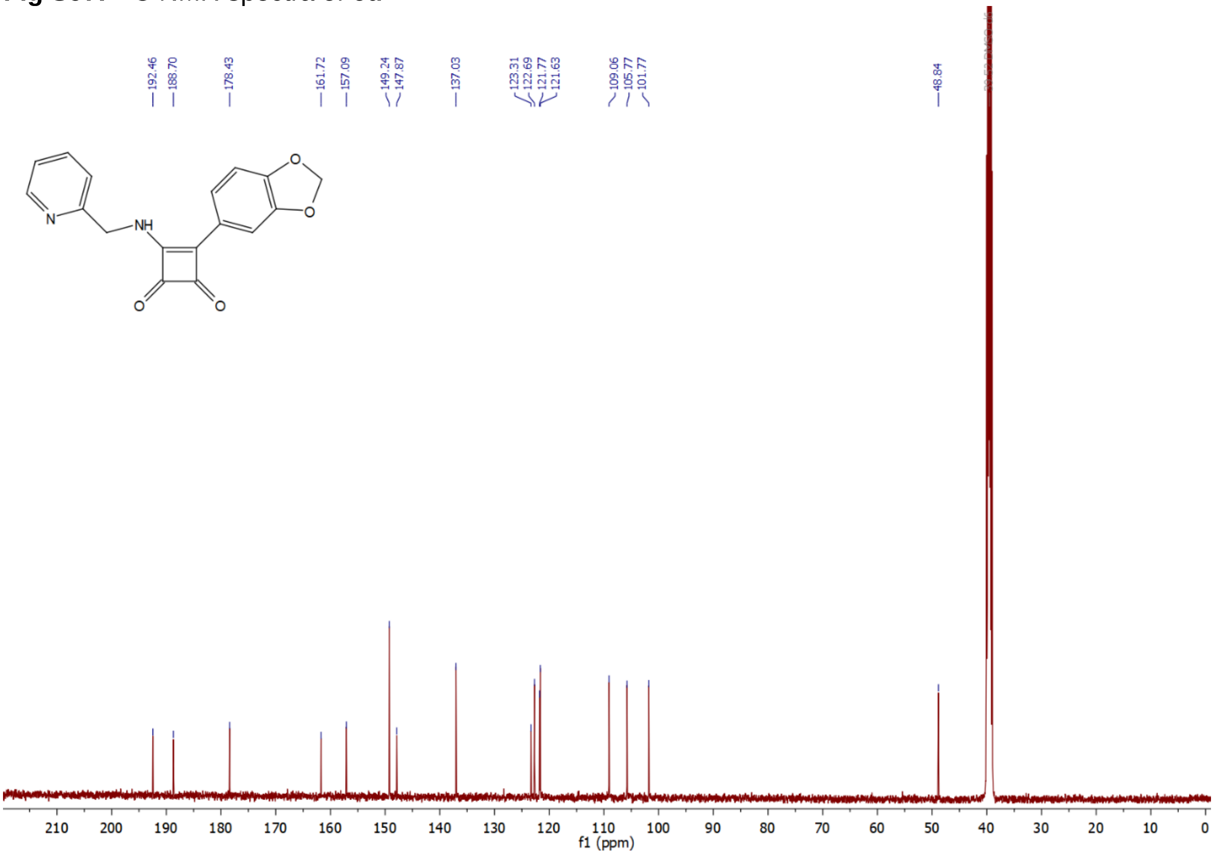

**Fig S98.** $^{1}$H NMR spectra of **5v**

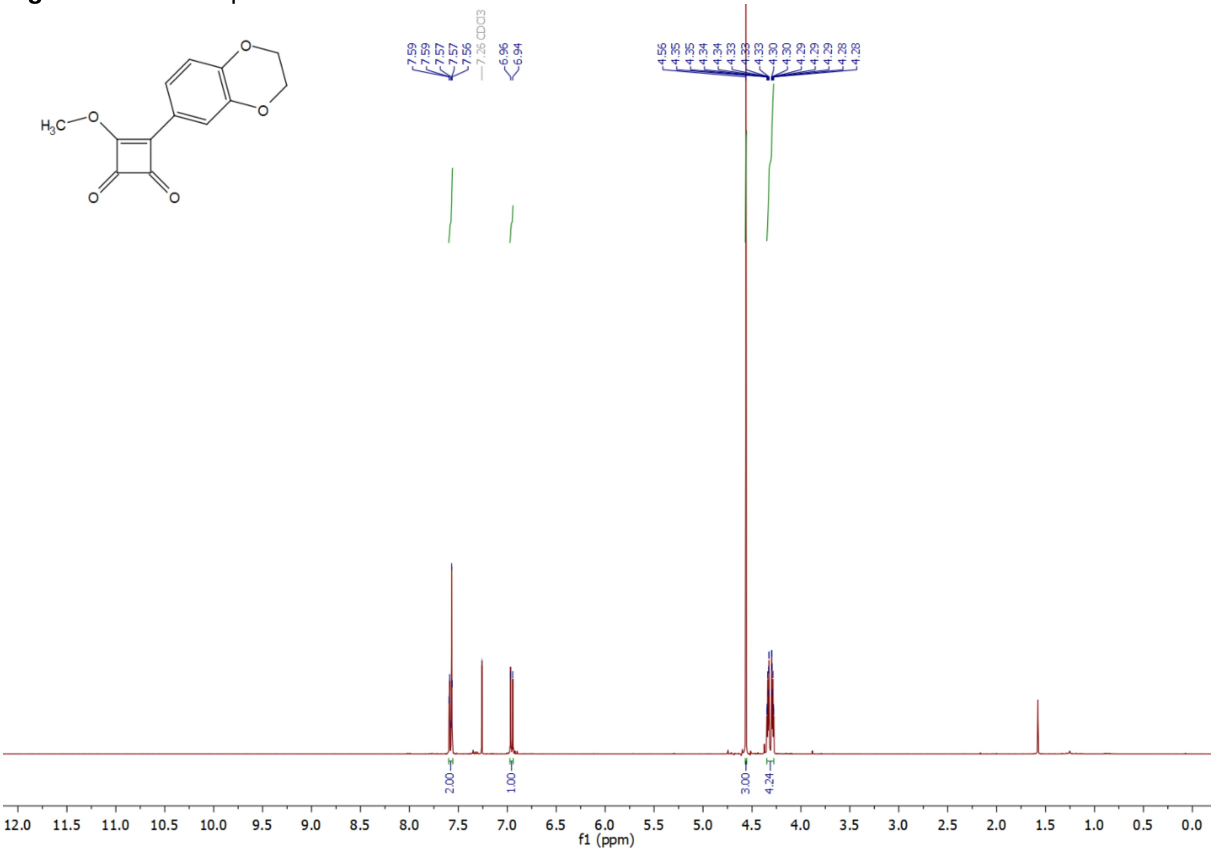


**Fig S99.** $^{13}$C NMR spectra of **5v**

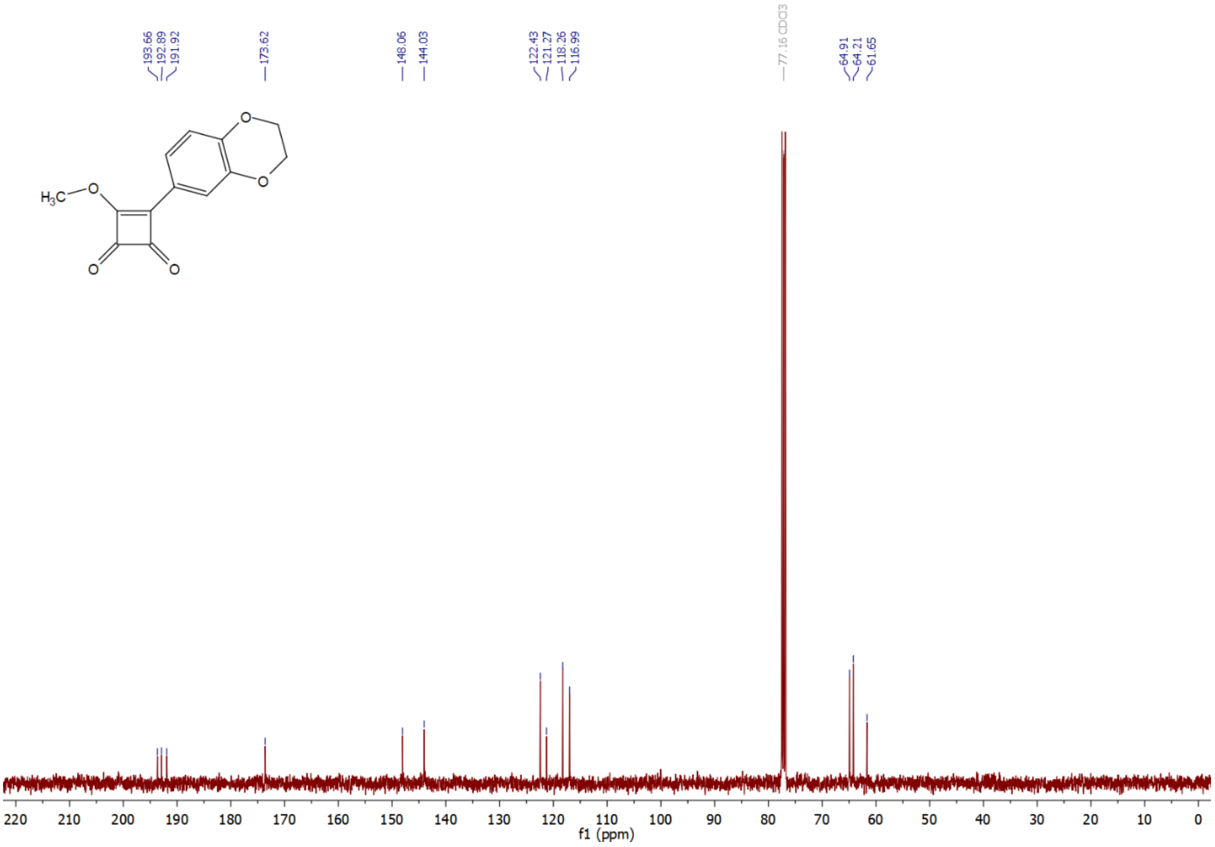

**Fig S100.** $^{1}H$ NMR spectra of **6v**

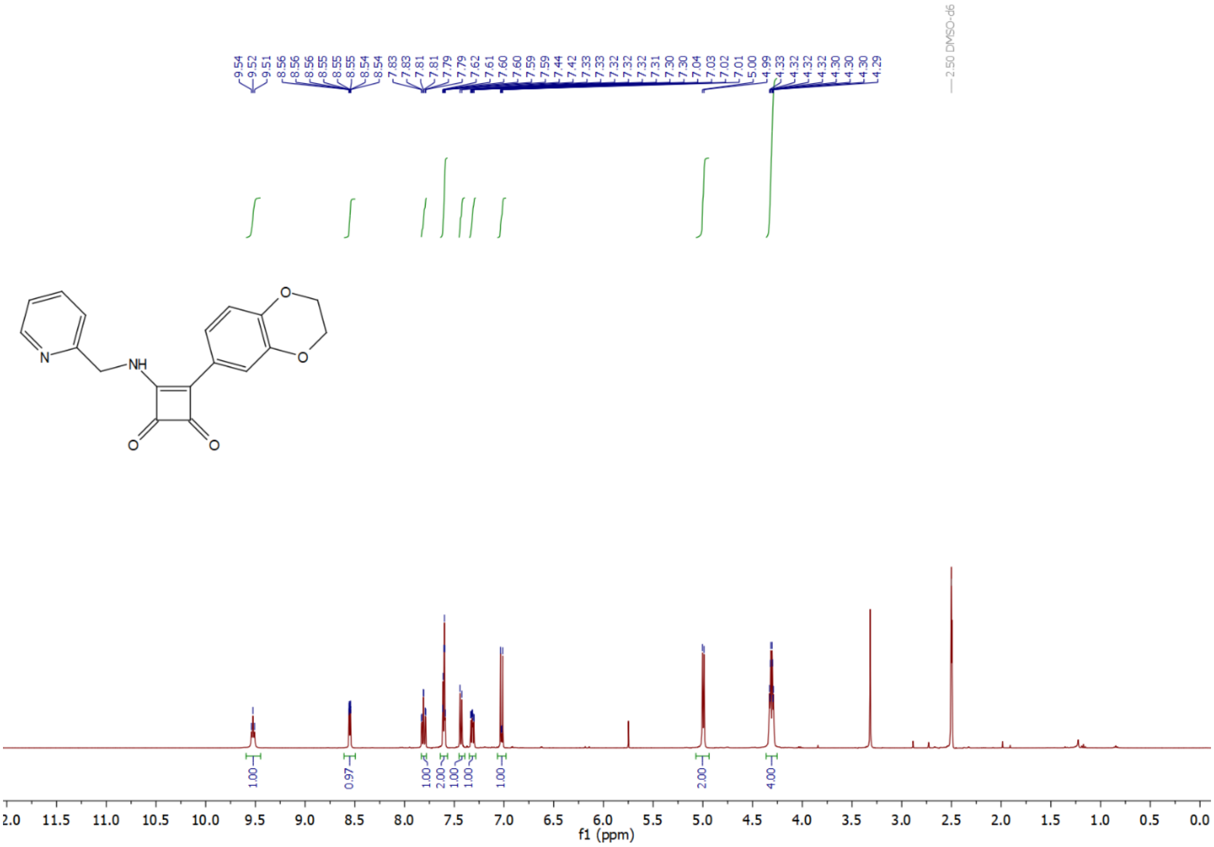


**Fig S101.** $^{13}C$ NMR spectra of **6v**

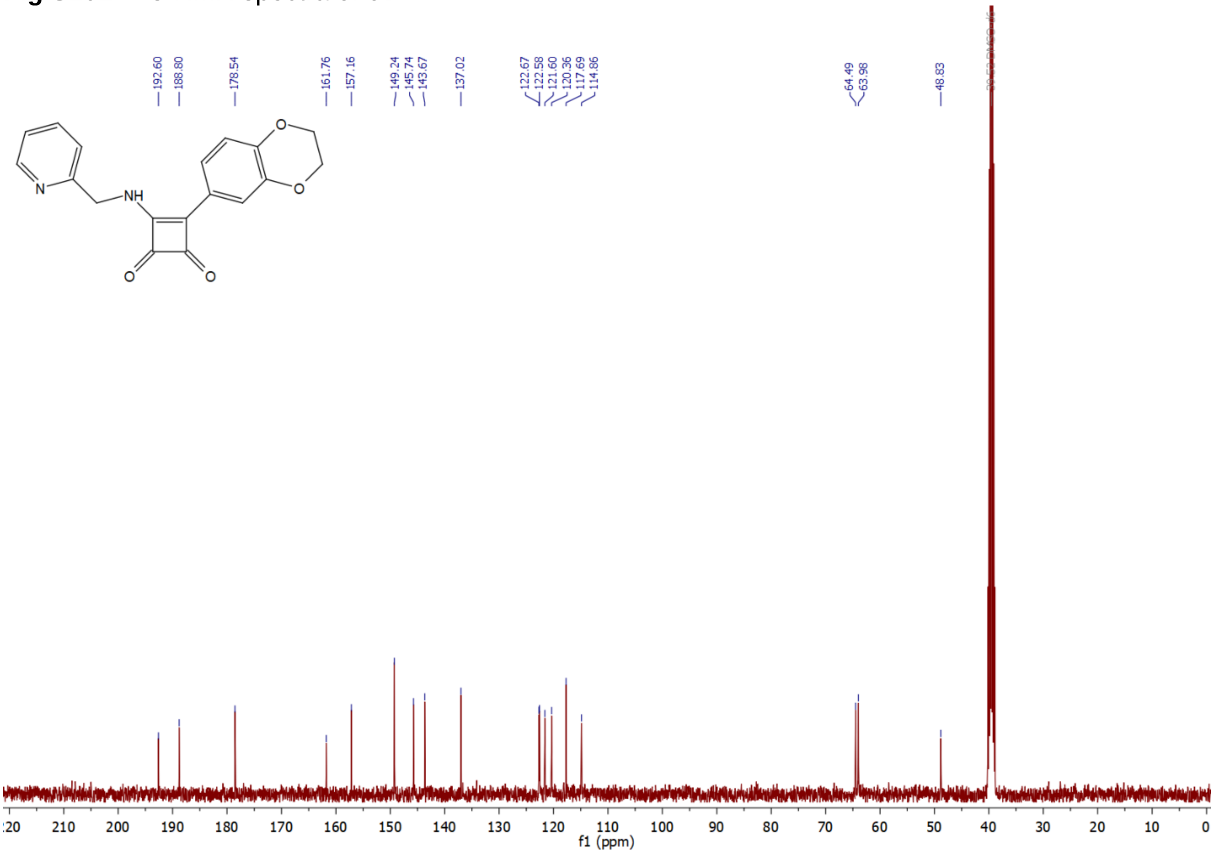

**Fig S102.** $^{1}$H NMR spectra of **5w**

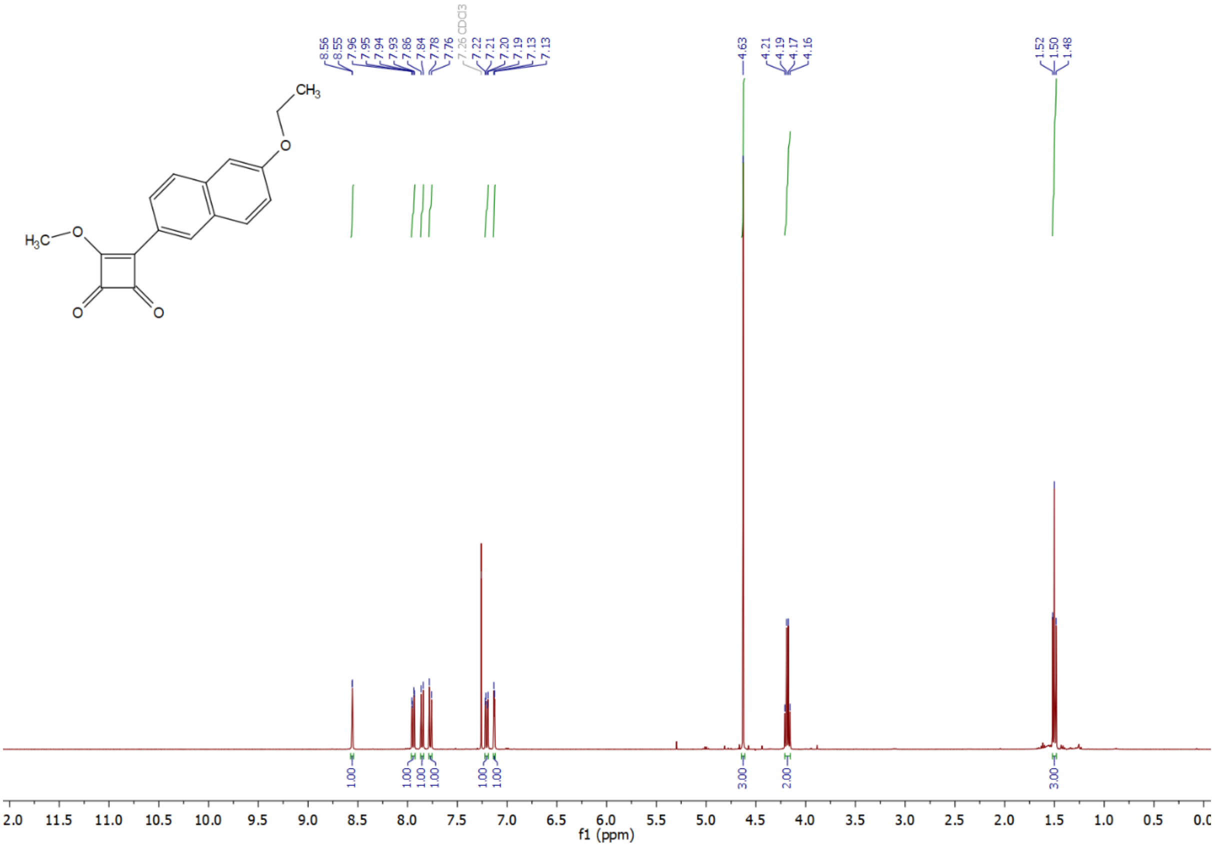


**Fig S103.** $^{13}$C NMR spectra of **5w**

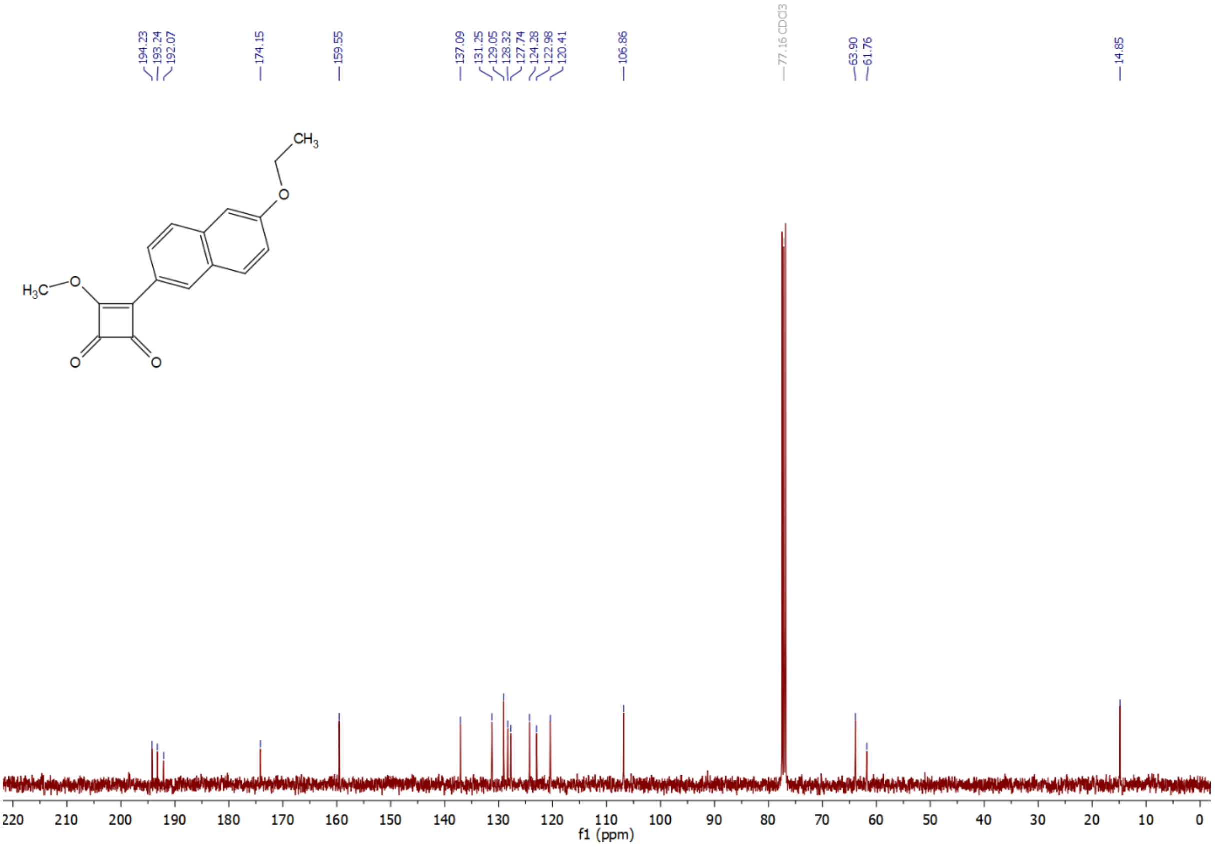

**Fig S104.** $^{1}$H NMR spectra of **6w**

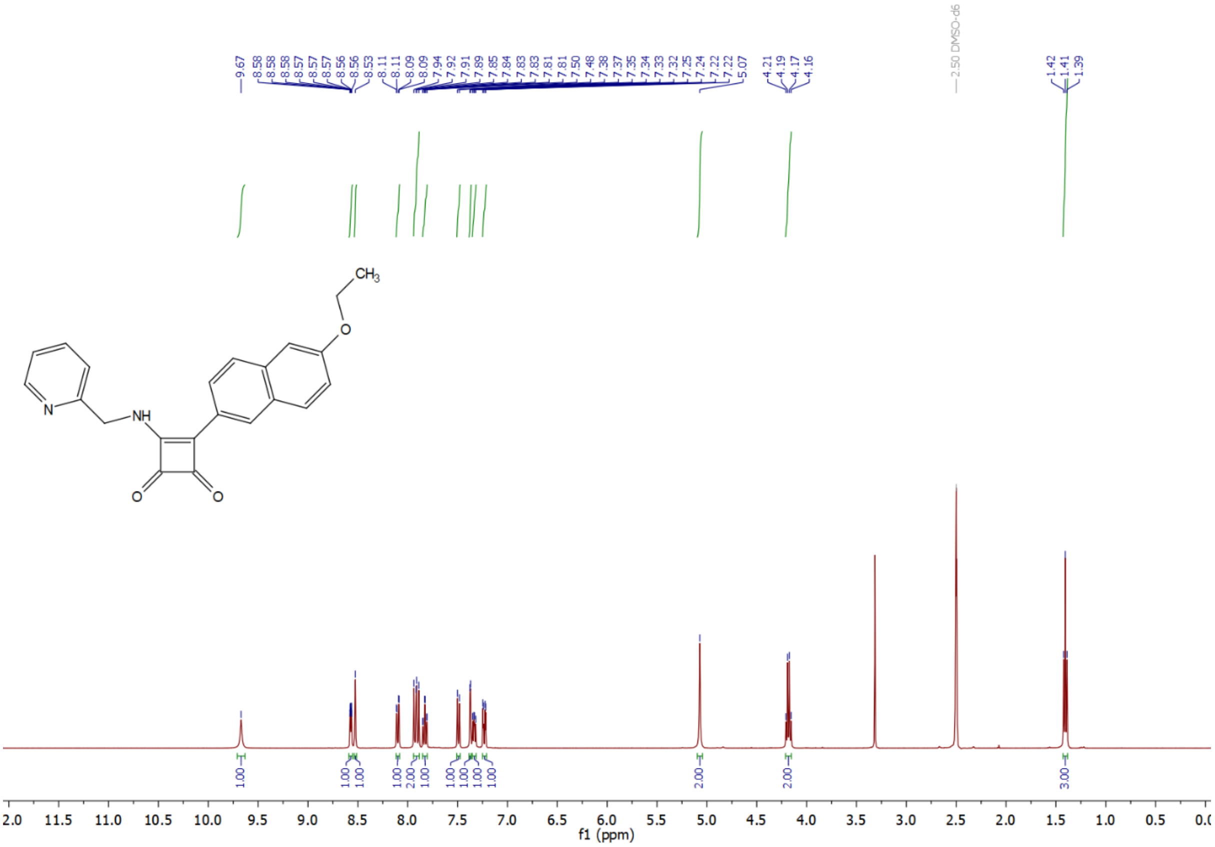

**Fig S105.** $^{13}$C NMR spectra of **6w**

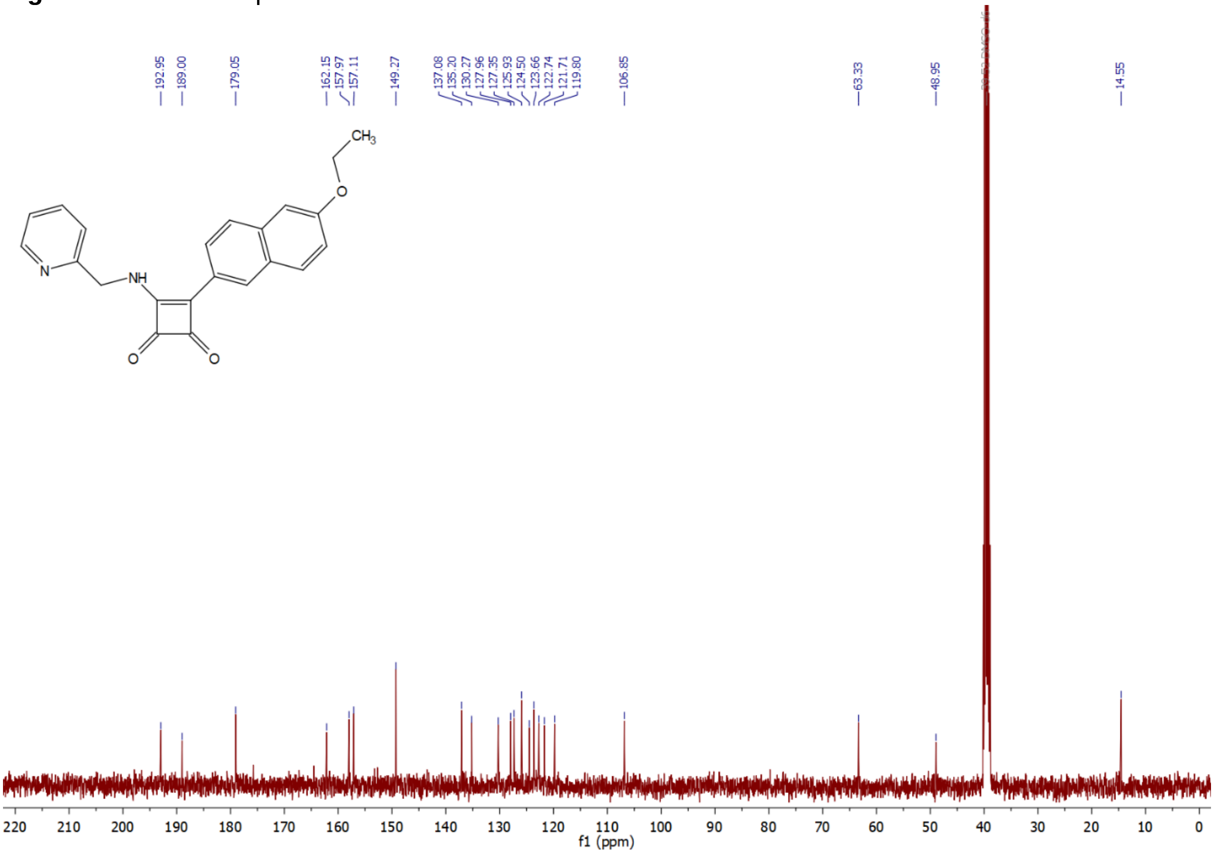

**Fig S106.** $^{1}$H NMR spectra of **5x**

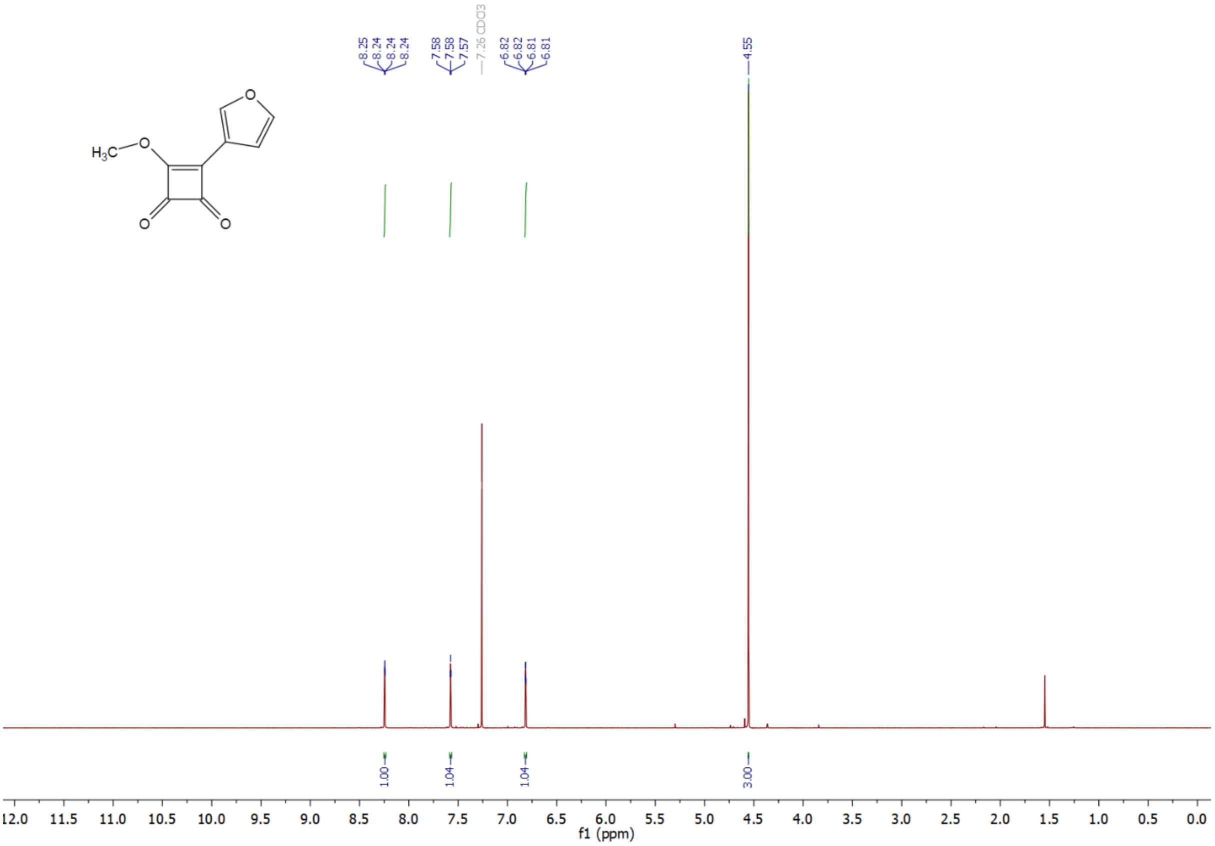


**Fig S107.** $^{13}$C NMR spectra of **5x**

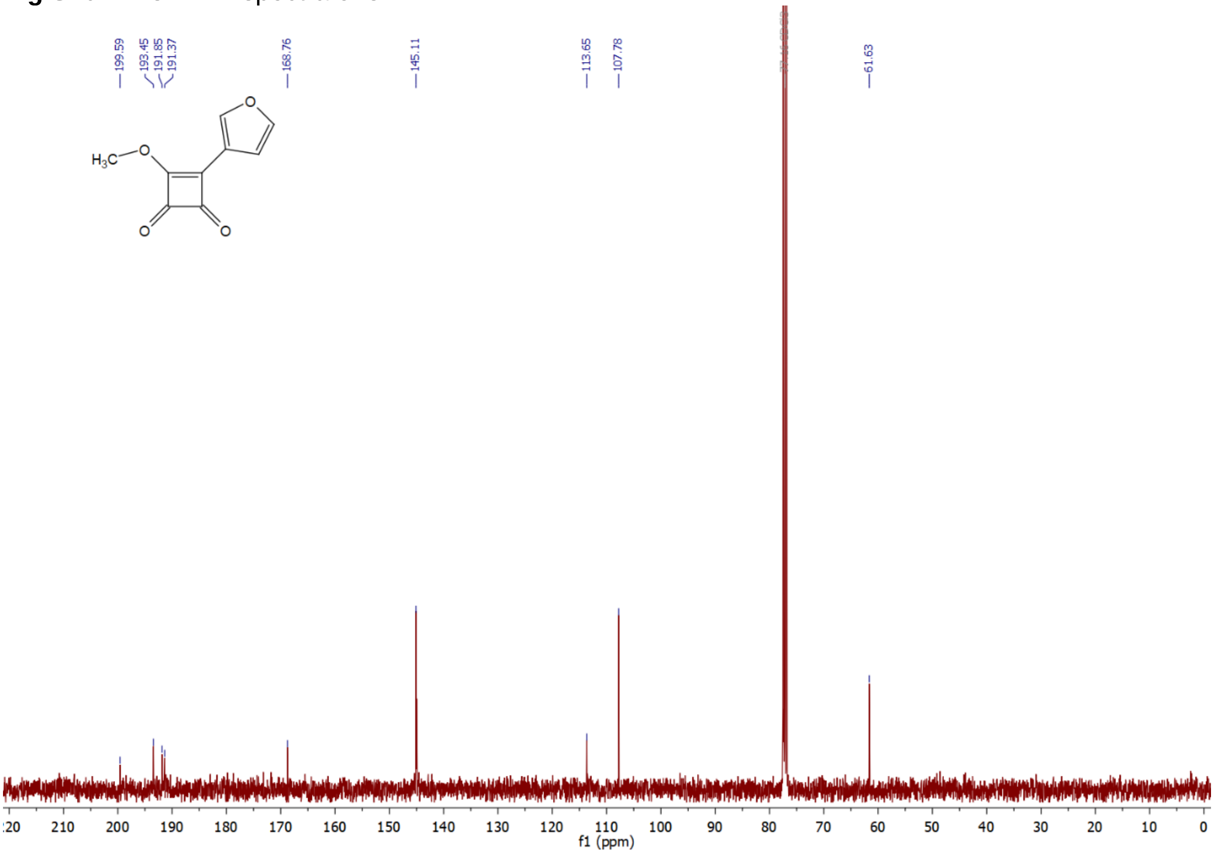

**Fig S108.** $^{1}$H NMR spectra of **6x**

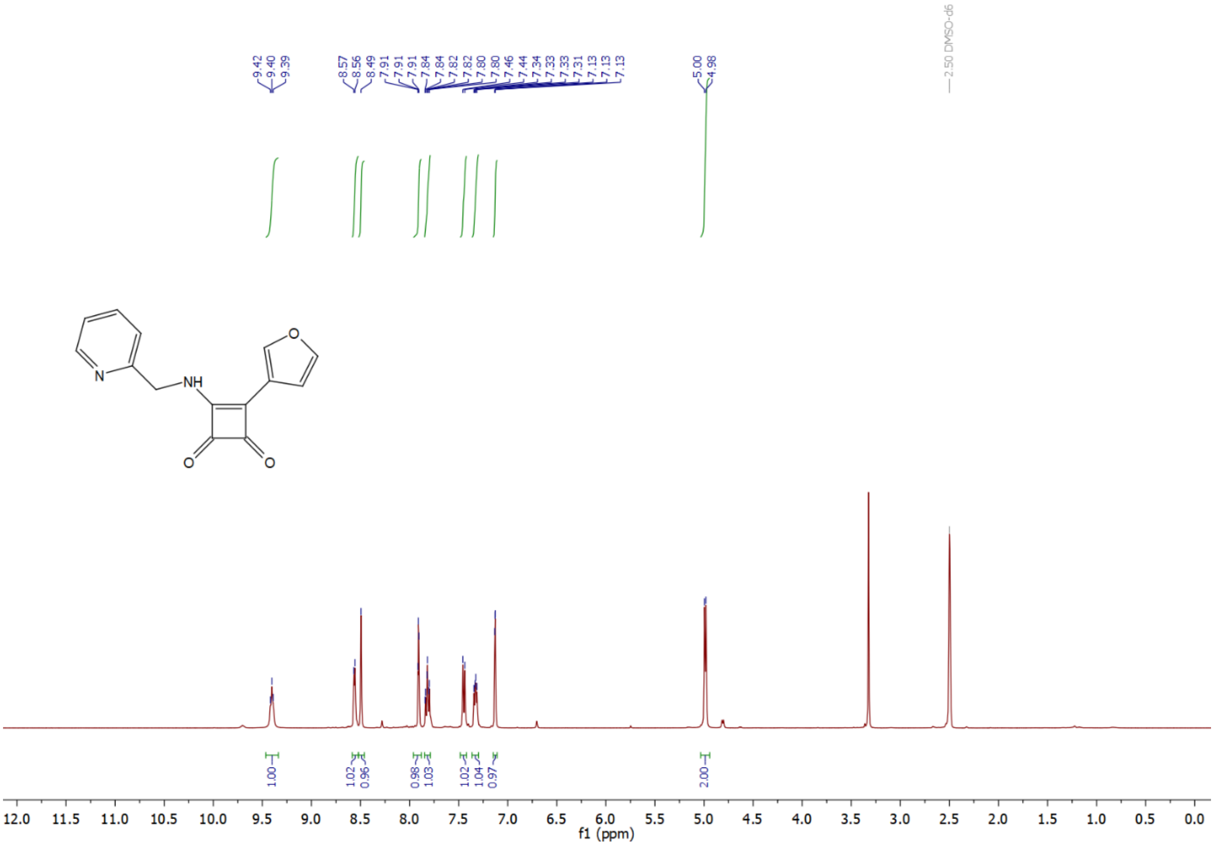


**Fig S109.** $^{13}$C NMR spectra of **6x**

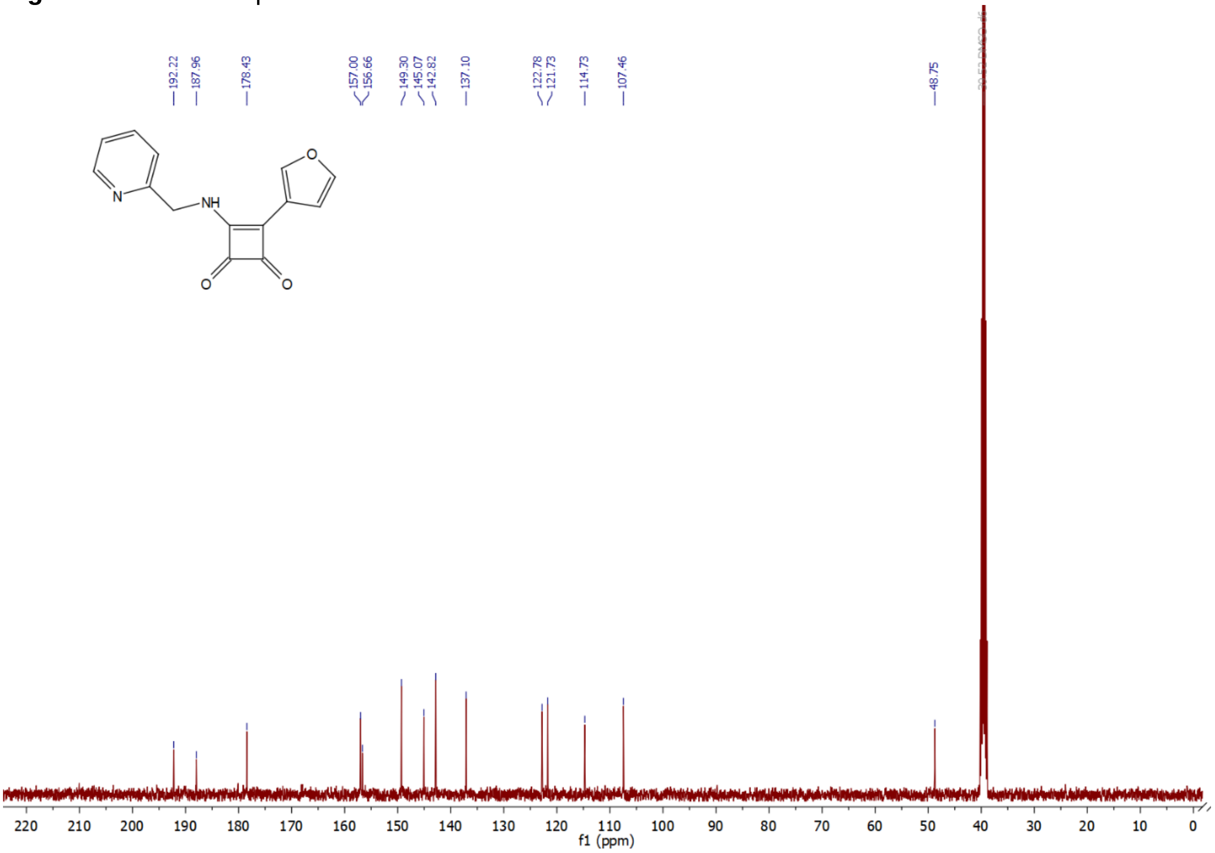

**Fig S110.** $^{1}$H NMR spectra of **5y**

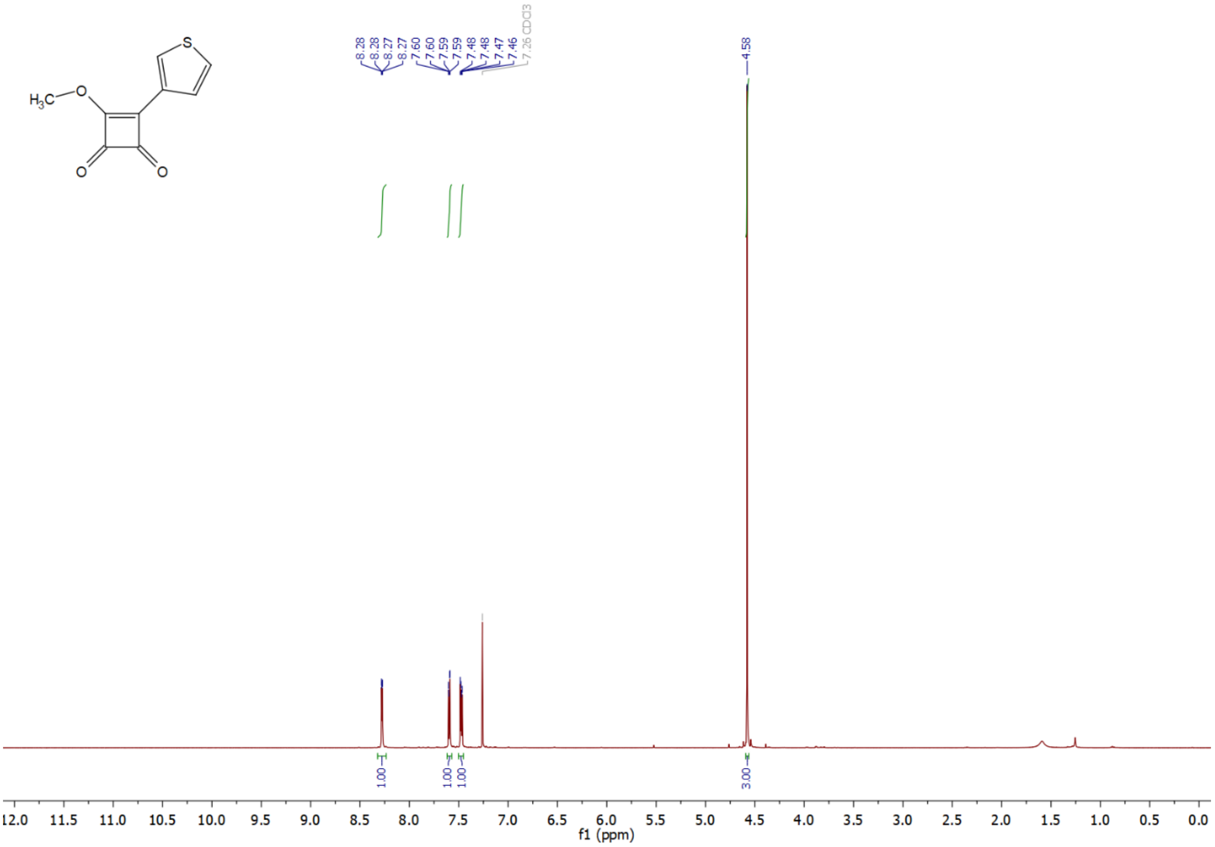


**Fig S111.** $^{13}$C NMR spectra of **5y**

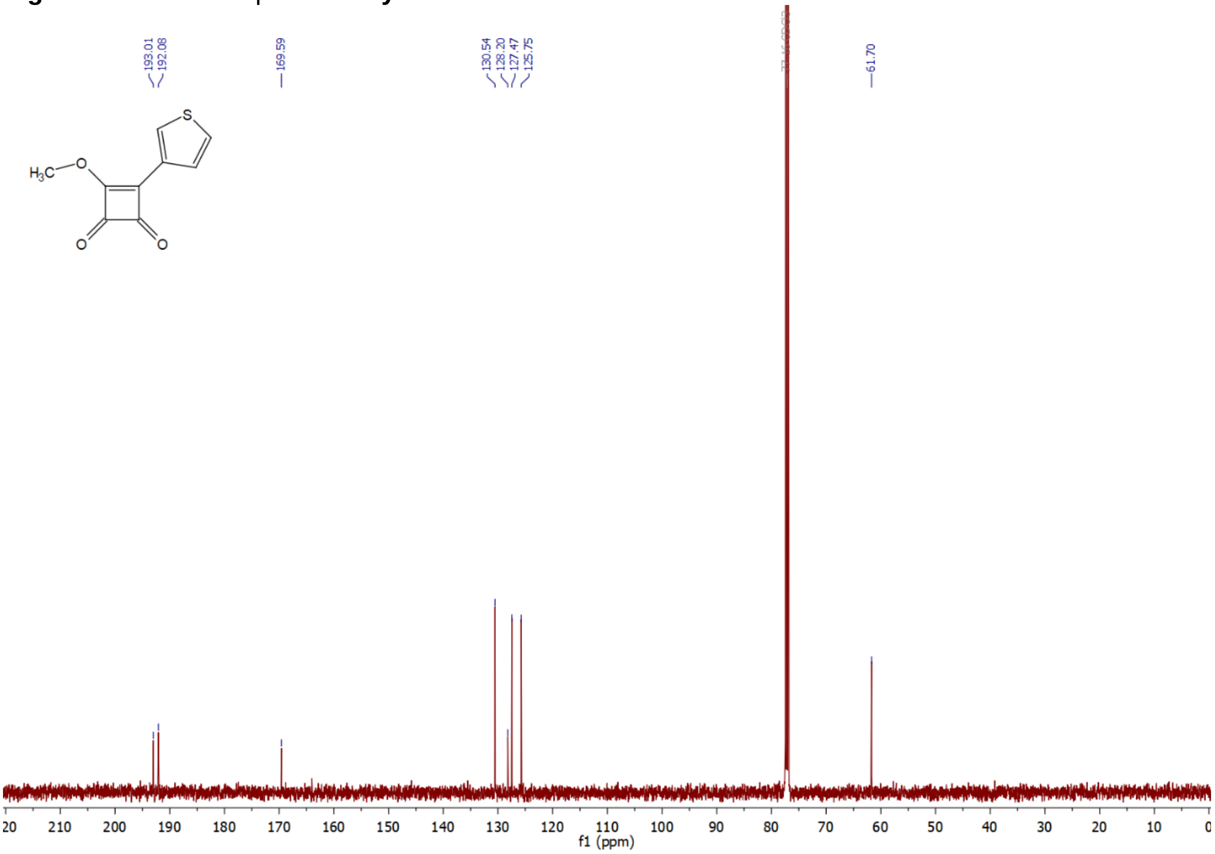

**Fig S112.** $^{1}H$ NMR spectra of **6y**

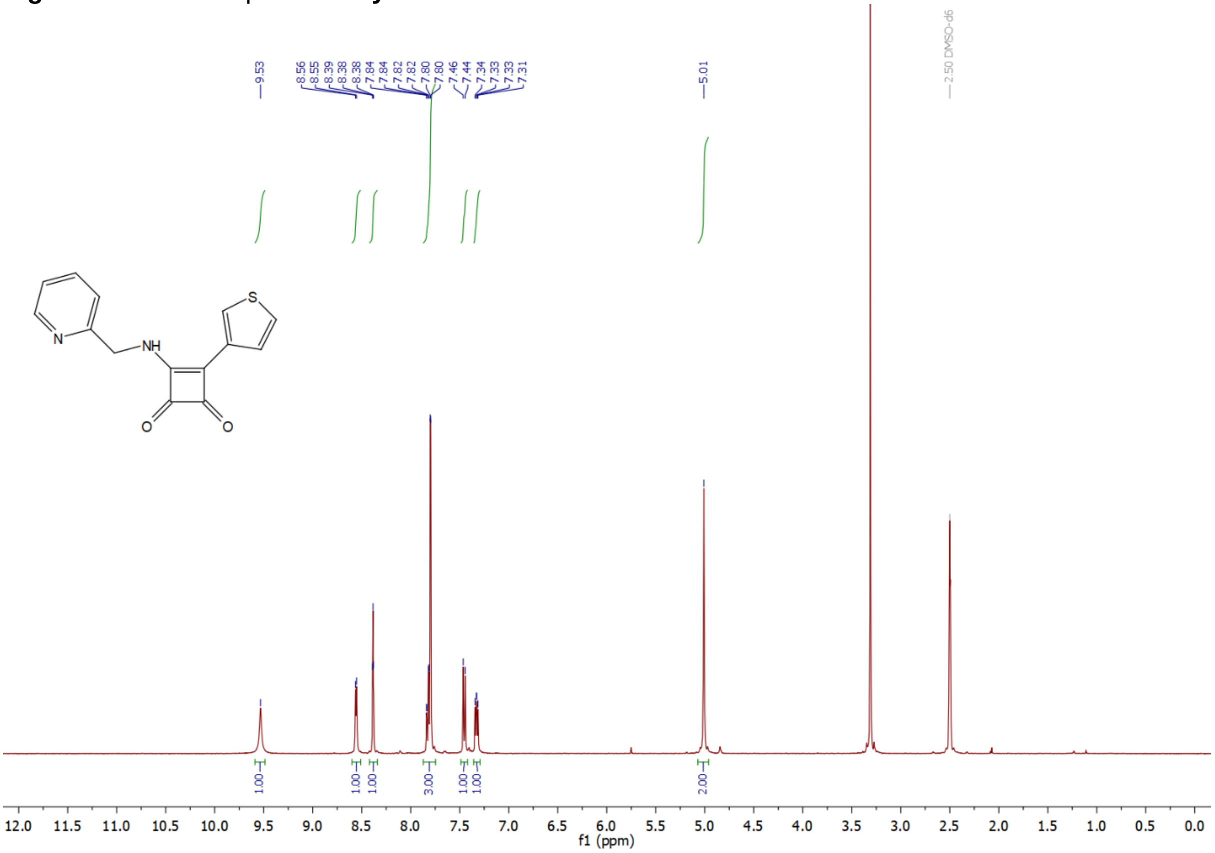

**Fig S113.** $^{13}C$ NMR spectra of **6y**

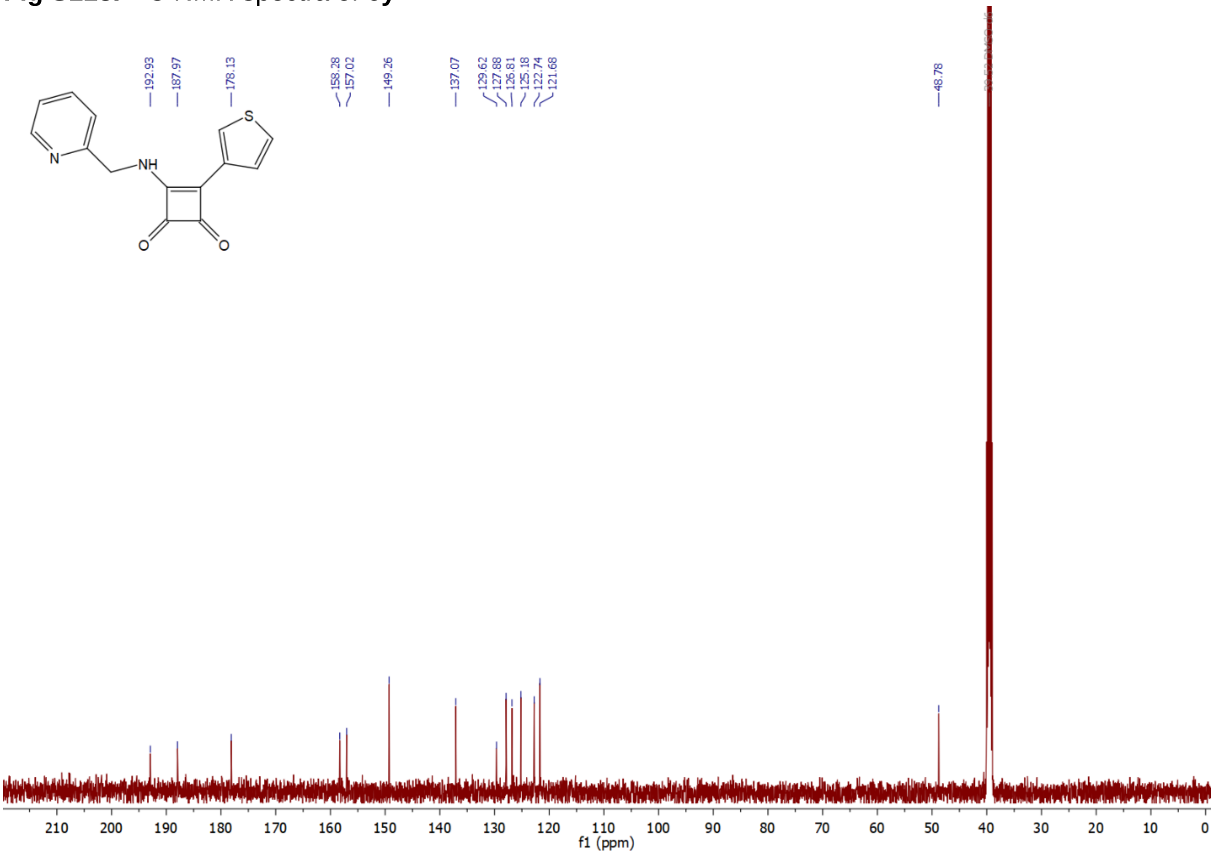

**Fig S114.** $^{1}$H NMR spectra of **5z**

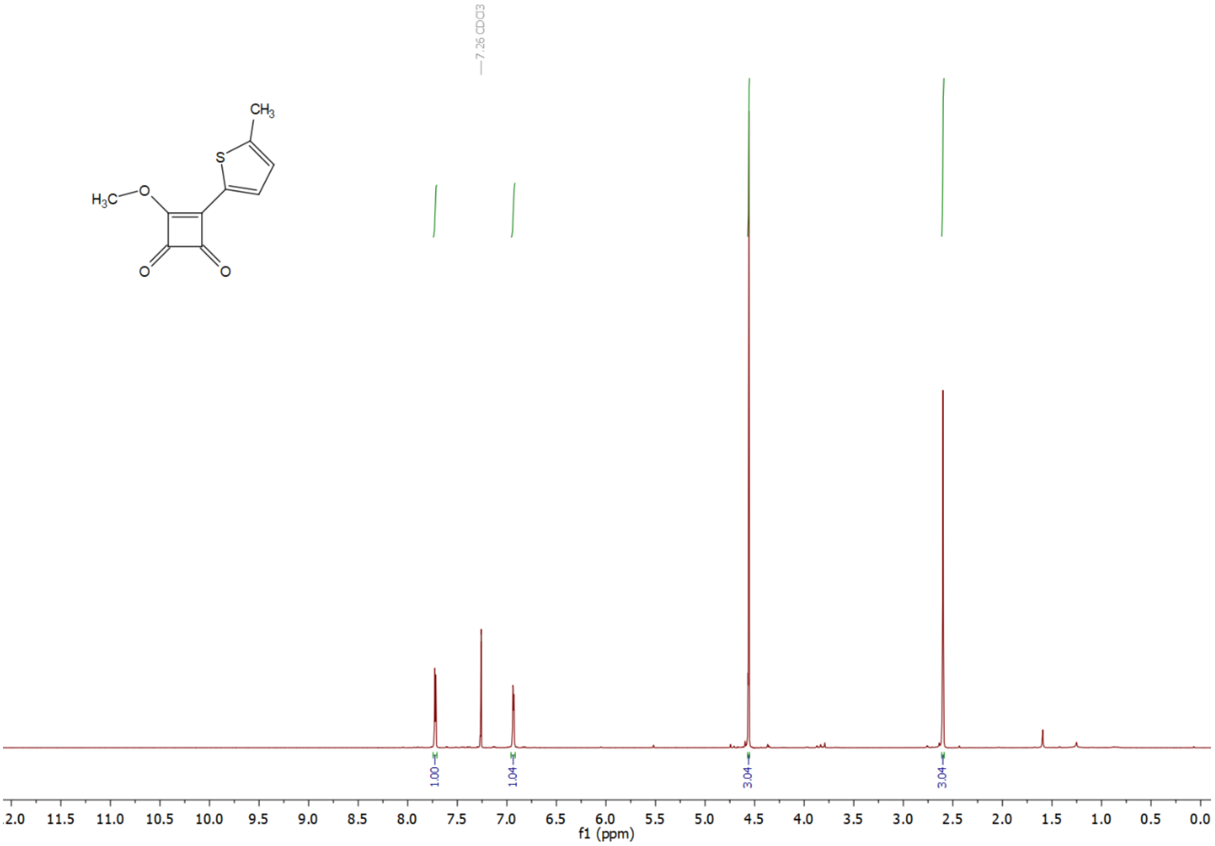

**Fig S115.** $^{13}$C NMR spectra of **5z**

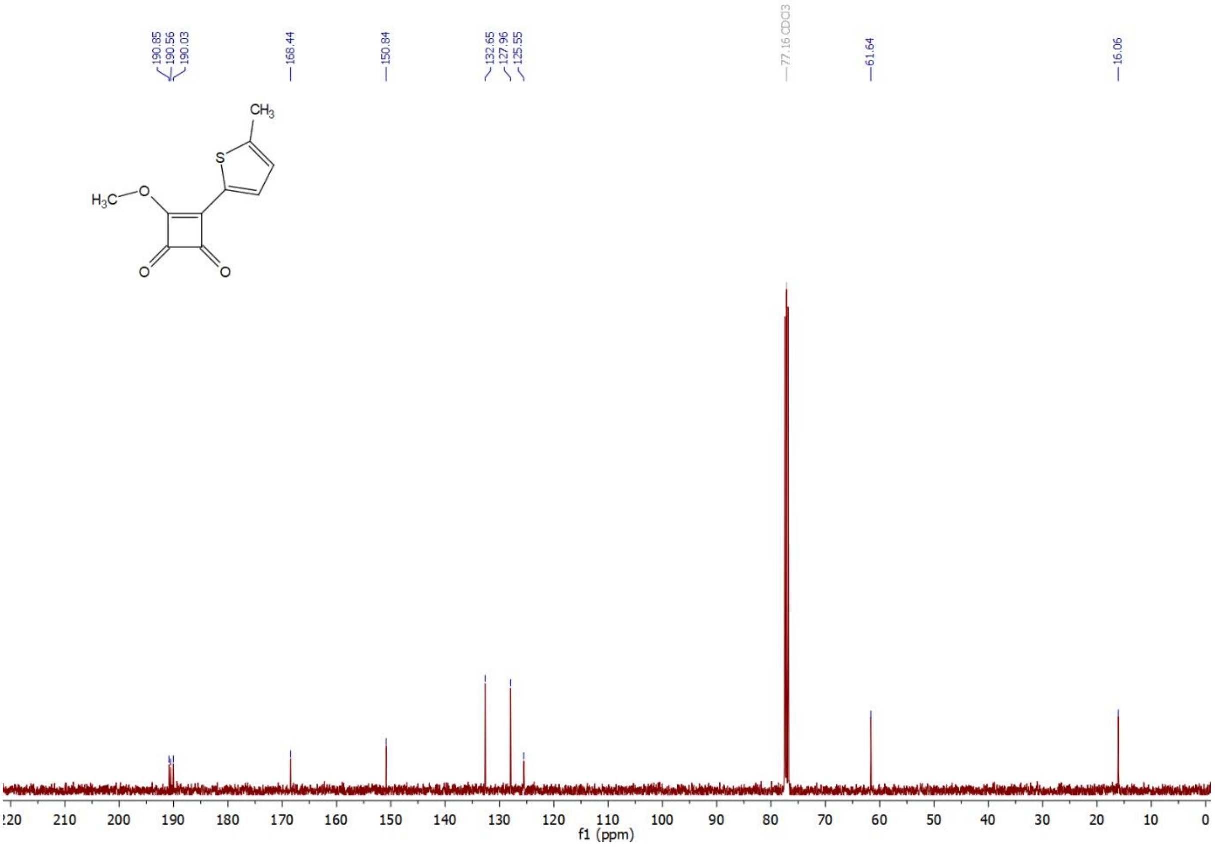

**Fig S116.** $^{1}$H NMR spectra of **6z**

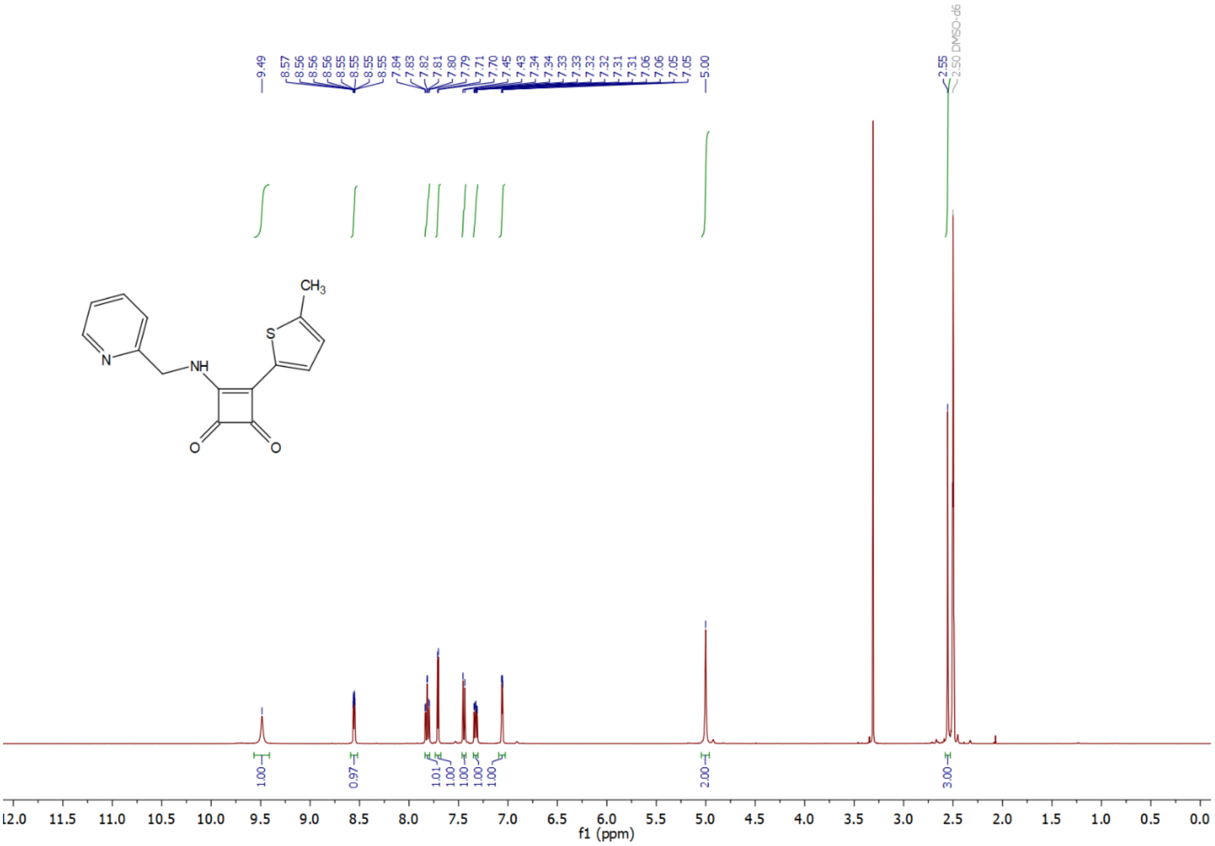

**Fig S117.** $^{13}$C NMR spectra of **6z**

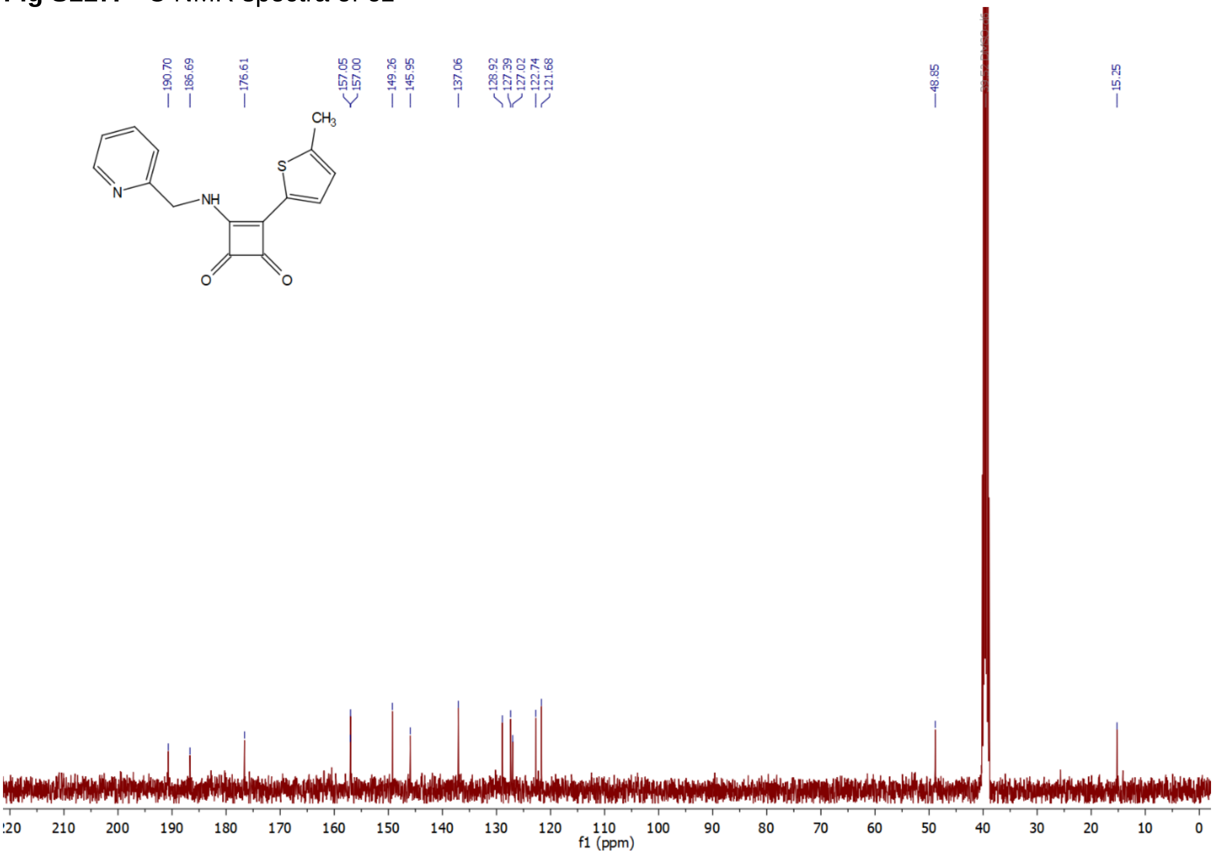

**Fig S118.** $^{1}H$ NMR spectra of **5aa**

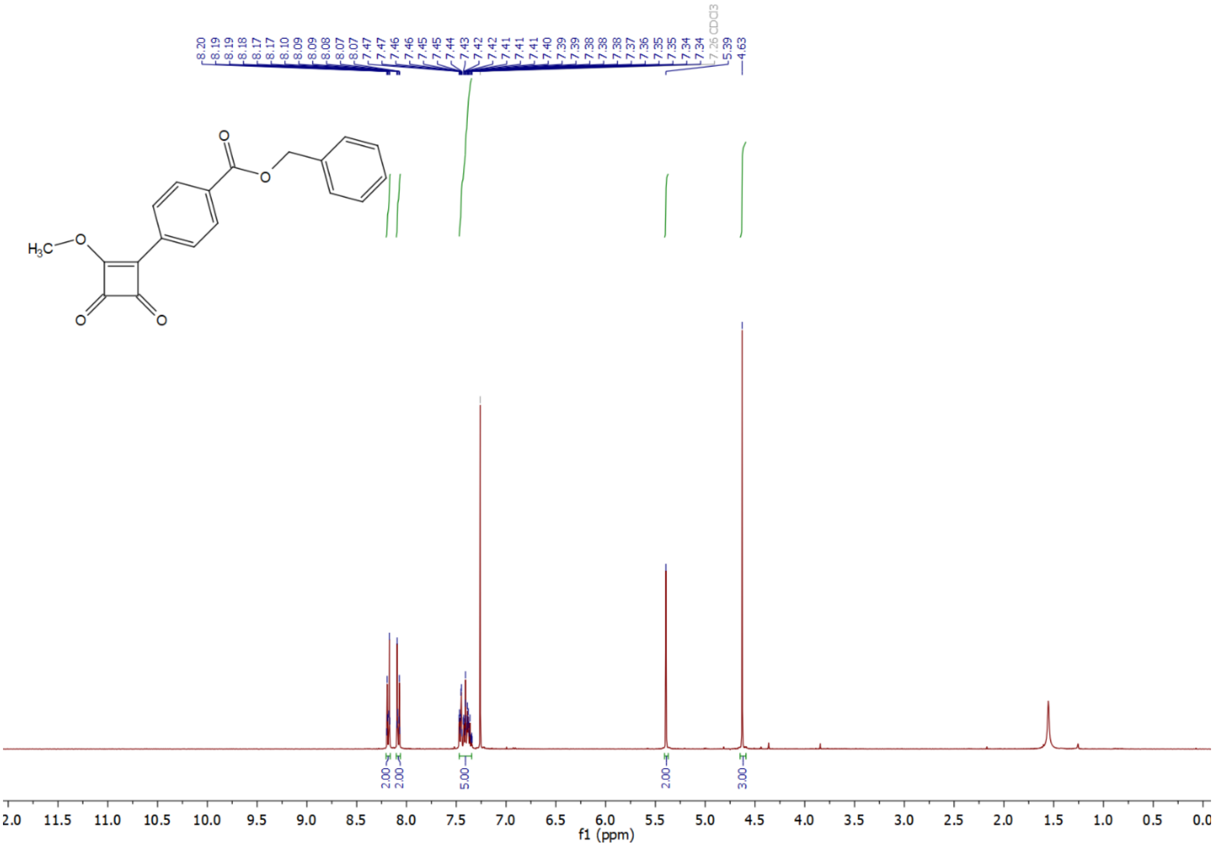

**Fig S119.** $^{1}H$ NMR spectra of **5ab**

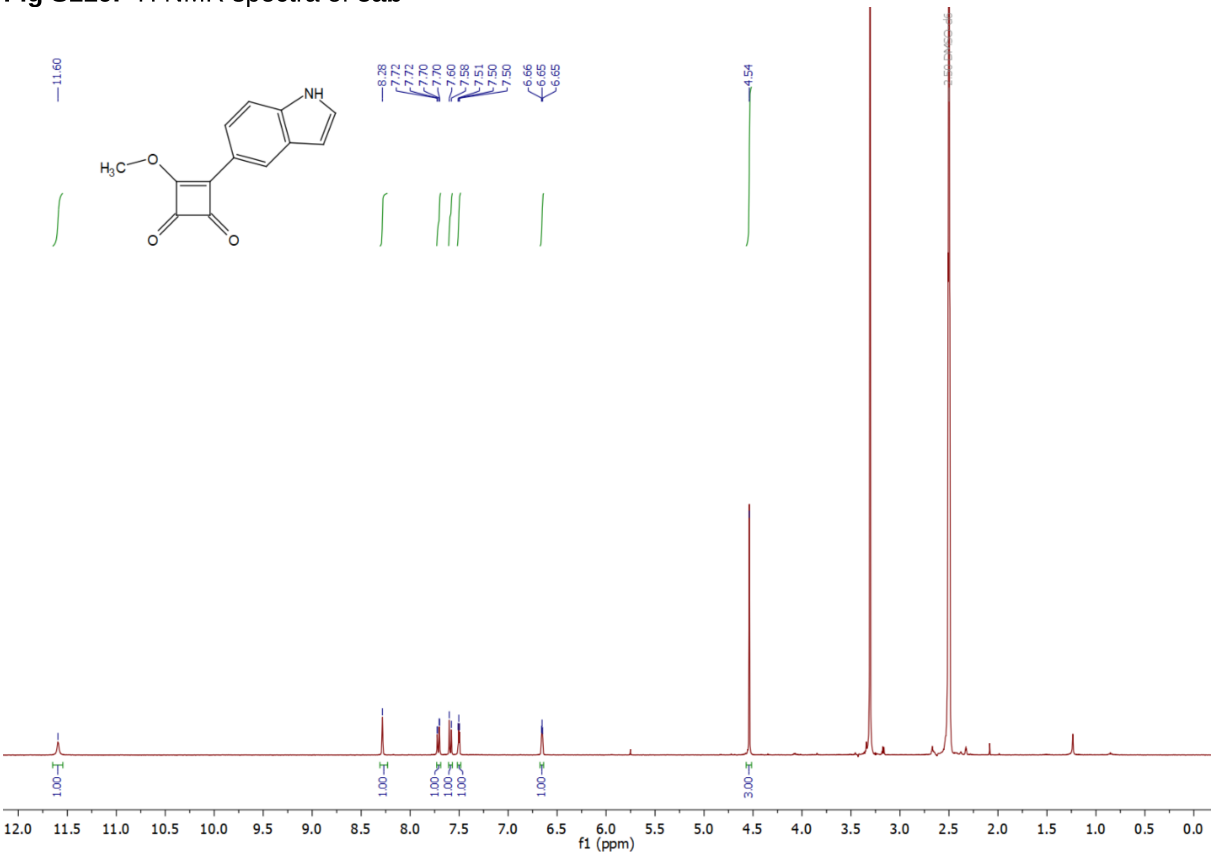

Supplement: Supplementary file 1 — ao4c04314_si_001.pdf [file ao4c04314_si_001.pdf]
