# Supplementary figures and images for: The Liebeskind–Srogl Cross-Coupling Reaction as a Crucial Step in the Synthesis of New Squaramide-Based Antituberculosis Agents
Source: ACS Omega. 2024 Jul 29;9(32):34808–28. doi: 10.1021/acsomega.4c04314 (PMC11325506; doi:10.1021/acsomega.4c04314)

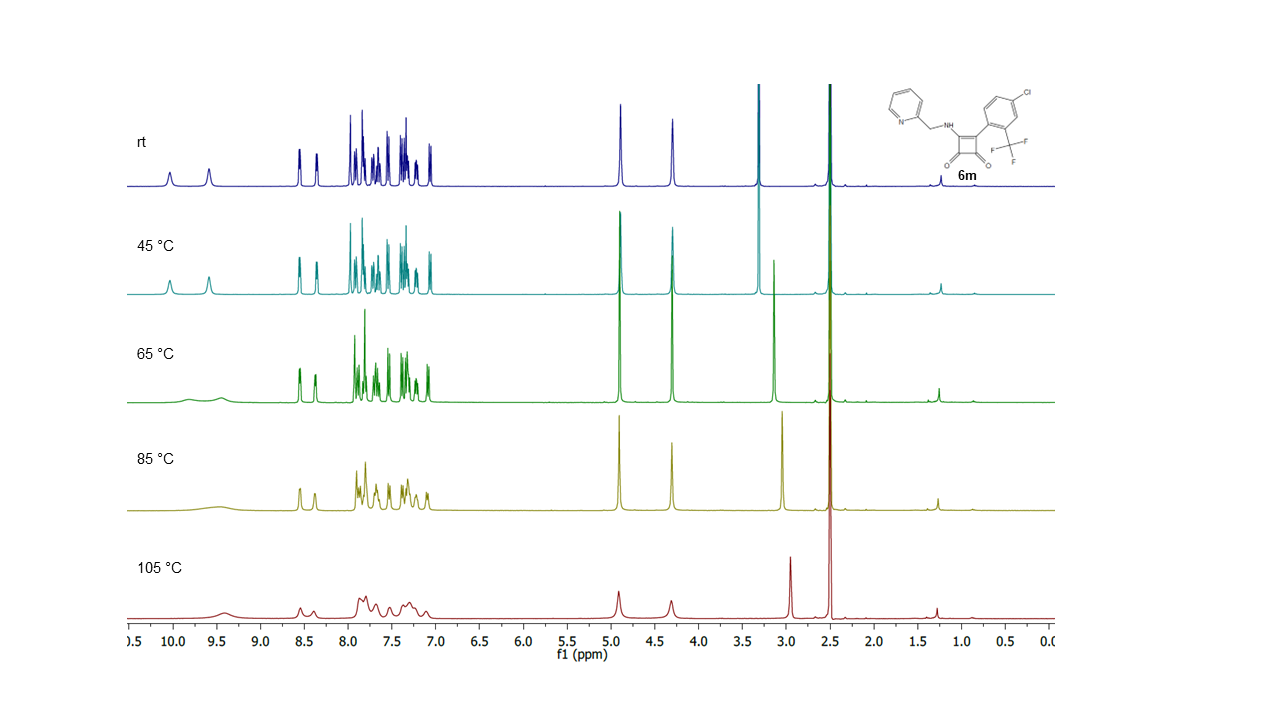

Supplement: Supplementary file 2 — ao4c04314_si_002.zip [file ao4c04314_si_002.zip › Obrázky rotamery/6m.tif]

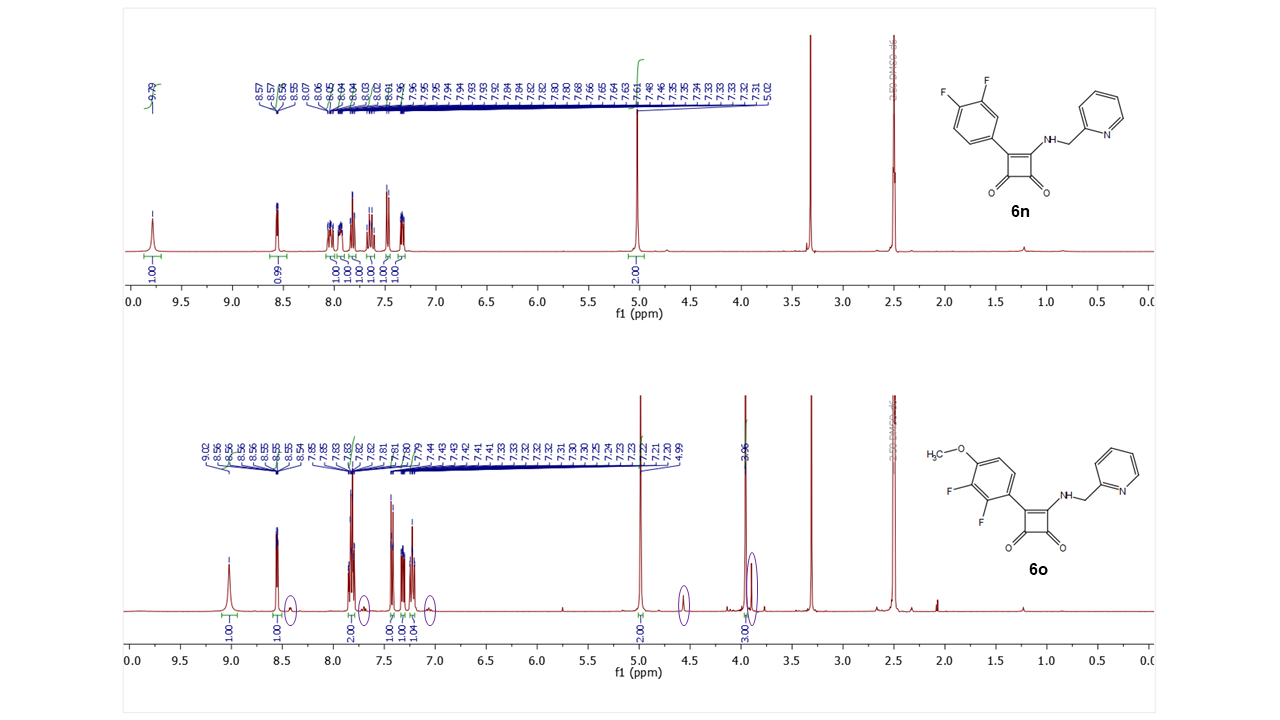

Supplement: Supplementary file 2 — ao4c04314_si_002.zip [file ao4c04314_si_002.zip › Obrázky rotamery/6n+6o.tif]

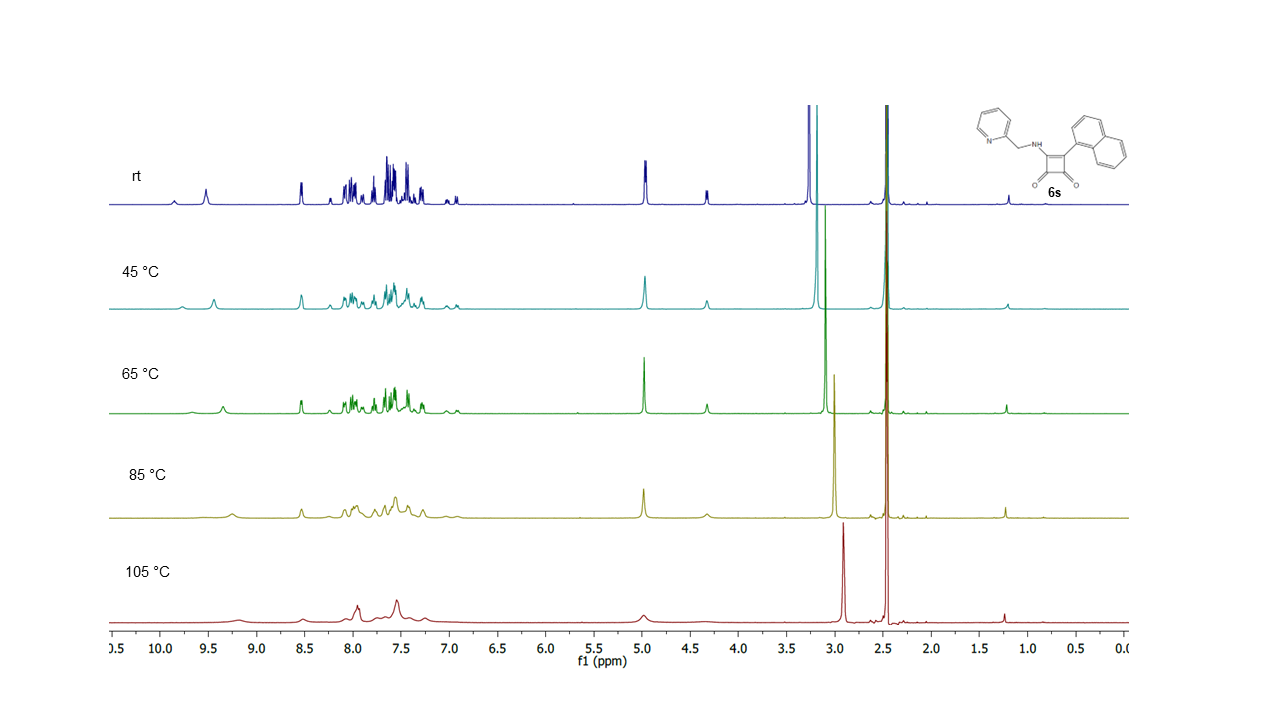

Supplement: Supplementary file 2 — ao4c04314_si_002.zip [file ao4c04314_si_002.zip › Obrázky rotamery/6s.tif]

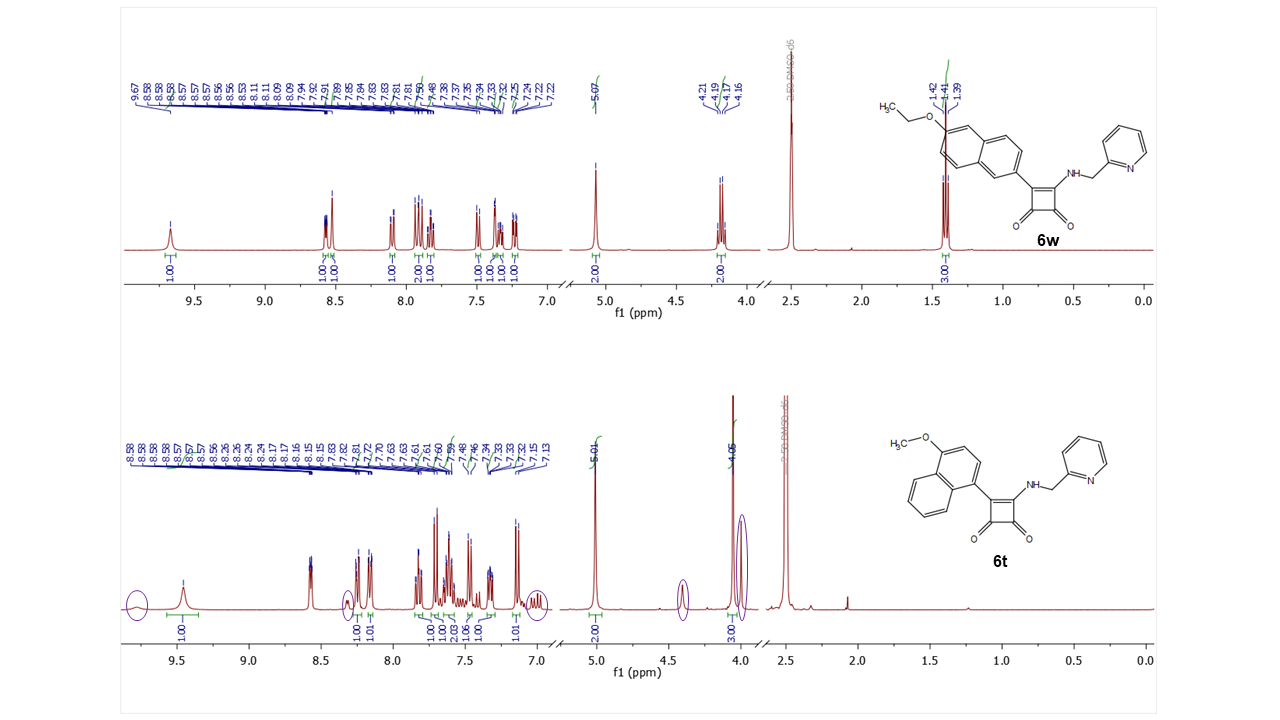

Supplement: Supplementary file 2 — ao4c04314_si_002.zip [file ao4c04314_si_002.zip › Obrázky rotamery/6w+6t.tif]
